# ﻿Five times over: 42 new *Angustopila* species highlight Southeast Asia’s rich biodiversity (Gastropoda, Stylommatophora, Hypselostomatidae)

**DOI:** 10.3897/zookeys.1147.93824

**Published:** 2023-02-13

**Authors:** Barna Páll-Gergely, András Hunyadi, Jaap J. Vermeulen, Jozef Grego, Chirasak Sutcharit, Alexander Reischütz, Pongrat Dumrongrojwattana, Zoltán Botta-Dukát, Aydin Örstan, Judit Fekete, Adrienne Jochum

**Affiliations:** 1 Centre for Agricultural Research, Plant Protection Institute, Eötvös Loránd Research Network, Herman Ottó út 15, H-1022 Budapest, Hungary Centre for Agricultural Research, Plant Protection Institute Budapest Hungary; 2 Adria sétány 10G 2/5., H-1148 Budapest, Hungary Unaffiliated Budapest Hungary; 3 JK Art and Science, Lauwerbes 8, 2318 AT Leiden, Netherlands JK Art and Science Leiden Netherlands; 4 Horná Mičiná 219, SK-97401 Banská Bystrica, Slovakia Unaffiliated Banská Bystrica Slovakia; 5 Animal Systematic Research Unit, Department of Biology, Faculty of Science, Chulalongkorn University, Bangkok 10330, Thailand Chulalongkorn University Bangkok Thailand; 6 Puechhaimgasse 52, A-3580 Horn, Austria Unaffiliated Horn Austria; 7 Department of Biology, Faculty of Science, Burapha University, 169 Longhardbangsaen Road, Muang District, Chonburi, 20131, Thailand Burapha University Chonburi Thailand; 8 Centre for Ecological Research, Institute of Ecology and Botany, Alkotmány 2–4, H-2600, Vácrátót, Hungary Centre for Ecological Research, Institute of Ecology and Botany Vácrátót Hungary; 9 12501 Milestone Manor Lane, Germantown, Maryland, 20876, USA Unaffiliated Germantown United States of America; 10 University of Pannonia, Centre of Natural Science, Research Group of Limnology, Egyetem u. 10, H-8200 Veszprém, Hungary University of Pannonia Veszprém Hungary; 11 Centre for Ecological Research, Institute of Aquatic Ecology, Department of Tisza Research, 18/c Bem square, H-4026 Debrecen, Hungary Centre for Ecological Research, Institute of Aquatic Ecology Debrecen Hungary; 12 Naturhistorisches Museum der Burgergemeinde Bern, CH-3005 Bern, Switzerland Naturhistorisches Museum der Burgergemeinde Bern Bern Switzerland; 13 Institute of Ecology and Evolution, University of Bern, CH-3012 Bern, Switzerland University of Bern Bern Switzerland; 14 Senckenberg Forschungsinstitut und Naturmuseum, 60325 Frankfurt am Main, Germany Senckenberg Forschungsinstitut und Naturmuseum Frankfurt am Main Germany

**Keywords:** Biodiversity, microsnails, systematics, taxonomy

## Abstract

The Southeast Asian genus *Angustopila*, currently comprising 13 nominal species, encompasses the world’s tiniest land snails. This work shows that there are far more species than previously suspected, and that this genus is in fact, a very speciose group of tiny snails widely distributed in Southeast Asia. *Angustopila* is revised based on type material of known species as well as 211 samples newly collected in China, Laos, Myanmar, Thailand and Vietnam. Altogether, 53 species and one subspecies are recognised, of which 42 species and subspecies are new to science: *A.akrodon* Páll-Gergely & Hunyadi, **sp. nov.**, *A.apiaria* Páll-Gergely & Hunyadi, **sp. nov.**, *A.apiostoma* Páll-Gergely & Vermeulen, **sp. nov.**, *A.apokritodon* Páll-Gergely & Hunyadi, **sp. nov.**, *A.antidomedon* Páll-Gergely & Hunyadi, **sp. nov.**, *A.babel* Páll-Gergely & Vermeulen, **sp. nov.**, *A.bathyodon* Páll-Gergely & Hunyadi, **sp. nov.**, *A.bidentata* Páll-Gergely & Jochum, **sp. nov.**, *A.cavicola* Páll-Gergely & Dumrongrojwattana, **sp. nov.**, *A.cicatricosa* Páll-Gergely & Vermeulen, **sp. nov.**, *A.coprologosuninodus* Páll-Gergely & Grego, **ssp. nov.**, *A.erawanica* Páll-Gergely & Dumrongrojwattana, **sp. nov.**, *A.fratermajor* Páll-Gergely & Vermeulen, **sp. nov.**, *A.fraterminor* Páll-Gergely & Vermeulen, **sp. nov.**, *A.gracilis* Páll-Gergely & Hunyadi, **sp. nov.**, *A.halongensis* Páll-Gergely & Vermeulen, **sp. nov.**, *A.hyron* Páll-Gergely & Vermeulen, **sp. nov.**, *A.maasseni* Páll-Gergely & Vermeulen, **sp. nov.**, *A.majuscula* Páll-Gergely & Hunyadi, **sp. nov.**, *A.margaritarion* Páll-Gergely & Hunyadi, **sp. nov.**, *A.megastoma* Páll-Gergely & Vermeulen, **sp. nov.**, *A.occidentalis* Páll-Gergely & Hunyadi, **sp. nov.**, *A.oostoma* Páll-Gergely & Vermeulen, **sp. nov.**, *A.papaver* Páll-Gergely & Hunyadi, **sp. nov.**, *A.parallela* Páll-Gergely & Hunyadi, **sp. nov.**, *A.prolixa* Páll-Gergely & Hunyadi, **sp. nov.**, *A.pusilla* Páll-Gergely & Hunyadi, **sp. nov.**, *A.pustulata* Páll-Gergely & Hunyadi, **sp. nov.**, *A.quadridens* Páll-Gergely & Vermeulen, **sp. nov.**, *A.rara* Páll-Gergely & Hunyadi, **sp. nov.**, *A.reticulata* Páll-Gergely & Hunyadi, **sp. nov.**, *A.somsaki* Páll-Gergely & Hunyadi, **sp. nov.**, *A.steffeki* Páll-Gergely & Grego, **sp. nov.**, *A.tetradon* Páll-Gergely & Hunyadi, **sp. nov.**, *A.thersites* Páll-Gergely & Vermeulen, **sp. nov.**, *A.tonkinospiroides* Páll-Gergely & Vermeulen, **sp. nov.**, *A.tridentata* Páll-Gergely & Hunyadi, **sp. nov.**, *A.tweediei* Páll-Gergely & Hunyadi, **sp. nov.**, *A.uvula* Páll-Gergely & Hunyadi, **sp. nov.**, *A.vandevenderi* Páll-Gergely & Jochum, **sp. nov.**, *A.vitrina* Páll-Gergely & Hunyadi, **sp. nov.**, *A.vomer* Páll-Gergely & Hunyadi, **sp. nov.**, *A.werneri* Páll-Gergely & Hunyadi, **sp. nov.**

*Angustopilasubelevata* Páll-Gergely & Hunyadi, 2015 is moved to the synonymy of *Angustopilaelevata* (F. G. Thompson & Upatham, 1997), and *A.singuladentis* Inkhavilay & Panha, 2016 is a junior synonym of *A.fabella* Páll-Gergely & Hunyadi, 2015. Three species, namely *A.elevata*, *A.fabella* and *A.szekeresi*, are widespread over several hundred kilometres while some other species (*A.huoyani*, *A.parallela***sp. nov.**, *A.cavicola***sp. nov.**) are known from just two sites a few hundred kilometres apart. All others are small range or single-site endemics. The reproductive anatomy of *A.erawanica***sp. nov.** is described.

## ﻿Introduction

A large fraction of tropical terrestrial snails are “microsnails”, possessing shells that are smaller than 5 mm (Panha and Burch 2005). When the majority of known Southeast Asian snail shells had been collected and described at the end of the 19^th^ century, mostly larger bodied species found their way into private and museum collections via adventurous collectors, travellers, missionaries, and soldiers. However, since microsnails were little known, their numbers have remained a mystery, and their true diversity is still uncertain. So far, the vast majority of microsnail species of Southeast Asia are known from their type localities only. Thus, it is largely unknown whether they are all strictly endemic or if widely distributed species are among them. Although a few studies (Páll-Gergely et al. 2015b, 2021) have shown that some Southeast Asian terrestrial snail species are widely distributed over several hundreds of kilometres, this condition has not yet been observed amongst the smallest microsnails. Probably the only known example is *Bensonellaplicidens* (Benson, 1849), which was claimed to have a disjunct distribution in the Himalaya and in East Asia, including Japan and eastern China (see Pilsbry 1918; Gittenberger et al. 2021). However, recent study has shown that the Himalayan and East Asian species belong to two distinct species (Páll-Gergely and White 2023).

*Angustopila* Jochum, Slapnik & Páll-Gergely, 2014 is a genus of land snails known to include the tiniest members of terrestrial gastropods (Páll-Gergely et al. 2015a, 2022; Dumrongrojwattana et al. 2021). Its range spans from Thailand and Vietnam to southern China from twelve localities. So far, only twelve nominal species are known, which are mostly reported from their type localities only.

The taxonomic history of this genus started at the end of the 20^th^ century. Thompson and Upatham (1997) described two species of *Systenostoma* Bavay & Dautzenberg, 1909 from Thailand, *S.concava* Thompson & Upatham, 1997 and *S.elevata* Thompson & Upatham, 1997. In addition to these species, they recognised three Vietnamese *Systenostoma*: *S.defixa* Bavay & Dautzenberg, 1912, *S.pauperrima* (Bavay & Dautzenberg, 1909) and *S.pulverea* (Bavay & Dautzenberg, 1909). Later, two Thai species, *S.tamlod* Panha & Burch, 2002 and *S.edentatum* Panha & Burch, 2002 were described. Jochum et al. (2014) introduced *Tonkinospira* Jochum, Slapnik & Páll-Gergely, 2014 as a replacement name for *Systenostoma* Bavay & Dautzenberg, 1909 (non *Systenostoma*Marsson 1887, Bryozoa), and included four species: *Tonkinospiradefixa*, *T.depressa* (S. Jaeckel, 1950), *T.pauperrima* and *T.pulverea*. *Tonkinospira* currently comprises ten species (Páll-Gergely et al. 2019). *Angustopila*, a genus differing from *Tonkinospira* by its smaller shell size, more elevated spire, slightly reflexed peristome, and general presence of dentition within the aperture (Jochum et al. 2014), was described to include *A.concava*, *A.elevata*, *A.huoyani* Jochum, Slapnik & Páll-Gergely, 2014 (from China), *A.neglecta* (van Benthem-Jutting, 1961) (Malay Peninsula) and *A.tamlod*, which was designated as the type species. Further species subsequently included in *Angustopila* are the following: *A.dominikae* Páll-Gergely & Hunyadi, 2015, *A.fabella* Páll-Gergely & Hunyadi, 2015, *A.subelevata* Páll-Gergely & Hunyadi, 2015, *A.szekeresi* Páll-Gergely & Hunyadi, 2015 (all four from China), *A.singuladentis* Inkhavilay & Panha, 2016 (Laos) and *A.stochi* Páll-Gergely & Jochum, 2017 (Vietnam). *Angustopilastochi* and *Paraboysidianeglecta* were moved to *Clostophis* Benson, 1860 due to their larger shell and denser spiral striation (Páll-Gergely et al. 2020; Páll-Gergely and Hunyadi 2022). Later, three additional species, *Angustopilapallgergelyi* Dumrongrojwattana, Chuenit & Wongkamhaeng, 2021, *Angustopilapsammion* Páll-Gergely, Vermeulen & Anker, 2022 and *A.coprologos* Páll-Gergely, Jochum & Hunyadi, 2022 were described (the latter two in Páll-Gergely et al. 2022). Finally, Das et al. (2021) assigned *Acmellamilium* (Benson, 1853) (originally described as *Cyclostoma*) to *Angustopila*, increasing the number of *Angustopila* species to 13.

The higher classification of Southeast Asian pupilloid microsnail genera is problematic. *Hypselostoma* Benson, 1856 and *Boysidia* Ancey, 1881 were classified in the family Pupillidae by Pilsbry (1917). Later, Zilch (1959) erected Hypselostomatinae Zilch, 1959 and Aulacospirinae Zilch, 1959 within Chondrinidae. These subfamilies were synonymized with Gastrocoptinae by Gittenberger (1973). Schileyko (1998) separated Hypselostomatidae as a distinct family and treated Aulacospirinae as its synonym, while Bouchet et al. (2017) treated Hypselostomatidae as a synonym of Gastrocoptidae Pilsbry, 1918. In recent publications, *Angustopila* Jochum, Slapnik & Páll-Gergely, 2014 and other probably related microsnail genera were classified in various families such as Pupillidae Turton, 1831 (e.g., Panha and Burch 2002) and Vertiginidae Fitzinger, 1833 (e.g., Inkhavilay et al. 2019). Currently, only one molecular systematic study has been conducted on the family (Tongkerd et al. 2004), revealing that the ‘hypselostomatid’ genera (*Anauchen* Pilsbry, 1917, *Aulacospira* Möllendorff, 1890, *Gyliotrachela*, *Hypselostoma*, *Krobylos* Panha & J. B. Burch, 2002) form a monophyletic clade distinct from Vertiginidae and Pupillidae. Unfortunately, no *Chondrina* Reichenbach, 1828 or *Gastrocopta* Wollaston, 1878 samples were used, which would have further clarified the relationship of ‘Hypselostomatidae’ with Chondrinidae and Gastrocoptidae. In conjunction with recent works (Jochum et al. 2014; Páll-Gergely et al. 2015a, 2017, 2022; Páll-Gergely and Hunyadi 2022), we treat *Angustopila* as a member of Hypselostomatidae.

In this work, we show that there are far more *Angustopila* species than previously suspected, and that this genus is in fact, a very species-rich group of snails. It becomes clear that these extremely tiny land snails are abundant and widely distributed in Southeast Asia and are not limited to certain areas. The present study is based on the examination of type material of all *Angustopila* species and 211 newly collected lots. Altogether, 53 species and one subspecies are recognised, of which 42 and the single subspecies herein, are described as new. We also show that three species have wide distributions, indicating that for the description of a new microsnail, it is critical to compare any possibly new species with species from a wide range to establish its novelty.

## ﻿Materials and methods

### ﻿Collecting methods

Rarely (pers. obs. PD), living snails were collected inside caves by picking them individually out of the sediment using fine forceps (see also Dumrongrojwattana et al. 2021). In all other cases, soil samples were either flotated in water (AH, JV) and the dried samples sieved, or they were directly sieved without floating the soil sample (AR). JG washed the cave mud through a large metal sieve installed over a nylon stocking for fine filtration. The dried samples were eventually sorted under stereo microscope.

### ﻿Handling and imaging of specimens

Shells were manually brushed clean of mud using wet, finely tapered brushes. The shells were viewed without coating under a low vacuum SEM (Hitachi Miniscope TM-1000 at Shinshu University, Matsumoto, Japan; Hitachi TM-4000 Plus SEM at the Research and Instrument Core Facility of the Faculty of Science, Eötvös Loránd University, Budapest, Hungary). Shell whorl number was counted to the nearest quarter whorl according to Kerney and Cameron (1979). Images were taken by B. Páll-Gergely if not stated otherwise.

### ﻿Examined characters

#### Shell size

All shells were measured using a Keyence Digital microscope. Spire height can vary from depressed (low convex (biscuit shaped)) to high conical, showing continuous variation across species from the most depressed to the highest shell. In each species, we indicate whether the shell is higher than wide, or wider than high, and for the measurements, we indicate the range of shell height to shell width as “H/D*100”.

For determining the size ranges, we averaged the smallest and largest measurements (shell width in the first, and shell height in the second two categories). The smallest species was *A.psammion* measuring 0.6 mm (shell diameter) and the largest, *A.majuscula* sp. nov. measuring 1.31 mm (shell height). The average measurement of all 53 species was 0.89 mm. Hence, small species were between 0.6–0.85 mm, medium-sized species between 0.86–1.05 mm, and large species between 1.06–1.31 mm. All measurement data are compiled in Suppl. material 2.

#### Shape of the dorsal side

**Figure 1.**
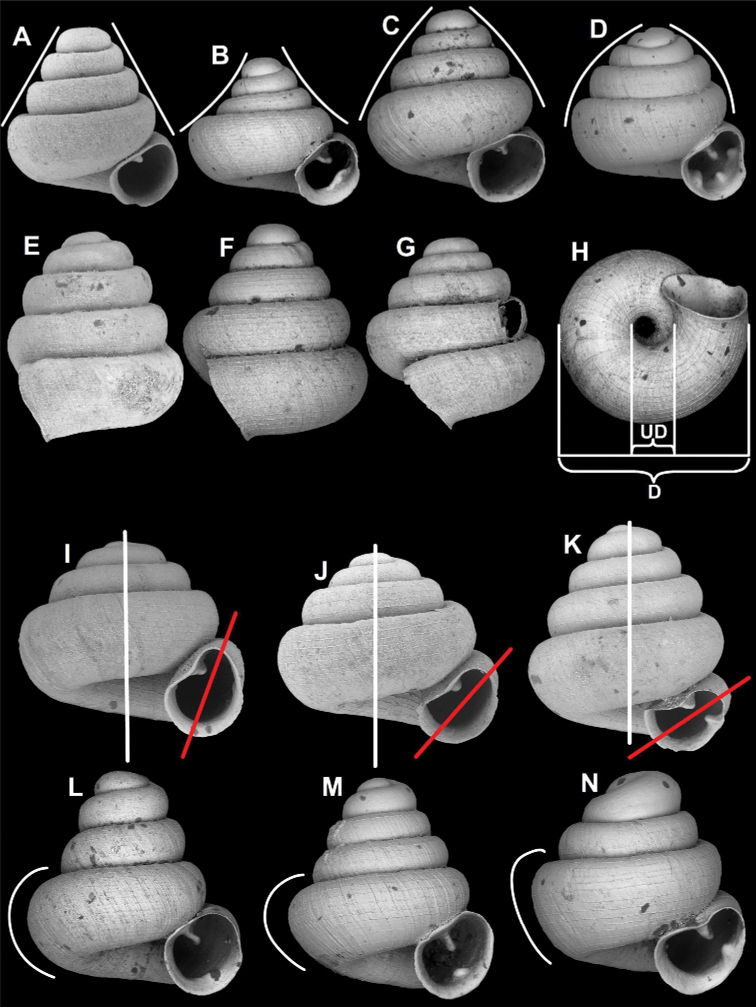
Shape of the dorsal side (**A–D**), position of aperture profile compared to the shell axis in lateral view (**E–G**), method of measuring the umbilicus (**H**) (D: shell diameter, UD: umbilicus diameter), apertural axis (red line) compared to the shell axis (white line) in frontal view (**I–K**) and differences of body whorl shape in apertural view (**L–N**) **A** conical **B** concave-conical **C** conical-globular **D** domed **E** slightly oblique **F** moderately oblique **G** strongly oblique **I** small angle **J** moderate angle **K** large angle **L** rounded **M** depressed-rounded **N** shouldered.

Shape of the upper (dorsal) side (conical, concave-conical, conical-globular, domed, see Fig. 1A–D) is crucial in species recognition.

The protoconch shape and orientation are never different from what we would expect from the general shell shape (i.e., low in case of depressed shells, high in case of high-spired shells).

#### Shell colour

All *Angustopila* shells are colourless (white, off-white, etc., probably depending on the condition of available shells), i.e., never brown, reddish, or yellowish, as in other genera of Hypselostomatidae. Although this trait has little taxonomic value within this genus (but important between genera!), we note whether the shells are conspicuously transparent or not.

#### Aperture orientation

Aperture profile can be slightly oblique, moderately oblique, and strongly oblique to the shell axis in lateral view (Fig. 1E–G). The angle of the aperture axis compared to the shell axis in apertural view can also be species specific. The relationships of the two axes are categorised into three groups, although there is a continuous variation across species. Namely, they can join under a small, a moderate and a large angle. The apertural axis can also be species specific compared to the shell axis in frontal view (Fig. 1I–K).

#### Umbilicus width

Relative umbilicus diameter (RUD: umbilicus diameter/shell diameter × 100) is often species specific, therefore it is indicated under Measurements under each species (Fig. 1H).

#### Shape of body whorl

Most species have a rounded body whorl from apertural view, others can have a depressed-rounded form (i.e., very slightly keeled) or shouldered body whorls (Fig. 1L–N). In the latter case, the body whorl looks as if it was pushed from the basolateral direction and thus, has a flat profile viewed from the bottom left corner.

#### Sculpture

Due to the small size of *Angustopila* species, their fine sculpture is usually not visible under a standard stereo microscope (ca. 40× magnification). However, the fine sculpture is usually visible via scanning electron microscopy (SEM). In the present paper, the description of the fine sculpture is based on 1–3 shells (per population of species) examined via SEM.

**Figure 2.**
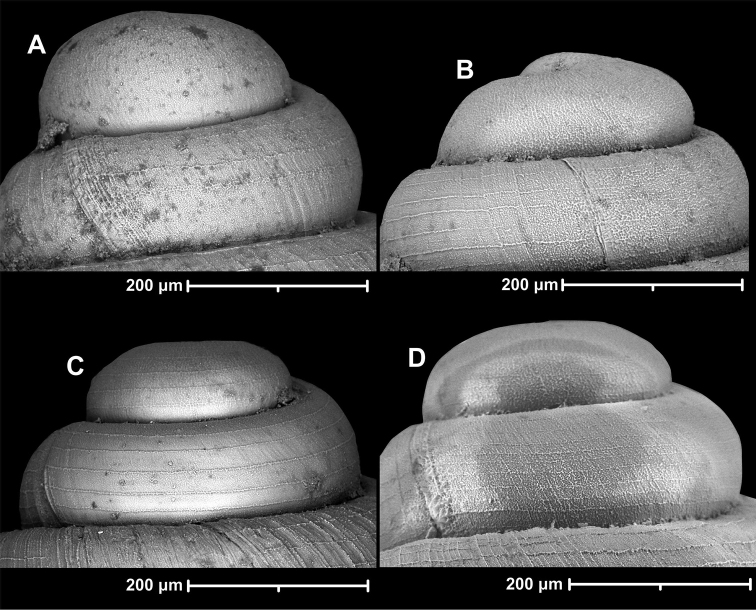
Protoconch sculpture of *Angustopila* spp. **A***A.fabella* (2019/128, type1, see Suppl. material 3: fig. S12) **B***Angustopilaapiostoma* Páll-Gergely & Vermeulen, sp. nov., holotype (see Fig. 9) **C***Angustopilatetradon* Páll-Gergely & Hunyadi, sp. nov., holotype (see Fig. 93) **D***Angustopilaoccidentalis* Páll-Gergely & Hunyadi, sp. nov., holotype (see Fig. 70).

The general fine sculpture of *Angustopila* species shows a dry, dimpled, pasty texture (like floury pizza dough), also reminiscent of cross-sectional view of trabecular bone. This dimpled, paste-like texture can be denser or less dense with the dimpled surface varying in structural constitution from raised to flat (nearly smooth), but the basic structure is always recognisable. This surface structure forms raised spiral striae on both the teleoconch and the protoconch. In practically all cases, the end of the protoconch just preceding the protoconch-teleoconch boundary, is spirally striated (Fig. 2A, B). In some species, the entire protoconch is spirally striated (Fig. 2C, D). The density of spiral striation is highly variable (Fig. 2C, D). Although examining this structure is difficult due to the limited number of observable shells, the presence/absence of spiral striation at the beginning of the protoconch is probably a species-specific trait.

**Figure 3.**
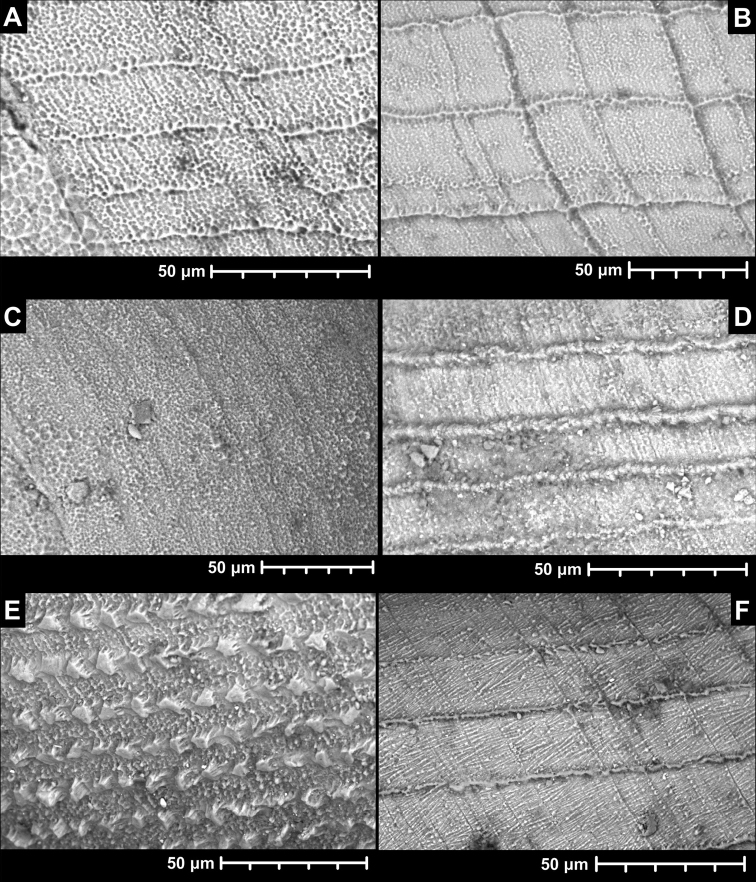
Teleoconch sculpture of *Angustopila* spp. **A** most common form of raised spiral striae (*A.thersites* Páll-Gergely & Vermeulen, sp. nov., WMVT.0333) **B** same as previous, with strong radial lines (*A.antidomedon* Páll-Gergely & Hunyadi, sp. nov., holotype) **C** fine paste-like surface texture without spiral striae (*A.fabella*, 2019/118, “SpeciesX”) **D** finely dimpled, paste-like texture with thick spiral striae (*A.psammion* Páll-Gergely, Vermeulen & Anker, 2022, holotype) **E** unique, stud-like islands aligned in spiral rows (*A.coprologoscoprologos* Páll-Gergely, Jochum & Hunyadi, 2022, holotype) **F** spiral striae with oblique scratches between rows (*A.pusilla* Páll-Gergely & Hunyadi, sp. nov., holotype).

The teleoconch is usually similar to the end of the protoconch. Namely, the general paste-like texture forms usually equidistant spiral striae with some growth lines of variable strength (Fig. 3A, B). In very rare cases, the shells have very weak, or practically no recognisable spiral striae (Fig. 3C). In *Angustopilapsammion*, the spiral striae are dense, and their connection to the basal, paste-like texture is not evident as in other species (Fig. 3D). *Angustopilacoprologos* has a rough sculpture with raised, flat-topped (or occasionally pointy) islets arranged like studs fastened onto the shell in spiral rows (Fig. 3E). In two species (*A.pusilla* sp. nov. and *A.fraterminor* sp. nov.), oblique scratches are present between the rows of spiral striae (Fig. 3F).

The density of spiral striation is variable within *Angustopila*, but generally, less than that of similar tiny shells of other SE Asian hypselstomatids possessing colourless shells (*Clostophis*, *Tonkinospira*).

#### Aperture shape

**Figure 4.**
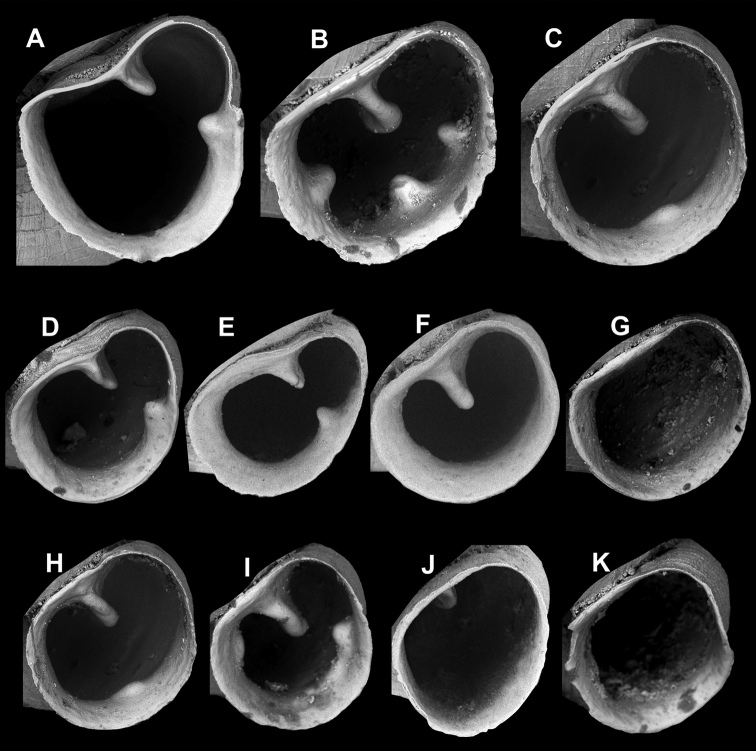
Shape of the parietal callus: **A** concave **B** straight **C** rounded (convex); Aperture shape **D** broad piriform (pear-shaped) **E** narrow piriform (pear-shaped) **F** reniform (kidney-shaped) **G** ovate-oblong **H** subcircular **I** ovoid **J** ovate **K** ovate-subquadrate.

When describing the aperture shape (Fig. 4), we refer to the outer outline of the aperture, i.e., the line of the peristome edge. The shape of the parietal callus (concave, straight, rounded) is crucial. In the case of a concave parietal callus, the aperture shape can be kidney-shaped (reniform) when the aperture is more or less symmetrical, and pear-shaped (piriform), when the sinulus is smaller/more elongate/slenderer than the lower part of the aperture. We can distinguish narrow and broad piriform shapes. The apertures with straight and convex parietal callus can be classified in the following categories:

- ovate-oblong (parietal callus straight, aperture otherwise nearly rounded);
- subcircular (almost rounded);
- ovoid (the sinulus is pointed, the other end of the aperture is rounded);
- ovate (the two ends of the aperture (sinulus and the basal/columellar end) are nearly equally rounded);
- ovate-subquadrate (reminiscent of a rhombus or square).

Naturally, these are artificial categories, and often just as in other taxonomic works describing shapes, these are subject to personal impressions and subjective categories. However, here we present these eight categories in order to provide a standard for the nomenclature we use.

#### Apertural barriers

**Figure 5.**
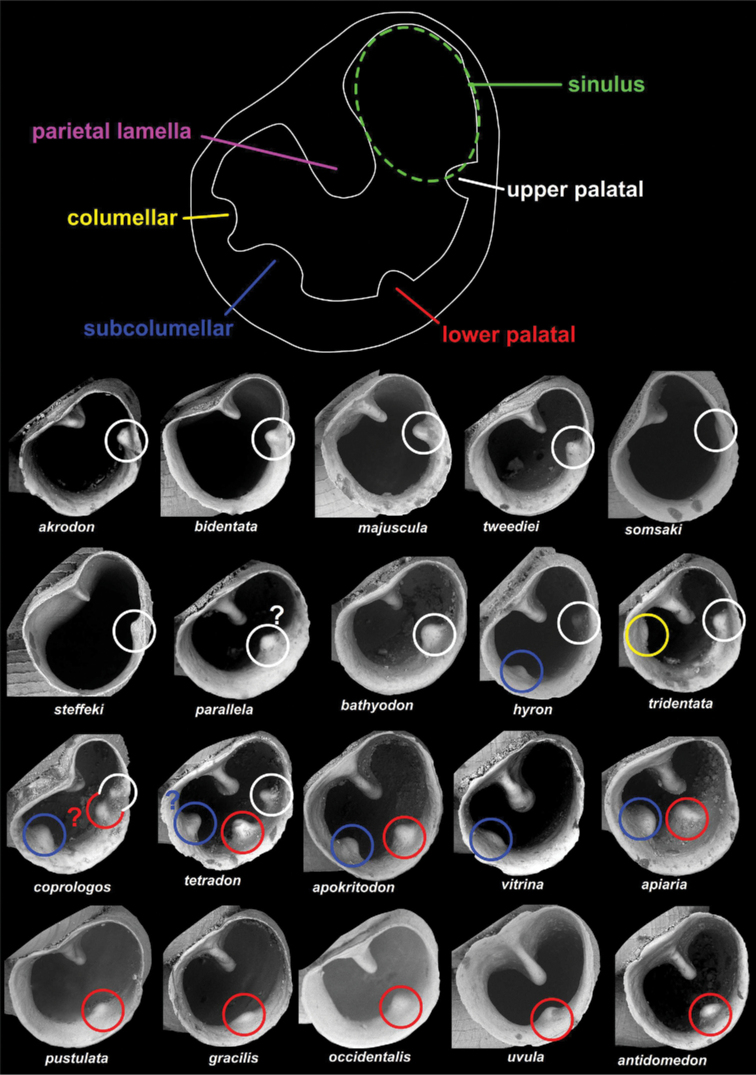
Homologies and nomenclature of apertural barriers. The colours on the upper schematic figure correspond with those of the circles on the images. The parietal lamella is not indicated on the images because it is always apparent.

We could identify five apertural barrier types in *Angustopila* (see Fig. 5).

1. The position of the parietal or angulo-parietal tooth (not clear whether it is homologous with the parietal lamella or the concrescent angular and parietal lamellae of related genera) appears consistent across the entire genus. Therefore, it is probably homologous amongst all *Angustopila* species. It can be very small, low, pointed (e.g., *A.szekeresi*), strongly elevated and long (*A.vomer* sp. nov. and *A.prolixa* sp. nov.), or entirely absent. Although it can have a club-shaped cross-sectional view (*A.uvula* sp. nov. and *A.margaritarion* sp. nov.), it is normally elevated and short in most species. Its depth can also be variable (e.g., deeply situated in *A.szekeresi*). If it is short and knob-like, its position can be species specific (i.e., *A.psammion*, *A.maasseni* sp. nov.).
2. A denticle in upper position, facing the parietal tooth may be present on the palatal wall. The position of this tooth can be very high (*A.akrodon* sp. nov., *A.bidentata* sp. nov., *A.majuscula* sp. nov., etc.) or somewhat lower (*A.parallela* sp. nov., *A.bathyodon* sp. nov.).

In *A.coprologos*, there are two teeth on the upper side of the palatal wall. In *A.coprologosuninodus* ssp. nov., there is only a single palatal tooth, which is similarly aligned as the upper tooth of the nominotypical subspecies. Thus, these are homologous structures. Hence, the lower positioned tooth in *A.coprologos* is considered homologous with the lower palatal denticle.

1. A lower palatal denticle is present in several *Angustopila* species, either together (*A.quadridens* sp. nov.) or without (*A.pustulata* sp. nov., *A.gracilis* sp. nov., *A.uvula* sp. nov., etc.) an upper palatal denticle. In some cases (e.g., *A.parallela* sp. nov.), it is difficult to determine whether the single tooth on the palatal lip represents the upper or lower palatal tooth.
2. Rarely, there is a denticle at the junction of the basal and columellar regions (*A.vitrina* sp. nov., *A.hyron* sp. nov., *A.apiaria* sp. nov.). We consider it homologous with the subcolumellar tooth termed by Pilsbry (1948). Alternatively, we remark that the lamella in the same position was termed columellaris by Hausdorf (1996).
3. The tooth on the columellar side of *A.tridentata* sp. nov. is treated differently than that comprising the subcolumellar tooth. However, it is not certain that the two are not homologous because, for example, *A.quadridens* sp. nov. bears a tooth that is seemingly intermediate between the two positions.

### ﻿Taxonomic treatment

In the current revision, only a single species (*Angustopilaerawanica* sp. nov.) could be anatomically examined. These samples provided some information on the morphology of the reproductive organs of the genus *Angustopila*, but anatomical traits could not be compared with those of congeners. Therefore, only conchological characters could be examined for species recognition and delimitation. Our approach was to delimit distinct morphotypes based on characters which are either discrete in nature or which are continuous but with distinct gaps. In other words, we mostly used the ‘morphospecies’ concept.

When two populations were overall similar but showed stable, clearly recognisable conchological differences (especially regarding apertural denticles), we treated them as separate species (i.e., *A.gracilis* sp. nov. vs. *A.pustulata* sp. nov.; *A.reticulata* sp. nov. vs. *A.tweediei* sp. nov.). In the case of *A.coprologos*, shells from the population with a single parietal denticle are separated from those possessing two teeth at the subspecies level because of the similarity of other shell characters and the very small geographic distance.

When distinct populations showed some minor conchological differences (mostly minor differences in shell, aperture shape, sculpture), we refrained from describing them as separate species. Instead, we identified the non-type population as ‘cf.’ of the species in question, and did not select them to be paratypes (see under *A.cavicola* sp. nov., *A.fratermajor* sp. nov.). Thus, throughout the manuscript, we applied rather a “lumping” approach.

Our revision resulted in the recognition of three widely distributed species (*A.elevata*, *A.fabella*, *A.szekeresi*). Interestingly, conchological variability was low in *A.elevata* and *A.szekeresi* despite the large geographic area. However, this was not the case for *A.fabella*. The populations treated as *A.fabella* show considerable differences in shell shape (conical, conical-globular, and concave-conical), microstructure and size. In several instances, more than one ‘type’ of *A.fabella* is found in sympatry or parapatry. In these cases, it was clear that we are referring to two distinct species, and thus, we described one of them as new (see *A.babel* sp. nov., *A.margaritarion* sp. nov., *A.vandevenderi* sp. nov.). Therefore, we had to partly rely on the biological species concept for species having a conical or concave-conical shell shape and a single parietal tooth.

In some cases, extraordinary diversity was found in a single sample. In one instance (sample 2019/118 of *A.fabella*), the two sympatric morphs form non-overlapping clusters on the shell diameter vs. shell height diagram. In the other two notable cases (sample 2019/128 of *A.fabella* and the type sample of *A.huoyani*), although most shells could be assigned to morphotypes, there was some uncertainty such that the clusters overlapped. In other cases (i.e., *Plagigeyeriazetaprotogona*, see Grego et al. 2017), our samples may represent thanatocoenoses only, i.e., they were washed together from various, geographically isolated subpopulations and therefore, the co-existence of the various morphs is just artefactual.

### ﻿Species diversity

**Figure 6.**
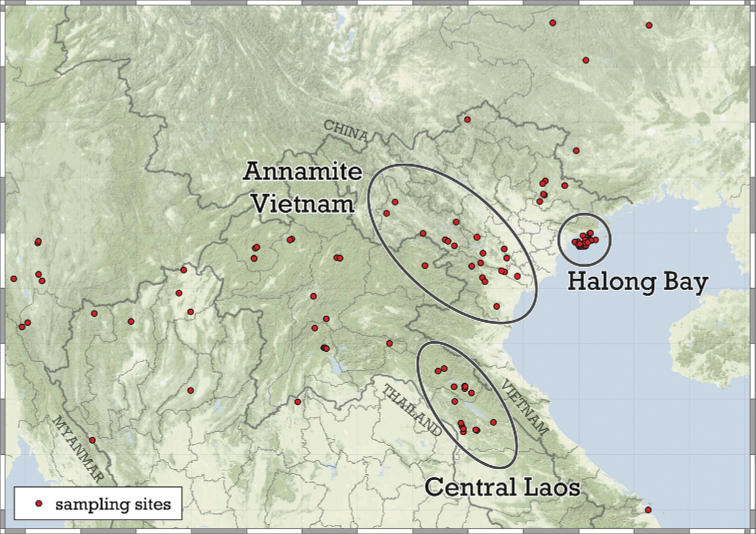
Sampled areas used for the estimation of the number of unobserved species.

The number of unobserved species was estimated for four datasets (areas) (see raw data in Suppl. material 1). Three relatively densely sampled areas (Annamite Mountains, Central Laos, Halong Bay, see Fig. 6) were considered in conjunction with the entire dataset. For the latter, we did not include *A.milium* from NE India because its type locality is situated very far from the nearest *Angustopila* records. We also lack SEM images of *A.milium*, making the identity of that species somewhat questionable. We additionally included one species not described in this study (*Angustopila* sp. 1.). For the Halong Bay, samples collected from the inner and outer sections of the same cave are treated as one.

The number of unobserved species was estimated by two non-parametric methods from incidence data: bias corrected version of the Chao estimator (Chao2-bc; Chao 2005) and the improved Chao estimator (iChao2; Chiu et al. 2014). The former uses only the number of rarest species (singletons and doubletons) and often underestimates the number of unobserved species. Thus, its value could be interpreted as a lower bound rather than the real value of unobserved diversity. iChao2 improves the estimate based on the Good-Turing frequency formula using the frequency of tripletons and quadrupletons. The cost of the improvement is that it needs more data for a reliable estimation; if sample size is small relative to the species richness, its confidence interval may be extremely wide. All calculations were done by SpadeR package (Chao et al. 2016) in R environment (R Core Team 2021).

### ﻿Abbreviations

**CUMZ**Chulalongkorn University Museum of Zoology (Bangkok, Thailand)

**D** Shell diameter

**H** Shell height

**HA** Collection András Hunyadi (Budapest, Hungary)

**HNHM**Hungarian Natural History Museum (Budapest, Hungary)

**j/b** juvenile/broken shells

**JG** Collection Jozef Grego (Banská Bystrica, Slovakia)

**JJV** Private collection of Jaap Vermeulen (Leiden, The Netherlands)

**MCSMNH** Malacological collection of the Slovenian Museum of Natural History (Ljubljana, Slovenia)

**MNHN**Muséum National d’Histoire Naturelle (Paris, France)

**NHM** The Natural History Museum (London, UK)

**NHMUK** When citing NHM registered specimens

**NHMW**Naturhistorisches Museum Wien (Vienna, Austria)

**NMBE**Naturhistorisches Museum der Burgergemeinde Bern (Bern, Switzerland)

**PD** Collection Pongrat Dumrongrojwattana (Bangsaen, Thailand)

**PGB** Collection Barna Páll-Gergely (Mosonmagyaróvár, Hungary)

**RE** Collection Reischütz (Horn, Austria)

**RMNH** National Museum of Natural History Naturalis (Leiden, The Netherlands)

**RUD** Relative umbilical diameter

**SMF**Senckenberg Forschungsinstitut und Naturmuseum (Frankfurt am Main, Germany)

**UF**Florida Museum of Natural History (Gainesville, USA)

**UMMZ**Museum of Zoology, University of Michigan (Ann Arbor, Michigan, USA)

**UMZC** University Museum of Zoology (Cambridge, United Kingdom)

**VNMN**Vietnam National Museum of Nature (Hanoi, Vietnam)

## ﻿Systematic descriptions

### ﻿Family Hypselostomatidae Zilch, 1959

#### ﻿Genus Angustopila

Taxon classificationAnimaliaStylommatophoraGastrocoptidae

Jochum, Slapnik & Páll-Gergely, 2014

E9C03A60-6DAA-54F9-8B7A-ADDCD55D43D3

Angustopila Jochum, Slapnik & Páll-Gergely, 2014 in Jochum et al. 2014: 26.

##### Type species.

*Systenostomatamlod* Panha & Burch, 2002, by original designation.

##### Content.

**Figure 7.**
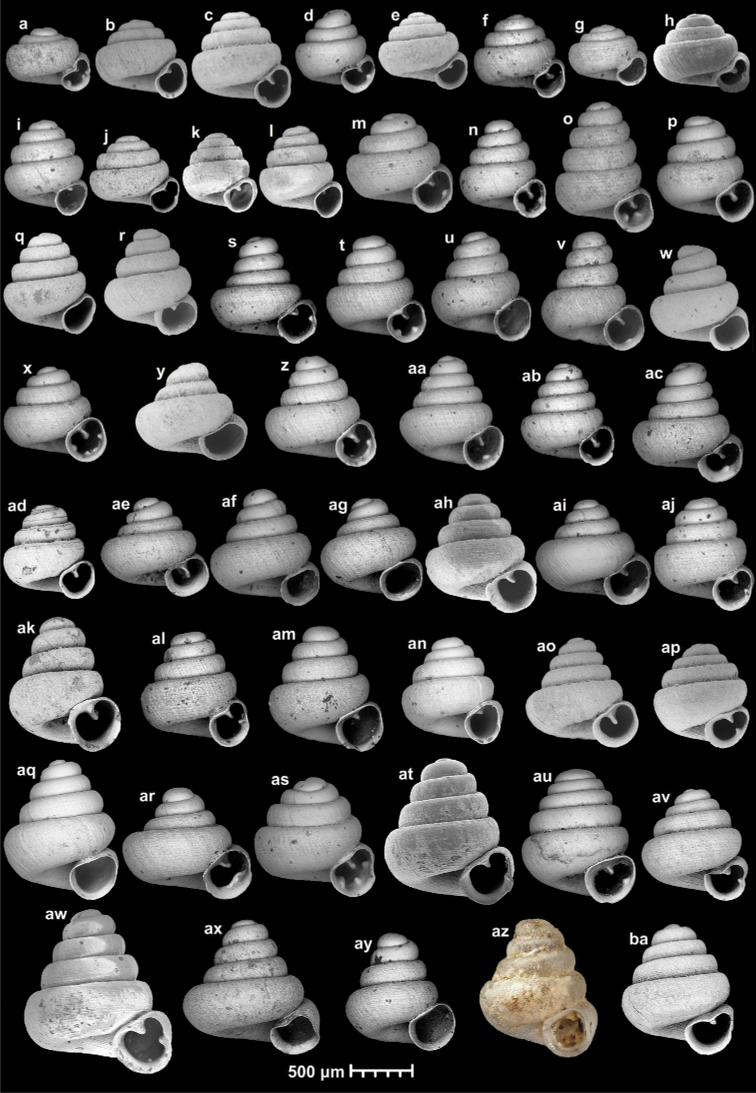
**a***A.coprologoscoprologos* Páll-Gergely, Jochum & Hunyadi, 2022 **b***A.somsaki* Páll-Gergely & Hunyadi, sp. nov. **c***A.hyron* Páll-Gergely & Vermeulen, sp. nov. **d***A.papaver* Páll-Gergely & Hunyadi, sp. nov. **e***A.maasseni* Páll-Gergely & Vermeulen, sp. nov. **f***A.prolixa* Páll-Gergely & Hunyadi, sp. nov. **g***A.psammion* Páll-Gergely, Vermeulen & Anker, 2022 **h***A.pallgergelyi* Dumrongrojwattana, Chuenit & Wongkamhaeng, 2021 **i***A.pusilla* Páll-Gergely & Hunyadi, sp. nov. **j***A.steffeki* Páll-Gergely & Grego, sp. nov. **k***A.cicatricosa* Páll-Gergely & Vermeulen, sp. nov. **l***A.fraterminor* Páll-Gergely & Vermeulen, sp. nov. **m***A.bathyodon* Páll-Gergely & Hunyadi, sp. nov. **n***A.rara* Páll-Gergely & Hunyadi, sp. nov. **o***A.apiaria* Páll-Gergely & Hunyadi, sp. nov. **p***A.margaritarion* Páll-Gergely & Hunyadi, sp. nov. **q***A.apiostoma* Páll-Gergely & Vermeulen, sp. nov. **r***A.szekeresi* Páll-Gergely & Hunyadi, 2015 **s***A.akrodon* Páll-Gergely & Hunyadi, sp. nov. **t***A.apokritodon* Páll-Gergely & Hunyadi, sp. nov. **u***A.oostoma* Páll-Gergely & Vermeulen, sp. nov. **v***A.gracilis* Páll-Gergely & Hunyadi, sp. nov. **w***A.elevata* (Thompson & Upatham, 1997) **x***A.tetradon* Páll-Gergely & Hunyadi, sp. nov. **y***A.megastoma* Páll-Gergely & Vermeulen, sp. nov. **z***A.tridentata* Páll-Gergely & Hunyadi, sp. nov. **aa***A.antidomedon* Páll-Gergely & Hunyadi, sp. nov. **ab***A.vitrina* Páll-Gergely & Hunyadi, sp. nov. **ac***A.uvula* Páll-Gergely & Hunyadi, sp. nov. **ad***A.vandevenderi* Páll-Gergely, sp. nov. **ae***A.vomer* Páll-Gergely & Hunyadi, sp. nov. **af***A.werneri* Páll-Gergely & Hunyadi, sp. nov. **ag***A.thersites* Páll-Gergely & Vermeulen, sp. nov. **ah***A.occidentalis* Páll-Gergely & Hunyadi, sp. nov. **ai***A.pustulata* Páll-Gergely & Hunyadi, sp. nov. **aj***A.tweediei* Páll-Gergely & Hunyadi, sp. nov. **ak***A.cavicola* Páll-Gergely & Dumrongrojwattana, sp. nov. **al***A.reticulata* Páll-Gergely & Hunyadi, sp. nov. **am***A.babel* Páll-Gergely & Vermeulen, sp. nov. **an***A.fratermajor* Páll-Gergely & Vermeulen, sp. nov. **ao***A.fabella* Páll-Gergely & Hunyadi, 2015 **ap***A.dominikae* Páll-Gergely & Hunyadi, 2015 **aq***A.tonkinospiroides* Páll-Gergely & Vermeulen, sp. nov. **ar***A.parallela* Páll-Gergely & Hunyadi, sp. nov. **as***A.quadridens* Páll-Gergely & Vermeulen, sp. nov. **at***A.tamlod* (Panha & Burch, 1999) **au***A.huoyani* Jochum, Slapnik & Páll-Gergely, 2014 **av***A.bidentata* Páll-Gergely & Jochum, sp. nov. **aw***A.majuscula* Páll-Gergely & Hunyadi, sp. nov. **ax***A.concava* (Thompson & Upatham, 1997) **ay***A.halongensis* Páll-Gergely & Vermeulen, sp. nov. **az***A.milium* (Benson, 1853) **ba***A.erawanica* Páll-Gergely & Dumrongrojwattana, sp. nov. (Note that only species-level taxa indicated).

53 species and one subspecies: *A.akrodon* Páll-Gergely & Hunyadi, sp. nov., *A.apiaria* Páll-Gergely & Hunyadi, sp. nov., *A.apiostoma* Páll-Gergely & Vermeulen, sp. nov., *A.apokritodon* Páll-Gergely & Hunyadi, sp. nov., *A.antidomedon* Páll-Gergely & Hunyadi, sp. nov., *A.babel* Páll-Gergely & Vermeulen, sp. nov., *A.bathyodon* Páll-Gergely & Hunyadi, sp. nov., *A.bidentata* Páll-Gergely & Jochum, sp. nov., *A.cavicola* Páll-Gergely & Dumrongrojwattana, sp. nov., *A.cicatricosa* Páll-Gergely & Vermeulen, sp. nov., *A.concava* (Thompson & Upatham, 1997), *A.coprologoscoprologos* Páll-Gergely, Jochum & Hunyadi, 2022, *A.coprologosuninodus* Páll-Gergely & Grego, ssp. nov., *A.dominikae* Páll-Gergely & Hunyadi, 2015, *A.elevata* (Thompson & Upatham, 1997), *A.erawanica* Páll-Gergely & Dumrongrojwattana, sp. nov., *A.fabella* Páll-Gergely & Hunyadi, 2015, *A.fratermajor* Páll-Gergely & Vermeulen, sp. nov., *A.fraterminor* Páll-Gergely & Vermeulen, sp. nov., *A.gracilis* Páll-Gergely & Hunyadi, sp. nov., *A.halongensis* Páll-Gergely & Vermeulen, sp. nov., *A.huoyani* Jochum, Slapnik & Páll-Gergely, 2014, *A.hyron* Páll-Gergely & Vermeulen, sp. nov., *A.maasseni* Páll-Gergely & Vermeulen, sp. nov., *A.majuscula* Páll-Gergely & Hunyadi, sp. nov., *A.margaritarion* Páll-Gergely & Hunyadi, sp. nov., *A.megastoma* Páll-Gergely & Vermeulen, sp. nov., *A.milium* (Benson, 1853), *A.occidentalis* Páll-Gergely & Hunyadi, sp. nov., *A.oostoma* Páll-Gergely & Vermeulen, sp. nov., *A.pallgergelyi* Dumrongrojwattana, Chuenit & Wongkamhaeng, 2021, *A.papaver* Páll-Gergely & Hunyadi, sp. nov., *A.parallela* Páll-Gergely & Hunyadi, sp. nov., *A.prolixa* Páll-Gergely & Hunyadi, sp. nov., *A.psammion* Páll-Gergely, Vermeulen & Anker, 2022, *A.pusilla* Páll-Gergely & Hunyadi, sp. nov., *A.pustulata* Páll-Gergely & Hunyadi, sp. nov., *A.quadridens* Páll-Gergely & Vermeulen, sp. nov., *A.rara* Páll-Gergely & Hunyadi, sp. nov., *A.reticulata* Páll-Gergely & Hunyadi, sp. nov., *A.somsaki* Páll-Gergely & Hunyadi, sp. nov., *A.steffeki* Páll-Gergely & Grego, sp. nov., *A.szekeresi* Páll-Gergely & Hunyadi, 2015, *A.thersites* Páll-Gergely & Vermeulen, sp. nov., *A.tonkinospiroides* Páll-Gergely & Vermeulen, sp. nov., *A.tamlod* (Panha & Burch, 2002), *A.tetradon* Páll-Gergely & Hunyadi, sp. nov., *A.tridentata* Páll-Gergely & Hunyadi, sp. nov., *A.tweediei* Páll-Gergely & Hunyadi, sp. nov., *A.uvula* Páll-Gergely & Hunyadi, sp. nov., *A.vandevenderi* Páll-Gergely & Jochum, sp. nov., *A.vitrina* Páll-Gergely & Hunyadi, sp. nov., *A.vomer* Páll-Gergely & Hunyadi, sp. nov., *A.werneri* Páll-Gergely & Hunyadi, sp. nov. (Fig. 7, Table 1).

**Table 1.** Key characters of *Angustopila* species.

Species	Key character	Similar (sub)species
*akrodon* sp. nov.	concave-conical shell, wide umbilicus, 1 parietal + 1 highly situated upper parietal tooth	*majuscula*
*antidomedon* sp. nov.	medium-sized, conical shell with a narrow umbilicus, 1 parietal + 1 lower palatal tooth	*bathyodon*, *gracilis*, *occidentalis*, *pustulata*, *uvula*
*apiaria* sp. nov.	high conical shell, strong spiral striation, 1 parietal + 1 palatal + 1 inward running subcolumellar teeth	*tridentata*
*apiostoma* sp. nov.	aperture shape, protruding aperture, narrow umbilicus	*fratermajor* (lives in sympatry), *elevata*
*apokritodon* sp. nov.	medium-sized, conical shell, 1 long parietal tooth + 1 lower palatal tooth + 1 subcolumellar tooth	*rara*, *tetradon*
*babel* sp. nov.	rather large, narrow umbilicus, 1 strong parietal tooth	*fabella* (differs from neary occurring fabella populations), *fratermajor*
*bathyodon* sp. nov.	small, conical-globular shell, 1 parietal + 1 upper (?) palatal tooth situated in some distance from the peristome	*antidomedon*, *occidentalis*, *pustulata*, *tweediei*
*bidentata* sp. nov.	small to large shell with a very narrow umbilicus, aperture oblique, with a very wide sinulus, 1 strong parietal + 1 upper palatal tooth	*dominikae*, *erawanica*, *huoyani*, *tamlod*
*cavicola* sp. nov.	rather large, conical shell few whorls, a narrow umbilicus, conspicuously large aperture, strongly expanded peristome, 1 parietal tooth	*fabella* (differs from syntopic *fabella* population)
*cicatricosa* sp. nov.	small, ovoid shell, strong spiral striae, 1 rather strong, pointed parietal tooth,	*fraterminor*, *maasseni*, *psammion*
*concava*	large, concave-conical shell, very strongly oblique aperture, narrow sinulus, 1 low parietal tooth	*erawanica*, *occidentalis*, *uvula*
*coprologos coprologos*	small, depressed-globular shell, strong striae consisting of coarse elevations in a chain-like pattern, 1 parietal + 2 palatal + 1 subcolumellar teeth	*coprologos uninodus*
*coprologosuninodus* ssp. nov.	small, depressed-globular shell, strong striae consisting of coarse elevations in a chain-like pattern, 1 parietal + 1 palatal + 1 subcolumellar teeth	*coprologos coprologos*
*dominikae*	medium-sized, corpulent (globular)shell, elongated aperture, 1 parietal + 1 upper palatal tooth	*bidentata*, *erawanica*, *huoyani*, *tamlod*
*elevata*	slightly concave-conical shell, toothless, rather subquadrate aperture	*apiostoma*, *halongensis*, *oostoma*, *werneri* (intraspecific variability must be understood to identify all other toothless species)
*erawanica* sp. nov.	rather large shell, narrow umbilicus, aperture with wide sinulus and impressed at the position of the parietal tooth, 1 strong parietal tooth	*bidentata*, *concava*
*fabella*	variable species (probably a species complex); conical or concave-conical shell, parietal tooth	Intraspecific variability must be understood to identify all other species with 1 tooth
*fratermajor* sp. nov.	variable in size, conical-globular shell, a relatively wide umbilicus, 1 weak, deeply set parietal tooth	*apiostoma*, *babel*, *fabella*, *fraterminor*, *maasseni*, *pusilla*, *tonkinospiroides*
*fraterminor* sp. nov.	rather small ovoid shell, 1 strong but deeply set parietal tooth	*fratermajor* (also live in sympatry)
*gracilis* sp. nov.	medium-sized, concave-conical shell, narrow initial whorls, 1 parietal + 1 blister-like lower palatal tooth	*antidomedon*, *pustulata*
*halongensis* sp. nov.	relatively large, toothless aperture	*elevata*, *werneri*
*huoyani*	rather large, conical-globular shell, very narrow umbilicus, 1 parietal + 1 upper palatal tooth	*dominikae*, *bidentata*, *erawanica*, *tamlod*
*hyron* sp. nov.	small, conical-globular shell, 1 strong parietal + 1 deeply set upper palatal + 1 subcolumellar tooth	*quadridens*
*maasseni* sp. nov.	small, depressed-globular shell, reniform aperture, apertural axis oblique to shell axis, 1 parietal tooth	*cicatricosa*, *psammion*, *somsaki*
*majuscula* sp. nov.	large, conical shell, wide umbilicus, 1 strong palatal + 1 highly placed parietal tooth	*akrodon*, *parallela*, *tamlod*, ‘*Hypselostoma*’ sp.
*margaritarion* sp. nov.	medium-sized, conical-globular shell, weak spiral striation, thickened peristome, elevated, 1 club-shaped parietal tooth	*fabella*, *vandevenderi*, *vitrina*
*megastoma* sp. nov.	large, asymmetrically coiled, concave-conical, depressed shell, weak sculpture, large, strongly oblique aperture, toothless, or with 1 deeply set parietal tooth	*thersites*, *Tonkinospira* spp.
*milium*	uncertain	all toothless species?
*occidentalis* sp. nov.	medium-sized, relatively low conical shell, 1 strong parietal + 1 weak palatal tooth (can be absent)	*antidomedon*, *bathyodon*, *concava*, *uvula*
*oostoma* sp. nov.	medium-sized, conical shell, ovate aperture and a deeply-set, 1 weak parietal tooth	*elevata*, *fabella*
*pallgergelyi*	small, depressed-globular shell with domed dorsal side, a kidney-shaped aperture with a slender sinulus, 1 parietal + 1 upper parietal tooth	*somsaki*, *steffeki*
*papaver* sp. nov.	small, conical to conical-globular shell, a shouldered body whorl, 1 strong parietal + 1 upper palatal tooth	*coprologos*, *psammion*
*parallela* sp. nov.	large, low or high concave-conical shell, wide umbilicus, 1 elevated parietal + 1 elongated or pointed lower palatal	*majuscula*, *pustulata*
*prolixa* sp. nov.	small, domed shell, 1 very high and long parietal tooth	*quadridens*, *vomer*
*psammion*	very small, depressed-globular shell, thick spiral striae, 1 parietal tooth not reaching parietal callus	*cicatricosa*, *maasseni*
*pusilla* sp. nov.	small, ovoid shell, oblique scratches between spiral striae, a suboval aperture with sharp edge, 1 deeply set parietal tooth	*fraterminor*
*pustulata* sp. nov.	medium-sized, usually concave-conical shell, weak sculpture, large aperture, 1 short parietal + 1 wart-like lower palatal tooth	*antidomedon*, *bathyodon*, *gracilis*, *parallela*
*quadridens* sp. nov.	rather small, conical-globular shell, 1 strong parietal + 1 blunt subcolumellar + 2 palatal teeth situated close to peristome edge	*tetradon*
*rara* sp. nov.	small, conical shell with a narrow umbilicus, few spiral striae, 1 parietal + 1 pointed palatal + 1 blunt columellar teeth	*apokritodon*, *tridentata*
*reticulata* sp. nov.	medium-sized, conical shell, strong radial sculpture, 1 strong parietal tooth reaching peristome edge + 1 strong upper palatal + sometimes a small columellar tooth	*tweediei*
*somsaki* sp. nov.	small, depressed-globular shell with domed dorsal side, aperture axis nearly parallel to shell axis, 1 strong parietal + 1 very weak upper palatal tooth	*maasseni*, *pallgergelyi*
*steffeki* sp. nov.	small, depressed-globular shell, strongly protruding aperture, 1 relatively weak parietal + 1 rather strong palatal tooth	*pallgergelyi*, *somsaki*
*szekeresi*	medium-sized, conical to slightly conical-globular, an adnate peristome, 1 weak parietal tooth (sometimes absent)	*fabella*
*tamlod*	rather large, conical shell with dense spiral striation, 1 strong parietal + 1 weaker upper palatal tooth	bidentata, dominikae, huoyani, majuscula
*tetradon* sp. nov.	small, conical shell, 1 parietal + 1 strong subcolumellar + 2 deeply situated palatal teeth	quadridens
*thersites* sp. nov.	irregularly growing whorls, strong spiral striation, toothless aperture	*megastoma* (lives in sympatry), *tonkinospiroides*, *Tonkinospira* spp
*tonkinospiroides* sp. nov.	rather large, conical, irregularly growing whorls, toothless aperture	*thersites*, *fratermajor*, *Tonkinospira* spp
*tridentata* sp. nov.	Medium-sized, concave-conical shell with a wide umbilicus, 1 elevated parietal + 1 pointed upper palatal + 1 blunt, elongated columellar teeth	*apiaria*, *rara*
*tweediei* sp. nov.	medium-sized conical to slightly conical-globular shell, 1 strong parietal tooth reaching peristome edge + 1 upper palatal tooth of comparable height	*bathyodon*, *reticulata*
*uvula* sp. nov.	medium-sized, concave-conical shell, wide umbilicus, 1 strong parietal + 1 lower palatal tooth	*antidomedon*, *concava*, *occidentalis*
*vandevenderi* sp. nov.	small, conical-globular shell, few (ca. 3.75) whorls, normally developed spiral striation, 1 parietal tooth	*fabella*, *margaritarion*
*vitrina* sp. nov.	rather small, conical shell, 1 parietal + 1 blister-like subcolumellar tooth	*margaritarion*
*vomer* sp. nov.	rather small, concave-conical shell, 1 very high and long parietal tooth	*fabella*, *prolixa*
*werneri* sp. nov.	rather large, concave-conical shell, comparatively small, wide umbilicus, toothless aperture	*elevata*, *halongensis*

##### Diagnosis.

Shell minute (diameter: 0.6–1.24 mm, height: 0.46–1.31 mm), transparent when fresh or whitish (practically colourless); conical, concave-conical, conical-globular, globular or depressed-globular, whorls mostly regularly growing, body whorl never detached (although the parietal callus may project from the penultimate whorl); protoconch with or without spiral striae; body whorl with 10–20 (exceptionally up to 22) spiral striae counted from standard apertural view in line with the shell axis; occasionally spiral lines are lacking; peristome expanded, not reflected, aperture with 0–5 teeth, parietal wall with or without 1 tooth; parietal tooth usually elongated, reaches or does not reach parietal callus; other denticles short, situated on or close to peristome; umbilicus open, 14–38% of shell width.

##### Differential diagnosis.

So far, the diagnosis was based on 13 species. Now that there are 53 known *Angustopila* species, the morphological variability of the genus considerably increased. Even with so many species included, the genus appears “compact” in terms of morphological characters, and in most cases, it is not difficult to identify shells to the genus level.

Regarding apertural denticles, *Angustopila* is very conservative. All non-parietal apertural barriers are short and situated on or very close to the peristome. The only exception is *Angustopilaapiaria* sp. nov., in which the palatal and subcolumellar teeth are elongated into the aperture. This, and the relatively disjunct distribution in Central Vietnam may indicate that it warrants a genus of its own.

Amongst Southeast Asian Hypselostomatidae, *Dentisphaera* Páll-Gergely & Jochum, 2017, *Clostophis* Benson, 1860 and *Tonkinospira* Jochum, Slapnik & Páll-Gergely, 2014 are similar to *Angustopila* in the tiny, colourless shells. *Dentisphaera* possesses an angular and parietal tooth, which readily distinguishes it from *Angustopila*, which always has a single tooth on the parietal side. Although *Clostophis* (see Páll-Gergely et al. 2020) and *Tonkinospira* (see Páll-Gergely et al. 2019) are possibly synonyms (Páll-Gergely and Hunyadi 2022), discussion however, is beyond the scope of the present paper. Both are characterised by shells larger than 1.2 mm (typically approximately 2 mm). In samples where both *Angustopila* and *Tonkinospira*/*Clostophis* species occur, it is already clear from the size groups, that *Angustopila* are distinct due to their small size. Moreover, the spiral striation of *Tonkinospira*/*Clostophis* is usually denser than that of *Angustopila* (22–36 ribs on the body whorl in apertural view, compared to usually 10–20 in *Angustopila*). However, there are three species from the Halong Bay Area (*A.megastoma* sp. nov., *A.thersites* sp. nov., and *A.tonkinospiroides* sp. nov.) that resemble several *Tonkinospira* species in the irregular growth of the whorls (especially the dominant body whorl), the comparatively large, mostly toothless aperture, and the relatively dense spiral striae. However, due to their small size, we treat these three species as *Angustopila*, although we remark that future examinations should address this issue.

##### Habitat.

Only three species, *A.cavicola* sp. nov., *A.erawanica* sp. nov., and *A.pallgergelyi* (see Dumrongrojwattana et al. 2021) have ever been collected alive, and with no exception in caves, far from the cave entrance, but occasionally also found around the cave entrance. Similarly, empty shells of several other species have only been found in soil samples collected inside caves. However, the majority of shells were found in soil samples collected at the base of large limestone rocks. In is unknown whether those shells have been washed out of caves and deep crevices, or if the living snails live outside of caves. Generally, *Angustopila* species most probably inhabit moist, deep limestone crevices and caves, close to (or on) root systems.

Soil samples containing *Angustopila* shells collected in and outside of caves, are mostly dominated by diplommatinids and hydrocenids, while shells of other small-bodied snails (hypselostomatids, alycaeids, diapherids, ‘subulinids’, *Microcystina* Mörch, 1872, *Kaliella* W. T. Blanford, 1863, *Philalanka* Godwin-Austen, 1898, locally assimineids) are also often included, but generally, in smaller quantities.

##### Conservation, threats.

The majority of *Angustopila* species are single-site or narrow range endemics. This will not change much, even when more detailed sampling is conducted in the region. Thus, destruction of a habitat of an *Angustopila* species can easily lead to the disappearance of a species. The most tangible threats to their preferred habitats are quarrying and modifying caves for tourism and recreational purposes.

##### Distribution.

**Figure 8.**
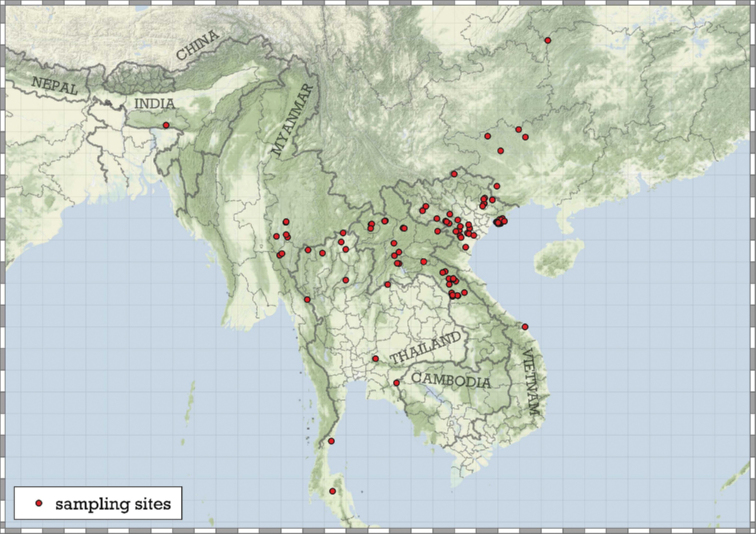
Distribution of the genus *Angustopila*.

Most species are known in Southeast Asia (entire Thailand, southern Shan and Kayah states of Myanmar, northern Laos and northern and central Vietnam). A few species are known from the Chinese province of Guangxi and a single record from Hunan. A single species from the Indian state of Meghalaya indicates that the genus is present in the south-eastern Himalaya (Fig. 8).

### ﻿Species without apertural barriers

**Remarks.** For simplicity, species in this revision are grouped based on the number of teeth. This classification probably does not reflect relatedness.

*Angustopilamegastoma* sp. nov. may lack a parietal tooth. *Angustopilaapiostoma* sp. nov. may rarely possess a parietal tooth.

#### ﻿ Angustopila apiostoma

Taxon classificationAnimaliaStylommatophoraGastrocoptidae

Páll-Gergely & Vermeulen sp. nov.

232000B6-2774-5E58-857D-6A60C113C991

https://zoobank.org/7369BED7-8447-443D-8FC7-627FEB3B1CBB

Figs 9 , 10

##### Type material.

***Holotype***: Vietnam • 1 empty shell (H: 0.84 mm, D. 0.71 mm); Quang Ninh Province, Halong Bay area, Thay Cave on Congfo Island, collected inside cave (locality code: WMVT.0327); 20°52.07'N, 107°12.06'E; 6 Sep. 2003; W.J.M. Maassen leg.; RMNH 5006718.

***Paratypes***: Vietnam • 2 figured shells; same data as for holotype; RMNH 5006722 • 5 shells; same data as for holotype; HNHM 100441 • 5 shells; same data as for holotype; NMBE 550640 • 28 shells; same data as for holotype; coll. PGB (ex coll. W. Maassen) • 987 shells; same data as for holotype; RMNH 347758 • 10 shells; same data as for holotype; coll. HA • 53 shells; Quang Ninh Province, Halong Bay area, unnamed island in Dau Moi Temple area (locality code: WMVT.0328); 20°55.69'N, 107°09.40'E; 13 Sep. 2003; W.J.M. Maassen leg.; RMNH 347759 • 41 shells (one of them is figured: Fig. 99D); Haiphong Province, Cat Ba Island, Cave Qua Vang, inside cave, large, ecologically intact active cave with speleothems; 20°48.64'N, 107°04.64'E; 60 m a.s.l.; 6 Jun. 2017; J.J. Vermeulen & K. Anker leg.; JJV 16614 • 1 shell; Haiphong Province, Cat Ba Island, Cave Qua Vang, around cave entrance, rocky limestone slope with low, rather mature forest; 6 Jun. 2017; J.J. Vermeulen & K. Anker leg.; JJV 16604 • 397 shells; Haiphong Province, Cat Ba Island, large, somewhat ecologically-disturbed, active cave with speleothems; 20°50.06'N, 106°55.91'E; 0–50 m a.s.l.; 7 Jun. 2017; J.J. Vermeulen & K. Anker leg.; JJV 16605 • 343 shells; Haiphong Province, Cat Ba Island, Cave Hoa Cuong, inside cave, polluted cave disturbed by tourism, with concrete paths; 20°50.41'N, 106°59.15'E; 50 m a.s.l.; 5 Jun. 2017; J.J. Vermeulen & K. Anker leg.; JJV 16609 • 13 shells; Haiphong Province, Cat Ba Island, Cave Xa Bac, inside cave; 20°50.07'N, 106°58.61'E; 30 m a.s.l.; 9 Jun. 2017; J.J. Vermeulen & K. Anker leg.; JJV 16615 • 29 shells; Quang Ninh Province, Halong Bay area, Dao Bo Hon, Song Sot Cave, drift material washed over sinkhole in cave; 20°50.83'N, 107°05.67'E; 2 Oct. 1998; J.J. Vermeulen & A.J. Whitten leg.; JJV 6268 • 248 shells; Quang Ninh Province, Halong Bay area, Dao Bo Hon, Song Sot Cave, guano-enriched sediments in cave; 20°50.83'N, 107°05.67'E; 2 Oct. 1998; J.J. Vermeulen & A.J. Whitten leg.; JJV 6269 • 4 shells; Haiphong Province, Cat Ba Island, Cave Xa Bac, around cave entrance; 20°50.07'N, 106°58.61'E; 30 m a.s.l.; 9 Jun. 2017; J.J. Vermeulen & K. Anker leg.; JJV 16616 • 6 shells; Haiphong Province, Cat Ba Island, cave Trung Trang, inside cave, cave disturbed by tourism and concrete paths; 20°47.30'N, 106°59.84'E; 50 m a.s.l.; 5 Jun. 2017; J.J. Vermeulen & K. Anker leg.; JJV 16602.

##### Diagnosis.

A small to medium-sized, toothless *Angustopila* species with a very narrow umbilicus, conspicuously protruding aperture, and a characteristically shaped aperture (kidney-shaped with narrow sinulus).

##### Description.

Shell small to medium-sized for the genus, shell height variable, but always higher than wide; pale grey, conical, last whorl widest from standard apertural view; protoconch consists of 1.5 whorls, microstructure finely pitted and granular with a powdery superficial texture, spiral striation not discernible; teleoconch finely ornamented with irregularly spaced radial growth lines crossed by fine rows of equidistantly-spaced microscopic spiral threads (ca. 18–24 on last whorl in apertural view); on frontal and ventral surface of body whorl spiral and radial lines prominent; whorls 4.25–5, shouldered; aperture slightly oblique to shell axis from lateral view; umbilicus deep, very narrow; ventral side characteristic due to comparatively long and straight tuba; aperture kidney-shaped with narrow sinulus; peristome slightly expanded, not reflected; parietal callus slightly protruding, aperture detached from penultimate whorl; aperture toothless or with a vestigial, deeply-situated parietal tooth.

##### Measurements (in mm).

H = 0.73–0.93, D = 0.67–0.85, H/D*100 = 101.2–128.2 (*n* = 14), RUD = 19.2–25.7 (*n* = 6).

##### Differential diagnosis.

*Angustopilaapiostoma* sp. nov. can be distinguished from *A.elevata*, by the very narrow umbilicus, the protruding aperture (visible in ventral view) and the kidney-shaped aperture. See also under *A.fratermajor* sp. nov. and remarks.

##### Etymology.

The name *apiostoma* derives from the Greek *ἄπιον* (= a pear) and *στόμα* (= mouth) referring to the pear-shaped aperture (to be used as a noun in apposition).

##### Distribution.

This species is known from two nearby localities in the Halong Bay Area, Vietnam (Fig. 11).

**Figure 9.**
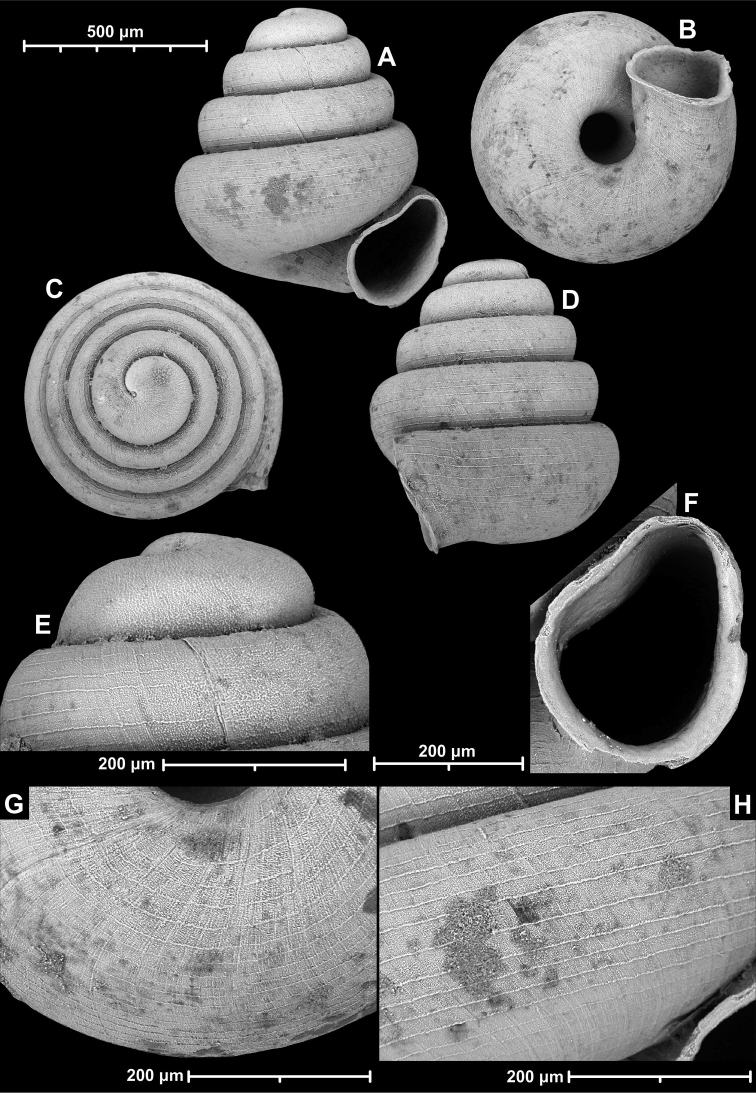
*Angustopilaapiostoma* Páll-Gergely & Vermeulen, sp. nov. (holotype, RMNH 5006718). Apertural (**A**), ventral (**B**), apical (**C**), and lateral (**D**) sides of the shell; microstructure on the protoconch (**E**), continuous edentate aperture (**F**); spiral microstructure on ventral (**G**) and frontal (**H**) surface of the body whorl.

**Figure 10.**
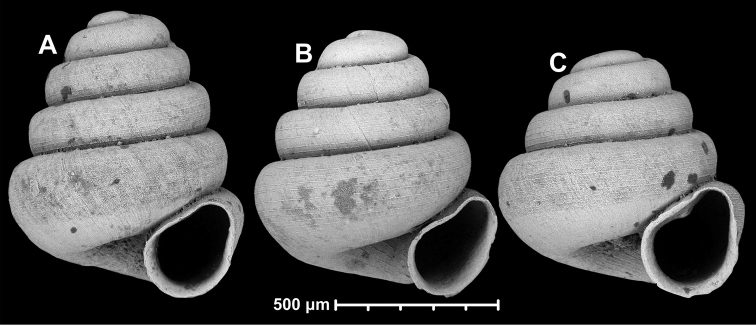
Conchological variability of *Angustopilaapiostoma* Páll-Gergely & Vermeulen, sp. nov. from site WMVT.0327 (**A, C** paratypes [RMNH 5006722] **B** holotype).

**Figure 11.**
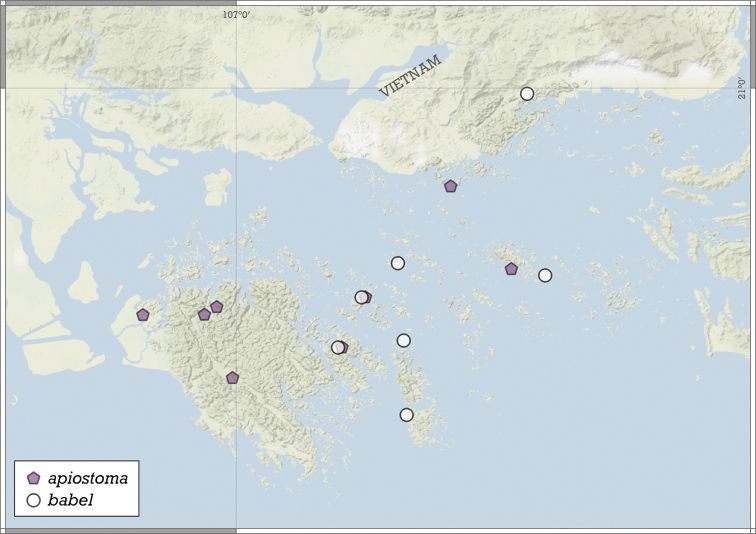
Distribution of *Angustopilaapiostoma* Páll-Gergely & Vermeulen, sp. nov. and *Angustopilababel* Páll-Gergely & Vermeulen, sp. nov.

##### Remarks.

The ventral side and the shape of the aperture of *A.apiostoma* sp. nov. resembles those of *Dentisphaeramaxema* Páll-Gergely & Jochum, 2017, which has a more protruding aperture (i.e., a longer tuba) and six apertural teeth. However, the two prominent teeth on the parietal side of *Dentisphaera* distinguishes this genus from *Angustopila*.

#### ﻿ Angustopila elevata

Taxon classificationAnimaliaStylommatophoraGastrocoptidae

(Thompson & Upatham, 1997)

C4FCA297-B860-56A5-A534-B1F02D40B928

Figs 12 , 13 , Suppl. material 3: figs S1–S6

Systenostoma elevata Thompson & Upatham, 1997: 232–233, figs 39–43.
Angustopila elevata — Jochum et al., 2014: 27.
Angustopila elevata — Páll-Gergely et al., 2015a: 33, fig. 11 (silhouette for size comparison with other species).
Angustopila subelevata Páll-Gergely et al., 2015a: 39, fig. 4. new synonym.
Angustopila subelevata Páll-Gergely et al., 2017: 332, figs 1B, 2A–G, 7E, F.

##### Type locality.

“Thailand, Chiang Mae Province, Doi Chiang Dao (Mountain), 7 km west of Chiang Dao; 600 m altitude (19°24.3'N, 98°54.2'E)” (*A.elevata*); China, Guangxi (广西), Hechi Shi (河池市), Bama Xian (巴马县), cliffs at the southern edge of Jiaole Cun (交乐村), 590 m, 24°7.045'N, 107°7.847'E” (*A.subelevata*).

##### Material examined.

China • 23 shells; Guangxi, Bose Shi, Leye Xian, ca. 2 km from Leye towards Huaping Zhen, cave above Niuping junction (locality code: 2013/85); 24°47.61'N 106°31.89'E; 590 m a.s.l.; 8 Sep. 2013; A. Hunyadi & M. Szekeres leg.; coll. HA • 3 shells; Guangxi, Hechi Shi, Huanjiang Maonanzu Zizhixian, south of Mulun Guojiaji Ziran Baohuqu, rocks above Dongning (locality code: 2013/99); 25°05.97'N 107°57.64'E; 530 m a.s.l.; 17 September 2013; A. Hunyadi & M. Szekeres leg.; coll. HA.

Myanmar • 1 figured shell; Shan State, 7.4 km from the centre of Hoponh towards Namsang along road no. 4, ca. 5 km north, Parpant Cave (locality code: 2018/36); 20°50.96'N, 97°14.26'E; 1170 m a.s.l.; 6 Oct. 2018; A. Hunyadi, K. Okubo & J.U. Otani leg.; HNHM 103484 • 6 shells + 2 j/b shells; same data as for preceding shell; coll. HA • 1 shell; Shan State, Hopong, Hopong Spring and cave (locality code: JG/2019/2); 20°49.05'N, 97°13.49'E; 2 Feb. 2019; J. Grego leg.; coll. JG.

Thailand • 1 figured shell; Tak Province, 6 km NNW Tha Song Yang, left side of road no. 105 (locality code: 2015/23); 17°15.36'N, 98°12.65'E; 140 m a.s.l.; 15 Feb. 2015; A. Hunyadi leg.; HNHM 100183 • 7 shells; same data as for preceding shell; coll. HA • 1 figured shell; Chiang Mai Province, north-eastern part of Doi Chiang Dao, Wat Tham Chiang Dao northwest 2 km; 19°24.02'N, 98°54.68'E; 835 m a.s.l.; 7 Feb. 2015; A. Hunyadi leg.; HNHM 100184; • 11 shells; same data as for preceding shell; coll. HA • 18 shells + 4 j/b shells; Chiang Rai Province, 7 to 9 km SSW of Mae Sai, around Wat Tham Pla (locality code: 2015/18); 20°19.72'N, 99°51.82'E; 400 m a.s.l.; 12 Feb. 2015; A. Hunyadi leg.; coll. HA • 1 figured shell; Nakhon Si Thammarat Province, Wat Tham Thong Panara, vicinity of the temple (locality code: 2015/31); 08°25.28'N, 99°22.76'E; 40 m a.s.l.; 19 Feb. 2015; A. Hunyadi leg.; HNHM 100180; • 7 adult + 2 j/b shells; same data as for preceding shell; coll. HA.

Laos • 56 adult + 6 j/b shells; Luang Namtha Province, 20.6 km southeast from centre of Vieng Phou Kha, 1.5 km south of Ban Phou Lek, right side of road (locality code: 2019/121); 20°32.21'N, 101°08.10'E; 785 m a.s.l.; 8 Oct. 2019; A. Hunyadi leg.; coll. HA • 70 adult + 22 j/b shells; Luang Namtha Province, 19.8 km southeast from centre of Vieng Phou Kha, south from Ban Phou Lek, southern edge of quarry (locality code: 2019/122); 20°32.54'N, 101°07.96'E; 780 m a.s.l.; 8 Oct. 2019; A. Hunyadi leg.; coll. HA • 21 shells; Udomxai Province, 10 km south of centre of Na Mor, 3.8 km east-southeast of Na Xay, rock wall facing north (locality code: 2019/119); 20°53.37'N, 101°48.96'E; 660 m a.s.l.; 7 Oct. 2019; A. Hunyadi leg.; coll. HA • 141 adult shells + 21 j/b shells; Luang Namtha Province, 43.8 km from centre of Luang Namtha towards Vieng Phou Kha, Phou Lan, 100 m from left side of road (locality code: 2019/123); 20°44.53'N, 101°11.10'E; 770 m a.s.l.; 8 Oct. 2019; A. Hunyadi leg.; coll. HA • 12 adult shells; Luang Prabang Province, 3.1 km from centre of Nong Khiaw towards Pak Xeng, Tham Pha Toke, around the cave (locality code: 2019/114); 20°33.22'N, 102°37.72'E; 345 m a.s.l.; 5 Oct. 2019; A. Hunyadi leg.; coll. HA • 3 adult shells; Luang Namtha Province, 48 km southwest from centre of Luang Namtha towards Vieng Phou Kha, Nam Eng, Tham Kao Rao (locality code: 2019/120); 20°43.45'N, 101°09.26'E; 745 m a.s.l.; 7 Oct. 2019; A. Hunyadi leg.; coll. HA • 1 shell; Khammouane Province, Tham Kong Lor (cave), 60 km direct N of Thakhek, at the base of limestone rocks above Recreational Area (locality code: La.2); 17°57.39'N, 104°45.65'E; 200 m a.s.l.; Mar. 2010; A. Reischütz leg.; NHMW-MO-112005 • 2 complete + 1 broken shells; same data as for preceding; coll. RE • 1 shell; Vientiane Province, Vang Vieng, rocks at Vang Vieng Resort, by river Nam Song, limestone rocks at Tham Chang cave (locality code: La.12); 18°54.66'N, 102°26.42'E; 280 m a.s.l.; Mar. 2010; A. Reischütz leg.; coll. RE • 1 shell; Vientiane Province, 4.5 km west from centre of Vang Vieng, Phone Ngeun, Tham Khan Kham (locality code: 2019/133); 18°55.53'N, 102°24.95'E; 280 m a.s.l.; 14 Oct. 2019; A. Hunyadi leg.; coll. HA.

Vietnam • 18 adult shells; Sơn La Province, Thuận Châu District, 900 m southeast from centre of Co Mạ, rock wall above unpaved road (locality code: 2020/18); 21°21.19'N, 103°31.68'E; 1300 m a.s.l.; 8 Feb. 2020; A. Hunyadi leg.; coll. HA • 1 shell; Sơn La Province, Mộc Châu, Hang Dơi, around the entrance of the cave (locality code: 2020/26); 20°50.96'N, 104°38.34'E; 865 m a.s.l.; 11 Feb. 2020; A. Hunyadi leg.; coll. HA • 4 adult shells; Lạng Sơn Province, Bình Gia, eastern edge of the village, Di Chí Kéo Lèng, rock wall (locality code: 2020/48); 21°56.30'N, 106°23.82'E; 375 m a.s.l.; 17 Feb. 2020; A. Hunyadi leg.; coll. HA • 1 shell; Sơn La Province, Quỳnh Nhai district, 20 km north from cross to Thuận Châu, Chiềng Khoang, cave above the village (locality code: 2020/9); 21°33.44'N, 103°40.91'E; 315 m a.s.l.; 7 Feb. 2020; A. Hunyadi leg.; coll. HA.

##### Diagnosis.

A small to medium-sized *Angustopila* species with relatively irregularly coiled, slightly concave-conical shell, and a toothless, rather subquadrate aperture. Individuals in most populations possess widely spaced radial ribs the on the ventral side and on the body whorl in apertural view.

##### Measurements (in mm).

H = 0.8–0.97, D = 0.74–0.88, H/D*100 = 95.4–123 (*n* = 58), RUD = 24.1–31.6 (*n* = 20).

##### Differential diagnosis.

See also under *A.apiostoma* sp. nov., *A.halongensis* sp. nov., *A.oostoma* sp. nov., and *A.werneri* sp. nov.

##### Distribution.

*A.elevata* is the most widely distributed *Angustopila* species, inhabiting Myanmar’s Shan State, northern and southern Thailand, northern Laos, northern Vietnam, and southern China (Guangxi) (see Fig. 14).

**Figure 12.**
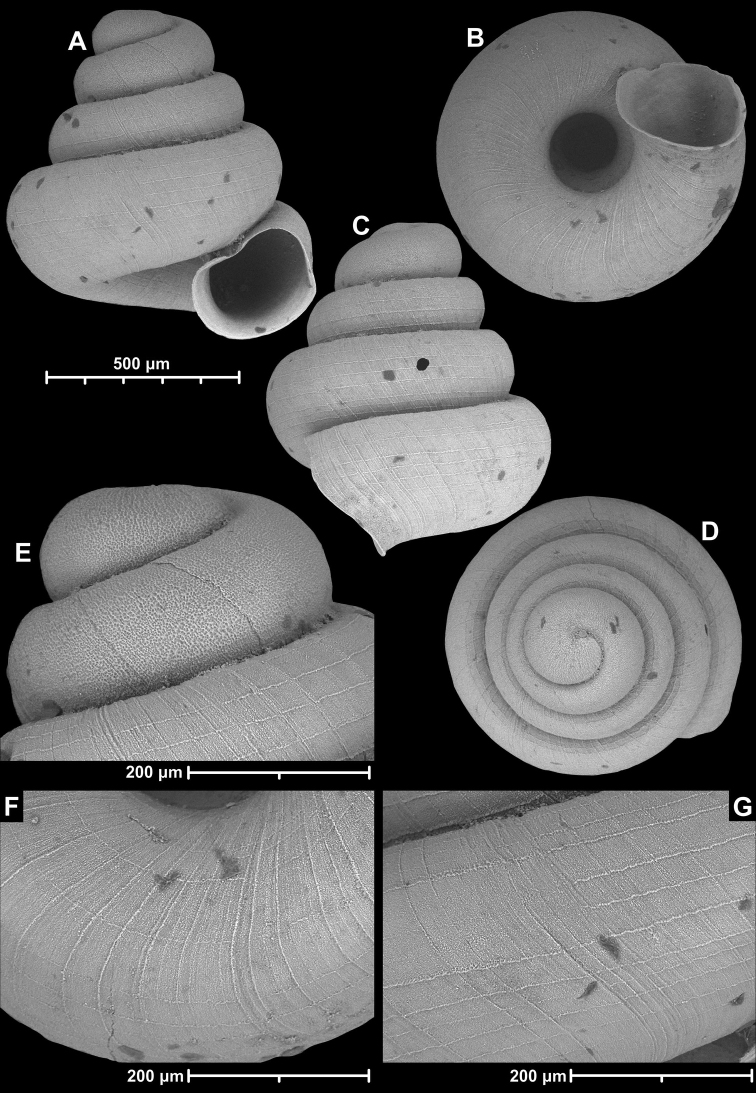
*Angustopilaelevata* (Thompson & Upatham, 1997) (specimen from the type locality, HNHM 100184). Apertural (**A**), ventral (**B**), lateral (**C**) and apical (**D**) sides of the shell; granular sculpture on the protoconch (**E**), ventral (**F**) and frontal (**G**) surface of the body whorl.

**Figure 13.**
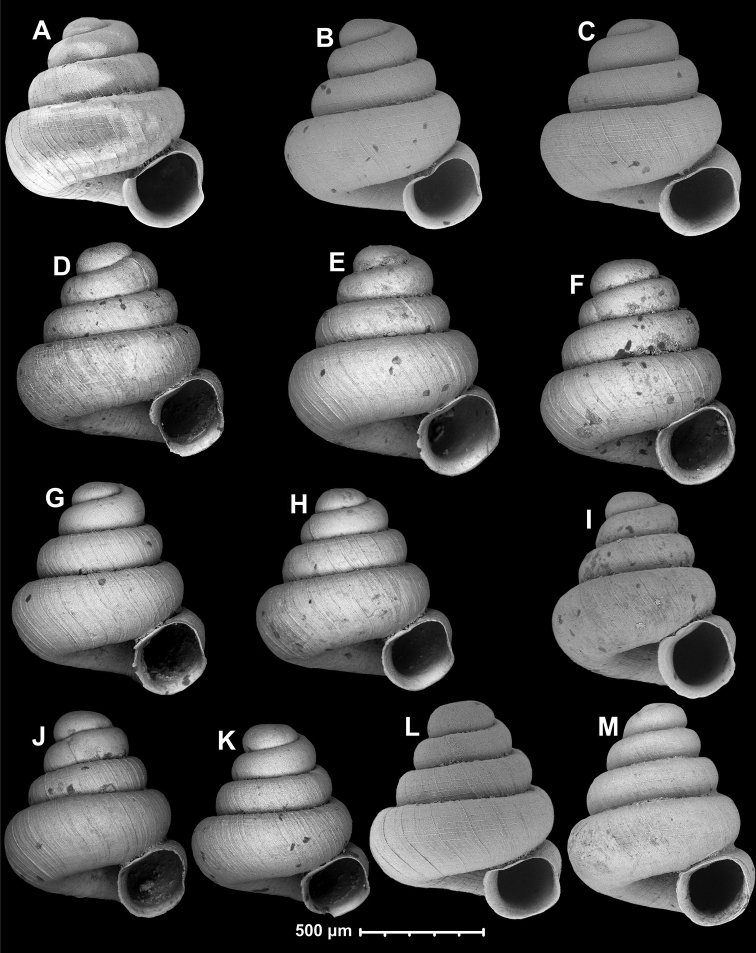
*Angustopilaelevata* (Thompson & Upatham, 1997) **A** Perpant Cave (Myanmar) **B** Chiang Dao **C** Tha Song Yang **D** Panara **E** 2019/123 **F** 2019/119 **G** 2020/18 **H** 2020/26 **I** Cong Troi Cave **J** 2020/52 **K** 2020/48 **L***A.subelevata* holotype **M** La.2.

**Figure 14.**
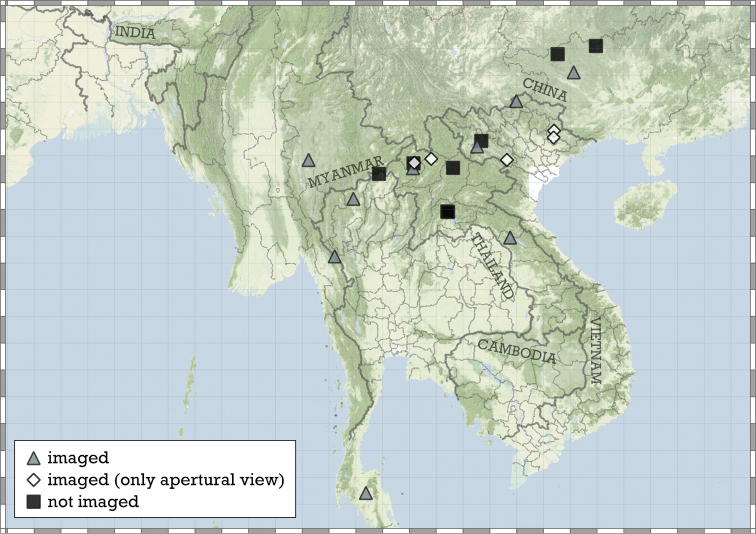
Distribution of *Angustopilaelevata* (Thompson & Upatham, 1997).

##### Remarks.

When *Angustopilasubelevata* was described, we relied on the original description of *A.elevata* only. Although it was evident that the two species were very similar, we considered the differences (*A.elevata* had a more slender shell, a deeper umbilicus, and was lacking spiral striae on its base) and the nearly 1000-km distance sufficient to describe *A.subelevata* as new. The newly collected shells from the type locality of *A.elevata* showed that it had weak spiral striae on the base. Furthermore, the geographical populations examined between the type localities of *A.elevata* and *A.subelevata* indicated that only minor conchological differences exist. Although the area spans nearly 2000 kilometres in a straight line from southern Thailand to Guangxi Province in China, the populations listed here show little variation in terms of conchological characters. Thus, we now treat them as a single species.

#### ﻿ Angustopila halongensis

Taxon classificationAnimaliaStylommatophoraGastrocoptidae

Páll-Gergely & Vermeulen sp. nov.

55490C30-E4D9-59D4-8E06-9A75AACA7E6E

https://zoobank.org/A7460B3E-084C-4AE1-BABB-08875616D8FB

Figs 15 , 16

##### Type material.

***Holotype***: Vietnam • 1 empty shell (H: 0.96 mm, D: 0.89 mm); Quang Ninh Province, Halong Bay, Cap La Cave, soil deposited through roof in pristine cave, vegetation outside cave tall and woody; 20°51.79'N, 107°13.54'E; 7 Mar. 2018; J.J. Vermeulen & K. Anker leg.; HNHM 103473.

***Paratypes***: Vietnam • 1094 shells; same data as for the holotype; JJV 17631 • 10 shells; same data as for the holotype; coll. HA • 33 shells; Quang Ninh Province, Halong Bay area, Dao Bo Hon, Song Sot Cave, drift material washed together over sinkhole in cave; 20°50.83'N, 107°05.67'E; 2 Oct. 1998; J.J. Vermeulen & A.J. Whitten leg.; JJV 6263 • 3 shells; Quang Ninh Province, Halong-Campha area, limestone hill S of Halong, mainly regrowth and bamboo thickets; 20°57.00'N, 107°04.72'E; 28 Sep. 1998; J.J. Vermeulen & A.J. Whitten leg.; JJV 6246 • 2 shells; Quang Ninh Province, Halong Bay area, unnamed small island east of Cong Do Island, sparsely-vegetated limestone rocks; 20°51.84'N, 107°13.19'E; 4 Oct. 1998; J.J. Vermeulen & A.J. Whitten leg.; JJV 6249 • 2 shells; Haiphong Province, Cat Ba Island, Cave Qua Vang, inside cave, large, ecologically intact active cave with speleothems; 20°48.64'N, 107°04.64'E; 60 m a.s.l.; 6 Jun. 2017; JJV 16611 • 1 shell; Haiphong Province, Cat Ba Island, Cave Qua Vang, around cave entrance, rocky limestone slope with low, mature forest; 20°48.64'N, 107°04.64'E; 100 m a.s.l.; 6 Jun. 2017; J.J. Vermeulen & K. Anker leg.; JJV 16612 • 3 shells (one of them is figured); Haiphong Province, Cat Ba Island, lake Ao Ek, high and damp primary forest (locality code: Vietnam 06); 20°48.05'N, 107°01.33'E; 26 Sep. 1998; W.J.M. Maassen leg.; RMNH.5006719 • 3 shells; Haiphong Province, Cat Ba Island, Cave Trung Trang, around cave entrance, steep limestone cliffs with vegetated ledges; 20°47.30'N, 106°59.84'E; 50 m a.s.l.; 6 Jun. 2017; J.J. Vermeulen & K. Anker leg.; JJV 16617 • 193 adult + 2 j/b shells; Haiphong Province, Cat Ba Island, Cave Minh Chou, inside cave, cave with freshwater stream flowing into the sea, much disturbed by water extraction and concrete paths; 20°45.21'N, 107°0.75'E; 50 m a.s.l.; 5 Jun. 2017; J.J. Vermeulen & K. Anker leg.; JJV 16610 • 1 shell; Quang Ninh Province, Halong Bay area, Hang Tai Island, southern end, beach drift sample; 20°46.24'N, 107°07.77'E; 4 Oct. 1998; J.J. Vermeulen & A.J. Whitten leg.; JJV 6250.

##### Diagnosis.

A small to medium-sized, conical, or concave-conical species of variable shell height with a relatively large, ovate-oblong, toothless aperture, and strong spiral striation.

##### Description.

Shell small to medium-sized for the genus, higher than wide or very rarely wider than high; pale grey, conical or rarely concave-conical with regularly increasing whorls (sometimes except for the last one), shell height variable, last whorl widest from standard apertural view; protoconch consists of 1.5 whorls, with strong spiral striae; teleoconch with some fine, weak, irregular radial growth lines and much stronger, elevated, irregularly spaced spiral striae of variable density (ca. 16–20 on body whorl in standard apertural view); whorls 4–4.5, rounded; aperture moderately oblique to shell axis from lateral view; umbilicus relatively narrow; aperture relatively wide, ovate-oblong with straight parietal part; peristome expanded, not reflected; parietal callus not detached from penultimate whorl; aperture toothless.

##### Measurements (in mm).

H = 0.79–0.94, D = 0.81–0.95, H/D*100 = 98.8–120.7 (*n* = 12), RUD = 21.0–25.6 (*n* = 5).

##### Differential diagnosis.

Similar to *A.elevata*, but has a comparatively larger aperture and more regular conical shell shape and denser spiral striation on the body whorl. *Angustopilawerneri* sp. nov. is larger with narrower aperture and wider umbilicus.

##### Etymology.

The specific epithet refers to the wide distribution of this new species in the Halong Bay Area, northern Vietnam.

##### Distribution.

This species is known only from the Halong Bay Area, Vietnam (Fig. 17).

**Figure 15.**
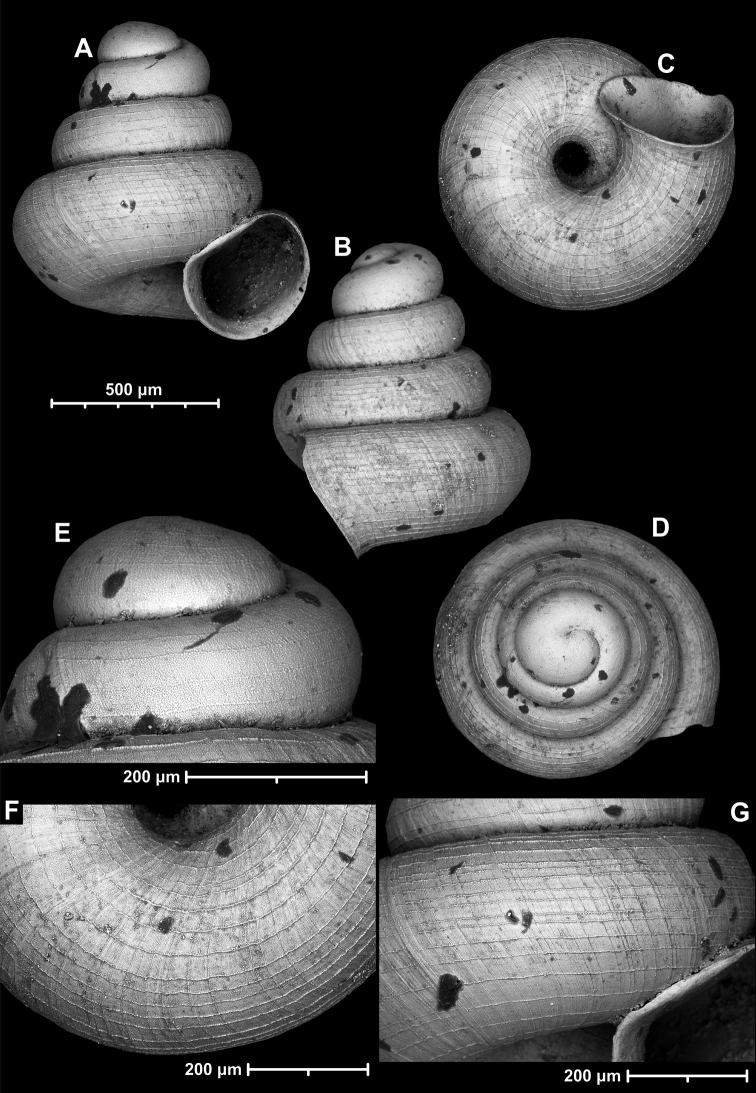
*Angustopilahalongensis* Páll-Gergely & Vermeulen, sp. nov., (holotype, HNHM 103473). Apertural (**A**), lateral (**B**), ventral (**C**) and apical (**D**) sides of the shell; sculpture on the protoconch (**E**), ventral (**F**) and frontal (**G**) surface of the body whorl.

**Figure 16.**
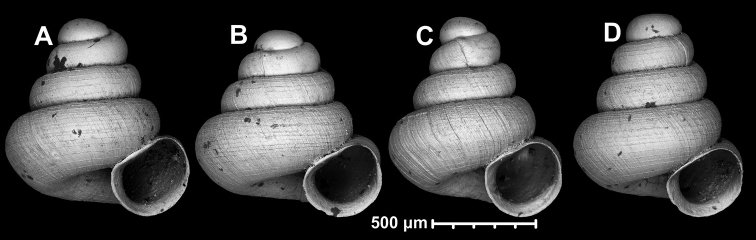
Variability of *Angustopilahalongensis* Páll-Gergely & Vermeulen, sp. nov., (JJV 17631) **A** holotype **B–D** paratypes.

**Figure 17.**
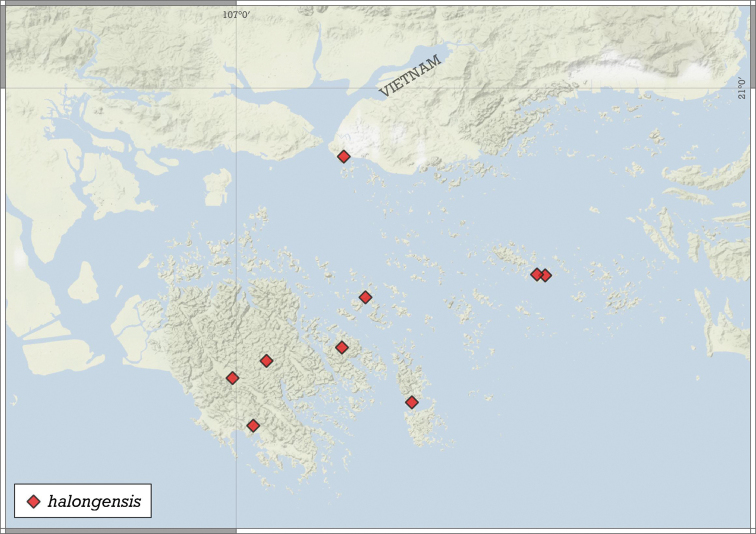
Distribution of *Angustopilahalongensis* Páll-Gergely & Vermeulen, sp. nov.

#### ﻿ Angustopila milium

Taxon classificationAnimaliaStylommatophoraGastrocoptidae

(Benson, 1853)

BC250699-DFE3-5C0F-91BA-34416297D0DC

Fig. 18

Cyclostoma Milium Benson, 1853: 285.
Hydrocena milium — W.T. Blanford 1864: 464. (perhaps Georissa); Godwin-Austen 1872: 515, pl. 30, fig. 3.
Cyathopoma milium — W.T. Blanford 1869: 178.
Georissa milium — Pfeiffer 1875–1876: 292.
Acmella milium — Gude 1921: 362; Ramakrishna et al. 2010: 111.
Angustopila milium — Das et al. 2021: 329; Preece et al. 2022: 154, fig. 70A.

##### Type locality.

“in muscis arborum ad Musmai, prope Cherra-poonjee” (India, Meghalaya, Cherrapunji, approximate GPS coordinates: 25°16.6'N, 91°44'E).

##### Material examined.

One syntype, possibly holotype, with no locality data (UMZC I.103805). An old label in unknown handwriting reads “*Hydrocenamilium* Bs.” and on the reverse side: “original shell described”.

##### Distribution.

Known from a single shell only (Fig. 19).

**Figure 18.**
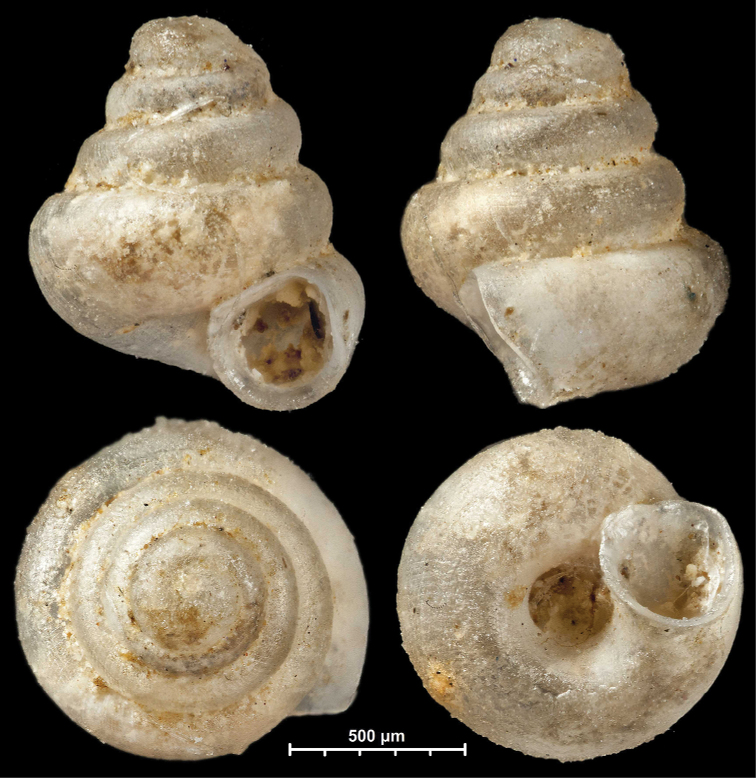
*Angustopilamilium* (Benson, 1853) (syntype, UMZC I.103805). Photos: H. Taylor (NHM). Scale bar 1 mm.

**Figure 19.**
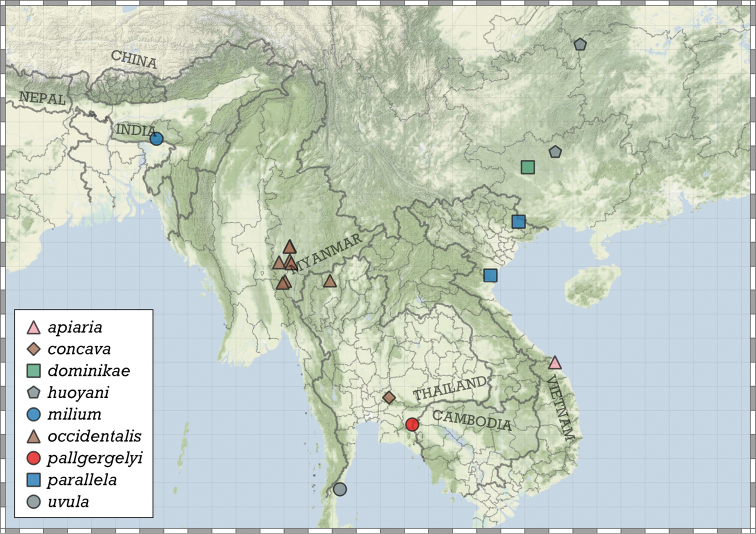
Distribution of *Angustopila* species.

##### Remarks.

*Angustopilamilium* was described as a *Cyclostoma* (caenogastropod) species. Even Godwin-Austen (1872) was convinced that this was an operculate species when he examined Benson’s type specimens. Gude (1914) considered it as juvenile *Boysidiaplicidens*. However, the combination of the shell characters (small, conical shell, narrow, deep umbilicus, toothless, subcircular aperture, expanded aperture, sparsely arranged spiral striae) indicates that this species represents an adult hypselostomatid, which undoubtedly fits to the genus *Angustopila*, significantly extending the genus distribution.

Here, we do not present a formal redescription because the type specimens could not be examined by SEM, and therefore, some important details cannot be seen. However, due to the large geographic distance between *A.milium* and other species discussed herein, it is highly unlikely that *A.milium* would be conspecific with any of them.

#### ﻿ Angustopila thersites

Taxon classificationAnimaliaStylommatophoraGastrocoptidae

Páll-Gergely & Vermeulen sp. nov.

6790B0D3-F016-57DD-BAEE-D4F3E892440A

https://zoobank.org/C9601C1B-14C6-4AED-8DE9-36D7B0C3DAF4

**Figure 20.**
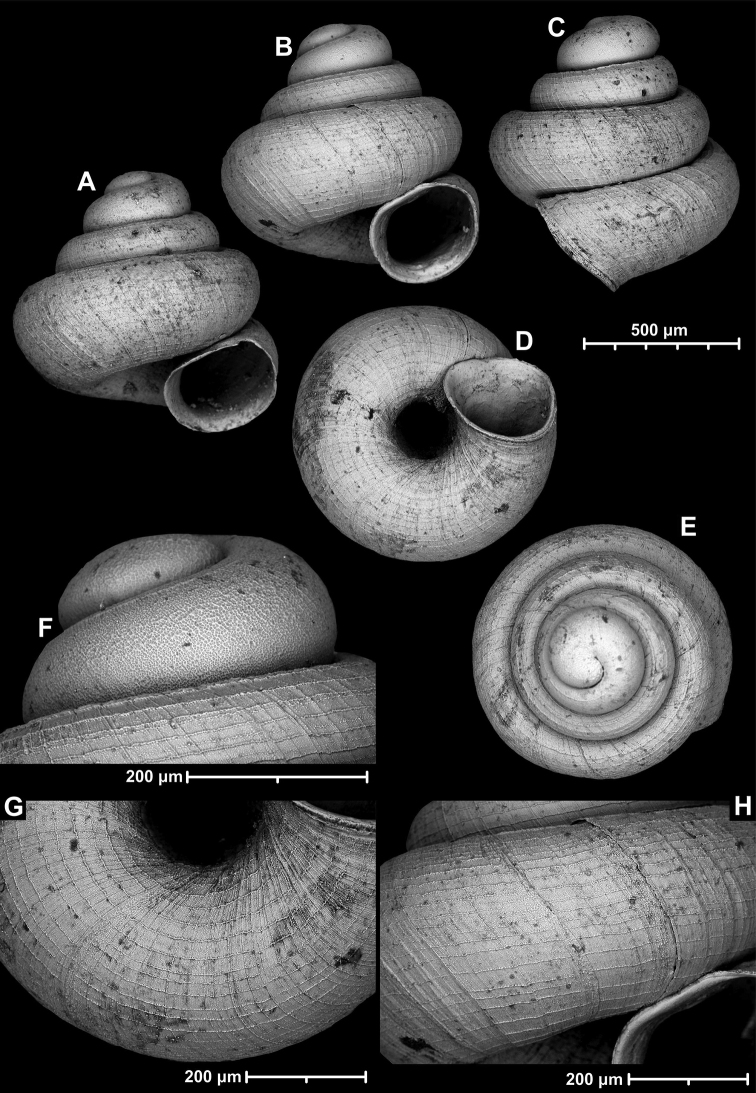
*Angustopilathersites* Páll-Gergely & Vermeulen, sp. nov. **A** paratype (RMNH 347761) **B–H** holotype (RMNH 347760). Apertural (**A, B**), lateral (**C**), ventral (**D**) and apical (**E**) sides of the shell; granular sculpture on the protoconch (**F**), ventral (**G**) and frontal (**H**) surface of the body whorl.

**Figure 21.**
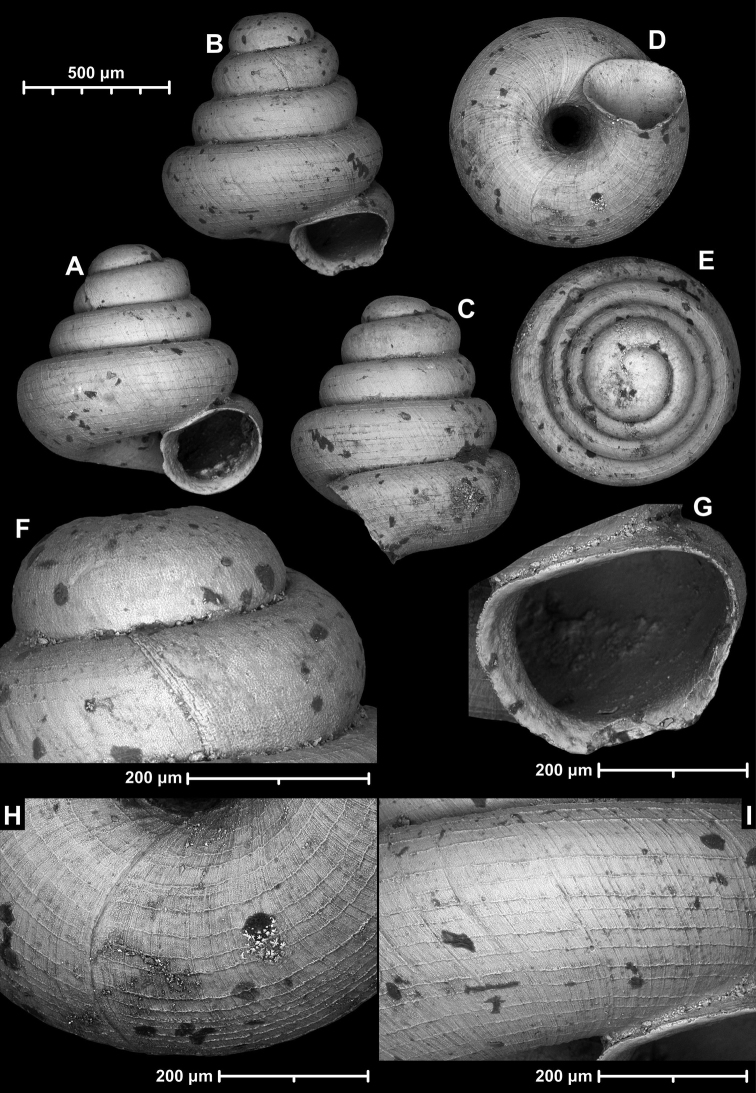
*Angustopilathersites* Páll-Gergely & Vermeulen, sp. nov., sample WMVT.0327 (RMNH 347764) **A** specimen 2 **B–I** specimen 1. Apertural (**A, B**), lateral (**C**), ventral (**D**) and apical (**E**) sides of the shell; granular sculpture on the protoconch (**F**), aperture (**G**), ventral (**H**) and frontal (**I**) surface of the body whorl.

Figs 20 , 21 , Suppl. material 3: figs S7, S8

##### Type material.

***Holotype***: Vietnam • 1 empty shell (H: 0.85 mm, D: 0.86 mm); Quang Ninh Province, Halong Bay area, Phao Trong Island (locality code: WMVT.0333); approx. GPS code: 20°49.80'N, 107°08.32'E; 11 Sep. 2003; W.J.M. Maassen leg.; RMNH 347760.

***Paratypes***: Vietnam • 9 adult shells; same data as for holotype; RMNH 347761 • 3 shells; Quang Ninh Province, Halong Bay area, Luoi Liem Island; 20°49.67'N, 107°09.90'E (locality code: WMVT.0334); 12 Sep. 2003; W.J.M. Maassen leg.; RMNH 347762 • 1 shell; Haiphong Province, Cat Ba Island, Cave Qua Vang, around cave entrance, rocky limestone slope with low, rather mature forest; 20°48.64'N, 107°04.64'E; 100 m a.s.l.; 6 Jun. 2017; J.J. Vermeulen & K. Anker leg.; JJV 16621 • 7 shells; Haiphong Province, Cat Ba Island, Cave Qua Vang, inside cave, large, little disturbed active cave with speleothems; 20°48.64'N, 107°04.64'E; 60 m a.s.l.; 6 Jun. 2017; J.J. Vermeulen & K. Anker leg.; JJV 16620 • 8 shells; Haiphong Province, Cat Ba Island, Cave Xa Bac, around cave entrance; 20°50.07'N, 106°58.61'E; 30 m a.s.l.; 9 Jun. 2017; J.J. Vermeulen & K. Anker leg.; JJV 16599 • 15 shells; Quang Ninh Province, Halong Bay area, Dao Bo Hon, Song Sot Cave, drift material washed together over sinkhole in cave; 20°50.83'N, 107°05.67'E; 2 Oct. 1998; J.J. Vermeulen & A.J. Whitten leg.; JJV 17654 (ex JJV 6237) • 1 shell; Haiphong Province, Cat Ba Island, along trail from headquarters to Viet Hai, along Ao Eck, sampling starting at coordinates, until 0.5 km beyond Ao Eck; 20°48.07'N, 107°00.83'E; 100 m a.s.l.; 4 Jun. 2017; J.J. Vermeulen & K. Anker leg.; JJV 16618 • 4 shells; Haiphong Province, Cat Ba Island, Cave Uy Ban, around cave entrance; 20°46.39'N, 107°00.88'E; 40 m a.s.l.; 9 Jun. 2017; J.J. Vermeulen & K. Anker leg.; JJV 16623 • 7 shells; Haiphong Province, Halong Bay area, unnamed island off E Coast Cat Ba, south facing bay with beach and densely vegetated limestone scree slope; 20°45.19'N, 107°04.45'E (approximate GPS data); 1 Oct. 1998; J.J. Vermeulen & K. Anker leg.; JJV 6236 • 2 shells; Haiphong Province, Cat Ba Island, large, somewhat disturbed, active cave with speleothems; 20°50.06'N, 106°55.91'E; 0–50 m a.s.l.; 7 Jun. 2017; J.J. Vermeulen & K. Anker leg.; JJV 16622 • 4 shells; Quang Ninh Province, Halong Bay area, unnamed island 1.8 km W of S point Cong Tai Island, steep limestone slope bordering beach, dense vegetation; 20°52.23'N, 107°18.25'E; 3 Oct. 1998; J.J. Vermeulen & A.J. Whitten leg.; JJV 6239 • 25 shells; Haiphong Province, Cat Ba Island, Cave Trung Trang, around cave entrance, steep limestone cliffs with vegetated ledges; 20°47.30'N, 106°59.84'E; 50 m a.s.l.; 6 Jun. 2017; J.J. Vermeulen & K. Anker leg.; JJV 16619 • 5 shells; same data as for preceding; coll. HA.

##### Additional material.

Vietnam • 2 j/b shells; same data as for holotype; RMNH 347763 • 7 shells; Quang Ninh Province, Halong Bay area, Thay Cave on Congfo Island, collected inside the cave; 20°52.07'N, 107°12.06'E (locality code: WMVT.0327); 6 Sep. 2003; W.J.M. Maassen leg.; RMNH 347764.

##### Diagnosis.

A small to medium-sized, concave-conical species with irregularly growing whorls, strong spiral striation, very narrow umbilicus, and a strongly oblique, toothless aperture.

##### Description.

Shell small to medium-sized for the genus, mostly wider than high or rarely higher than wide (see population WMVT.0327), concave-conical, tightly and asymmetrically coiled, seemingly globular due to narrowing last whorl; body whorl widest in apertural view (although slightly wider than penultimate whorl) or penultimate whorl widest; protoconch bulbous, consists of ca. 1.25 whorls, with weak spiral striation preceding the first teleoconch whorl; teleoconch with irregular radial growth lines and dense, strong, rather regular wavy spiral striation (ca. 22 spiral striae on body whorl in apertural view); whorls 3.5–3.75, rounded; aperture curved and strongly oblique to shell axis in lateral view; aperture ovate-oblong; aperture tucked tightly under inflated penultimate whorl in apertural view; peristome slightly expanded, not reflected; not attached to penultimate whorl; umbilicus very narrow, ca. 1/5 of shell width; aperture toothless.

##### Measurements (in mm).

H = 0.75–0.87, D = 0.81–0.93, H/D*100 = 87.2–101.2 (*n* = 10), RUD = 23.5–24.1 (*n* = 3) (populations except for WMVT.0327); H = 0.81–0.85, D = 0.87–0.93, H/D*100, = 102.4–114.8, RUD = 22.2–24.7 (*n* = 2, WMVT.0327).

##### Differential diagnosis.

The most similar species is *A.megastoma* sp. nov., which also occurs sympatrically with this species. Distinguishing these two species is sometime difficult because both have quite an irregular shell, and exhibit a rather large intraspecific variability. Nevertheless, there are several differences as follows: *A.megastoma* sp. nov. is larger, and has a generally lower spire; its aperture is less rounded (rather semilunar because the body whorl is more attached to the penultimate whorl); the spiral striation of *A.megastoma* sp. nov. is denser and much fainter, in fact it is nearly invisible under the stereo microscope (only visible with SEM); the last whorl of *A.megastoma* sp. nov. is wider compared to the penultimate whorl than that of *A.thersites* sp. nov. (in the latter species often the penultimate whorl is wider than the last one in apertural view). See also under *A.tonkinospiroides* sp. nov.

##### Etymology.

This new species is named after Thersites (*Θερσίτης* = the ugliest Greek soldier laying siege to Troy) referring to the irregularly growing whorls.

##### Distribution.

**Figure 22.**
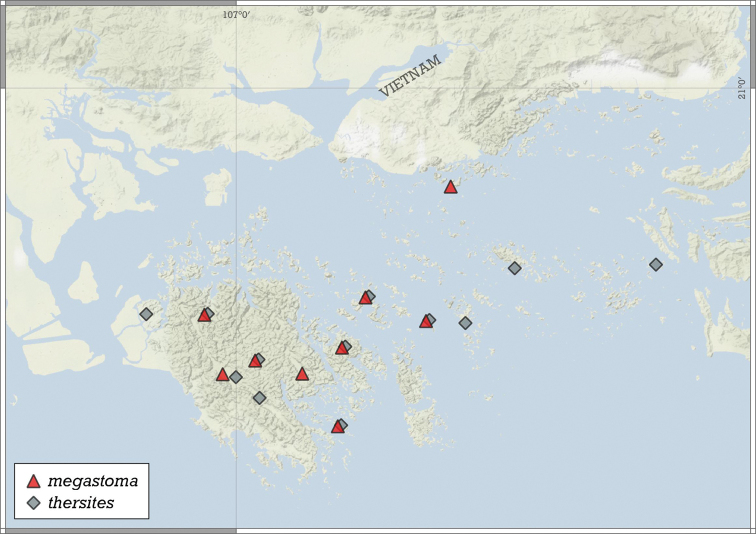
Distribution of *Angustopila* species.

This species is known from several sites in the Halong Bay Area, northern Vietnam (Fig. 22).

##### Remarks.

Shells from sample WMVT.0327 are unusually high-spired and are less concave-conical (some of them are even conical) than typical *A.thersites* sp. nov. shells. Although the differences in shell shape between most shells of sample WMVT.0327 and other *A.thersites* sp. nov. shells are obvious, the shell with the lowest spire of sample WMVT.0327, and the shell with the highest spire of the other *A.thersites* sp. nov. samples are very similar. In addition, the strong spiral striation, the aperture shape, the narrow umbilicus, and the very oblique aperture suggests that they are conspecific with *A.thersites* sp. nov.

#### ﻿ Angustopila tonkinospiroides

Taxon classificationAnimaliaStylommatophoraGastrocoptidae

Páll-Gergely & Vermeulen sp. nov.

8A93224F-7F81-5030-9AF5-78EEDA5526D1

https://zoobank.org/74271797-1383-4115-AADB-1436FBB11313

Fig. 23

##### Type material.

***Holotype***: Vietnam • 1 empty shell (H: 1.07 mm, D: 0.88 mm); Quang Ninh Province, Halong Bay area, Dao Bo Hon, Song Sot Cave, drift material washed together over sinkhole in cave; 20°50.83'N, 107°05.67'E; 2 Oct. 1998; J.J. Vermeulen & A.J. Whitten leg.; HNHM 100179.

***Paratypes***: Vietnam • 400 shells; same data as for holotype; JJV 17655 (ex JJV 6263) • 10 shells; same data as for holotype; coll. HA • 31 shells; Haiphong Province, Cat Ba Island, Cave Minh Chou, inside cave, cave with freshwater stream flowing into the sea, much disturbed by water extraction and concrete paths; 20°45.21'N, 107°0.75'E; 50 m a.s.l.; 5 Jun. 2017; J.J. Vermeulen & K. Anker leg.; JJV 18885 (ex JJV 16610).

##### Diagnosis.

A medium-sized to large, toothless *Angustopila* species with a conical spire, wide penultimate whorl, and a narrow, subovate-shaped umbilicus.

##### Description.

Shell normal- to large-sized for the genus, (much) higher than wide; pale grey; shell shape conical, penultimate whorl is the widest in standard apertural view; protoconch consists of 1.5 whorls, with spiral striation preceding the first teleoconch whorl; teleoconch finely ornamented with usually weak, irregularly spaced radial growth lines crossed by fine rows of somewhat stronger, equidistantly-spaced spiral threads (ca. 20 in standard apertural view); whorls 4.5–4.75, rounded, but rather irregular, with some traces of compression of the convex whorls from lateral/umbilical direction; aperture slightly curved and oblique to shell axis from lateral view; umbilicus narrow, eccentrically teardrop-shaped due to unusual coiling of body whorl, concentric spiral striation intensified around umbilicus; aperture subovate with straight parietal side; peristome slightly expanded, not reflected, rather sharp; parietal callus slightly protruding, detached from penultimate whorl; aperture toothless, a fine swelling discernible just behind peristome, running parallel with it.

##### Measurements (in mm).

H = 0.98–1.16, D = 0.85–0.95, H/D*100 = 104.3–128.9 (*n* = 14), RUD = 22.7–28.9 (*n* = 4).

##### Differential diagnosis.

The irregularly growing whorls, the higher than wide, rather large shell, the toothless aperture and the teardrop-shaped umbilicus distinguishes this species from its congeners. *Angustopilathersites* sp. nov. is smaller, and has a concave-conical, more irregularly increasing shell. *Angustopilafratermajor* sp. nov. is smaller, has a parietal tooth, and possesses a wider umbilicus. *Tonkinospira* species are usually larger, depressed-conical in shape and have denser spiral striation on both the protoconch and teleoconch.

##### Etymology.

First we considered this species to belong to the genus *Tonkinospira.* Due to the tonkinospirid aspects (i.e., large conical shell, regularly growing whorls, superficial microstructure and lack of apertural dentition), the specific epithet reflects this similarity.

##### Distribution.

This new species is known from two sites in the Halong Bay area in northern Vietnam (Fig. 24).

**Figure 23.**
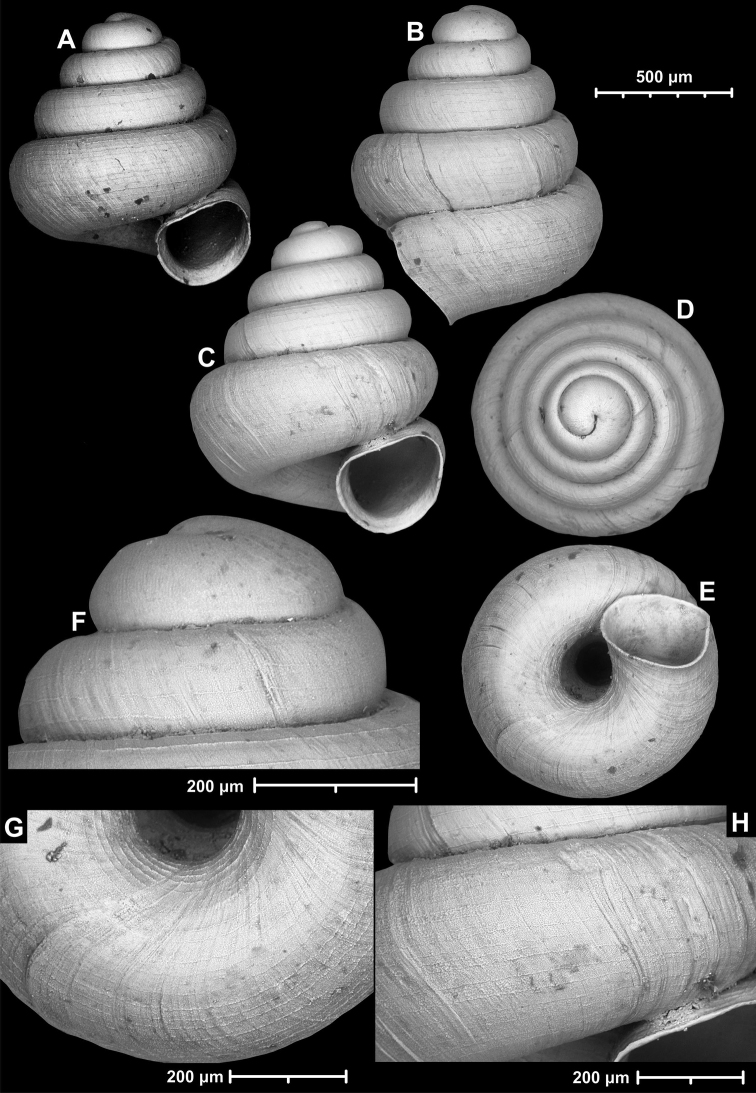
*Angustopilatonkinospiroides* Páll-Gergely & Vermeulen, sp. nov., JJV 6263. Paratype (**A**) and holotype (HNHM 100179) (**B–H**). Apertural (**A, C**), ventral (**E**), apical (**D**) and lateral (**B**) sides of the shell; sculpture on the protoconch (**F**), ventral (**G**) and frontal (**H**) surface of the body whorl.

**Figure 24.**
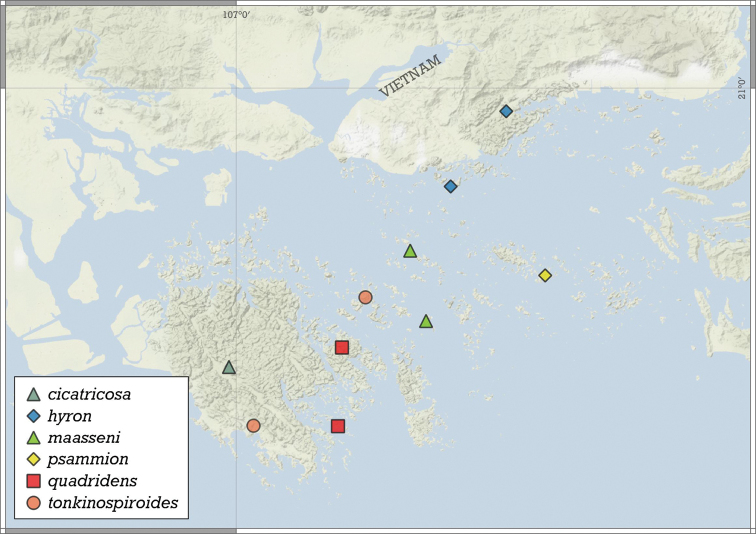
Distribution of *Angustopila* species in the Halong Bay area.

#### ﻿ Angustopila werneri

Taxon classificationAnimaliaStylommatophoraGastrocoptidae

Páll-Gergely & Hunyadi sp. nov.

7198D467-5C3C-5CA0-872B-7CD41DB37F5E

https://zoobank.org/FD6D047C-701F-4283-83D2-58450746D738

Figs 25 , 26

##### Type material.

***Holotype***: Vietnam • 1 empty shell (H: 1.09 mm, D: 1.01 mm); Lạng Sơn Province, Bắc Sơn District, Quinh Son, southern edge of the village, left side of road no. 241 (locality code: 2020/49); 21°53.41'N, 106°20.94'E; 400 m a.s.l.; 18 Feb. 2020; A. Hunyadi leg.; HNHM 105280.

***Paratypes***: Vietnam • 10 adult shells; same data as for holotype; coll. HA.

##### Additional material.

Vietnam • 4 juvenile shells; same data as holotype; coll. HA • 7 shells; Lạng Sơn Province, Hữu Lũng District, Hữu Liên, 1400 m west from Đȏng Lâm along road no. 241 (locality code: 2020/53); 21°41.91'N, 106°21.77'E; 210 m a.s.l.; 19 Feb. 2020; A. Hunyadi leg.; coll. HA.

##### Diagnosis.

A medium- to large-sized, concave-conical *Angustopila* species with a comparatively small, toothless aperture and a wide umbilicus.

##### Description.

Shell medium to large-sized for the genus, slightly higher than wide or slightly wider than high; concave-conical; last whorl widest from standard apertural view; protoconch consists of 1.25 whorls, with strong spiral striae; teleoconch with some fine, weak, radial growth lines and much stronger, elevated, dense, mostly regularly spaced spiral striae (ca. 18–20 on body whorl in standard apertural view); whorls 4.25, rounded, or body whorl slightly shouldered; aperture strongly oblique to shell axis from lateral view; umbilicus wide; aperture relatively small, subcircular, irregular in shape, parietal part straight or convex; peristome slightly expanded, not reflected; parietal callus considerably detached from penultimate whorl; aperture toothless.

##### Measurements (in mm).

H = 0.94–1.09, D = 0.92–1.01, H/D*100 = 97–110.4 (*n* = 8), RUD = 29.7–34.7 (*n* = 4).

##### Differential diagnosis.

The most similar species in terms of shell and aperture shape is *A.elevata*, which is much smaller, has a more conical (rather than concave-conical) shell resulting in a narrower umbilicus, and less prominent radial sculpture. See also under *A.halongensis* sp. nov.

##### Etymology.

The new subspecies is dedicated to and named after Ervin Werner (1936–2021), the prominent botanist and biology teacher in secondary school of the first author.

##### Distribution.

The two known populations are located ca. 20 kilometres from each other in Lạng Sơn Province, northern Vietnam (Fig. 27).

**Figure 25.**
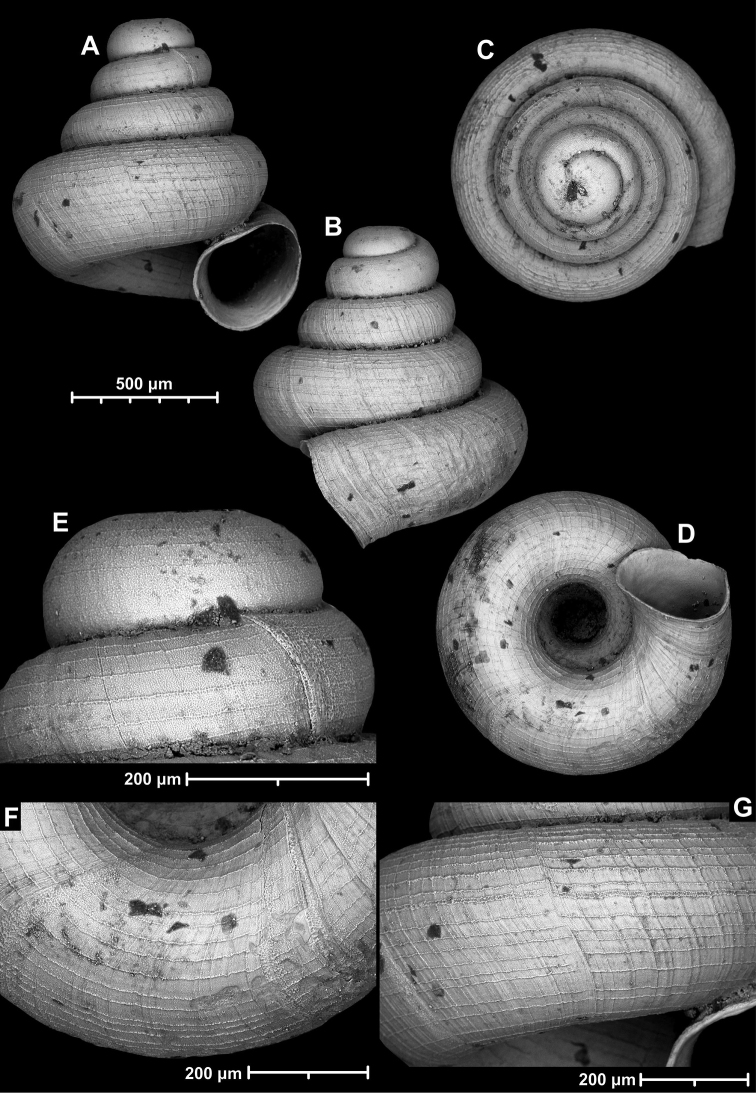
*Angustopilawerneri* Páll-Gergely & Hunyadi, sp. nov., (holotype, HNHM 105280). Apertural (**A**), lateral (**B**), apical (**C**), and ventral (**D**) sides of the shell; sculpture on the protoconch (**E**), ventral (**F**) and frontal (**G**) surface of the body whorl.

**Figure 26.**
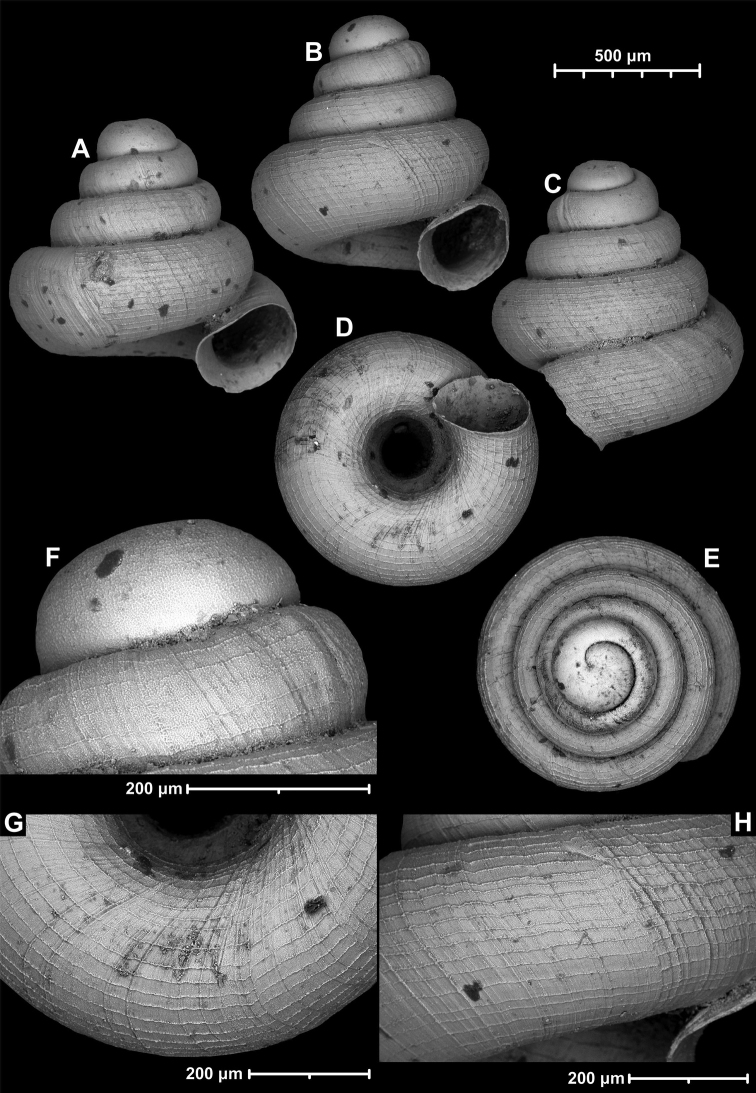
*Angustopilawerneri* Páll-Gergely & Hunyadi, sp. nov. 2020/53 (paratypes) **A** specimen 2, **B–H** specimen 1. Apertural (**A, B**), lateral (**C**), ventral (**D**) and apical (**E**) sides of the shell; sculpture on the protoconch (**F**), ventral (**G**) and frontal (**H**) surface of the body whorl.

**Figure 27.**
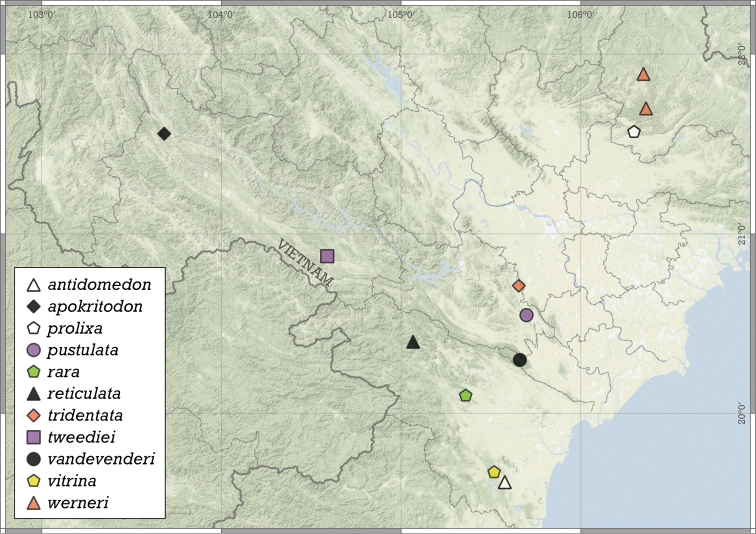
Distribution of *Angustopila* species.

##### Remarks.

Shells from locality 2020/53 have a comparatively smaller aperture than those of the typical shells, but no other differences were found.

### ﻿Species with a single parietal tooth

**Remarks.***Angustopilamegastoma* sp. nov. may lack a parietal tooth. *Angustopilaapiostoma* sp. nov. may rarely possess a parietal tooth.

The *Angustopila* species presented as new in this study are distinguished based on unique character states or a unique combination of character states consisting of shell and aperture shape, apertural barriers, and sculpture. The most challenging effort was to distinguish between species possessing a concave-conical or conical shell (rarely conical-globular) with a single parietal tooth (hereafter termed “difficult *Angustopila*”). The criteria used to delimit species within this group are listed below. So far, there are two known *Angustopila* species having a conical shell and a well-developed parietal tooth: *A.fabella* and *A.singuladentis*. Since the latter falls within the morphological variability of the former, it is treated as a junior synonym. Typical *A.fabella* shells are characterised by a high parietal tooth, which is bigger than in most other populations of “difficult *Angustopila*”. Nevertheless, some “difficult *Angustopila*” specimens we examined have a similarly large and extensive parietal tooth. Thus, the name *A.fabella* can be applied to most populations of “difficult *Angustopila*”.

(1) Strict sympatry: When two or more ‘morphotypes’ of “difficult *Angustopila*” live in sympatry, they cannot belong to the same species. This was the case for *Angustopilamargaritarion* sp. nov., which lived in sympatry with *Angustopilafabella*.

(2) Allopatry: When a ‘morphotype’ of “difficult *Angustopila*” was geographically surrounded by another ‘morphotype’. This was the case with *Angustopilavandevenderi* sp. nov., which was surrounded by populations of *A.fabella*. Additionally, *Angustopilababel* sp. nov. is also treated as a species different from *A.fabella* because in the Halong Bay area, besides the *A.babel* sp. nov.-like populations (conical, narrow umbilicus, parietal tooth reaching parietal callus), there are three lots with different traits (concave-conical, wider umbilicus, parietal tooth not reaching parietal callus) (identified as *A.fabella*).

(3) Adjunct character: In addition to the conical shell and the single parietal tooth, there is another character that distinguishes a species from other *A.fabella* populations. For example, *A.cavicola* sp. nov. has an unusually expanded peristome and a comparatively wide aperture. We note that the non-type sample of *A.cavicola* sp. nov. (2019/118) lives in sympatry with *A.fabella*.

#### ﻿ Angustopila babel

Taxon classificationAnimaliaStylommatophoraGastrocoptidae

Páll-Gergely & Vermeulen sp. nov.

92466D68-DA31-5792-8290-18A068703ACE

https://zoobank.org/405BA4E4-A738-488E-8061-4EDEA99667E3

Figs 28 , 29C–E

##### Type material.

***Holotype***: Vietnam • 1 empty shell (H: 1.07 mm, D: 0.97 mm); Quang Ninh Province, Halong Bay area, unnamed island in Dau Moi Temple area (locality code: WMVT.0328); 20°55.69'N, 107°09.40'E; 13 Sep. 2003; W.J.M. Maassen leg.; RMNH 347765.

***Paratypes***: Vietnam • 12 shells; same data as for holotype; RMNH 347766 • 491 shells; Quang Ninh Province, Halong Bay, Cap La Cave, deposit of soil fallen in through roof in pristine cave, vegetation outside cave tall and woody; 20°51.79'N, 107°13.54'E; 7 Mar. 2018; J.J. Vermeulen & K. Anker leg.; JJV 17632 • 10 shells; same data as for preceding; coll. HA • 20 shells; Quang Ninh Province, Halong Bay area, Dao Bo Hon, Song Sot Cave, drift material washed together over sinkhole in cave; 20°50.83'N, 107°05.67'E; J.J. Vermeulen & A.J. Whitten leg.; 2 Oct. 1998; JJV 6220 • 12 shells; Quang Ninh Province, Halong Bay area, Dao Bo Hon, Song Sot Cave, guano enriched sediments in cave; 20°50.83'N, 107°05.67'E; 2 Oct. 1998; J.J. Vermeulen & A.J. Whitten leg.; JJV 6221 • 1 shell; Haiphong Province, Cat Ba Island, Cave Qua Vang, around cave entrance, rocky limestone slope with low, somewhat mature forest; 20°48.64'N, 107°04.64'E; 100 m a.s.l.; 6 Jun. 2017; J.J. Vermeulen & K. Anker leg.; JJV 17657 (ex JJV 16612) • 20 shells; Haiphong Province, Cat Ba Archipelago, Dao Dau Be, rocks and small soil deposits bordering beach; 20°45.68'N, 107°07.47'E; 10 m a.s.l.; 8 Jun. 2017; J.J. Vermeulen & K. Anker leg.; JJV 16591 • 73 shells; Haiphong Province, Cat Ba Island, Cave Qua Vang, inside cave, large, ecologically intact active cave with speleothems; 20°48.64'N, 107°04.64'E; 60 m a.s.l.; 6 Jun. 2017; J.J. Vermeulen & K. Anker leg.; JJV 16590 • 1 shell; Quang Ninh Province, Halong Bay area, May Den Island, Tam Cung Cave, densely-vegetated limestone hill, near cave; approx. GPS coordinates: 20°52.33'N, 107°07.09'E; 3 Oct. 1998; J.J. Vermeulen & A.J. Whitten leg.; JJV 6248 • 2 shells; Quang Ninh Province, Halong-Campha area, 2.5 km SW Quang Hanh, foot of limestone cliff with degraded regrowth, near gardens; 20°59.750'N, 107°12.750'E; 29 Sep. 1998; J.J. Vermeulen & A.J. Whitten leg.; JJV 6218.

##### Diagnosis.

A medium-sized to large *Angustopila* species with a conical shell, regularly growing whorls, a strong parietal fold reaching the peristome and a narrow umbilicus.

##### Description.

Shell medium-sized to large-sized for the genus, higher than wide, very rarely slightly wider than high; conical, body whorl widest in standard apertural view; protoconch consists of 1.25 whorls with weak spiral striae preceding the first teleoconch whorl; teleoconch with very fine irregularly spaced, weak radial growth lines crossed with equally strong, rather regularly and sparsely-spaced spiral striae (ca. 12 on body whorl from apertural view); on both ventral and dorsal surfaces of body whorl spiral lines dominant or some radial lines are of comparable strength; sculpture overall weaker than in most other *Angustopila* species; whorls ca. 3.25–4, rounded or very slightly shouldered; aperture oblique to shell axis from lateral view; umbilicus narrow; aperture ovate-oblong, parietal callus straight; sinulus very wide, weakly isolated due to the rather low parietal tooth; peristome expanded, not reflected; parietal callus only very slightly detached from penultimate whorl, not smeared onto it; parietal tooth moderately elevated, of normal length, reaches parietal callus (in some, probably subadult shells it does not), perpendicular to parietal wall.

##### Measurements (in mm).

H = 0.94–1.17, D = 0.92–1.16, H/D*100 = 89.0–121.7 (*n* = 18), RUD = 22.1–25.2 (*n* = 8).

##### Differential diagnosis.

Shells of *A.fabella* lots in the Halong Bay area differ from *A.babel* sp. nov. by having a concave-conical shell (instead of the conical shape of *A.babel* sp. nov.), a wider umbilicus and a more deeply set parietal tooth.

From *A.fabella* populations living outside of the Halong Bay area, *A.babel* sp. nov. can be distinguished by the following traits: *Angustopilababel* sp. nov. is usually larger, has a less pointed (rather blunt) apex, and a comparatively larger aperture, a narrower umbilicus, and the parietal tooth reaches the parietal callus. Some *A.fabella* populations are, however, strikingly similar in shape. For example, sample 2020/58, which is smaller and has a more deeply situated parietal tooth, and sample 2020/32, which is also smaller, and has a more pointed apex and a smaller aperture. See also under *A.fratermajor* sp. nov.

##### Etymology.

Named after the conical shape, resembling the Tower of Babel as depicted by Pieter Breughel the Elder (ca. 1563). To be used as a noun in apposition.

##### Distribution.

Known from several populations in the Halong Bay area only (Fig. 11).

##### Remarks.

Conical or concave-conical shells with a single parietal tooth belong to two distinct morphological groups in the Halong Bay Area. Both types would otherwise fit the morphological continuum of the widespread and variable *Angustopilafabella*. However, since the two types are clearly distinct within such a small area, only one of them is identified *A.fabella*, and the other one must be a distinct species. There are three populations in the Halong Bay area with concave-conical shells bearing a single parietal tooth. Individuals from these populations are identified here as *A.fabella*. Conversely, the other populations of morphospecies that are conical, have a blunt apex, and possess a single parietal tooth are described as *A.babel* sp. nov.

**Figure 28.**
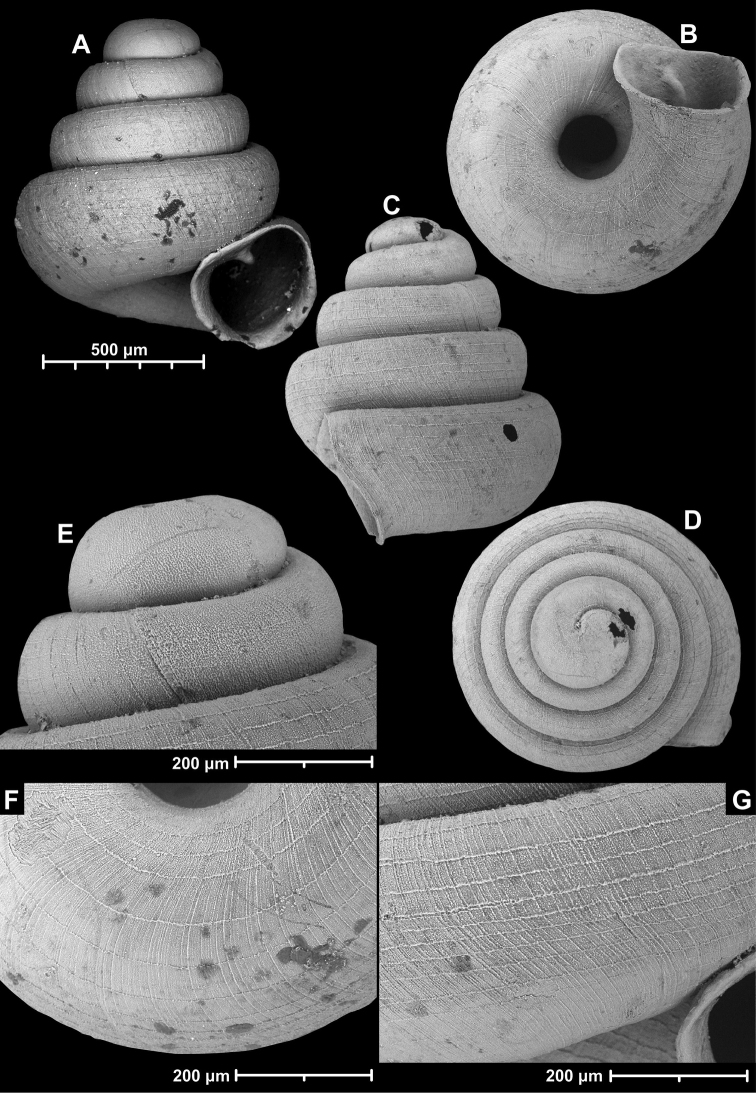
*Angustopilababel* Páll-Gergely & Vermeulen, sp. nov. (holotype, RMNH 347765). Apertural (**A**), ventral (**B**), lateral (**C**) and apical (**D**) sides of the shell; microstructure of the protoconch (**E**), ventral (**F**) and frontal (**G**) surface of the body whorl.

**Figure 29.**
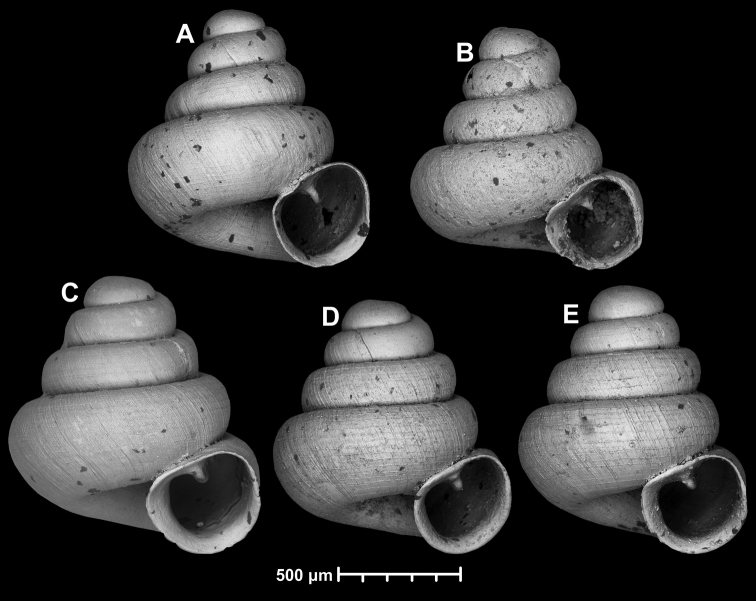
Variability of *Angustopilafabella* Páll-Gergely & Hunyadi, 2015 in the Halong Bay **A**JJV 6217 **B** WMVT.0338 and *Angustopilababel* Páll-Gergely & Vermeulen, sp. nov. **C**JJV 16590 **D**JJV 17634, Specimen 1 **E**JJV 17634, Specimen 2.

#### ﻿ Angustopila cavicola

Taxon classificationAnimaliaStylommatophoraGastrocoptidae

Páll-Gergely & Dumrongrojwattana sp. nov.

38F0069F-A0FE-542C-9F40-15B5339F2B44

https://zoobank.org/4EEC485D-638D-4136-95BF-340F508B04E8

Figs 30 , 32 , 33

##### Type material.

***Holotype***: Thailand • 1 empty shell (H: 1.18 mm, D: 0.92 mm); Loei Province, Phu Tham Pha Tang; approx. GPS coordinates: 17°56.91'N, 101°55.35'E; 29 Jun. 2016; P. Dumrongrojwattana, P. Juangsantad, K. Khwantong, N. Namisa & P. Panthong leg.; CUMZ 7441.

***Paratypes***: Thailand • 2 figured shells, same data as for holotype; HNHM 100181 • 8 shells; same data as for holotype; coll. PD • 8 shells; same data as for holotype; coll. PGB • 2 specimens in ethanol; same data as for holotype; coll. PGB.

##### Additional material.

Thailand • 11 j/b shells; same data as for holotype; coll. PD. Laos • 1 shell; Udomxai Province, 6.5 km southeast from centre of Na Mor towards Udomxai, Ban Nathong, Tham Nathong, below cave spring (locality code: 2019/118); 20°52.37'N, 101°46.98'E; 635 m a.s.l.; 7 Oct. 2019; A. Hunyadi leg.; coll. HA.

##### Diagnosis.

A medium-sized to large, conical *Angustopila* species with few whorls, a narrow umbilicus, a conspicuously large aperture, a strongly expanded peristome and a short but strong parietal tooth that points slightly towards the palatal wall.

##### Description (of the type sample).

Shell medium to large-sized for the genus, higher to much higher than wide; off-white, conical, apex broad, body whorl widest from apertural view; protoconch consists of 1.25 whorls, without spiral striation; teleoconch finely ornamented with irregular radial growth lines crossed by weak rows of irregularly spaced spiral threads (ca. 12–14 on body whorl from apertural view); sculpture weaker than in most other *Angustopila* species; whorls 3.5–4 (conspicuously few compared to large shell size), rounded; aperture slightly oblique to shell axis from lateral view; umbilicus very narrow; aperture subcircular, parietal callus convex; peristome strongly expanded especially at parietal and palatal region, not reflected; parietal callus overlapping and not smeared onto penultimate whorl; parietal tooth elevated, strong, normally long, slightly bent towards palatal side, does not reach parietal callus.

##### Measurements (in mm).

H = 1.02–1.18, D = 0.87–0.97, H/D*100 = 109.3–130.3 (shells from the type locality; *n* = 6), RUD = 17.6–19.8 (*n* = 3); H = 1.27, D = 0.99, H/D*100 = 128.3, RUD = 20.2 (2019/118; *n* = 1).

##### Differential diagnosis.

*Angustopilafabella* is smaller, has a more pointed apex, a comparatively smaller aperture, a weaker sculpture, and a less expanded peristome. All other conical species with a single tooth are much smaller than *A.cavicola* sp. nov.

##### Etymology.

This new species was collected inside a cave, in the dark zone (*cavicola* means cave-dweller in Latin).

##### Distribution.

The type series is known from Loei Province, Thailand. An additional, single shell, reminiscent of the typical *A.cavicola* sp. nov. shells, was collected in Northern Laos ca. 325 km north of the type series (Fig. 31).

**Figure 30.**
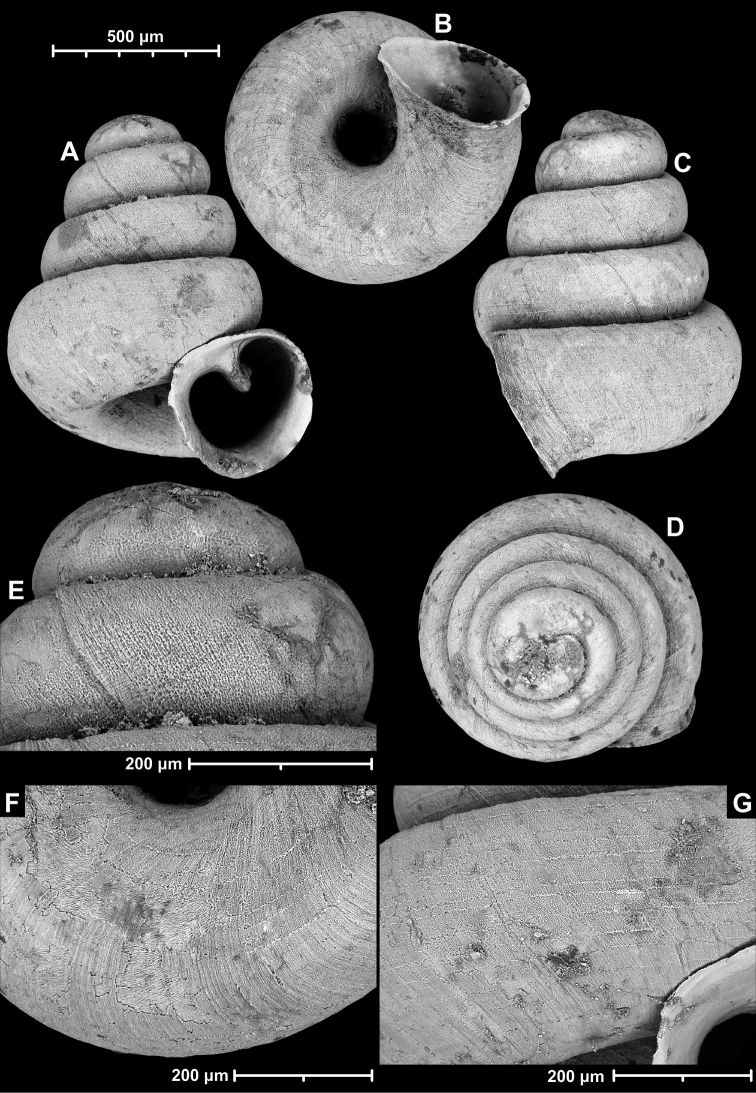
*Angustopilacavicola* Páll-Gergely & Dumrongrojwattana, sp. nov. (holotype, CUMZ 7441). Apertural (**A**), ventral (**B**), lateral (**C**) and apical (D) sides of the shell; microstructure of the protoconch showing protoconch-teleoconch boundary (**E**), eroded ventral (**F**) and frontal (**G**) surface of the body whorl.

**Figure 31.**
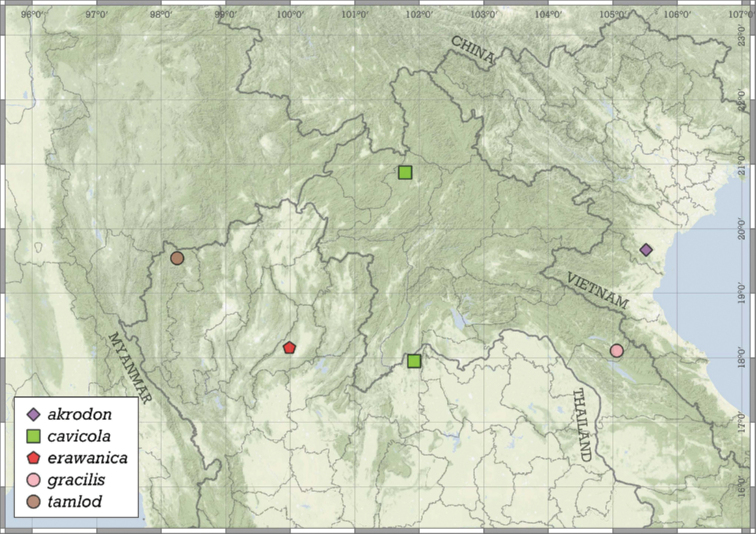
Distribution of *Angustopila* species.

**Figure 32.**
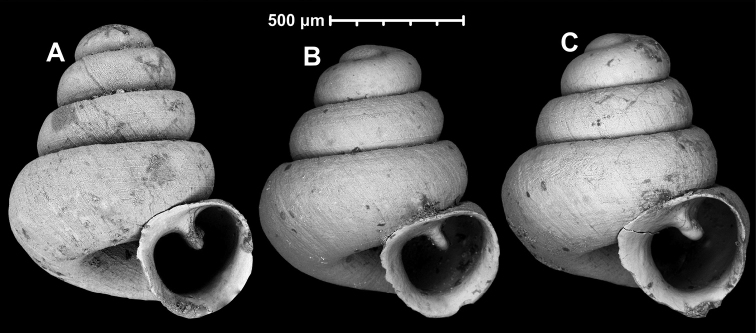
Variability of *Angustopilacavicola* Páll-Gergely & Dumrongrojwattana, sp. nov. **A** holotype (CUMZ 7441) **B, C** paratypes (HNHM 100181).

**Figure 33.**
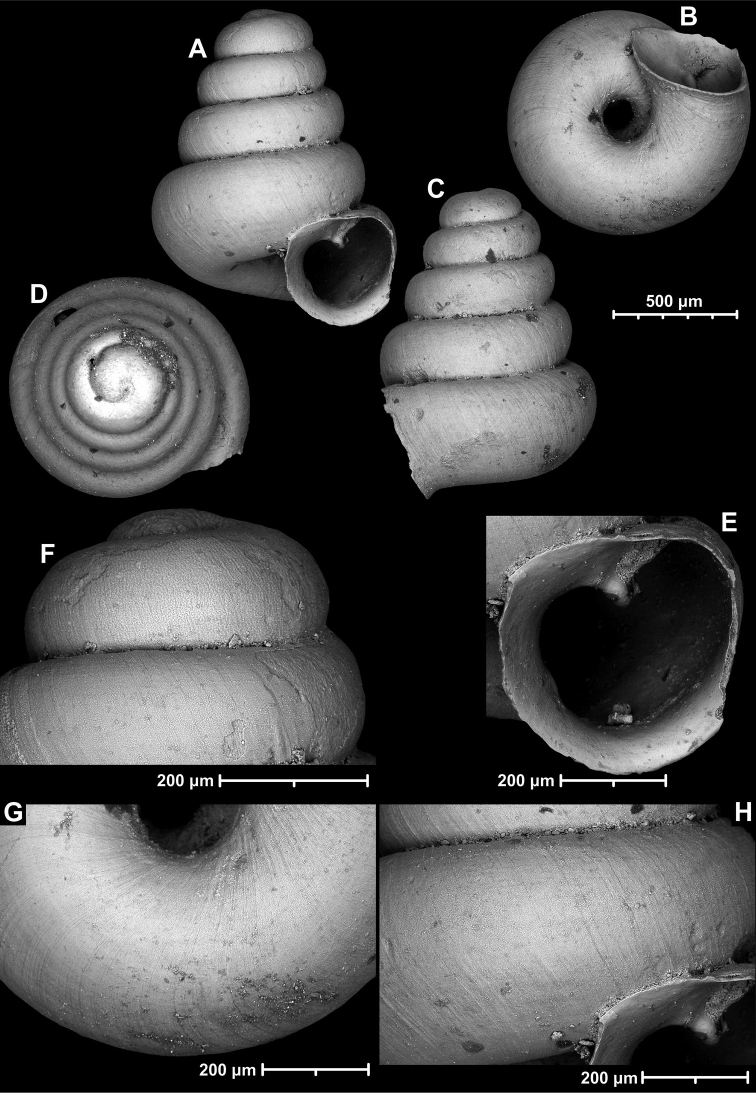
*Angustopilacavicola* Páll-Gergely & Dumrongrojwattana, sp. nov. from Laos (2019/118). Apertural (**A**), ventral (**B**), lateral (**C**) and apical (**D**) sides of the shell; aperture (**E**), microstructure of the protoconch (**F**), ventral (**G**) and frontal (**H**) surface of the body whorl.

##### Remarks.

The single shell from Laos has an even weaker sculpture (practically smooth) and a lower parietal tooth than the Thai ones. Otherwise, it is similar to the Thai population in terms of shell size, and shell and aperture shapes.

#### ﻿ Angustopila cicatricosa

Taxon classificationAnimaliaStylommatophoraGastrocoptidae

Páll-Gergely & Vermeulen sp. nov.

014EC9D2-E914-5CA7-BE46-31358A66B9AA

https://zoobank.org/94DF3D46-7607-4A10-9F28-A4B20D8E4618

Fig. 34

##### Type material.

***Holotype***: Vietnam • 1 empty shell (H: 0.68 mm, D: 0.68 mm); Haiphong Province, Cat Ba Island, near Nat. Park Headquarters, Trung Trang Cave, steep limestone cliff, woody regrowth at base; 20°47.78'N, 106°59.68'E; 25 Sep. 1998; J.J. Vermeulen & A.J. Whitten leg.; HNHM 103481 (original inventory number: JJV 6270).

##### Diagnosis.

A small *Angustopila* species with an ovoid shell, a rather strong, pointed parietal tooth, and very strong, elevated spiral striae.

##### Description.

Shell small for the genus, as high as wide, transparent, glossy, ovoid, dorsal side nearly domed, body whorl widest from standard apertural view; protoconch consists of 1.5 whorls, with very weak spiral striation preceding the first teleoconch whorl; teleoconch ornamented with irregular, very weak radial growth lines and strong, elevated rows of spiral threads (ca. 14 on body whorl from apertural view); whorls 4, rounded; aperture slightly oblique to shell axis from lateral view; umbilicus wide; aperture reniform; peristome edge slightly thickened and expanded, not reflected; parietal callus separated from penultimate whorl; aperture with a well-developed, deeply situated, pointed (short) parietal tooth.

##### Measurements (in mm).

H = 0.68, D = 0.68, H/D*100 = 100, RUD = 27.9 (holotype).

##### Differential diagnosis.

This species is characterised by the small size and the strong spiral striation. *Angustopilafraterminor* sp. nov. is larger with a higher spire, its shell surface is dull (glossy in *A.cicatricosa* sp. nov.), the aperture bears a longer parietal tooth (pointed in *A.cicatricosa* sp. nov.), and its peristome edge is less thickened, rather sharp, and slightly expanded. *Angustopilamaasseni* sp. nov. also has a duller shell surface with weaker spiral striae, and a lower spire. *Angustopilapsammion* has a lower spire, a wider umbilicus, and a smaller parietal tooth.

##### Etymology.

The specific epithet refers to the scar-like, strong spiral striae.

##### Distribution.

This species is only known from the type locality, Trung Trang Cave (Fig. 24).

##### Remarks.

At first, we considered this individual shell to belong to *A.fraterminor* sp. nov., to *A.maasseni* sp. nov., or to *A.psammion*. However, in addition to the differences regarding shell shape between *A.cicatricosa* sp. nov. and those two species, *A.cicatricosa* has an entirely different appearance (strongly striated, glossy) under the microscope. Given that single-site endemic species are not uncommon in the Halong Bay area, we consider this morphologically distinct shell a separate species.

**Figure 34.**
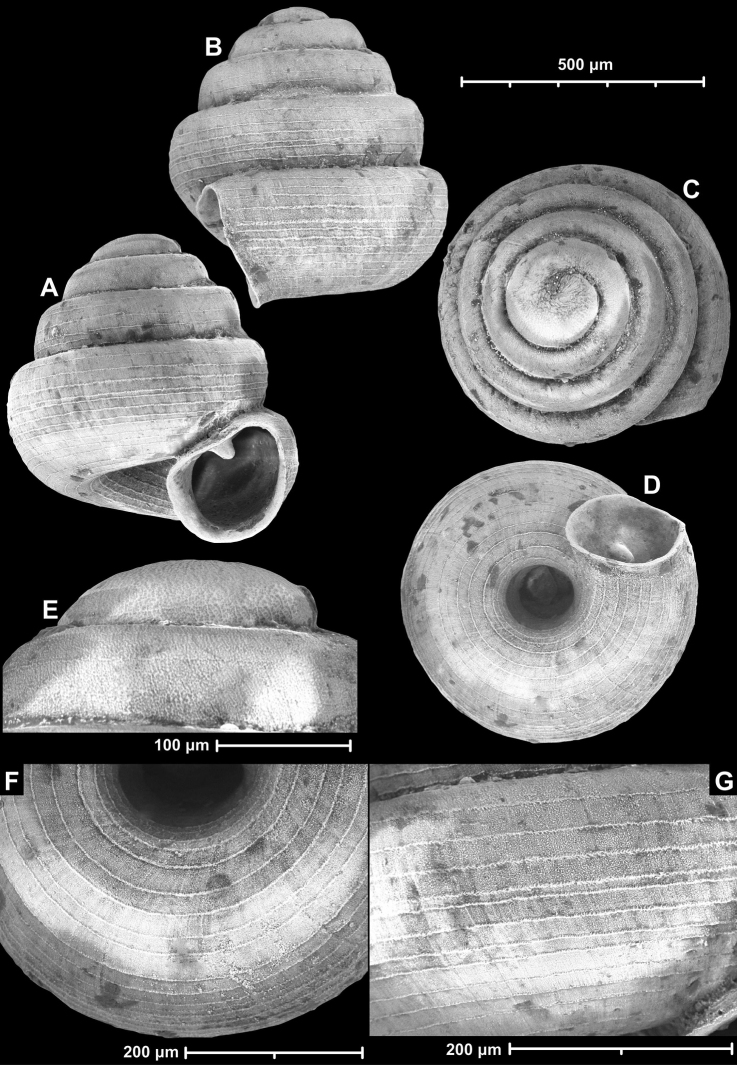
*Angustopilacicatricosa* Páll-Gergely & Vermeulen, sp. nov. (holotype, HNHM 103481). Apertural (**A**), lateral (**B**), apical (**C**) and ventral (**D**) sides of the shell; microstructure of the protoconch (**E**), microstructure on ventral (**F**) and frontal (**G**) surface of the body whorl.

#### ﻿ Angustopila concava

Taxon classificationAnimaliaStylommatophoraGastrocoptidae

(Thompson & Upatham, 1997)

E520A4F1-1F8B-5168-B2E8-D06CBED39A25

Figs 35 , 36

Systenostoma concava Thompson & Upatham, 1997: 231–232, figs 32–38.
Systenostoma concava — Panha and Burch 2005: 118–119, fig. 101.
Angustopila concava — Jochum et al. 2014: 27.

##### Type locality.

“Thailand, Nakhon Ratchasima Province, limestone hill 3.4 km west of Ban Mu Si, 380 m altitude (14°32.0'N, 101°22.5'E).”

##### Material examined.

***Holotype***: Thailand • 1 shell; Nakhon Ratchasima Province, limestone hill 3.4 km west of Ban Mu Si; 14°32.00'N, 101°22.50'E; 380 m a.s.l.; 5 May 1987; F.G. Thompson leg.; UF 113541 (not figured in the original description).

***Paratypes***: Thailand • 9 shells; same data as for holotype; UF 113542.

##### Diagnosis.

A large (H: 1.02–1.21 mm, D: 1.05–1.12 mm, H/D*100: 91.1–115.2 (*n* = 5), RUD = 28.3–32.7 (*n* = 3)), concave-conical *Angustopila* species with a very strongly oblique aperture, a low parietal tooth reaching peristome edge, a strongly concave parietal callus, and a conspicuously narrow sinulus.

##### Description.

Shell large for the genus, higher than wide, rarely slightly wider than high; concave-conical, body whorl widest in standard apertural view; protoconch consists of 1.25 whorls with weak spiral striae on the entire teleoconch; teleoconch dominated by an irregularly dimpled, pasty texture, with very fine irregularly spaced, weak radial growth lines crossed with very weak, obscure, sparsely-spaced spiral striae (impossible to count in standard apertural view, but on ventral side they are more widely-spaced than in other *Angustopila* species); spiral striae overall weaker than in most other *Angustopila* species; whorls ca. 4.5–4.75, rounded; aperture strongly oblique to shell axis from lateral view; umbilicus wide; aperture ovate-subquadrate, parietal callus concave; sinulus wide, weakly isolated due to the low parietal tooth; peristome expanded, especially on the palatal region, not reflected; parietal callus detached from penultimate whorl, protruding; parietal tooth very low, short, situated close to the parietal callus.

##### Differential diagnosis.

See under *Angustopilaerawanica* sp. nov., *A.occidentalis* sp. nov. and *A.uvula* sp. nov., and under Remarks.

##### Distribution.

This species is known only from the type locality in Thailand’s Nakhon Ratchasima Province (Fig. 19).

##### Remarks.

The original description mentions a slight tubercular thickening on the palatal lip. We have not found any thickening there that would be similar to the palatal teeth of any other *Angustopila* species.

*Angustopilaconcava* is similar to several populations of *A.fabella* in terms of shell size and the concave-conical shape, but differs from them in the strongly oblique aperture, the strongly sinuous parietal callus, and the conspicuously pointed (slender) sinulus. The parietal tooth of *A.concava* is weaker and situated closer to the parietal callus than that of the majority of *A.fabella* populations. The parietal tooth of only one *A.fabella* sample (15L06) is situated as close to the parietal callus as are those of *A.concava*. However, the other traits still differ between *A.fabella* sample 15L06 and *A.concava*.

**Figure 35.**
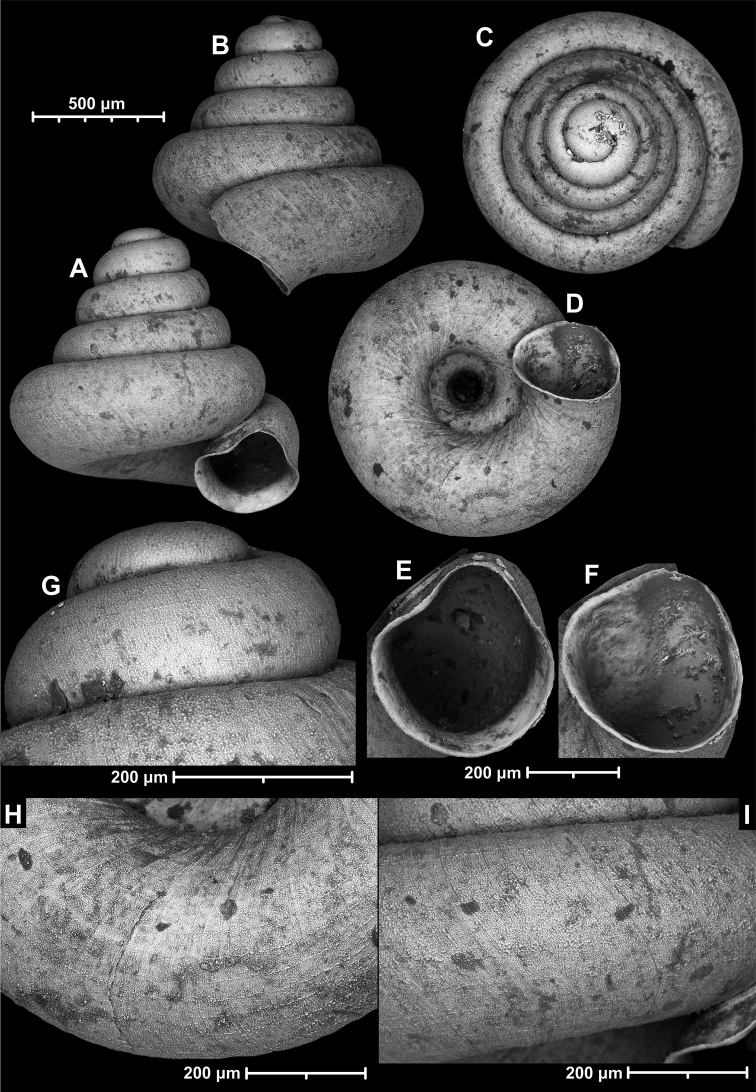
*Angustopilaconcava* (Thompson & Upatham, 1997), holotype (UF 113541). Apertural (**A**), lateral (**B**), apical (**C**) and ventral (**D**) sides of the shell; aperture (**E, F**), microstructure on the protoconch (**G**), microstructure on ventral (**H**) and frontal (**I**) surface of the body whorl.

**Figure 36.**
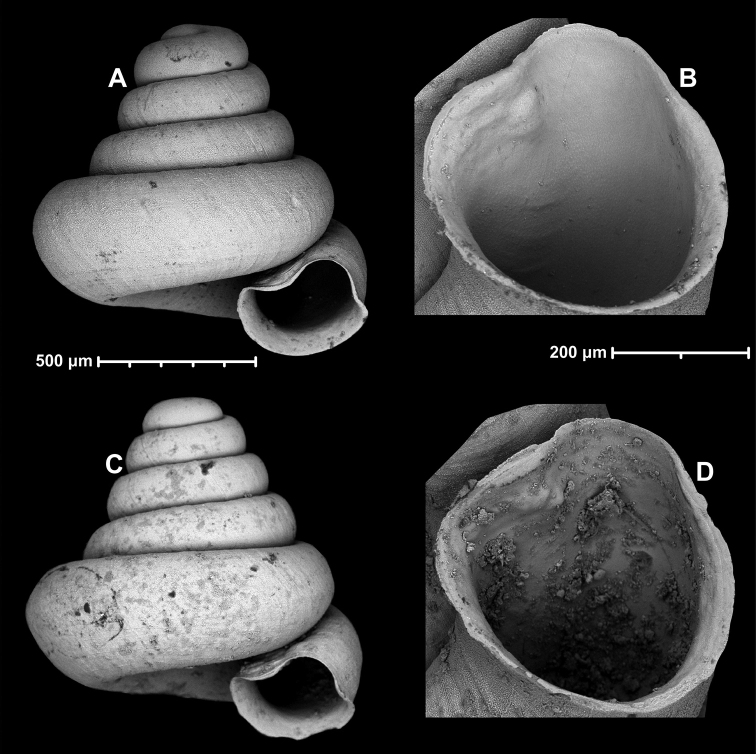
*Angustopilaconcava* (Thompson & Upatham, 1997). Paratypes (UF 113542, carbon-coated shells, probably imaged in the original description) **A, B** paratype, specimen 1 **C, D** paratype, specimen 2.

#### ﻿ Angustopila erawanica

Taxon classificationAnimaliaStylommatophoraGastrocoptidae

Páll-Gergely & Dumrongrojwattana sp. nov.

2FDE4A26-983B-5238-8379-B1950E652C7A

https://zoobank.org/0C2132C3-73C5-4330-8ADE-CBF861481089

Figs 37 , 38 , 39

##### Type material.

***Holotype***: Thailand • 1 empty shell (H: 1.11 mm, D: 0.97 mm); Phrae Province, Long District, Tham Erawan (cave) near Maharat Rock Garden; 18°09.21'N, 99°59.24'E; 18 Jun. 2016; P. Dumrongrojwattana, P. Juangsantad, K. Khwantong, N. Namisa & P. Panthong leg.; CUMZ 7437.

***Paratypes***: Thailand • 2 figured shells; same data as for holotype; HNHM 103474 • 2 dissected paratypes in ethanol; same data as for holotype; HNHM 103472 • 2 shells; same data as for holotype; NMBE 551275 • 7 specimens in ethanol; same data as for holotype; coll. PGB • 8 specimens in ethanol; same data as for holotype; coll. PD • 10 shells; same data as for holotype; coll. PD • 2 shells; same data as for holotype; coll. HA • 34 shells; same data as for holotype; coll. PGB.

##### Additional material.

Thailand • 18 j/b shells; same data as for holotype; coll. PD.

##### Diagnosis.

A medium-sized to large *Angustopila* species with a narrow umbilicus and an elliptical or ovoid, slightly protruding aperture with wide sinulus and impressed at the position of the strong parietal tooth.

##### Description.

Shell medium- to large-sized for the genus, slightly higher than wide or slightly wider than high; pale grey, conical-ovoid, last or penultimate whorl widest from standard apertural view; protoconch consists of 1.25 whorls, with very slight indication of spiral striae preceding the first teleoconch whorl; teleoconch sculpture overall weak; teleoconch finely ornamented with irregularly spaced radial growth lines crossed by fine rows of regularly spaced spiral striae (ca. 16–18 spiral striae on body whorl in apertural view, the denser striation on the holotype is probably due to duplication of spiral striation, other shells have fewer striae); on both frontal and ventral surfaces of body whorl spiral lines dominant; whorls 4.5, slightly shouldered; aperture strongly oblique to shell axis from lateral view; umbilicus very narrow, less than 1/4 of shell width; aperture slightly protruding (visible from ventral and lateral views); aperture elliptical or ovoid, impressed at position of parietal tooth, sinulus wide; peristome slightly expanded, not reflected; parietal callus strongly elevated, sharp, detached from penultimate whorl; parietal tooth strongly developed; parietal side and parietal tooth align at an angle less than 90 degrees.

##### Measurements (in mm).

H = 0.94–1.11; D = 0.97–1.05, H/D*100 = 95.9–114.4 (*n* = 4), RUD = 20.4–22.4 (*n* = 3).

##### Differential diagnosis.

The aperture shape, in combination with the large size and narrow umbilicus, distinguishes this species from its congeners. *Angustopilaerawanica* sp. nov. is most similar to *A.bidentata* sp. nov. by its narrow umbilical form and the shape of the aperture, but that species has a strong palatal tooth, a less protruding and more oblique aperture, and stronger spiral striation on the protoconch. *Angustopiladominikae* also possesses a strong palatal tooth and has a more globular shell. *Angustopilaconcava* has a wider body whorl, a wider umbilicus, and a more oblique aperture. See also under *A.huoyani*.

##### Anatomy.

Two specimens were dissected. Due to the small size of the animals, some traits could not be examined in detail. The traits of the distal genitalia could be relatively clearly seen, but proximal to the vagina, the animals consisted of a very soft, gelatinous material. No bursa copulatrix or any other female glands found, but this can be due to the difficulties to separate these organs. Penis well-developed but short, with triangular, thick penial caecum; retractor muscle inserts at end of caecum; epiphallus starts from base of caecum, thickened at its proximal end; vas deferens slender, but could not be followed (got torn just after its distal end); the lumen of the penis is visible under light microscope as a whiter (= less translucent) area, this can be followed from the distal penis throughout the penial caecum to the proximal end of epiphallus; vagina thick, longer than penis, although its proximal end could not be identified, inside vagina was a thick whiter area.

**Figure 37.**
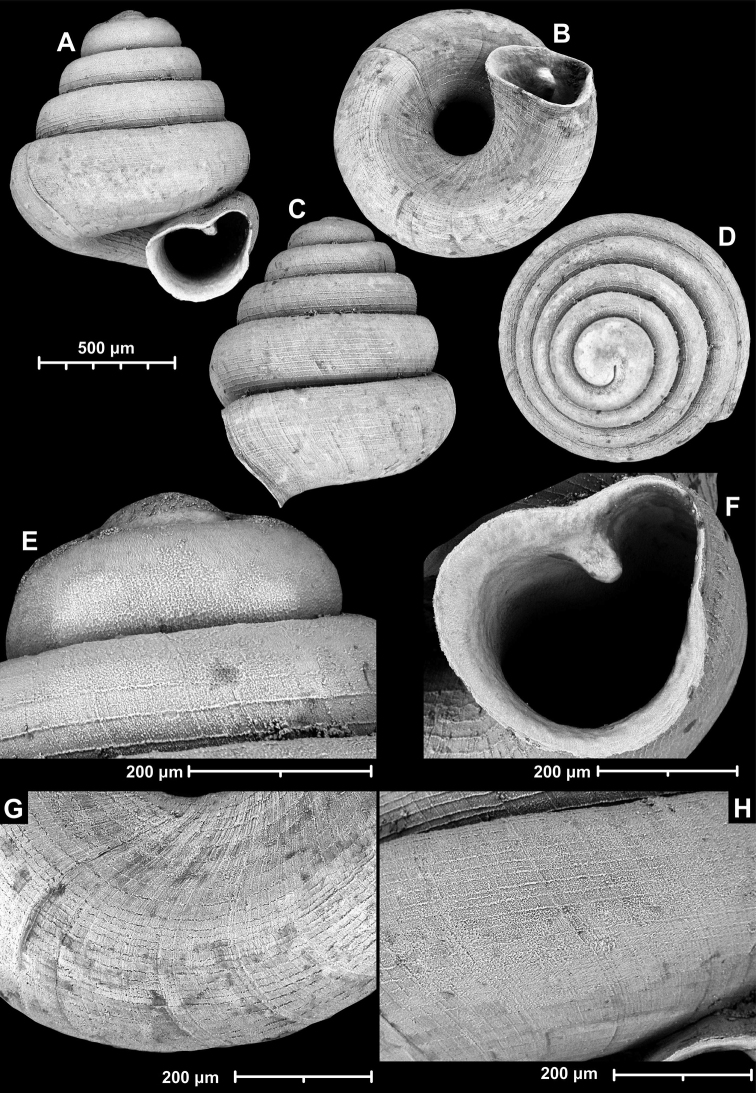
*Angustopilaerawanica* Páll-Gergely & Dumrongrojwattana, sp. nov. (holotype, CUMZ 7437). Apertural (**A**), ventral (**B**), lateral (**C**) and apical (**D**) sides of the shell; aperture (**F**); sculpture of the protoconch (**E**), ventral (**G**) and frontal (**H**) surface of the body whorl.

**Figure 38.**
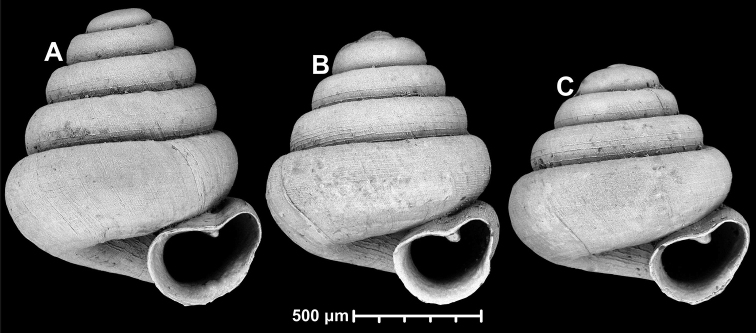
Conchological variability of *Angustopilaerawanica* Páll-Gergely & Dumrongrojwattana, sp. nov. **A** and **C** paratypes [HNHM 103474] **B** holotype [CUMZ 7437].

**Figure 39.**
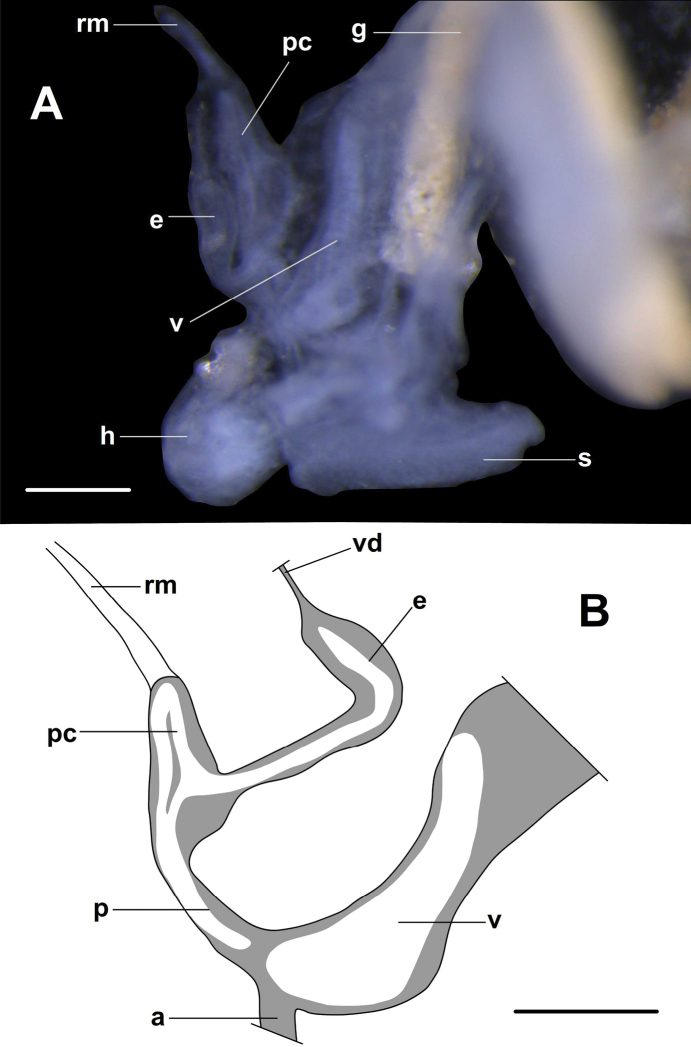
Anatomy of *Angustopilaerawanica* Páll-Gergely & Dumrongrojwattana, sp. nov. (HNHM 103472) **A** photo of the left lateral side of the body for which the genitalia are dissected out and specified in B **B** distal genitalia. Abbreviations: a: atrium; e: epiphallus; g: gut; h: head; p: penis; pc: penial caecum; rm: retractor muscle; s: sole; v: vagina; vd: vas deferens. White areas in **B** indicate parts of the genitalia that were lighter in colour (i.e., less translucent). Scale bars: 200 μm.

##### Etymology.

The new species is named after its type locality (Erawan cave). Specific epithet is used as a noun in apposition. Erawan is the Thai form of the mythical three- or thirty-three-headed, white elephant ridden by Indra (Sanskrit), the diety of rain, thunder, weather, and river flows.

##### Distribution.

This species is known from the type locality only (Fig. 31).

#### ﻿ Angustopila fabella

Taxon classificationAnimaliaStylommatophoraGastrocoptidae

Páll-Gergely & Hunyadi, 2015

5D31E405-49B4-5B58-83BB-18B81977264E

Figs 29A, B , 40 , 41 Suppl. material 3: figs S9–S29

Angustopila fabella Páll-Gergely et al., 2015a: 36, fig. 2.
Angustopila singuladentis Inkhavilay & Panha in Inkhavilay et al. 2016: 224, fig. 5. new synonym.
Angustopila singuladentis — Inkhavilay et al. 2019: 59, fig. 26A.

##### Type locality.

“China, Guangxi (广西), Chongzuo Shi (崇左市), Longzhou Xian (龙州县), cliffs north of Lenglei (楞垒), north of the Nonggang Nature Reserve (弄岗国家级自然保护区), 220 m, 22°29.161'N, 106°57.357'E” (*A.fabella*); “Xang Lod Cave, Viengxay District, Houaphane Province, Laos (20°24'31.3"N, 104°13'19.7"E), 882 m a.s.l.” (*A.singuladentis*).

##### Type material examined.

Holotype of *A.singuladentis*, CUMZ 7063 (reimaged for this study Fig. 41); paratypes, CUMZ 7064.

##### Additional material examined.

**Wide umbilicus** Vietnam • 9 adult + 2 j/b shells; Sơn La Province, Yên Châu District, Xã Chiềng On, Bản Trạm Hốc, Hang Nhả Nhung, around the cave (locality code: 2020/20); 20°59.48'N, 104°11.27'E; 970 m a.s.l.; 9 Feb. 2020; A. Hunyadi leg.; coll. HA • 1 shell; Sơn La Province, 27 km ESE from centre of Phù Yên, Mường Do, Bàn Han Mȏt, 300 m south of the village (locality code: 2020/7); 21°11.73'N, 104°47.13'E; 810 m a.s.l.; 6 Feb. 2020; A. Hunyadi leg.; coll. HA • 7 adult + 3 j/b shells; Sơn La Province, 10 km from centre of Mộc Châu towards Sơn La, right side of road no. 6, south of Tất Ngoằng (locality code: 2020/5); 20°52.58'N, 104°35.34'E; 715 m a.s.l.; 5 Feb. 2020; A. Hunyadi leg.; coll. HA • 5 shells; Sơn La Province, Mộc Châu District, Vân Hồ, northwestern edge of Pa Cốp towards Bó Nhàng (locality code: 2020/24); 20°46.00'N, 104°45.20'E; 980 m a.s.l.; 10 Feb. 2020; A. Hunyadi leg.; coll. HA • 3 adult shells; Hòa Bình Province, Tân Lạc District, 1300 m west from Quy Hậu on road no. 6, rock wall (locality code: 2020/28); 20°37.93'N, 105°16.17'E; 150 m a.s.l.; 11 Feb. 2020; A. Hunyadi leg.; coll. HA.

**Specimens with wide umbilicus and narrow umbilicus with transitional forms** Vietnam • 318 shells; Sơn La Province, Quỳnh Nhai district, 20 km north from cross to Thuận Châu, Chiềng Khoang, cave above the village (locality code: 2020/9); 21°33.44'N, 103°40.91'E; 315 m a.s.l.; 7 Feb. 2020; A. Hunyadi leg.; coll. HA.

“**Regular form**” Vietnam • 64 adult + 20 j/b shells; Thanh Hóa Province, Quan Hóa District, Khu di tích Hang Ma, gorge of Sȏng Luồng (locality code: 2020/30); 20°23.89'N, 105°04.02'E; 100 m a.s.l.; 12 Feb. 2020; A. Hunyadi leg.; coll. HA • 2 shells; Lạng Sơn Province, vicinity of Chùa Tam Thanh (locality code: 2020/58); 21°51.35'N, 106°44.81'E; 265 m a.s.l.; 21 Feb. 2020; A. Hunyadi leg.; coll. HA • 6 shells; Lạng Sơn Province, Hữu Lũng District, Hữu Liên, cross of roads no. 1B-241, 33.5 km towards Ba Nàng, left side of road (locality code: 2020/52); 21°41.06'N, 106°22.87'E; 220 m a.s.l.; 19 Feb. 2020; A. Hunyadi leg.; coll. HA • 2 shells; Lạng Sơn Province, Hữu Lũng District, Hữu Liên, 1400 m west from Đȏng Lâm along road no. 241 (locality code: 2020/53); 21°41.91'N, 106°21.77'E; 210 m a.s.l.; 19 Feb. 2020; A. Hunyadi leg.; coll. HA • 18 shells; Ninh Binh Province, Cuc Phuong National Park, Prehistorical Man Cave, steep limestone slope with disturbed forest; approx. GPS data: 20°21'N, 105°54'E; 10 Oct. 1998; J.J. Vermeulen & L. Deharveng leg.; JJV 6223 • 134 shells; Thanh Hóa Province, Lang Chánh District, Đồng Lương, 9.4 km northeast from centre of Lang Chánh towards Làng Thung (locality code: 2020/32); 20°11.52'N, 105°15.59'E; 300 m a.s.l.; 12 Feb. 2020; A. Hunyadi leg.; coll. HA • 1 adult + 2 broken shells; Thanh Hóa Province, 9.8 km from centre of Ngọc Lặc towards Lang Chánh, Ngọc Khê, left side of road (locality code: 2020/34); 20°07.19'N, 105°18.28'E; 115 m a.s.l.; 13 Feb. 2020; A. Hunyadi leg.; coll. HA • 3 shells; Thanh Hóa Province, Như Thanh District, Hải Vân, Hang Lò Cao Kháng Chiến, vicinity of the cave (locality code: 2020/41); 19°37.08'N, 105°34.63'E; 20 m a.s.l.; 14 Feb. 2020; A. Hunyadi leg.; coll. HA • 1 shell; Sơn La Province, Mộc Châu, Hang Dơi, around the entrance of the cave (locality code: 2020/26); 20°50.96'N, 104°38.34'E; 865 m a.s.l.; 11 Feb. 2020; A. Hunyadi leg.; coll. HA.

Laos • 1 shell; Luang Namtha Province, 19.8 km southeast from centre of Vieng Phou Kha, south from Ban Phou Lek, southern edge of quarry (locality code: 2019/122); 20°32.54'N, 101°07.96'E; 780 m a.s.l.; 8 Oct. 2019; A. Hunyadi leg.; coll. HA • 1 shell; Luang Namtha Province, 48 km southwest from centre of Luang Namtha towards Vieng Phou Kha, Nam Eng, Tham Kao Rao (locality code: 2019/120); 20°43.45'N, 101°09.26'E; 745 m a.s.l.; 7 Oct. 2019; A. Hunyadi leg.; coll. HA • 79 small shells + 7 larger shells + 7 j/b shells (see Suppl. material 3: figs S9–S12); Udomxai Province, 6.5 km southeast from centre of Na Mor towards Udomxai, Ban Nathong, Tham Nathong, below cave spring (locality code: 2019/118); 20°52.37'N, 101°46.98'E; 635 m a.s.l.; 7 Oct. 2019; A. Hunyadi leg.; coll. HA • 21 shells; Udomxai Province, 10 km south of centre of Na Mor, 3.8 km east-southeast of Na Xay, rock wall facing north (locality code: 2019/119); 20°53.37'N, 101°48.96'E; 660 m a.s.l.; 7 Oct. 2019; A. Hunyadi leg.; coll. HA • 1 shell (figured); Luang Prabang province, just NE of Phou Khoun, under rocks in old secondary forest above large cave (locality code: 32L06); 19°26.78'N, 102°26.29'E; 1180 m a.s.l.; 15 Nov. 2006; A. Abdou & I.V. Muratov leg.; MNHN-IM-2014-6413 • 55 concave-conical + 76 low concave-conical + 33 conical + 73 j/b shells; Luang Prabang Province, 2.8 km northeast from Phou Khoun, rock wall above the cave (locality code: 2019/128); 19°26.79'N, 102°26.34'E; 1200 m a.s.l.; 11 Oct. 2019; A. Hunyadi leg.; coll. HA • 6 shells; Vientiane Province, 8.5 km north from centre of Kasi, northwest of the quarry (locality code: 2019/129); 19°16.82'N, 102°13.89'E; 540 m a.s.l.; 12 Oct. 2019; A. Hunyadi leg.; coll. HA • 1 figured shell; Vientiane Province, Tham Pou Kham (cave), W Vang Vieng, at the base of rocks (locality code: La.11); 18°55.60'N, 102°23.71'E; 280 m a.s.l.; Mar. 2010; A. Reischütz leg.; NHMW-MO-112015 • 2 shells; same data as for preceding; NMBE 550645 • 2 shells; same data as for preceding; coll. PGB • 17 shells; same data as for preceding; coll. RE • 57 + 11 j/b shells; Vientiane Province, 7.5 km west from centre of Vang Vieng, Ban Naka, Tham Poukham (locality code: 2019/131); 18°55.61'N, 102°23.77'E; 240 m a.s.l.; 14 Oct. 2019; A. Hunyadi leg.; coll. HA • 1 shell; Bolikhamsay Province, cave entrance above karst spring 500 m N of road from Vieng Thong to Ban Samsok (and Sôp Sang), 16 km from Vieng Thong, 500 m N of the road around large cave entrance above karst spring with travertine cascades (locality code: JG20A); 18°33.06'N, 104°34.19'E; 19 Feb. 2017; J. Grego leg.; coll. JG • 2 shells; Bolikhamsay Province, 1 km SE of Vieng Thong towards Houaykhay on road 1D, caves among boulders at SW bases of rocky cliffs (locality code: JG22); 18°30.28'N, 104°27.47'E; 290 m a.s.l.; 20 Feb. 2017; J. Grego leg.; coll. JG • 11 adult + 2 j/b shells; Bolikhamsay Province, large spring lake at S base of limestone massif 2 km NNE of Na Pavan village, ca. 5 km from Phontan crossing at road 8 (locality code: JG16); 18°13.30'N, 104°44.81'E; 18 Feb. 2017; J. Grego leg.; coll. JG • 1 shell; same data as for preceding; NMBE 550644 • 1 shell; Bolikhamsay Province, NE base of Pha Hông limestone massif 7 km N of Lak Sao (locality code: JG12); 18°13.91'N, 104°56.41'E; 16 Feb. 2017; J. Grego leg.; coll. JG • 1 figured shell; same data as for preceding; NHMUK 20170304.

**Halong Bay area** Vietnam • 18 shells; Quang Ninh Province, Halong-Campha area, 4.5 km SW Quang Hanh, limestone hill with regrowth; 20°58.98'N, 107°11.83'E; 29 Sep. 1998; J.J. Vermeulen & A.J. Whitten leg.; JJV 6217 • 1 shell; Haiphong Province, Halong Bay area, unnamed island off E Coast Cat Ba, south facing bay with beach and densely-vegetated limestone scree slope; 20°45.19'N, 107°04.46'E; 1 Oct. 1998; J.J. Vermeulen & A.J. Whitten leg.; JJV 6219 • 1 shell; Quang Ninh Province, Halong Bay area, Tien Ong Cave on Hang Trai Island (locality code: WMVT.0338); 20°48.96'N, 107°07.33'E; 6 Sep. 2003; W.J.M. Maassen leg.; RMNH 347767.

**Large shell with anteriorly situated parietal tooth** Laos • 1 shell (figured); Luang Prabang province, ca. 9 km SE of Luang Prabang, on the left side of river Nam Khan, limestone, black soil in limestone pockets, clay, under rocks and logs in secondary forest under cliff (locality code: 15L06); 19°51.38'N, 102°12.46'E; 365 m a.s.l.; 3 Nov. 2006; A. Abdou & I.V. Muratov leg.; MNHN-IM-2014-6414.

**Tiny shell with large variability** Vietnam • 4 shells (H: 0.72–0.83 mm, D: 0.70–0.82 mm); Xieng Khouang Province, foot of limestone ridge 3 km NW of Thathom, 800 m S of road 1D crossing a small river (locality code: JG26); 19°00.33'N, 103°34.90'E; 22 Feb. 2017; J. Grego leg.; coll. JG.

##### Diagnosis.

A small to large, mostly conical or concave-conical (rarely conical-globular) species with a single parietal tooth, shell mostly higher than wide, rarely slightly wider than high. Extremely variable in shell shape across and even within populations.

##### Measurements (in mm).

H = 0.72–1.16, D = 0.70–1.12, H/D*100 = 87.3–119.8 (*n* = 99), RUD = 21.4–32.3 (*n* = 34); H = 1.12, D = 1.19 H/D*100 = 94.1, RUD = 31.9 (15L06).

##### Differential diagnosis.

Species with conical and concave-conical shells and a single parietal tooth are the following: *Angustopilaconcava*, *A.babel* sp. nov., *A.oostoma* sp. nov., *A.margaritarion* sp. nov., *A.cavicola* sp. nov., *A.szekeresi*, and *A.vandevenderi* sp. nov. Among them, *A.margaritarion* sp. nov., *A.cavicola* sp. nov., *A.szekeresi* are sympatric with *A.fabella*, while *A.babel* sp. nov., *A.oostoma* sp. nov., and *A.vandevenderi* sp. nov. occur in parapatry with *A.fabella* (i.e., populations of one of those three species occur geographically close to an *A.fabella* population). For comparisons, see under those species. See also under *A.cicatricosa* sp. nov.

##### Distribution.

Known from several localities across northern Laos, northern Vietnam and from one site in Guangxi, China, close to the Vietnamese border (Fig. 42).

**Figure 40.**
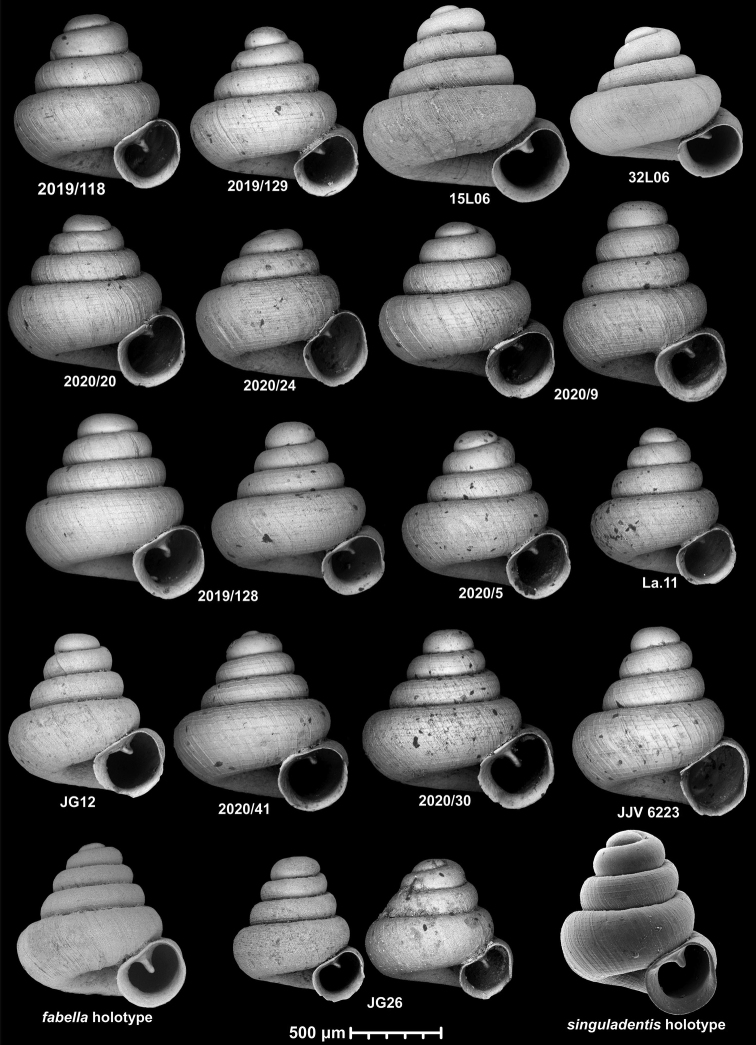
Synoptic view of *Angustopilafabella* Páll-Gergely & Hunyadi, 2015.

**Figure 41.**
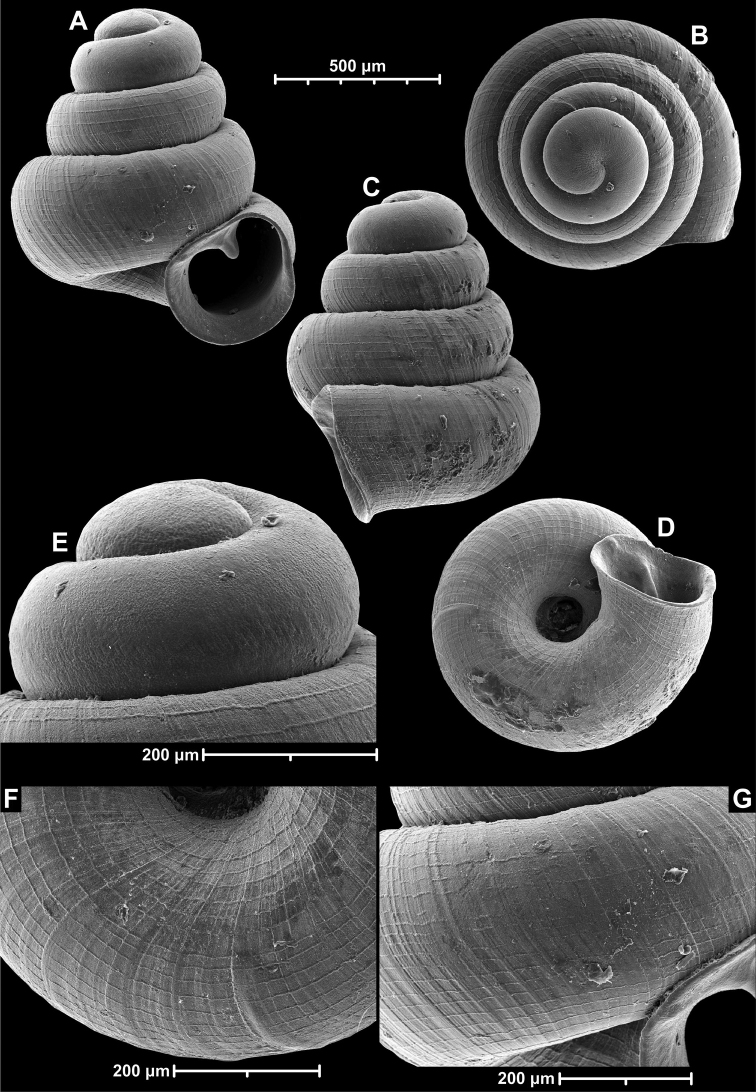
*Angustopilafabella* Páll-Gergely & Hunyadi, 2015 (holotype of *Angustopilasinguladentis* Inkhavilay & Panha, 2016). Apertural (**A**), apical (**B**), lateral (**C**) and ventral (**D**) sides of the shell; microstructure of the protoconch (**E**), ventral (**F**) and frontal (**G**) surface of the body whorl. SEM Images: Chirasak Sutcharit.

**Figure 42.**
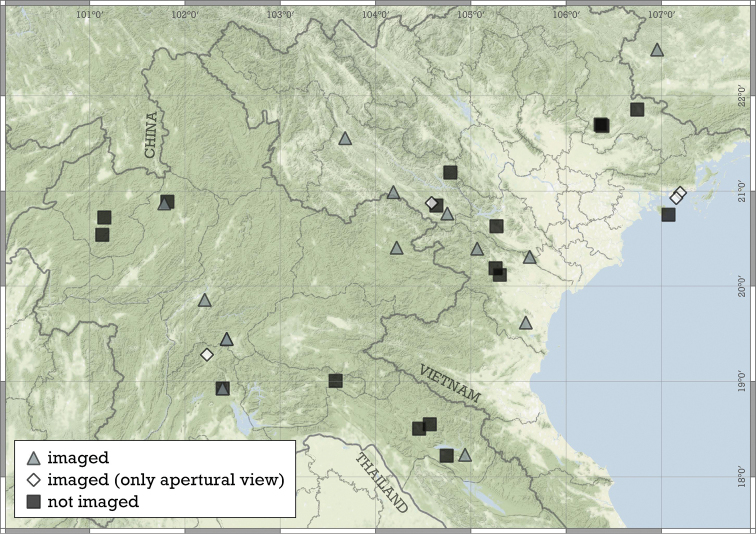
Distribution of *Angustopilafabella* Páll-Gergely & Hunyadi, 2015.

##### Remarks.

This is a variable species that is characterised by a conical or concave-conical (in some populations nearly domed) shell and a single parietal tooth. The key traits of the examined populations are compiled in Table 2. Among the three widely distributed species, the morphological variability of *A.fabella* is considerably greater than that of the other two species (*A.szekeresi* and *A.elevata*). This suggests that the samples listed under *A.fabella* may belong to several distinct species, and species delimitation can only be revealed by molecular phylogenetic means. The following populations should be noted:

**Table 2.** Main morphological differences of *A.fabella* populations.

	Shell shape	Parietal tooth	Umbilicus	Other distinguishing character	Image
*A.fabella* type	concave-conical	strongly elevated	normal		Fig. 40
2020/58	conical	normal	normal	comparatively large aperture	no
2020/52	conical	normal	normal		no
2020/53	conical	normal	normal		no
JJV 6217	concave-conical	normal	wide		no
WMVT.0338	concave-conical	normal	wide		no
JJV 6219	concave-conical	normal	wide		no
2020/09	low conical to conical	normal	normal to wide	very variable sample	Suppl. material 3: figs S20, S21
2020/20	low conical	normal	wide		Suppl. material 3: fig. S18
2020/7	concave-conical (low)	normal	wide		no
2020/5	conical to concave-conical (low)	normal	normal to wide		Fig. 40
2020/26	conical	normal	normal	strong spiral striation	Fig. 40
2020/24	low conical	normal	wide	dense spiral striation	Suppl. material 3: fig. S19
2020/28	low conical	normal	wide		Fig. 40
2020/30	concave-conical (low)	strongly elevated	wide		Suppl. material 3: fig. S23
2020/32	conical	normal to elevated	normal		Fig. 40
2020/34	concave-conical	normal	normal		no
JJV 6223	concave-conical	normal	normal to wide	strong spiral striation	Suppl. material 3: fig. S26
2020/41	concave-conical	normal	wide	comparatively small aperture	Suppl. material 3: fig. S24
2019/120	conical	strongly elevated	normal		no
2019/122	conical	strongly elevated	normal		Fig. 40
2019/119	conical	strongly elevated	wide		Fig. 40
2019/118	conical	normal to elevated	normal to wide	variable sample, 2 distinct size categories present	Suppl. material 3: figs S9–S12
15L06	concave-conical	normal	wide	shell very large, parietal tooth situated close to callus	Suppl. material 3: fig. S17
32L06, 2019/128	conical to concave-conical (low)	normal	normal to wide	very variable sample, more or less distinct morphological groups found	Suppl. material 3: figs S13–S16, S22
2019/129	concave-conical	normal	wide		Fig. 40
LA.11, 2019/131	concave-conical	very low	normal		Suppl. material 3: fig. S27
JG22	concave-conical	normal	wide	the two shells differ considerably from each other, more material is needed	no
JG20a	concave-conical	normal	normal		yes
JG16	concave-conical	strongly elevated	wide	very similar to typical *A.fabella*	no
JG26	conical	normal	normal	very small shell	Suppl. material 3: fig. S28–29S
JG12	concave-conical	normal	normal		Suppl. material 3: fig. S25

1. The sample 2019/118 contained two ‘morphotypes’, which differed only in shell size. By measuring each shell, it was revealed that the two ‘morphotype’ groups form non-overlapping clusters on the shell diameter vs. shell height diagram. One explanation is that they belong to two morphologically similar species differing only in size. The other explanation is that this is a single species and at the geographically rather broad (ca. 100 meters) collection site, individuals inhabited different microhabitats, which resulted in differences in shell size. In the absence of other conchological characters, we accept the latter hypothesis.
2. Sample 2019/128 contained three more or less clear ‘morphotypes’: strongly concave-conical with pointed apex and wide umbilicus; concave-conical with blunt apex and wide umbilicus; and conical with broad apex and narrower umbilicus. The allocation of some shells into either group was problematic, indicating that this is a very variable single species.
3. The single shell from site 15L06 is much larger than that seen in any of the other *A.fabella* shells whereby the parietal tooth is situated much more anteriorly, so that it reaches the edge of the parietal callus. This shell may well belong to a distinct species. However, given the large morphological variability of *A.fabella*, the absence of samples from the close vicinity, and the fact that we have only one shell from sample 15L06, we refrain from describing it as new. *Angustopilaconcava* possesses a similar, anteriorly positioned (albeit lower) parietal tooth, and a similar large, concave-conical shell, but it has a more oblique aperture.
4. All *A.fabella* samples from Sơn La and Hòa Bình Provinces have conical-globular shells with a wide umbilicus except for one sample, which has a conical shell and narrow umbilicus. However, shells from site 2020/9 (Sơn La Province) are extremely variable and there is a continuous variation from conical-globular shells with a wide umbilicus to conical shells with a narrow umbilicus. Therefore, we consider the conical-globular-type shell a local variation of *A.fabella*, which nonetheless, warrants further attention.
5. Sample JG26 from Xieng Khouang Province, Laos, contained four shells showing remarkable diversity in terms of shell shape. Moreover, these are the smallest *A.fabella* shells amongst all examined populations.

#### ﻿ Angustopila fratermajor

Taxon classificationAnimaliaStylommatophoraGastrocoptidae

Páll-Gergely & Vermeulen sp. nov.

23B68695-676C-52EE-A324-995D0CAAD907

https://zoobank.org/12980F6D-6003-44A5-9734-A81BC10C2CA6

**Figure 43.**
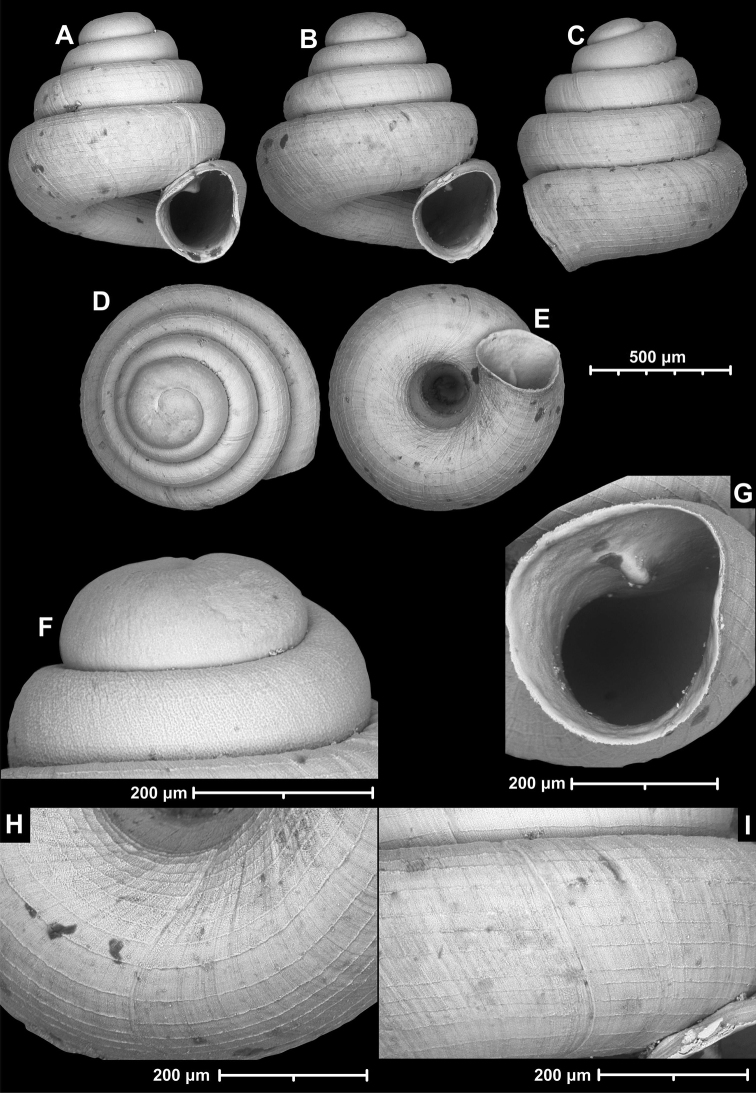
*Angustopilafratermajor* Páll-Gergely & Vermeulen, sp. nov., paratype (**A**) and holotype (HNHM 105286), JJV 17605 (**B–I**). Apertural (**A, B**), lateral (**C**), apical (**D**) and ventral (**E**) sides of the shell; aperture (**G**), microstructure of the protoconch (**F**), ventral (**H**) and frontal (**I**) surface of the body whorl.

Fig. 43 , Suppl. material 3: fig. S30

##### Type material.

***Holotype***: Vietnam • 1 empty shell (H: 0.84 mm, D: 0.8 mm); Haiphong Province, Cat Ba Island, large, somewhat disturbed, active cave with speleothems; 20°50.06'N, 106°55.91'E; 0–50 m a.s.l.; 7 Jun. 2017; J.J. Vermeulen & K. Anker leg.; HNHM 105286 (original inventory number: JJV 16605).

***Paratypes***: Vietnam • 68 shells; same data as for holotype; JJV 17658 (ex JJV 17605) • 10 shells; Quang Ninh Province, Halong-Campha area, 4.5 km SW Quang Hanh, limestone hill with regrowth; 20°58.98'N, 107°11.83'E; 29 Sep. 1998; J.J. Vermeulen & A.J. Whitten leg.; JJV 6267 • 37 shells (one of them is figured: Fig. 99E); Haiphong Province, Cat Ba Island, Cave Qua Vang, inside cave, large, ecologically intact active cave with speleothems; 20°48.64'N, 107°04.64'E; 60 m a.s.l.; 6 Jun. 2017; J.J. Vermeulen & K. Anker leg.; JJV 16600 • 8 shells; Haiphong Province, Cat Ba Island, Cave Qua Vang, around cave entrance, rocky limestone slope with low, somewhat mature forest; 20°48.64'N, 107°04.64'E; 100 m a.s.l.; 6 Jun. 2017; J.J. Vermeulen & K. Anker leg.; JJV 18866 (ex JJV 16604) • 41 shells; Haiphong Province, Cat Ba Island, Cave Hoa Cuong, inside cave, polluted cave disturbed by tourism, with concrete paths; 20°50.41'N, 106°59.15'E; 50 m a.s.l.; 5 Jun. 2017; J.J. Vermeulen & K. Anker leg.; JJV 16593 • 3 shells (one of them is corroded); Haiphong Province, Cat Ba Island, Cave Xa Bac, inside cave; 20°50.07'N, 106°58.61'E; 9 Jun. 2017; J.J. Vermeulen & K. Anker leg.; JJV 16606 • 13 shells; Quang Ninh Province, Halong Bay area, unnamed island 1.8 km west of the southernmost point of Cong Tai Island, Steep limestone slope bordering beach, dense vegetation; 20°52.43'N, 107°18.32'E; 3 Oct. 1998; J.J. Vermeulen & A.J. Whitten leg.; JJV 6265 • 1 shell; Haiphong Province, Cat Ba Island, limestone cliff at SE end of Viet Hai polje, base of cliff, surrounded by degraded woodland; 20°47.50'N, 107°02.90'E; 30 m a.s.l.; 10 Jun. 2017; J.J. Vermeulen & K. Anker leg.; JJV 16608 • 1 shell; Haiphong Province, Cat Ba Island, Cave Trung Trang, around cave entrance, steep limestone cliffs with vegetated ledges; 20°47.30'N, 106°59.84'E; 50 m a.s.l.; 6 Jun. 2017; J.J. Vermeulen & K. Anker leg.; JJV 18867 (ex JJV 16617) • 2 shells; Haiphong Province, Cat Ba Island, Cave Minh Chou, inside cave, cave with freshwater stream ending in the sea, much disturbed by water extraction and concrete paths; 20°45.21'N, 107°00.75'E; 50 m a.s.l.; 5 Jun. 2017; J.J. Vermeulen & K. Anker leg.; JJV 16594 • 533 shells; Quang Ninh Province, Halong Bay, Cap La Cave, deposit of soil fallen in through roof in pristine cave, vegetation outside cave tall and woody; 20°51.79'N, 107°13.54'E; 7 Mar. 2018; J.J. Vermeulen & K. Anker leg.; JJV 17634 • 10 shells, same data as for preceding, coll. HA • 22 shells; Haiphong Province, Cat Ba Island, Cave Uy Ban, inside small, disturbed cave; 20°46.39'N, 107°00.88'E; 40 m a.s.l.; 9 Jun. 2015; J.J. Vermeulen & K. Anker leg.; JJV 16613.

##### Additional material.

Vietnam • 1 figured shell (Suppl. material 3: fig. S30) + 5 shells; Thanh Hoa Province, Pu Puong National Park, limestone hill near small native village Am (locality code: WMVT.0344); 20°27.39'N, 105°13.65'E; 21 Sep. 2003; W.J.M. Maassen leg.; RMNH.5006717 • 1 shell; same data as for preceding; NMBE 550646.

##### Diagnosis.

A small to large *Angustopila* species with a conical-globular shell, a relatively wide umbilicus, and a weak, deeply set parietal tooth.

##### Description.

Shell small to large for the genus, mostly higher than wide, rarely slightly wider than high; off-white, transparent, conical or more often conical-globular, only slightly higher than wide, body whorl widest from standard apertural view; protoconch consists of 1.5 whorls, with very weak spiral striation preceding the first teleoconch whorl; teleoconch finely ornamented with irregular radial growth lines crossed by fine rows of equidistantly-spaced microscopic spiral threads (ca. 14–17 on body whorl from apertural view); on frontal and ventral surfaces of body whorl spiral and radial lines dominant; whorls 4–4.5, rounded, slightly shouldered and/or pushed from basolateral direction; aperture oblique to shell axis from lateral view; umbilicus relatively wide; aperture teardrop shaped to ovoid with straight or convex parietal part; peristome slightly expanded, not reflected; parietal callus separated from penultimate whorl; aperture with a weak (low), deeply-set parietal tooth; some shells are toothless (probably representing immature individuals).

##### Measurements (in mm).

H = 0.8–1.11, D = 0.75–0.85, H/D*100 = 97.5–117.5 (*n* = 16, specimens from the Halong Bay), RUD = 25.6–29.6 (*n* = 3); H = 0.8–0.93, D = 0.8–0.85, H/D*100 = 97.6–114.8 (*n* = 6, WMVT.0344), RUD = 30.2 (*n* = 1).

##### Differential diagnosis.

Some specimens of *A.fratermajor* sp. nov. are almost as small and have a lower spire than *A.maasseni* sp. nov. However, the two species can be distinguished based on aperture shape. Namely, that of *A.fratermajor* sp. nov. is comparatively larger, and more rounded (more impressed from parietal direction in *A.maasseni* sp. nov.), and the parietal tooth is situated deeper (reaching or nearly reaching the peristome in *A.maasseni* sp. nov.).

*Angustopilafratermajor* sp. nov. was found together with *A.apiostoma* sp. nov. in multiple samples. The two species are similar in size and general shape and therefore, careful attention needs to be given to distinguish them. The differences are the following: *Angustopilafratermajor* sp. nov. has a lower conical shell with a wider umbilicus, the body whorl is “sharper” from umbilical view (i.e., there is a tendency towards a blunt periumbilical keel); there is a distinct parietal tooth (however, it is situated deep within the shell and difficult to recognise in some specimens), which is rare and if present, very low in *A.apiostoma* sp. nov.; the parietal callus is straight or convex (concave in *A.apiostoma* sp. nov.), and the aperture is less protruding anteriorly than in the other species.

*Angustopilafabella* shells of the Halong Bay area are concave-conical in shape (conical-globular in *A.fratermajor* sp. nov.).

*Angustopilababel* sp. nov. possesses larger shells and a comparatively larger aperture with a more anteriorly situated parietal tooth than *A.fratermajor* sp. nov. *Angustopilatonkinospiroides* sp. nov. is superficially similar in shell shape, but it is larger, and has no parietal tooth. See also under *A.fraterminor* sp. nov.

##### Etymology.

The specific epithet *fratermajor* (Latin: older brother) refers to the presence of two similar (a smaller and a larger) species in the Halong Bay area.

##### Distribution.

**Figure 44.**
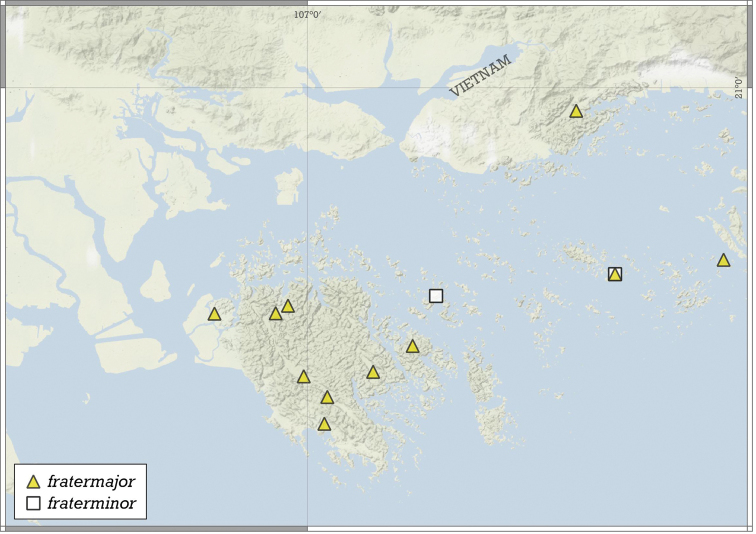
Distribution of *Angustopila* species. Note that the occurrence of *Angustopilafratermajor* Páll-Gergely & Vermeulen, sp. nov. in the Pu Puong National Park (Thanh Hoa Province) is not indicated.

This species is known from several sites of the Halong Bay area, and the sample from the Pu Puong National Park, Thanh Hoa Province is also assigned to this species (see Remarks) (Fig. 44).

##### Remarks.

The shells from the Pu Puong National Park have a weaker parietal tooth and show a more precisely shaped conical shell form than those deriving from the Halong Bay area. Therefore, we have chosen against selecting their serving as paratypes.

#### ﻿ Angustopila fraterminor

Taxon classificationAnimaliaStylommatophoraGastrocoptidae

Páll-Gergely & Vermeulen sp. nov.

EEB57B1A-E286-5EC2-8ABB-942002FF06EE

https://zoobank.org/01F82B78-E4C7-45C1-B843-A01B489F6EF7

Fig. 45

##### Type material.

***Holotype***: Vietnam • 1 empty shell (H: 0.77 mm, D: 0.7 mm); Quang Ninh Province, Halong Bay, Cap La Cave, deposit of soil fallen in through roof in pristine cave, vegetation outside cave high and woody; 20°51.79'N, 107°13.54'E; 7 Mar. 2018; J.J. Vermeulen & K. Anker leg.; HNHM 105287.

***Paratypes***: Vietnam • 368 shells; same data as for holotype; JJV 17635 • 10 shells; same data as for preceding, coll. HA • 1 shell; Quang Ninh Province, Halong Bay area, Dao Bo Hon, Song Sot Cave, drift material washed together over sinkhole in cave; 20°50.83'N, 107°05.67'E; 2 Oct. 1998; J.J. Vermeulen & A.J. Whitten leg.; JJV 6247.

##### Diagnosis.

A small to medium-sized *Angustopila* species with an ovoid shell, and a strongly developed, but deeply set parietal tooth.

##### Description.

Shell small to medium-sized for the genus, slightly higher than wide; off-white, ovoid, apex nearly domed, body whorl widest from standard apertural view; protoconch consists of 1.5 whorls, with very weak spiral striation preceding the first teleoconch whorl; teleoconch finely ornamented with irregular radial growth lines crossed by fine rows of equidistantly-spaced spiral threads; on frontal and ventral surfaces of body whorl spiral and radial lines dominant (ca. 12 on body whorl from apertural view); spiral striae occasionally with oblique scratches between rows; whorls 4, rounded or slightly shouldered; aperture oblique to shell axis from lateral view; umbilicus moderately narrow; last 1/4 whorl slightly loosely coiled (reflected in the widened umbilicus); aperture ovoid, peristome slightly expanded, not reflected; parietal callus detached from penultimate whorl; aperture with a well-developed, deeply set parietal tooth.

##### Measurements (in mm).

H = 0.75–0.93, D = 0.7–0.82, H/D*100 = 104.1–113.4 (*n* = 6), RUD = 25.7–28.6 (*n* = 3).

##### Differential diagnosis.

*Angustopilafraterminor* sp. nov. differs from *A.fratermajor* sp. nov. by the smaller, more ovoid shell, and the somewhat stronger parietal tooth. These two species also live in sympatry. See also under *A.cicatricosa* sp. nov. and *A.pusilla* sp. nov.

##### Etymology.

The specific epithet refers to *A.fratermajor* sp. nov., which is a very similar, but larger species from the same geographic area.

##### Distribution.

This species is only known from the type locality of Cap La Cave, Quang Ninh Province (Fig. 44).

##### Remarks.

The sculpture of *A.fraterminor* sp. nov. and *A.pusilla* sp. nov. is very similar, which may indicate that they are close relatives. See also under that species.

**Figure 45.**
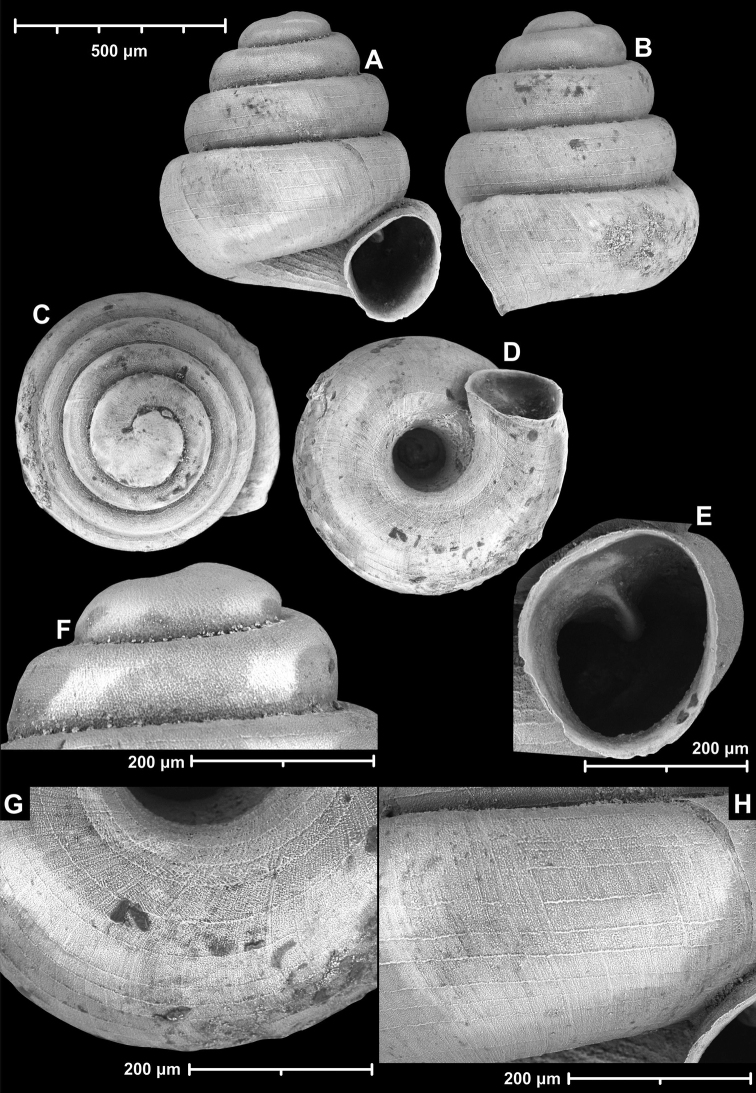
*Angustopilafraterminor* Páll-Gergely & Vermeulen, sp. nov. (holotype, HNHM 105287). Apertural (**A**), lateral (**B**), apical (**C**) and ventral (**D**) sides of the shell; aperture (**E**), microstructure of the protoconch (**F**), ventral (**G**) and frontal (**H**) surface of the body whorl.

#### ﻿ Angustopila maasseni

Taxon classificationAnimaliaStylommatophoraGastrocoptidae

Páll-Gergely & Vermeulen sp. nov.

5CCA69CA-E8A9-5ABA-8E0D-1ECC161BA830

https://zoobank.org/FFE0990E-F790-403C-8900-299E77877A12

Fig. 46

##### Type material.

***Holotype***: Vietnam • 1 empty shell (H: 0.62 mm, D: 0.7 mm); Quang Ninh Province, Halong Bay area, Phao Trong Island (locality code: WMVT.0333); 20°49.80'N, 107°08.32'E; 11 Sep. 2003; W.J.M. Maassen leg.; RMNH.5006715.

***Paratypes***: Vietnam • 1 shell; same data as for holotype; NMBE 550643 • 9 shells; same data as for holotype; RMNH 347768 • 1 shell; Quang Ninh Province, Halong Bay area, small (ca. 150 m longest section) sparsely vegetated limestone rock island; 20°52.88'N, 107°07.63'E; 3 Oct. 1998; J.J. Vermeulen & T. Whitten leg.; JJV 6227.

##### Additional material.

Vietnam • 3 juvenile shells; same data as for holotype; RMNH 347769.

##### Diagnosis.

A small, depressed-globular *Angustopila* species with a reniform aperture having an apertural axis oblique to shell axis, and a strong parietal tooth.

##### Description.

**Figure 46.**
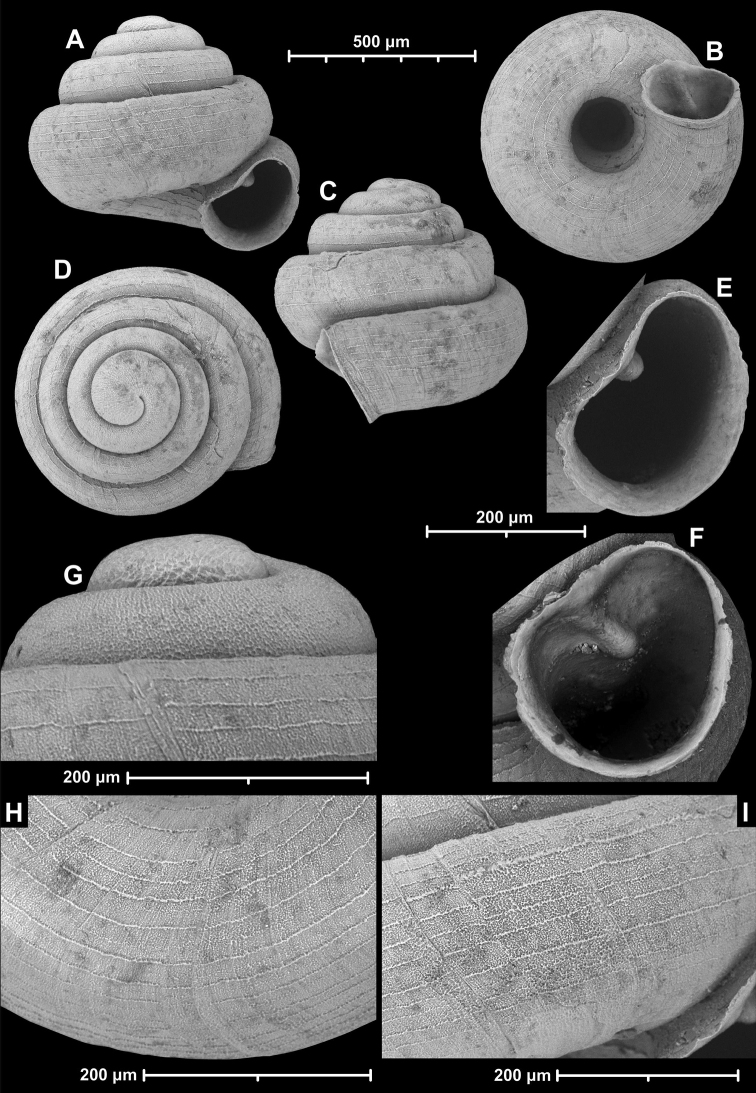
*Angustopilamaasseni* Páll-Gergely & Vermeulen, sp. nov. (holotype, RMNH.5006715). Apertural (**A**), ventral (**B**), lateral (**C**), and apical (**D**) sides of the shell; aperture (**E, F**); sculpture of the protoconch (**G**), ventral (**H**) and frontal (**I**) surface of the body whorl.

Shell small for the genus, wider than high; off-white, depressed-globular form with domed spire; body whorl widest from standard apertural view; protoconch consists of 1.25–1.5 whorls, microstructure pitted and granular with a powdery superficial texture, no spiral striation discernible; teleoconch ornamented by some fine radial growth lines and stronger, equidistantly-arranged spiral striae (ca. 13–15 on body whorl from standard apertural view); whorls 3.5, slightly shouldered; aperture slightly oblique to shell axis from lateral view; umbilicus wide; aperture reniform with strong indentation along the outer parietal margin; apertural axis joins shell axis under a large angle; sinulus wide; peristome expanded, not reflected; parietal callus strongly protruding but in line with curvature of penultimate whorl and beyond aperture edge (profile) in lateral view [Fig. 46C], detached from penultimate whorl; parietal tooth relatively weak, low, quite long, nearly reaching peristome.

##### Measurements (in mm).

H = 0.6–0.63, D = 0.67–0.97 H/D*100 = 88.4–94 (*n* = 5), RUD = 28.2–29.7 (*n* = 2).

##### Differential diagnosis.

*Angustopilasomsaki* sp. nov. is slightly larger, has a wider umbilicus, has a vestigial palatal tooth, and a protoconch with spiral striation, its reniform aperture is vertically positioned (not pushed from parietal direction causing it to be positioned sideways), and the apertural axis (sinulus–base) is oriented in a smaller angle to the shell axis. In contrast, the aperture of *A.maasseni* sp. nov. is rather kidney-shaped due to the forward angle of the body whorl, whereby the apertural axis is positioned at a larger angle in respect to the shell axis. *Angustopilapsammion* has a smaller, more depressed shell than *A.maasseni* sp. nov., and has a narrower umbilicus. Moreover, the parietal tooth of that species is pointed and short, and does not reach the peristome edge. In contrast, the parietal tooth is elongated and reaches the peristome in *A.maasseni* sp. nov. See also under *A.cicatricosa* sp. nov.

##### Etymology.

This new species is named after and dedicated to our friend and malacologist, Wim Maassen (1947–2021), who collected several of the new species described in this study.

##### Distribution.

This species is known from two nearby localities in the Halong Bay, northern Vietnam (Fig. 24).

#### ﻿ Angustopila margaritarion

Taxon classificationAnimaliaStylommatophoraGastrocoptidae

Páll-Gergely & Hunyadi sp. nov.

B017F5EE-15CB-534D-8FAE-048A87387739

https://zoobank.org/70045902-5F7C-4C67-BCEA-0E580BAC90EA

Fig. 47

##### Type material.

***Holotype***: Laos • 1 empty shell; Luang Namtha Province, 19.8 km southeast from centre of Vieng Phou Kha, south from Ban Phou Lek, southern edge of quarry (locality code: 2019/122); 20°32.54'N, 101°07.96'E; 780 m a.s.l.; 8 Oct. 2019; A. Hunyadi leg.; HNHM 105288.

***Paratypes***: Laos • 3 shells; same data as for holotype; coll. HA • 3 shells; Luang Namtha Province, 43.8 km from centre of Luang Namtha towards Vieng Phou Kha, Phou Lan, 100 m from left side of road (locality code: 2019/123); 20°44.53'N, 101°11.10'E; 770 m a.s.l.; 8 Oct. 2019; A. Hunyadi leg.; coll. HA.

##### Diagnosis.

A medium-sized *Angustopila* species with a conical-globular shell, few (ca. 3.75) whorls, glossy shells with very weak spiral striation, a strongly thickened peristome, and an elevated, club-shaped parietal tooth.

##### Description.

Shell of normal size for the genus, slightly higher than wide; conical-globular; body whorl widest in standard apertural view; protoconch consists of ca. 1.25 whorls (although the protoconch-teleoconch boundary is not clearly discernible), with very weak spiral striation; teleoconch glossy, with extremely fine growth lines and very weak spiral striae (ca. 14 on body whorl from apertural view); on both ventral and dorsal surfaces of body whorl spiral lines dominant; whorls 3.75–4, slightly shouldered; aperture slightly oblique to shell axis from lateral view; umbilicus moderately narrow; aperture ovate-oblong with a straight parietal callus; sinulus very wide, isolated due to elevated parietal tooth; peristome expanded, not reflected; parietal callus only very slightly detached from penultimate whorl; parietal tooth elevated, high, with club-shaped cross sectional view, perpendicular to parietal wall or slightly bent towards columella.

##### Measurements (in mm).

H = 0.87–0.96, D = 0.79–0.84, H/D*100 = 107.4–114.3 (*n* = 6), RUD = 23.8–27.5 (*n* = 3).

##### Differential diagnosis.

*Angustopilamargaritarion* sp. nov. lives sympatrically with *A.fabella*, which is larger, has a conical (instead of conical-globular) shell, has a matt shell surface, stronger spiral striation, and more slender parietal tooth. See also under *A.vandevenderi* sp. nov. and *A.vitrina* sp. nov.

##### Etymology.

The glossy shell resembles a tiny pearl (*μαργαριτάριον* in Greek). The specific epithet is to be used as a noun in apposition.

##### Distribution.

This new species is known from two nearby localities (ca. 23 km between them in a straight line) in Luang Namtha Province, northern Laos (Fig. 48).

**Figure 47.**
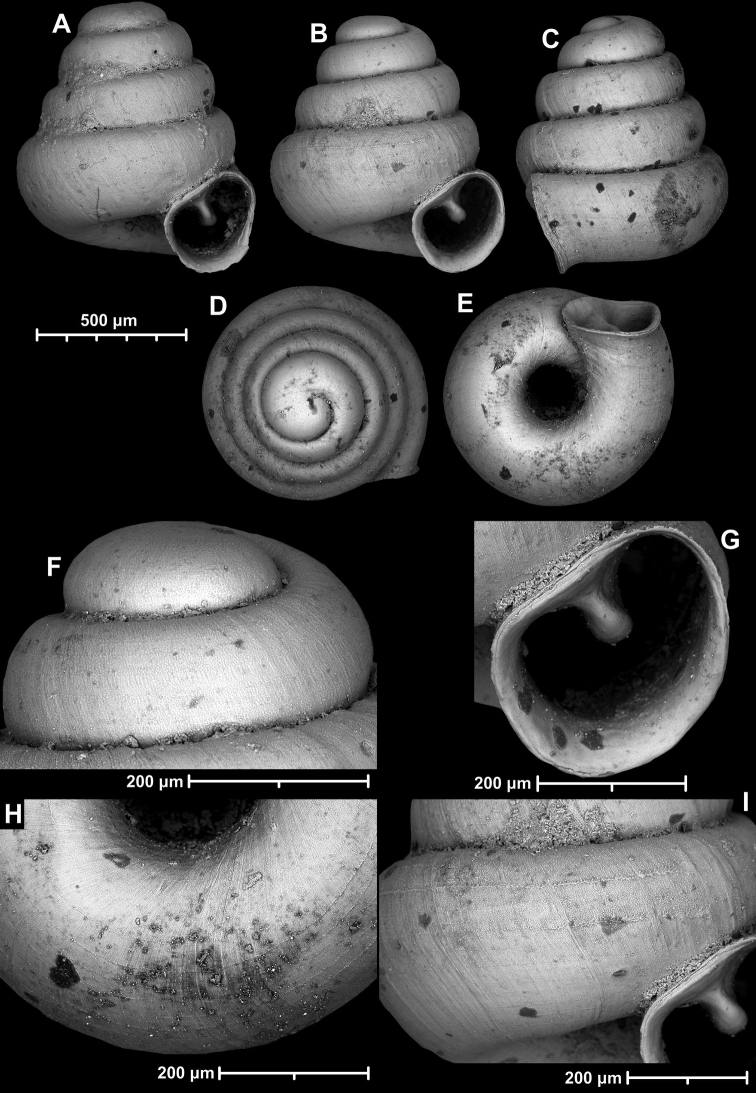
*Angustopilamargaritarion* Páll-Gergely & Hunyadi, sp. nov., paratype (**A**) and holotype (HNHM 105288) (**B–I**). Apertural (**A, B**), lateral (**C**), apical (**D**) and ventral (**E**) sides of the shell; aperture (**G**), microstructure of the protoconch (**F**), ventral (**H**) and frontal (**I**) surface of the body whorl.

**Figure 48.**
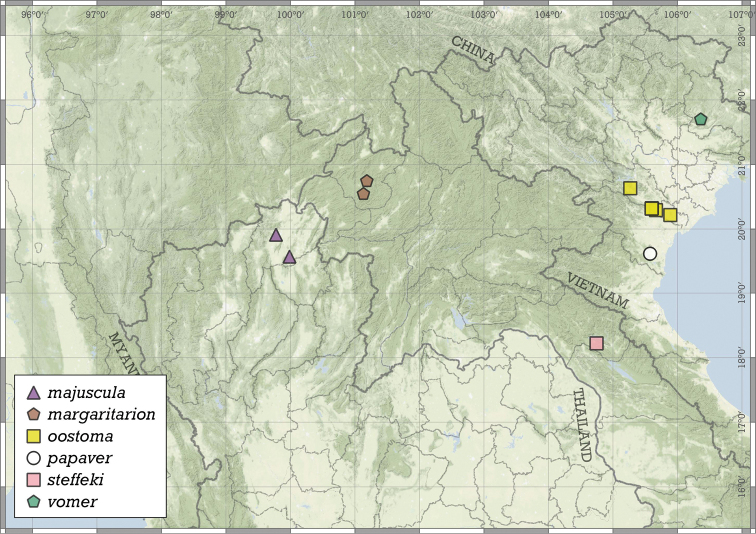
Distribution of *Angustopila* species.

#### ﻿ Angustopila megastoma

Taxon classificationAnimaliaStylommatophoraGastrocoptidae

Páll-Gergely & Vermeulen sp. nov.

C0EDD993-FE43-5681-AE39-BB9DCF4D08F7

https://zoobank.org/EA43A4A3-021A-4005-8428-57553F41D5FB

**Figure 49.**
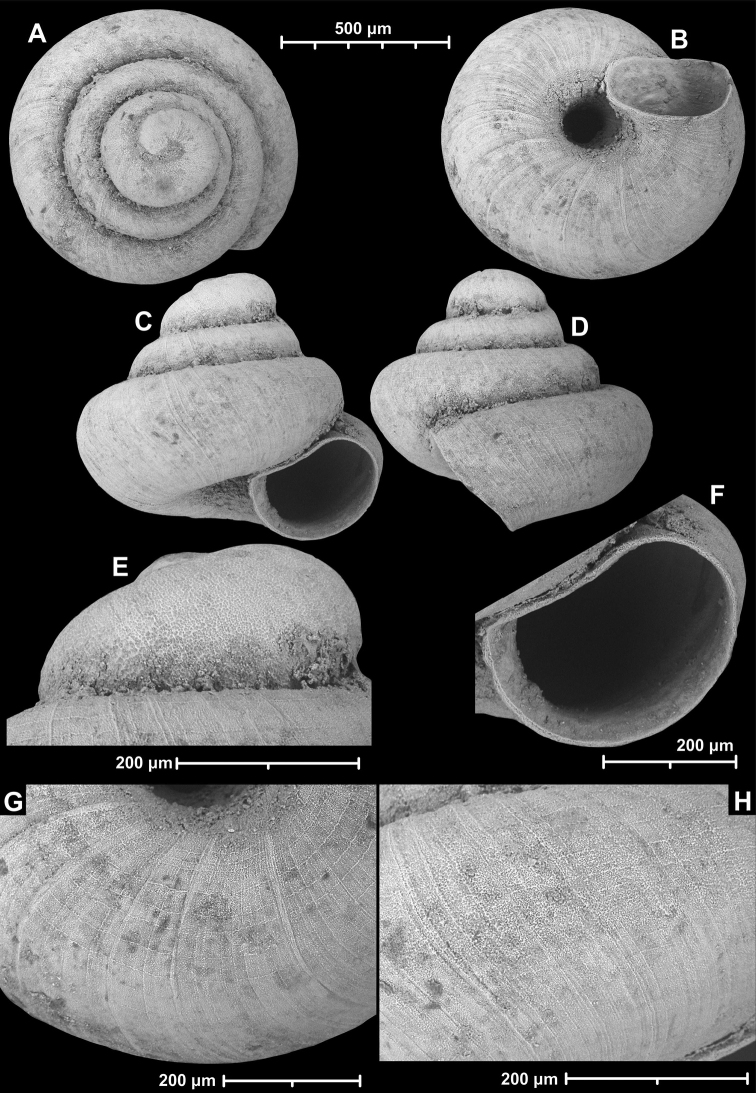
*Angustopilamegastoma* Páll-Gergely & Vermeulen, sp. nov., (holotype, RMNH 347770). Lateral (**A**), apical (**B**), apertural (**C**) and ventral (**D**) sides of the shell; sculpture on the protoconch (**E**), ventral (**F**) and frontal (**G**) surface of the body whorl.

**Figure 50.**
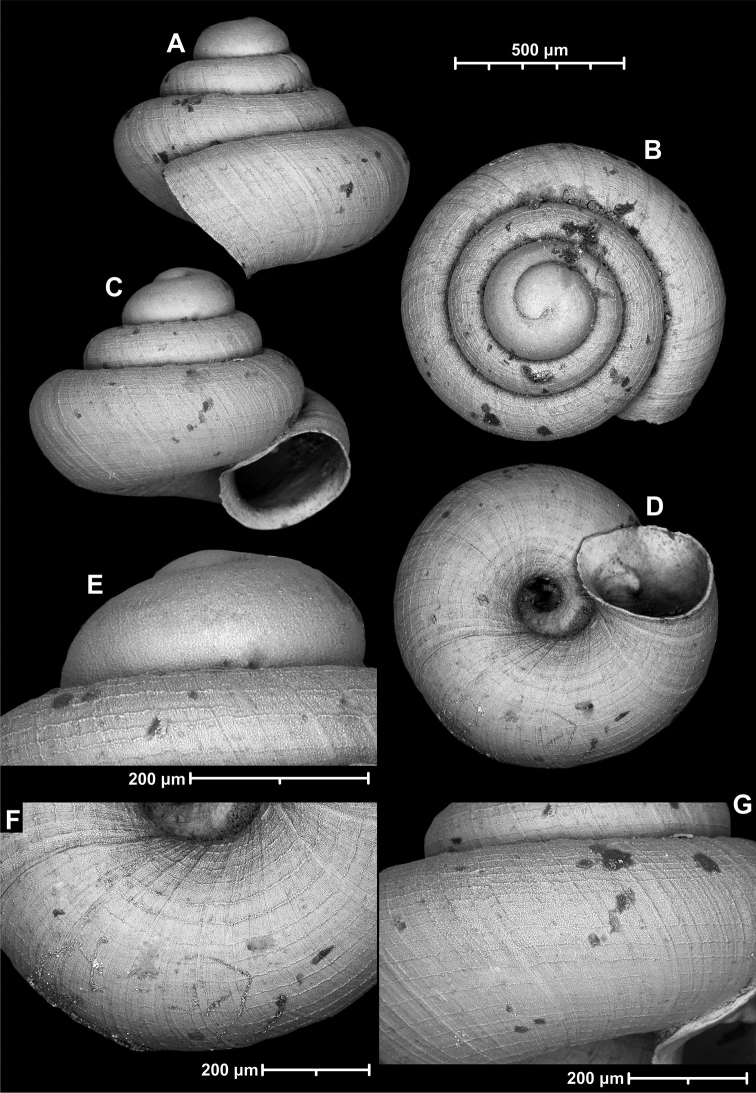
*Angustopilamegastoma* Páll-Gergely & Vermeulen, sp. nov., JJV 16596. Lateral (**A**), apical (**B**), apertural (**C**) and ventral (**D**) sides of the shell; sculpture on the protoconch (**E**), ventral (**F**) and frontal (**G**) surface of the body whorl.

Figs 49 , 50 , Suppl. material 3: figs S31–S33

##### Type material.

***Holotype***: Vietnam • 1 empty shell (H: 0.8 mm, D: 1.0 mm); Haiphong Province, Cat Ba Island, near National Park Headquarters, Trung Trang Cave (locality code: Vietnam 02); 20°47.47'N, 106°59.41'E; 5 Sep. 1998; W.J.M. Maassen leg.; RMNH 347770.

***Paratypes***: Vietnam • 21 adult shells; same data as for holotype; RMNH 347771 • 1 shell; Quang Ninh Province, Halong Bay area, Phao Trong Island (locality code: WMVT.0333); approx. GPS coordinates: 20°49.80'N, 107°08.32'E; 11 Nov. 2003; W.J.M. Maassen leg.; RMNH 347772 • 7 shells; Haiphong Province, Cat Ba Island, Cave Qua Vang, around cave entrance, rocky limestone slope with low, rather mature forest; 20°48.64'N, 107°04.64'E; 100 m a.s.l.; 6 Jun. 2017; J.J. Vermeulen & K. Anker leg.; JJV 16597 • 221 shells; Haiphong Province, Cat Ba Island, Cave Xa Bac, around cave entrance; 20°50.07'N, 106°58.61'E; 30 m a.s.l.; 9 Jun. 2017; J.J. Vermeulen & K. Anker leg.; JJV 16625 • 615 shells; Haiphong Province, Cat Ba Island, Cave Xa Bac, inside cave; 20°50.07'N, 106°58.61'E; 30 m a.s.l.; 9 Jun. 2017; J.J. Vermeulen & K. Anker leg.; JJV 16624 • 10 shells; same data as for preceding; coll. HA • 210 shells; Quang Ninh Province, Halong Bay area, Dao Bo Hon, Song Sot Cave, drift material washed together over sinkhole in cave; 20°50.83'N, 107°05.67'E; 2 Oct. 1998; J.J. Vermeulen & A.J. Whitten leg.; JJV 6237 • 3 shells; Haiphong Province, Cat Ba Island, Cave Qua Vang, inside cave, large, ecologically intact active cave with speleothems; 20°48.64'N, 107°04.64'E; 60 m a.s.l.; 6 Jun. 2017; J.J. Vermeulen & K. Anker leg.; JJV 16596 • 7 shells; Quang Ninh Province, Halong Bay area, unnamed island in Dau Moi Temple area (locality code: WMVT.0338); 20°55.69'N, 107°09.40'E; 13 Sep. 2003; W.J.M. Maassen leg.; RMNH 347773 • 5 shells; Haiphong Province, Cat Ba Island, along trail from headquarters to Viet Hai, along Ao Eck, sampling starting at coordinates, until 0.5 km beyond Ao Eck; 20°48.07'N, 107°00.83'E; 100 m a.s.l.; 4 Jun. 2017; J.J. Vermeulen & K. Anker leg.; JJV 17656 (ex JJV 16618) • 12 shells; Haiphong Province, Cat Ba Island, limestone cliff at SE end of Viet Hai polje, foot of cliff, surrounded by degraded woodland; 20°47.50'N, 107°02.90'E; 30 m a.s.l.; 10 Jun. 2017; J.J. Vermeulen & K. Anker leg.; JJV 16626 • 27 shells; Haiphong Province, Halong Bay area, unnamed island off E Coast Cat Ba, south facing bay with beach and densely vegetated limestone scree slope; 20°45.19'N, 107°4.45'E (approximate GPS data); 1 Oct. 1998; J.J. Vermeulen & K. Anker leg.; JJV 17653 (ex JJV 6236).

##### Additional material.

Vietnam • 7 j/b shells; same data as for holotype; RMNH 347774 • 4 j/b shells; Quang Ninh Province, Halong Bay area, Phao Trong Island (locality code: WMVT.0333); approx. GPS data: 20°49.80'N, 107°08.32'E; 11 Nov. 2003; W.J.M. Maassen leg.; RMNH 347775 • 55 j/b shells; Haiphong Province, Cat Ba Island, Cave Xa Bac, around cave entrance; 20°50.07'N, 106°58.61'E; 30 m a.s.l.; 9 Jun. 2017; J.J. Vermeulen & K. Anker leg.; JJV 17660 (ex JJV 16625).

##### Diagnosis.

A large, asymmetrically coiled, concave-conical, depressed species with few whorls, weak sculpture, narrow umbilicus, a comparatively large, strongly oblique aperture, and a tiny, pointed parietal tooth (can be absent).

##### Description.

Shell large for the genus, wider than high, concave-conical, depressed; protoconch consists of 1.25–1.5 whorls, with weak spiral striation preceding the first teleoconch whorl; teleoconch sculpture overall weak, with irregular radial growth lines and dense, weak, rather regular spiral striation (ca. 18–20 spiral striae on body whorl in apertural view); whorls 3.5–3.75, body whorl rounded or depressed-rounded; aperture strongly oblique to shell axis in lateral view; aperture reniform due to concave parietal side (body whorl strongly attached to the penultimate whorl); peristome slightly expanded, not reflected; parietal callus smeared onto penultimate whorl, although its edge is sharp, not fused to penultimate whorl; umbilicus narrow, 1/4 to 1/5 of shell width, its edge slightly covered by reflected peristome edge; aperture sometimes toothless or with low, pointed parietal tooth.

##### Measurements (in mm).

H = 0.79–0.91, D = 0.91–1.05, H/D*100 = 79.0–91.6 (*n* = 13), RUD = 24.5–25.5 (*n* = 3).

##### Differential diagnosis.

Based on the low conical shell and the narrow umbilicus, this species can be easily recognised and distinguished from other *Angustopila* species, except for *A.thersites* sp. nov., which also occurs sympatrically. For comparisons, see under that species.

*Tonkinospiradefixa* is larger, has a more depressed shell, a wider umbilicus, and keeled body whorl, and a stronger spiral sculpture. *Tonkinospirachytrophora* is larger, has a more globular shell and a stronger sculpture.

##### Etymology.

The specific epithet *megastoma* (from Greek *μέγας* = big and *στόμα* = mouth) refers to the large aperture characteristic for this species.

##### Distribution.

This species is known from several sites in the Halong Bay Area, northern Vietnam (Fig. 22).

#### ﻿ Angustopila oostoma

Taxon classificationAnimaliaStylommatophoraGastrocoptidae

Páll-Gergely & Vermeulen sp. nov.

E972FC6A-323A-5C2A-B3E9-6FF4072666AF

https://zoobank.org/9C120D4D-9473-4CB6-8C19-E02953CD7038

**Figure 51.**
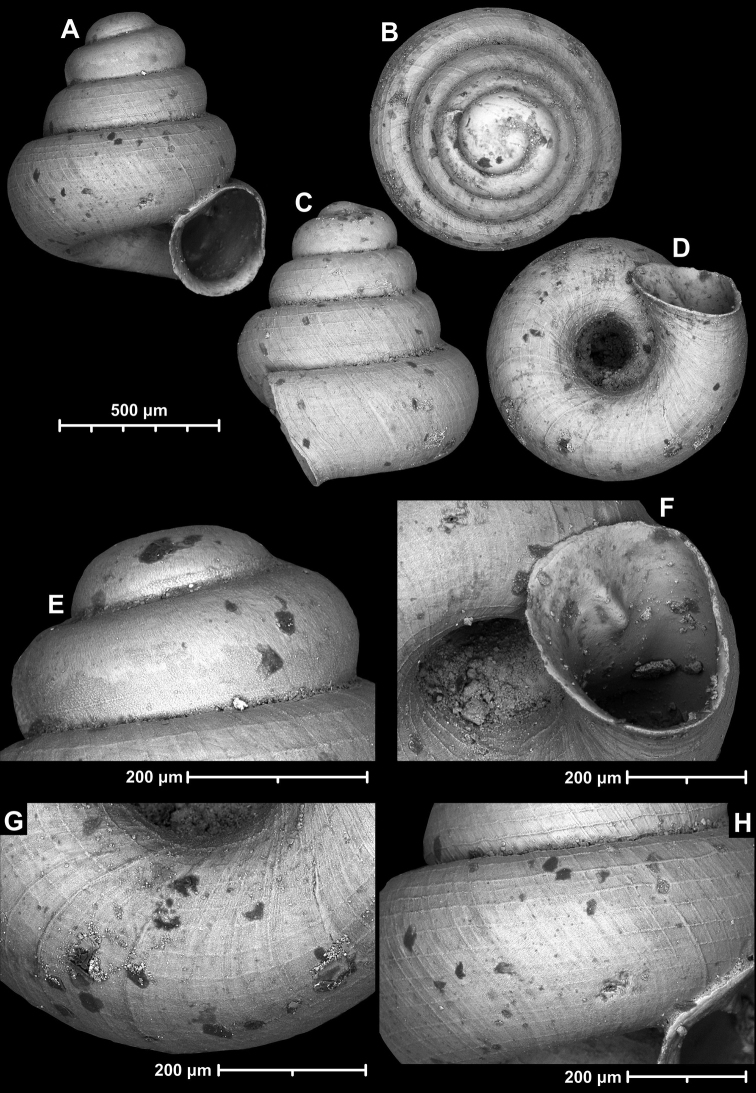
*Angustopilaoostoma* Páll-Gergely & Vermeulen, sp. nov. (holotype, HNHM 105289). Apertural (**A**), apical (**B**), lateral (**C**) and ventral (**D**) sides of the shell; eroded surface showing some microstructure on the protoconch (**E**), aperture (**F**), ventral (**G**) and frontal (**H**) surface of the body whorl.

**Figure 52.**
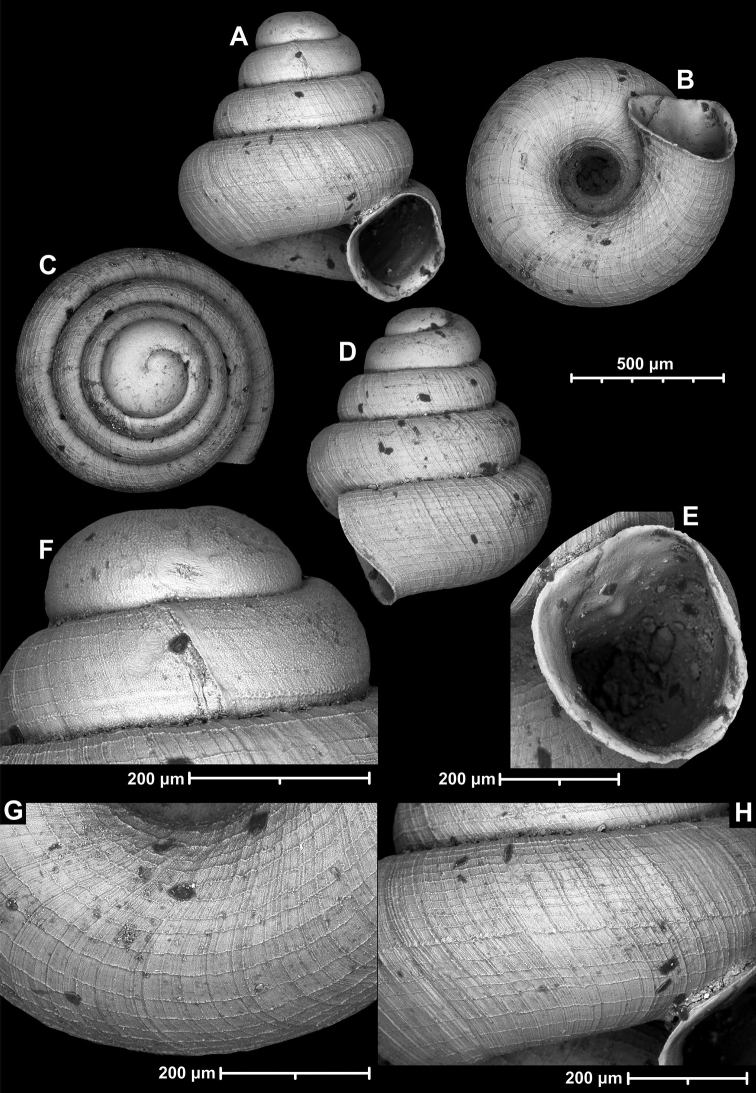
*Angustopilaoostoma* Páll-Gergely & Vermeulen, sp. nov. (2020/28). Apertural (**A**), ventral (**B**), apical (**C**) and lateral (**D**), sides of the shell; aperture (**E**), microstructure of the protoconch showing protoconch-teleoconch boundary (**F**), ventral (**G**) and frontal (**H**) surface of the body whorl.

Figs 51 , 52 , Suppl. material 3: fig. S34

##### Type material.

***Holotype***: Vietnam • 1 empty shell (H: 0.89 mm, D: 0.81 mm); Ninh Binh Province, Cuc Phuong National Park, path to Fairy Cave, primary forest, limestone outcrops in shale bedrock; 20°13.06'N, 105°53.43'E; 10 October 1998; J.J. Vermeulen & L. Deharveng leg.; HNHM 105289 (original inventory number: JJV 6224).

***Paratypes***: Vietnam • 1 adult shell with imperfectly grown last whorl; Ninh Binh Province, Cuc Phuong National Park, 1 km WNW of Xom Bong, primary forest, small limestone outcrops in clay soil; approximate GPS coordinates: 20°55.57'N, 105°9.24'E; 10 Oct. 1998; J.J. Vermeulen & L. Deharveng leg.; JJV 6251 • 2 shells; Ninh Binh Province, Cuc Phuong National Park, 2 km road Xom Bong to Headquarters, primary forest, limestone outcrops in thick clayey soil; 10 Oct. 1998; J.J. Vermeulen & L. Deharveng leg.; JJV 6222.

##### Additional material.

Vietnam • 3 adult shells; Hòa Bình Province, Tân Lạc District, 1300 m west from Quy Hậu on road no. 6, rock wall (locality code: 2020/28); 20°37.93'N, 105°16.17'E; 150 m a.s.l.; 11 Feb. 2020; A. Hunyadi leg.; coll. HA • 3 j/b shells; Ninh Binh Province, Cuc Phuong National Park, 2 km road Xom Bong to Headquarters, primary forest, limestone outcrops in thick clayey soil; 10 Oct. 1998; J.J. Vermeulen & L. Deharveng leg.; JJV 17629 (ex 6222) • 1 shell; Ninh Binh Province, Cuc Phuong National Park, Prehistoric Man Cave, steep limestone slope with disturbed forest; 20°17.61'N, 105°40.11'E; 10 Oct. 1998; J.J. Vermeulen & L. Deharveng leg.; JJV 6252.

##### Diagnosis.

A medium-sized *Angustopila* species with a conical shell, an ovate aperture and a deeply-set, weak parietal tooth.

##### Description.

Shell of normal size for the genus, slightly higher than wide, body whorl widest from standard apertural view; protoconch consists of 1.25–1.5 whorls, with fine spiral striation; specimens from the type locality with weaker, the shell from site 2020/28 with stronger and denser radial lines; typical shells with ca. 14, the shell from site 2020/28 with 19 spiral striae on body whorl from apertural view; whorls 4, rounded; aperture slightly oblique to shell axis from lateral view; umbilicus wide; aperture large, ovoid (higher than wide) to ovate-subquadrate; peristome slightly expanded, not reflected; parietal callus does not elevate from penultimate whorl; parietal tooth elongated, low, deeply-set (does not reach parietal callus).

##### Measurements (in mm).

H = 0.89–1.0, D = 0.81–0.95, H/D*100 = 105.3–111.6 (*n* = 7), RUD = 27.4–32.3 (*n* = 4).

##### Differential diagnosis.

Generally larger than *A.elevata*, has a rather conical shell (concave-conical in *A.elevata*), a less pointed apex, a wider umbilicus, and a comparatively larger, ovate aperture (although *A.elevata* samples 2020/18 and 2019/120 have a rather oval aperture) with a weak parietal tooth. In contrast, *A.elevata* is always toothless. We note that the tooth-like thickening of the parietal callus in one *A.elevata* shell (Páll-Gergely et al. 2017: fig. 7F) is not considered to be a tooth. Moreover, although not in all *A.elevata* populations, the radial sculpture of that species is more prominent. Populations assigned to *Angustopilafabella* are larger, possess a stronger parietal tooth, a comparatively smaller aperture and usually, a more pointed apex.

##### Etymology.

The specific epithet refers to the ovoid aperture (*ᾠόν* = egg, *στόμα* = mouth).

##### Distribution.

This new species is known from two sites: the Cuc Phuong National Park (type locality) and another site ca. 40 km northwest (Fig. 48).

##### Remarks.

The single shell collected at the Cave of Prehistoric Man at Cuc Phuong National Park (JJV 6252) lacks a parietal tooth, has a narrower umbilicus, and weaker sculpture than the other *A.oostoma* sp. nov. shells. Thus, it may belong to a different species. We refer to it herein as A.cf.oostoma sp. nov.

#### ﻿ Angustopila prolixa

Taxon classificationAnimaliaStylommatophoraGastrocoptidae

Páll-Gergely & Hunyadi sp. nov.

087FA79F-36DB-5C62-B2B0-3BA9CE1747C2

https://zoobank.org/154CBF6C-709D-4109-A00E-541E28D2EC65

Fig. 53

##### Type material.

***Holotype***: Vietnam • 1 empty shell (H: 0.67 mm, D: 0.73 mm); Lạng Sơn Province, Hữu Lũng District, Minh Tiến, 1400 m northeast Cầu Cheo Minh Tiến (locality code: 2020/55); 21°34.02'N, 106°17.79'E; 30 m a.s.l.; 20 Feb. 2020; A. Hunyadi leg.; HNHM 105290.

***Paratypes***: Vietnam • 12 shells; same data as for holotype; coll. HA.

##### Additional material.

Vietnam • 1 j/b shell; same data as for holotype; coll. HA.

##### Diagnosis.

A small, domed *Angustopila* species with a very high and long parietal tooth.

##### Description.

Shell small for the genus, slightly wider than high or as wide as high; transparent, pale grey, dorsal side domed; body whorl widest from standard apertural view; protoconch consists of almost 1.75 whorls, with strong spiral striation; teleoconch ornamented by weak, irregular radial growth lines and irregularly spaced, much stronger spiral striae (ca. 10 or 11 on body whorl from standard apertural view); whorls 3.75, rounded or depressed-rounded; aperture slightly oblique to shell axis from lateral view; umbilicus very wide; parietal callus detached from penultimate whorl; aperture pear-shaped; peristome expanded but not reflected; parietal tooth starts from parietal callus, strongly elongated inside aperture, conspicuously elevated (extends beyond 1/2 aperture), slightly curves in direction of palatal wall.

**Figure 53.**
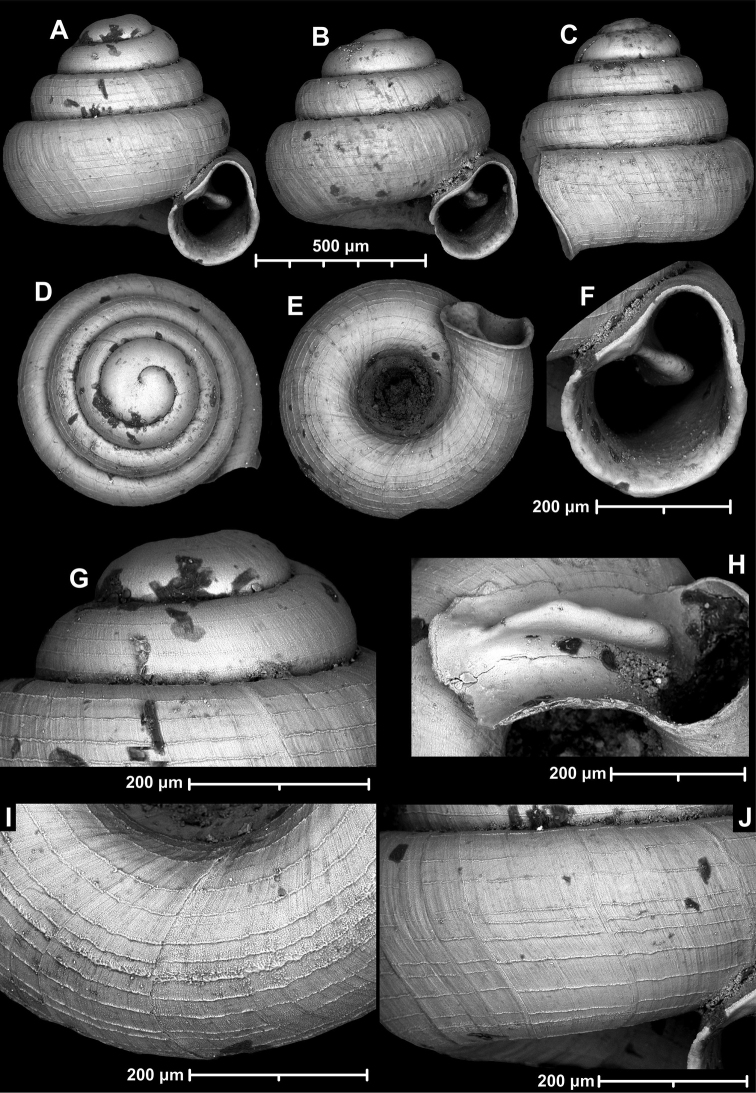
*Angustopilaprolixa* Páll-Gergely & Hunyadi, sp. nov. Holotype (HNHM 105290): **A, C–G, I–J**), paratypes (2020/55, specimen 1: **B** 2020/55, specimen 3: **H**). Apertural (**A, B**), lateral (**C**), apical (**D**) and ventral (**E**) sides of the shell; aperture (**F**), sculpture on the protoconch (**G**), parietal tooth (**H**); ventral (**I**) and frontal (**J**) surface of the body whorl.

##### Measurements (in mm).

H = 0.67–0.77, D = 0.73–0.78, H/D*100 = 91.8–100 (*n* = 5), RUD = 33.8–36.4 (*n* = 2).

##### Differential diagnosis.

The nearby occurring, *A.vomer* sp. nov., is similar in possessing a highly elevated, long parietal tooth. However, it has a larger, concave-conical (rather than domed) shell, a narrower umbilicus, a more rounded aperture (wider sinulus), and its parietal tooth does not turn towards the palatal wall, and does not reach the parietal callus. The most similar species in terms of shell and aperture shape is *A.quadridens* sp. nov., which possesses four teeth.

##### Etymology.

The specific epithet (*prolixo* = elongated in Latin) refers to the unusually long parietal tooth of this new species.

##### Distribution.

This new species is known only from its type locality (Fig. 27).

#### ﻿ Angustopila psammion

Taxon classificationAnimaliaStylommatophoraGastrocoptidae

Páll-Gergely, Anker & Vermeulen, 2022

42B7AD41-089D-5D58-8482-2A7A0B4DD9D2

Fig. 7

Angustopila psammion Páll-Gergely, Anker & Vermeulen, in Páll-Gergely et al. 2022: 69, fig. 3.

##### Type locality.

“Vietnam, Quang Ninh Province, Halong Bay, Cap La Cave, 20°51'47.61"N 107°13'32.46"E, soil deposit fallen through roof in pristine cave, vegetation outside cave tall and woody, J.J. Vermeulen & K. Anker leg., 07.03.2018, JJV 17633.”

##### Diagnosis.

A small *Angustopila* species with a depressed-globular shell with dome-shaped spire, thick spiral striae, kidney-shaped aperture with single parietal denticle not reaching parietal callus.

##### Measurements (in mm).

H = 0.46–0.57, D = 0.6–0.68, H/D*100: 75.7–88.5 (*n* = 24), RUD = 27.7–28.1 (*n* = 2).

##### Differential diagnosis.

See under *A.cicatricosa* sp. nov. and *A.maasseni* sp. nov.

##### Distribution.

This species is known only from its type locality (Fig. 24).

##### Remarks.

Currently the smallest known land snail globally (Páll-Gergely et al. 2022).

#### ﻿ Angustopila pusilla

Taxon classificationAnimaliaStylommatophoraGastrocoptidae

Páll-Gergely & Hunyadi sp. nov.

7F76822C-62C7-518B-9109-1EE055E38D21

https://zoobank.org/2CA4DCF6-4B6F-4453-808D-EDB2055348FD

Fig. 54

##### Type material.

***Holotype***: Laos • 1 empty shell (H: 0.77 mm, D: 0.7 mm); Vientiane Province, 4.5 km west from centre of Vang Vieng, Phone Ngeun, Tham Khan Kham (locality code: 2019/133); 18°55.53'N, 102°24.95'E; 280 m a.s.l.; 14 Oct. 2019; A. Hunyadi leg.; HNHM 105291.

***Paratypes***: Laos • 5 shells; same data as for holotype; coll. HA • 3 shells; Vientiane Province, 7.5 km west from centre of Vang Vieng, Ban Naka, Tham Poukham (locality code: 2019/131); 18°55.61'N 102°23.77'E; 240 m a.s.l.; 14 Oct. 2019; A. Hunyadi leg.; coll. HA.

##### Additional material.

Laos • 3 j/b shells; same data as for holotype; coll. HA.

##### Diagnosis.

A small *Angustopila* species with an ovoid shell, oblique scratches between spiral striae; a suboval aperture with sharp edge and a deeply set parietal tooth.

##### Description.

Shell small for the genus, slightly higher than wide; off-white, ovoid, apex nearly domed, body whorl widest from standard apertural view; protoconch consists of 1.25 whorls, with very weak spiral striation; teleoconch finely ornamented with irregular radial growth lines crossed by much stronger rows of equidistantly-spaced microscopic spiral threads; on frontal and ventral surfaces of body whorl spiral and radial lines dominant (ca. 10–12 on body whorl from apertural view); spiral striae with oblique scratches between rows; whorls 4.5, slightly shouldered; aperture slightly oblique to shell axis from lateral view; umbilicus moderately wide; aperture ovoid, peristome very slightly expanded, not reflected, sharp; parietal callus detached from penultimate whorl; aperture with a tiny parietal tooth situated close to parietal callus, but not reaching it.

##### Measurements (in mm).

H = 0.74–0.77, D = 0.64–0.7, H/D*100 = 110–115.6 (*n* = 4), RUD = 26.5–27.9 (*n* = 2).

##### Differential diagnosis.

*Angustopilapusilla* sp. nov. is most similar to *A.fraterminor* sp. nov. in terms of shell size, shape, aperture shape, the oblique scratches between spiral striae and the presence of a single parietal tooth. However, the tooth is situated deep in *A.fraterminor* sp. nov. and near the peristome edge in *A.pusilla* sp. nov. Moreover, the spiral striation is much more prominent (elevated) in *A.pusilla* sp. nov.

##### Distribution.

This species is known from two nearby localities in Vientiane Province, northern Laos (Fig. 55).

**Figure 54.**
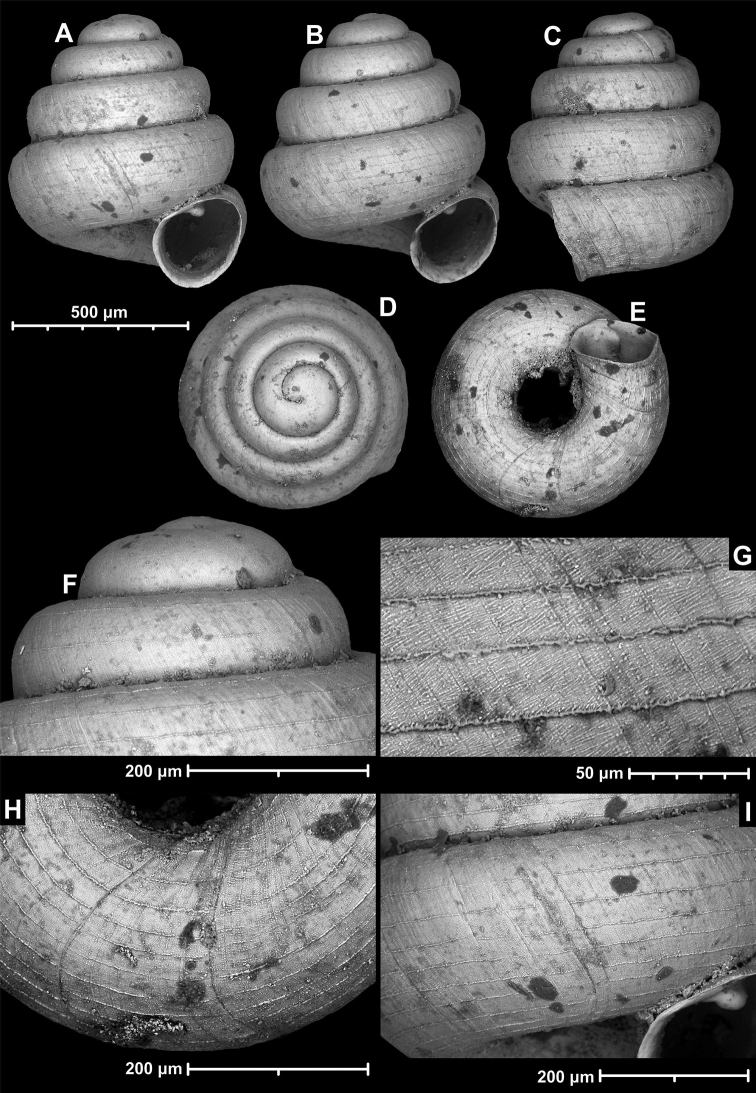
*Angustopilapusilla* Páll-Gergely & Hunyadi, sp. nov. Holotype (HNHM 105291) (**A, C–I**) and paratype (**B**). Apertural (**A, B**), lateral (**C**), apical (**D**) and ventral (**E**) sides of the shell; microstructure of the protoconch (**F**), fine sculpture of the frontal part of the body whorl (**G**), ventral (**H**) and frontal (**I**) surface of the body whorl.

**Figure 55.**
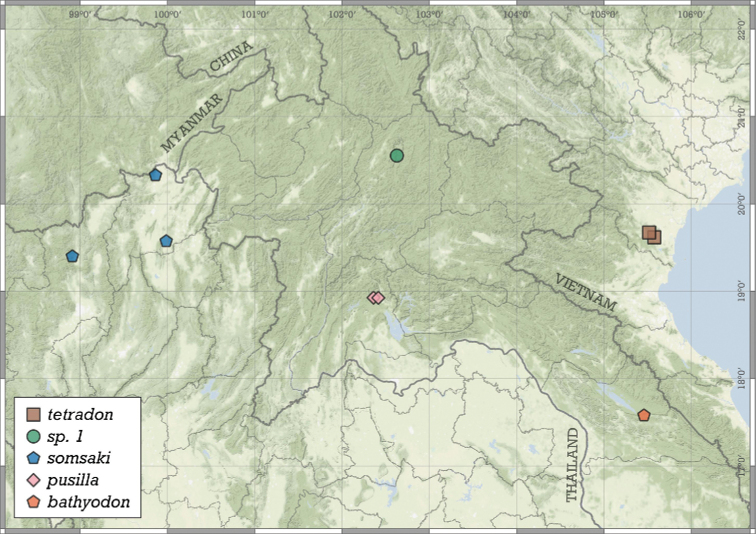
Distribution of *Angustopila* species.

##### Remarks.

The oblique scratches between the spiral striae of the teleoconch are known in only two species, *A.pusilla* sp. nov. and *A.fraterminor* sp. nov. This may, despite the large geographic distance, indicate their close relatedness.

#### ﻿ Angustopila szekeresi

Taxon classificationAnimaliaStylommatophoraGastrocoptidae

Páll-Gergely & Hunyadi, 2015

C07A73CC-DA66-5F3B-B3E9-E8FBC045A737

**Figure 56.**
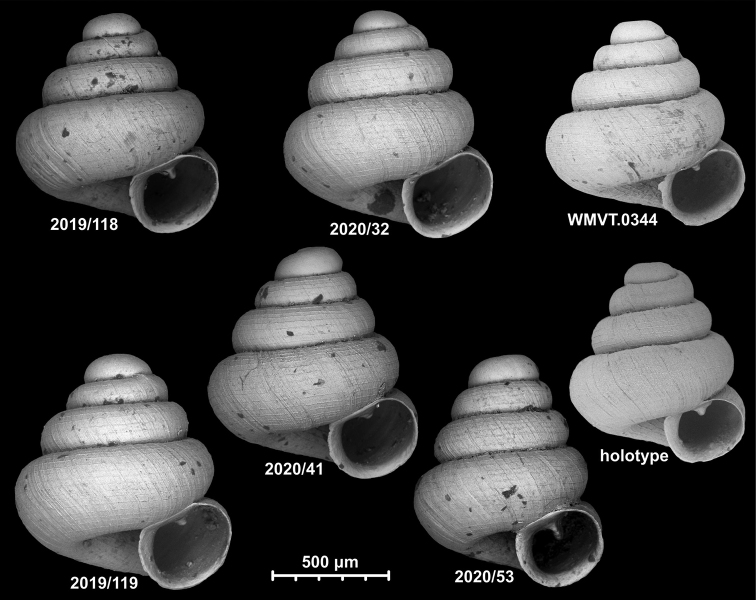
Synoptic view of shells from different *Angustopilaszekeresi* Páll-Gergely & Hunyadi, 2015 populations.

Fig. 56 , Suppl. material 3: figs S35–S39

Angustopila szekeresi Páll-Gergely et al., 2015a: 42–45, fig. 5.

##### Type locality.

“China, Guangxi (广西), Hechi Shi (河池市), Bama Xian (巴马县), cliffs at the southern edge of Jiaole Cun (交乐村), 590 m, 24°7.045'N, 107°7.847'E.”

##### Material examined.

Vietnam • 1 figured + 1 juvenile shell; Thanh Hoa Province, Pu Puong National Park, limestone hill near small native village Am (locality code: WMVT.0344); 20°27.39'N, 105°13.65'E; 21 Sep. 2003; W.J.M. Maassen leg.; RMNH.5006720 • 59 adult + 36 j/b shells; Thanh Hóa Province, Như Thanh District, Hải Vân, Hang Lò Cao Kháng Chiến, vicinity of the cave (locality code: 2020/41); 19°37.08'N, 105°34.63'E; 20 m a.s.l.; 14 Feb. 2020; A. Hunyadi leg.; coll. HA • 26 adult shells; Thanh Hóa Province, Như Thanh District, 600 m south from Xuân Khang along road no. 45, around the cave temple (locality code: 2020/40); 19°40.35'N, 105°31.07'E; 80 m a.s.l.; 14 Feb. 2020; A. Hunyadi leg.; coll. HA • 288 adult + 122 j/b shells; Thanh Hóa Province, Lang Chánh District, Đồng Lương, 9.4 km northeast from centre of Lang Chánh towards Làng Thung (locality code: 2020/32); 20°11.52'N, 105°15.59'E; 300 m a.s.l.; 12 Feb. 2020; A. Hunyadi leg.; coll. HA • 3 shells; Sơn La Province, Mộc Châu, Hang Dơi, around the entrance of the cave (locality code: 2020/26); 20°50.96'N, 104°38.34'E; 865 m a.s.l.; 11 Feb. 2020; A. Hunyadi leg.; coll. HA • 12 shells; Hòa Bình Province, Kim Bȏi District, Cao Dương, north of Đồng Phú, 58 km from Nho Quan towards Hanoi on the Hồ Chí Minh road (locality code: 2020/47); 20°42.59'N, 105°39.30'E; 10 m a.s.l.; 16 Feb. 2020; A. Hunyadi leg.; coll. HA • 9 adult + 10 j/b shells; Sơn La Province, Yên Châu District, Xã Chiềng On, Bản Trạm Hốc, Hang Nhả Nhung, around the cave (locality code: 2020/20); 20°59.48'N, 104°11.27'E; 970 m a.s.l.; 9 Feb. 2020; A. Hunyadi leg.; coll. HA • 1 shell; Lạng Sơn Province, Hữu Lũng District, Hữu Liên, cross of roads no. 1B-241, 33.5 km towards Ba Nàng, left side of road (locality code: 2020/52); 21°41.06'N, 106°22.87'E; 220 m a.s.l.; 19 Feb. 2020; A. Hunyadi leg.; coll. HA (with unusually high parietal tooth) • 1 shell; Lạng Sơn Province, Hữu Lũng District, Hữu Liên, 1400 m west from Đȏng Lâm along road no. 241 (locality code: 2020/53); 21°41.91'N, 106°21.77'E; 210 m a.s.l.; 19 Feb. 2020; A. Hunyadi leg.; coll. HA (with unusually high parietal tooth).

Laos • 141 adult + 42 j/b shells; Udomxai Province, 6.5 km southeast from centre of Na Mor towards Udomxai, Ban Nathong, Tham Nathong, below cave spring (locality code: 2019/118); 20°52.37'N, 101°46.98'E; 635 m a.s.l.; 7 Oct. 2019; A. Hunyadi leg.; coll. HA • 75 adult shells; Udomxai Province, 10 km south of centre of Na Mor, 3.8 km east-southeast of Na Xay, rock wall facing north (locality code: 2019/119); 20°53.37'N, 101°48.96'E; 660 m a.s.l.; 7 Oct. 2019; A. Hunyadi leg.; coll. HA • 12 adult + 6 j/b shells; Luang Prabang Province, 12 km from centre of Nong Khiaw towards Pak Xeng, northeast from Ban Huai Lek, left side of the road (locality code: 2019/115); 20°32.59'N, 102°41.16'E; 5 Oct. 2019; A. Hunyadi leg., coll. HA.

##### Diagnosis.

A small to medium-sized *Angustopila* species with conical to slightly conical-globular shell (shell height variable), an adnate peristome, and a weak parietal tooth (sometimes absent).

##### Measurements (in mm).

H = 0.82–0.96, D = 0.77–0.9, H/D*100 = 96.6–126.5 (*n* = 50, newly collected populations), RUD = 18.1–25.3 (*n* = 11); H = 0.88–1.03, D = 0.77–0.89, H/D*100 = 82.5–92.1 (shells from the type locality, *n* = 6).

##### Differential diagnosis.

Distinguishable from all congeners (including the widespread *A.fabella*, which also lives in sympatry) based on the conical-globular shell, the relatively large aperture, and the deeply situated parietal tooth.

##### Distribution.

**Figure 57.**
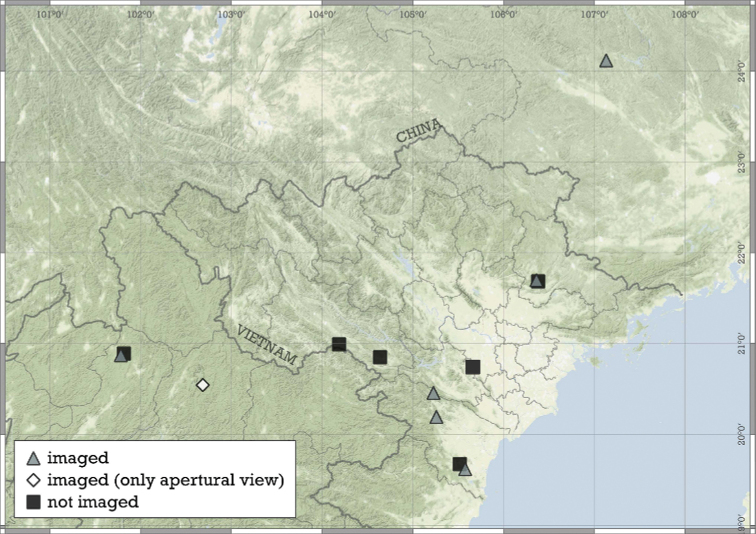
Distribution of *Angustopilaszekeresi* Páll-Gergely & Hunyadi, 2015.

*Angustopilaszekeresi* was described from Guangxi Province, China. Here we report this species from several new localities in northern Laos and northern Vietnam (Fig. 57).

##### Remarks.

Most of the newly discovered populations show little variability in terms of shell characters. The only notable samples are 2020/52 and 2020/53 (distance between them is only 2.5 km) in Vietnam, which have an unusually high parietal tooth and a conical (not conical-globular) shell. More material is necessary to infer the taxonomic position of the latter populations because, for both samples, single shells are all we have. For the time being, they are referred to as Angustopilacf.szekeresi. We do not assign these two populations to *A.fabella* because they are both sympatric with *A.fabella* (distinguishable based on the larger, proportionally wider shell).

The shells from the type locality are taller than the newly collected ones. However, no other important shell characters are found to be different.

#### ﻿ Angustopila vandevenderi

Taxon classificationAnimaliaStylommatophoraGastrocoptidae

Páll-Gergely & Jochum sp. nov.

F7156F1E-B1AF-5D9C-8726-6C3051AB7C26

https://zoobank.org/C9F2FB15-5E0E-4FFF-9923-C8C70EACE9C7

Fig. 58

##### Type material.

***Holotype***: Vietnam • 1 empty shell (H: 0.8 mm, D: 0.74 mm); Cuc Phuong National Park; 20°17.80'N, 105°39.66'E; 14–16 May 2008; W. van Devender leg.; VNMN-IZ 000.002.301.

***Paratype***: Vietnam • 1 shell; same data as for holotype; VNMN-IZ 000.002.302.

##### Additional material.

Vietnam • 1 j/b shell; same data as for holotype; VNMN-IZ 000.002.303.

##### Diagnosis.

A small *Angustopila* species with a conical-globular shell, few (ca. 3.75) whorls, normally developed spiral striation and a parietal tooth.

##### Description.

Shell small for the genus, slightly higher than wide; conical-globular; body whorl widest in standard apertural view; protoconch consists of 1.5 whorls with weak spiral striation preceding the first teleoconch whorl; teleoconch with very fine irregularly spaced, weak radial growth lines crossed by much stronger rather regularly and sparsely-spaced spiral striae (ca. 11 or 12 on body whorl from apertural view); on both ventral and dorsal surfaces of body whorl, spiral lines dominate or some radial lines are of comparable strength; whorls ca. 3.75, rounded; aperture very slightly oblique to shell axis from lateral view; umbilicus moderately wide; aperture ovate-oblong, parietal callus convex; sinulus very wide, weakly isolated due to the rather thick and deeply extending parietal tooth; peristome expanded, not reflected; parietal callus only very slightly detached from penultimate whorl; parietal tooth moderately elevated, of normal length, reaches parietal callus, perpendicular to parietal wall.

##### Measurements (in mm).

H = 0.79–0.8, D = 0.74, H/D*100 = 106.8–108.1, RUD = 26.7–29.5 (*n* = 2).

##### Differential diagnosis.

*Angustopilavandevenderi* sp. nov. is smaller than shells of the surrounding known populations of *A.fabella*, has a conical-globular (instead of a conical or concave-conical) shell, and a stronger parietal tooth. *Angustopilamargaritarion* sp. nov. is also similar to this new species in terms of shell size and shape. However, *A.vandevenderi* sp. nov. has stronger spiral striation (*A.margaritarion* sp. nov. is practically smooth, glossy), and has a lower, more slender parietal tooth (more elevated and club-shaped in *A.margaritarion* sp. nov.).

##### Etymology.

This new species is dedicated to and named after Robert Wayne Van Devender, who collected the shells.

##### Distribution.

This new species is known only from its type locality in the Cuc Phuong National Park, northern Vietnam (Fig. 27).

##### Remarks.

A juvenile shell of possible *A.fabella* was found together with the two shells of *A.vandevenderi* sp. nov. If the identification of that shell is correct, it would mean that the two species live syntopically, and are thus, distinct biological species.

**Figure 58.**
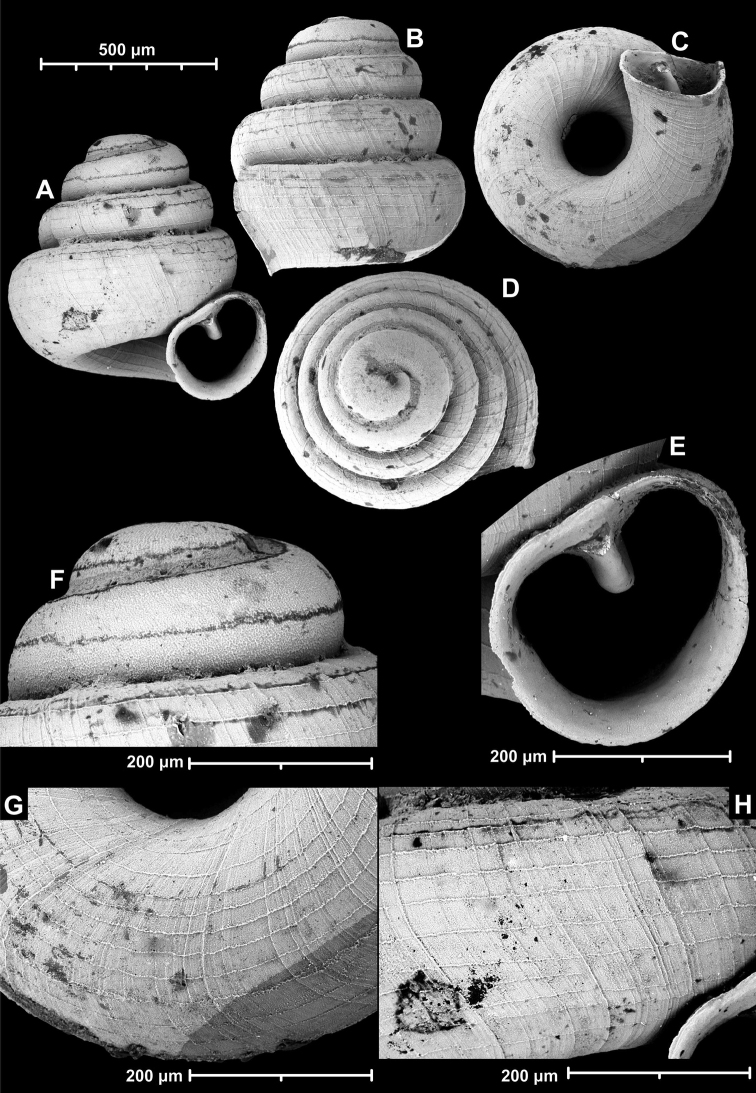
*Angustopilavandevenderi* Páll-Gergely & Jochum, sp. nov. (holotype, VNMN-IZ 000.002.301). Apertural (**A**), lateral (**B**), ventral (**C**) and apical (**D**) sides of the shell; aperture (**E**), microstructure of the protoconch (**F**), ventral (**G**) and frontal (**H**) surface of the body whorl.

#### ﻿ Angustopila vomer

Taxon classificationAnimaliaStylommatophoraGastrocoptidae

Páll-Gergely & Hunyadi sp. nov.

89B124C9-3CDB-5432-B93F-38C7B6462BCE

https://zoobank.org/72DD5DBA-EDF2-4F41-8360-B05C84634FC5

Fig. 59

##### Type material.

***Holotype***: Vietnam • 1 empty shell (H: 0.85 mm, D: 0.83 mm); Lạng Sơn Province, Hữu Lũng District, Hữu Liên, 1400 m west from Đȏng Lâm along road no. 241 (locality code: 2020/53); 21°41.91'N, 106°21.77'E; 210 m a.s.l.; 19 Feb. 2020; A. Hunyadi leg.; HNHM 105292.

***Paratypes***: Vietnam • 2 shells; same data as for holotype; coll. HA.

##### Diagnosis.

A small to medium-sized, concave-conical *Angustopila* species with a very high and long parietal tooth.

##### Description.

Shell small to medium-sized for the genus, slightly higher than wide or slightly wider than high; transparent, pale grey, concave-conical; body whorl widest from standard apertural view; protoconch consists of 1.5 whorls, with very slight spiral striation preceding the first teleoconch whorl; teleoconch ornamented by rather strong, irregularly spaced radial growth lines and equidistantly-spaced spiral striae of comparable strength or even stronger than the radial lines (ca. 10–11 on body whorl from standard apertural view); whorls 3.75, rounded or depressed-rounded; aperture strongly oblique to shell axis from lateral view; moderately wide; parietal callus attached (smeared) onto penultimate whorl; aperture subcircular-quadrate with strongly expanded peristome; parietal tooth starts in some distance from parietal callus, strongly elongated inside aperture, conspicuously elevated (extends beyond half aperture), straight or slightly curves in direction of palatal wall.

##### Differential diagnosis.

Some populations of *Angustopilafabella* are similar in overall shell shape, but all of those have a lower (less elevated) parietal tooth. See also under *A.prolixa* sp. nov.

##### Measurements (in mm).

H = 0.8–0.85, D = 0.82–0.88, H/D*100 = 90.9–103.7 (*n* = 3), RUD = 27.1–27.3 (*n* = 2).

##### Etymology.

This species is named after its elevated (high) parietal tooth resembling the vomer bone, forming part of the nasal septum of the human skull (to be used as noun in apposition).

**Figure 59.**
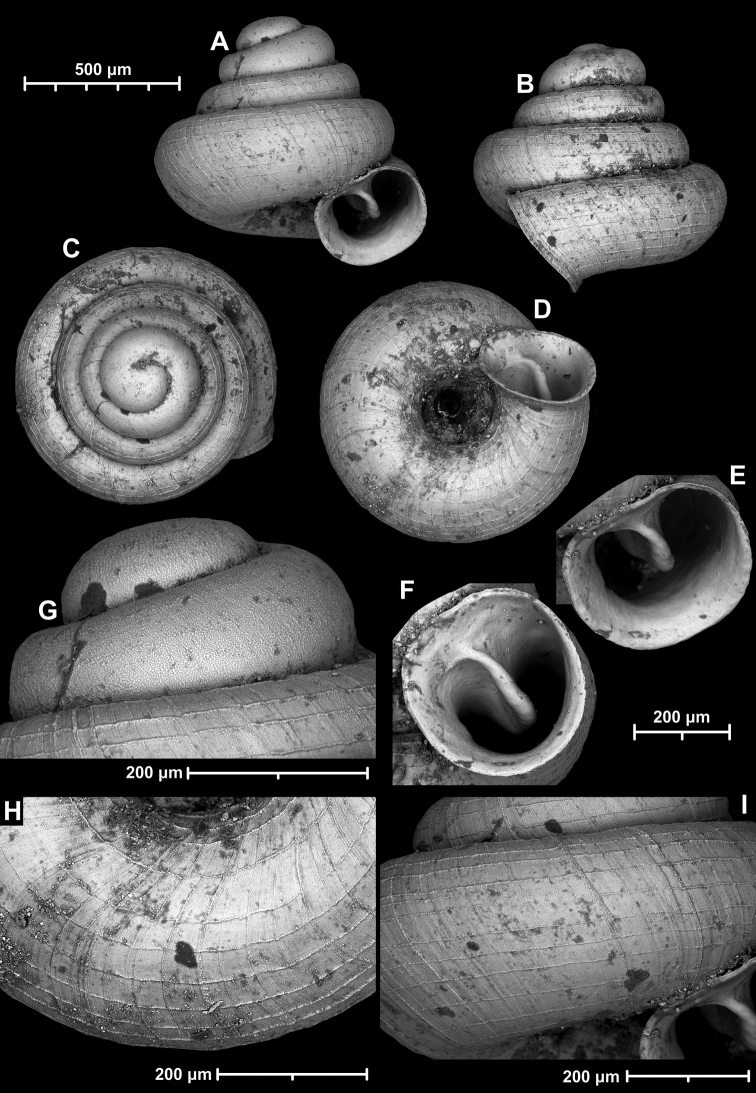
*Angustopilavomer* Páll-Gergely & Hunyadi, sp. nov. (holotype, HNHM 105292). Apertural (**A**), lateral (**B**), apical (**C**) and ventral (**D**) sides of the shell; aperture (**E, F**); sculpture on the protoconch (**G**), ventral (**H**) and frontal (**I**) surface of the body whorl.

##### Distribution.

This new species is known only from its type locality (Fig. 48).

### ﻿Species with two apertural denticles

**Remarks.***Angustopilaoccidentalis* sp. nov. may lack a palatal tooth. *Angustopilaantidomedon* sp. nov. may have an additional subcolumellar tooth.

#### ﻿ Angustopila akrodon

Taxon classificationAnimaliaStylommatophoraGastrocoptidae

Páll-Gergely & Hunyadi sp. nov.

899BFE2A-D7FD-55EB-9E4E-29850A56BE95

https://zoobank.org/432EC6FD-07A1-4E80-8DF4-B37E836443C8

Fig. 60

##### Type material.

***Holotype***: Vietnam • 1 empty shell (H: 0.88 mm, D: 0.87 mm); Thanh Hóa Province, Như Thanh District, 600 m south from Xuân Khang along road no. 45, around the cave temple (locality code: 2020/40); 19°40.35'N, 105°31.07'E; 80 m a.s.l.; 14 Feb. 2020; A. Hunyadi leg.; HNHM 105293.

***Paratypes***: Vietnam • 16 shells; same data as for holotype; coll. HA.

##### Diagnosis.

A medium-sized, concave-conical *Angustopila* species with a wide umbilicus, dense spiral striation, a prominent palatal and a highly situated upper parietal tooth.

##### Description.

Shell of normal size for the genus, higher than wide, or rarely slightly wider than high; off-white, concave-conical; body whorl widest from standard apertural view; protoconch consists of 1.25 whorls, with very slight indication of spiral striation preceding the first teleoconch whorl; teleoconch with fine, dense, irregularly spaced radial growth lines and much stronger, equidistantly-arranged dense spiral lines (ca. 16–20 on body whorl from standard apertural view); whorls 4.5, slightly shouldered; aperture oblique to shell axis from lateral view; umbilicus moderately wide; aperture pear-shaped with a parabolic sinulus caused by alignment of the parietal and upper palatal tooth; peristome expanded, not reflected; parietal callus protruding, detached from penultimate whorl; parietal tooth straight, strong but short, almost reaching peristome edge; upper palatal tooth situated very high in the aperture, points in direction of parietal tooth, blunter and wider than parietal tooth.

##### Measurements (in mm).

H = 0.88–1.04, D = 0.86–0.91, H/D*100 = 98.9–118.2 (*n* = 5), RUD = 26.4–27.3 (*n* = 2).

##### Differential diagnosis.

*Angustopilaakrodon* sp. nov. differs from the much larger *A.majuscula* sp. nov. in the concave-conical shell shape, the blunter apex, denser spiral striation and the narrower sinulus. *Angustopilaconcava* is larger, it has a more oblique aperture and a weaker parietal tooth and lacks a palatal tooth.

**Figure 60.**
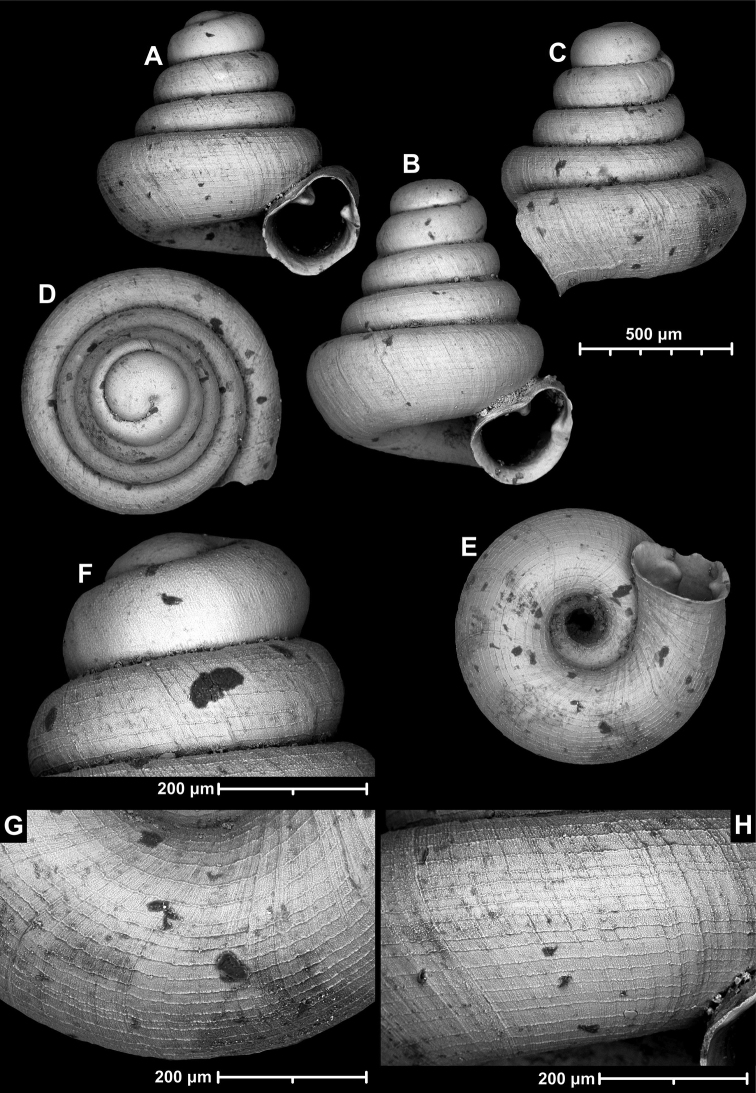
*Angustopilaakrodon* Páll-Gergely & Hunyadi, sp. nov. **A, C–H** holotype (HNHM 105293) **B** paratype. Apertural (**A, B**), lateral (**C**), apical (**D**) and ventral (**E**) sides of the shell; sculpture on the protoconch (**F**), ventral (**G**) and frontal (**H**) surface of the body whorl.

##### Etymology.

The upper parietal tooth is situated very high compared to other species with two apertural teeth (*ἄκρον* = highest, *ὀδούς* = tooth in Greek).

##### Distribution.

*Angustopilaakrodon* sp. nov. is known from the type locality only in Thanh Hóa Province of northern Vietnam (Fig. 31).

#### ﻿ Angustopila antidomedon

Taxon classificationAnimaliaStylommatophoraGastrocoptidae

Páll-Gergely & Hunyadi sp. nov.

029FBA58-E0EF-5698-BCD7-A759AB9D7294

https://zoobank.org/F3B6C9CA-F389-46AE-8C49-CA7223A0BE72

Fig. 61

##### Type material.

***Holotype***: Vietnam • 1 empty shell (H: 0.94 mm, D: 0.83 mm); Thanh Hóa Province, Như Thanh District, Hải Vân, Hang Lò Cao Kháng Chiến, vicinity of the cave (locality code: 2020/41); 19°37.08'N, 105°34.63'E; 20 m a.s.l.; 14 Feb. 2020; A. Hunyadi leg.; HNHM 105294.

***Paratypes***: Vietnam • 1 imaged + 8 other shells; same data as for holotype; coll. HA.

##### Diagnosis.

A medium-sized, conical *Angustopila* species with a narrow umbilicus, a parietal tooth and a blunt but strong palatal tooth situated at the lower part of the palatal side.

##### Description.

Shell of normal size for the genus, higher than wide; off-white, conical; body whorl widest from standard apertural view; protoconch consists of 1.25 whorls, with weak spiral striation preceding the first teleoconch whorl; teleoconch with rather strong, irregularly spaced radial growth lines and equally strong, equidistantly and sparsely-arranged spiral striae (ca. 12–19 on body whorl from standard apertural view); whorls 4.5 or slightly less, rounded or very slightly depressed-rounded in form; aperture oblique to shell axis from lateral view; umbilicus narrow; aperture subovoid or reniform with very wide, not distinctly separated sinulus; peristome expanded, not reflected; parietal callus slightly protruding, somewhat detached from penultimate whorl; parietal tooth straight, strong, normally high and long, situated inside shell at some distance from peristome edge; lower palatal tooth, blunt but strong; approximately half of the available shells has a tiny subcolumellar tooth.

##### Measurements (in mm).

H = 0.86–0.94; D = 0.82–0.88, H/D*100 = 103.6–113.3 (*n* = 4), RUD = 24.2–25.0 (*n* = 2).

##### Differential diagnosis.

*Angustopilapustulata* sp. nov. is generally larger (wider), although the size ranges slightly overlap (D of *A.antidomedon* sp. nov.: 0.82–0.88 mm, D of *A.pustulata* sp. nov.: 0.87–0.93 mm), it has a lower spire (Fig. 77A is a rare example, nearly all shells have a low spire, similar to the holotype), and its palatal tooth is much lower (weaker). Moreover, some shells of *A.antidomedon* sp. nov. possess a small columellar tooth. *Angustopilaoccidentalis* sp. nov. is larger, has a lower spire and a comparatively larger aperture with usually weaker palatal tooth. *Angustopilauvula* sp. nov. has a concave-conical shell and a stronger parietal lamella. See also under *A.bathyodon* sp. nov. and *A.gracilis* sp. nov.

**Figure 61.**
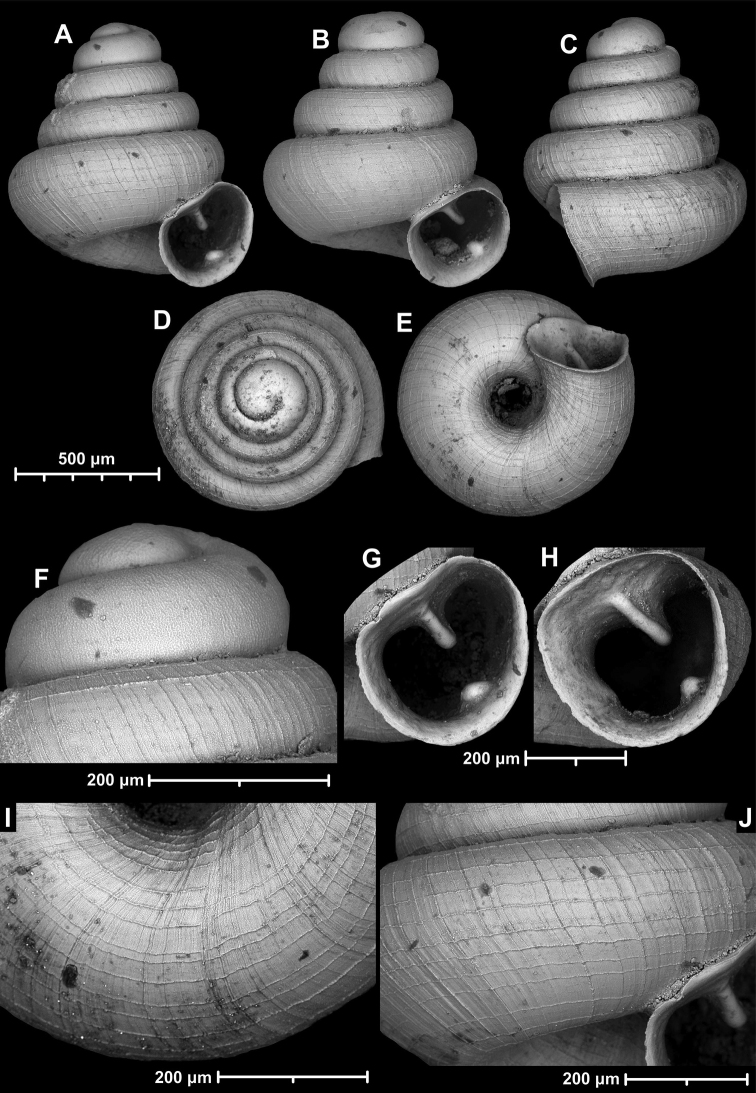
*Angustopilaantidomedon* Páll-Gergely & Hunyadi, sp. nov. **A, C–F, I, J** holotype (HNHM 105293) **B, H** paratype (specimen 2). Apertural (**A, B**), lateral (**C**), apical (**D**) and ventral (**E**) sides of the shell; sculpture on the protoconch (**F**), aperture (**G, H**); ventral (**I**) and frontal (**J**) surface of the body whorl.

##### Etymology.

From the Greek *ἀντιδομή* (= opposed) and *ὀδούς* (= tooth) referring to the position of the palatal tooth in relation to the parietal tooth.

##### Distribution.

This species is known from the type locality only (Fig. 27).

#### ﻿ Angustopila bathyodon

Taxon classificationAnimaliaStylommatophoraGastrocoptidae

Páll-Gergely & Hunyadi sp. nov.

FAB0508E-0DEF-54CA-BFF2-04D9D738AA58

https://zoobank.org/29C5CA54-04BF-43EC-9B85-689E9F252E81

Fig. 62

##### Type material.

***Holotype***: Laos • 1 empty shell (H: 0.78 mm, D: 0.82 mm); Khammouane Province, 43.4 km from Nhommalath towards Nongchan (bridge on road #12), southern edge of Ban Khamhé, right side of road (locality code 2019/108); 17°34.74'N, 105°27.52'E; 210 m a.s.l.; 30 Sep. 2019; A. Hunyadi leg.; HNHM 105295.

***Paratypes***: Laos • 34 shells; same data as for holotype; coll. HA • 3 shells; same data as for holotype; coll. JJV.

##### Additional material.

Laos • 11 j/b shells; same data as for holotype; coll. HA.

##### Diagnosis.

A small *Angustopila* species with a conical to conical-globular shell, regularly developed parietal and palatal teeth that are situated inside the shell some distance from the peristome.

##### Description.

Shell small for the genus, slightly wider than high; off-white, translucent, conical to conical-globular; body whorl widest in standard apertural view; protoconch consists of 1.5 whorls with weak spiral striation preceding the first teleoconch whorl; teleoconch with very fine irregularly spaced, very weak radial growth lines crossed by much stronger, regularly spaced spiral striae (ca. 14–18 on body whorl from standard apertural view); on both ventral and dorsal surfaces of body whorl spiral lines far more dominant than radial lines; whorls 3.75–4, rounded or very slightly shouldered; aperture oblique to shell axis from lateral view; umbilicus wide, slightly excentric; aperture reniform, sinulus wide and weakly isolated due to the weak parietal and upper palatal teeth; peristome expanded, not reflected; parietal callus not protruding, not detached from penultimate whorl; parietal tooth rather low, short, situated inside some distance from parietal callus, perpendicular to parietal side; upper palatal tooth almost as high as parietal but wider, blunter, situated inside the shell some distance from peristome edge, at middle of palatal region, just opposite of parietal tooth.

**Figure 62.**
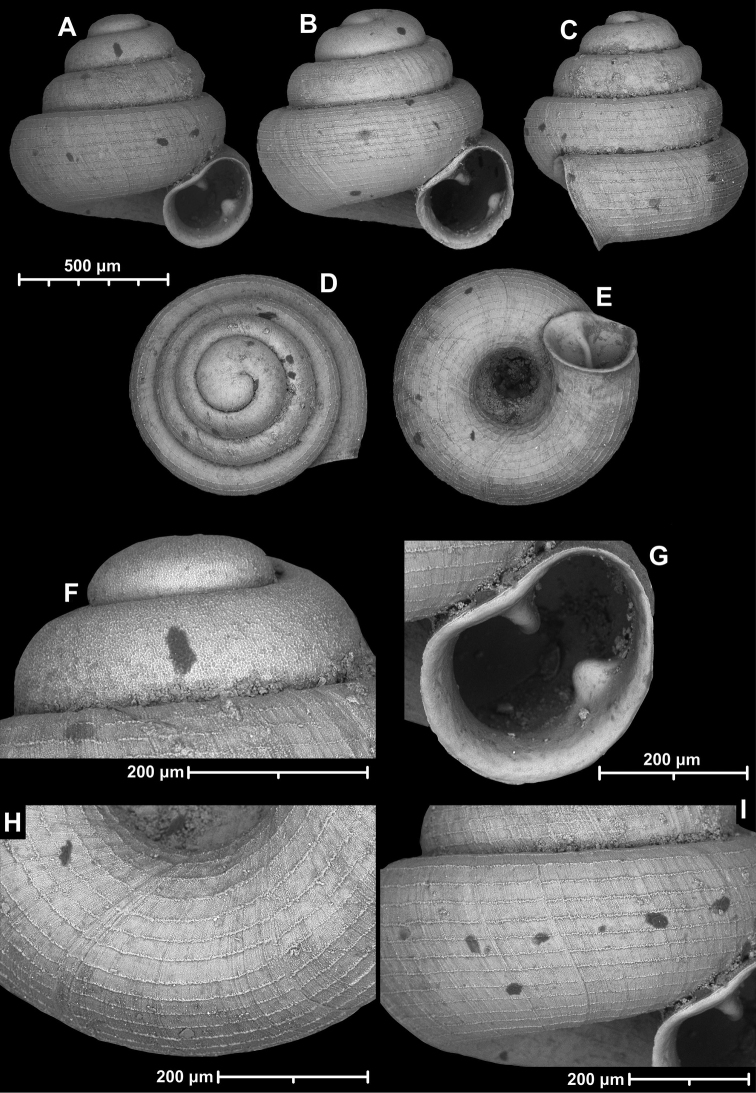
*Angustopilabathyodon* Páll-Gergely & Hunyadi, sp. nov. **A, C–I** holotype (HNHM 105295) **B** paratype. Apertural (**A, B**), lateral (**C**), apical (**D**) and ventral (**E**) sculpture on the protoconch (**F**), sides of the shell; aperture (**G**); ventral (**H**) and frontal (**I**) surface of the body whorl.

##### Measurements (in mm).

H = 0.67–0.78; D = 0.72–0.82, H/D*100 = 91.8–97.2 (*n* = 4), RUD = 30.7–31.3 (*n* = 2).

##### Differential diagnosis.

*Angustopilaantidomedon* sp. nov. has a higher spire, a more conical shell, and a lower situated parietal tooth. *Angustopilaoccidentalis* sp. nov. is much larger and has a lower situated parietal tooth. *Angustopilapustulata* sp. nov. has a concave-conical shell, it is also larger, has a weaker sculpture, and its palatal tooth is situated lower. See under *A.tweediei* sp. nov.

##### Etymology.

The specific epithet (*bathyodon*) refers to the position of the apertural barriers (*βαθύς* = deep, *ὀδούς* = tooth in Greek). Namely, they are situated more posteriorly than those of the most similar species *A.tweediei* sp. nov.

##### Distribution.

*Angustopilabathyodon* sp. nov. is known from the type locality only in Khammouane Province of Laos (Fig. 55).

#### ﻿ Angustopila bidentata

Taxon classificationAnimaliaStylommatophoraGastrocoptidae

Páll-Gergely & Jochum sp. nov.

21F2EC08-75F4-5665-9321-26BCA413C50A

https://zoobank.org/485E89E4-1CF2-48E2-B0BC-2EDE8440F451

**Figure 63.**
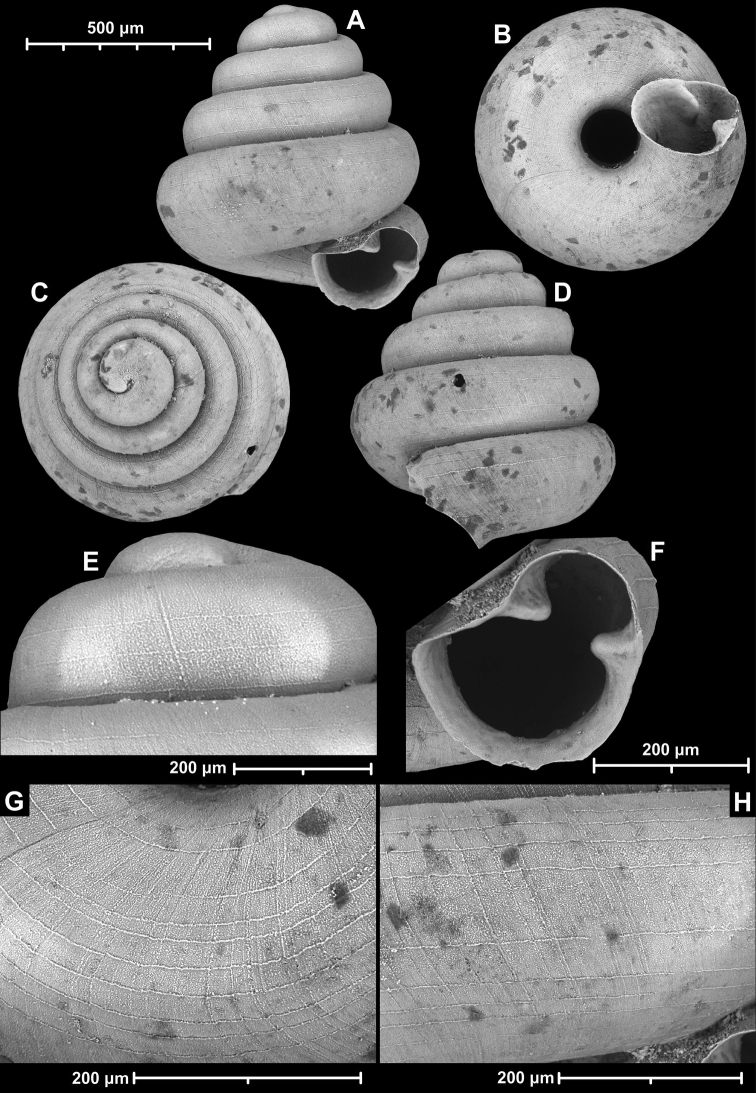
*Angustopilabidentata* Páll-Gergely & Jochum, sp. nov. (holotype, MNHN-IM-2014-6411). Apertural (**A**), ventral (**B**), apical (**C**) and lateral (**D**) sides of the shell; sculpture on the protoconch (**E**), aperture (**F**); ventral (**G**) and frontal (**H**) surface of the body whorl.

Fig. 63 , Suppl. material 3: figs S40, S41

##### Type material.

***Holotype***: Laos • 1 empty shell (H: 0.88 mm, D: 0.8 mm); South-Central Laos, Khammouane Province, ca. 35 km ENE of Thakhek (Muang Khammouane), ca. 7 km WNW of Mahaxai, on and under rocks in dry secondary forest under S exposed cliff (locality code: 4L07); 17°26.74'N, 105°08.36'E; 25 Nov. 2007; A. Abdou & I.V. Muratov leg.; MNHN-IM-2014-6411.

***Paratypes***: Laos • 3 shells; same data as for holotype; MNHN-IM-2014-6412 • 2 shells; South-Central Laos, Khammouane Province, ca. 15 km NE of Thakhek (Muang Khammouane), ca. 12.5 km SE of Ban Nase, on and under rocks in dry secondary forest near large flooded cave under W exposed cliff (locality code: 7L07); 17°30.55'N, 104°53.44'E; 130 m a.s.l.; 28 Nov. 2007; A. Abdou & I.V. Muratov leg.; MNHN-IM-2014-6410 • 12 shells (some are damaged); South-Central Laos, Khammouane Province, ca. 37 km ENE of Thakhek (Muang Khammouane), ca. 4.5 km WNW of Mahaxai, on and under rocks in dry secondary forest under E exposed cliff (locality code: 3L07); 17°25.96'N, 105°09.67'E; 150 m a.s.l.; 25 Nov. 2007; A. Abdou & I.V. Muratov leg.; MNHN-IM-2014-6409 • 10 adult shells; Khammouane Province, 17.5 km from centre of Thakhek towards Mahaxay, Tham Nang Aen (cave) (locality code: 2019/96); 17°26.65'N, 104°57.02'E; 190 m a.s.l.; 27 Sep. 2019; A. Hunyadi leg.; coll. HA • 5 shells; South-Central Laos, Khammouane Province, ca. 10.5 km E of Thakhek (Muang Khammouane), on and under rocks, cave deposits, in secondary forest under entrance and in large cave on exposed NE steep slope (locality code: 25L07); 17°24.34'N, 104°54.89'E; 160 m a.s.l.; 9 Dec. 2007; A. Abdou & I.V. Muratov leg.; MNHN-IM-2014-6408 • 1 damaged shell; Khammouane Province, Tham Nam Dôn Cave, sediment deposited by temporary side rivulet at passage entrance (locality code: JG2A); 17°33.82'N, 104°52.30'E; 160 m a.s.l.; 11 Feb. 2017; J. Grego leg.; coll. JG • 1 shell; Khammouane Province, Tham Nam Dôn Cave, Earthquake Dome, sand sediments at bank of river in cave (locality code: JG2B); 17°33.82'N, 104°52.30'E; 160 m a.s.l.; 11 Feb. 2017; J. Grego leg.; coll. JG • 1 shell; Khammouane Province, Tham Pha Inh (cave), E Thakhek, at the base of limestone rocks (Locality code: La.7); 17°27.69'N, 104°54.95'E; 180 m a.s.l.; Mar. 2010; A. Reischütz leg.; coll. RE • 1 shell; Khammouane Province, 40 km from centre of Thakhek towards Mahaxay, 2.3 km southeast from Ban Na Coc, rock wall (locality code: 2019/95); 17°25.96'N, 105°09.67'E; 155 m a.s.l.; 29 Sep. 2019; A. Hunyadi leg.; coll. HA.

##### Additional material.

Laos • 1 damaged shell with missing aperture (identity questionable, not paratype, much smaller than other shell from the same locality, see remarks); Khammouane Province, Tham Nam Dôn Cave, sediment deposited by temporary side rivulet at passage entrance (locality code: JG2A); 17°33.82'N, 104°52.30'E; 160 m a.s.l.; 11 Feb. 2017; J. Grego leg.; coll. JG • 6 j/b shells; Khammouane Province, 17.5 km from centre of Thakhek towards Mahaxay, Tham Nang Aen (cave) (locality code: 2019/96); 17°26.65'N, 104°57.02'E; 190 m a.s.l.; 27 Sep. 2019; A. Hunyadi leg.; coll. HA.

##### Diagnosis.

A small to large *Angustopila* species with a very wide sinulus, an oblique aperture, a very narrow umbilicus, and strong parietal and upper palatal teeth.

##### Description.

Shell small to large for the genus (mostly medium sized), higher than wide, or rarely slightly wider than high; pale grey, conical-globular, last or penultimate whorl widest from standard apertural view; protoconch consists of 1.5 whorls, with fine spiral striation on the entire protoconch (five spiral lines visible); teleoconch finely ornamented with irregularly spaced radial growth lines crossed by fine rows of regularly or irregularly spaced spiral striae (ca. 13–19 on body whorl from apertural view); on frontal and ventral surface of body whorl spiral and radial lines of comparable strength, or spiral ones dominant; whorls 4.5, slightly shouldered; aperture very strongly oblique to shell axis from lateral view; umbilicus, very narrow; aperture reniform, sinulus wide and strongly separated by parietal and palatal teeth; peristome slightly expanded, not reflected; mid-section comprising the parietal tooth sinuous and slightly protruding (in side view); aperture detached from the penultimate whorl forming a short, downward-directed tubus (lateral view); parietal callus strongly elevated, sharp; parietal tooth strongly developed and pointed towards the palatal tooth, which is situated high up on the palatal side; parietal and palatal teeth are directly opposite to each other.

##### Measurements (in mm).

H = 0.8–1.1, D = 0.75–0.95, H/D*100 = 98.8–115.4 (*n* = 8, from populations excluding JG2A), RUD = 18.7–25.8 (*n* = 10); H: 1.21, D = 1.09, H/D*100 = 111 (JG2A).

##### Differential diagnosis.

*Angustopilabidentata* sp. nov. can be distinguished from all other *Angustopila* species by the presence of two apertural denticles on a semi-detached tuba and the strongly oblique, wing-shaped aperture formed by the very wide sinulus and a very narrow umbilicus. The latter two traits allow the distinction of this species from congeners even at the juvenile stage. The most similar species is probably *A.dominikae*, which has an oblique, oblong, and adnate aperture, and a more globular shell. See also under *A.erawanica* sp. nov., *A.huoyani*, and *A.tamlod*.

##### Etymology.

The specific epithet *bidentata* (Latin, meaning two-teeth) refers to the two strongly developed apertural teeth of this new species.

##### Distribution.

**Figure 64.**
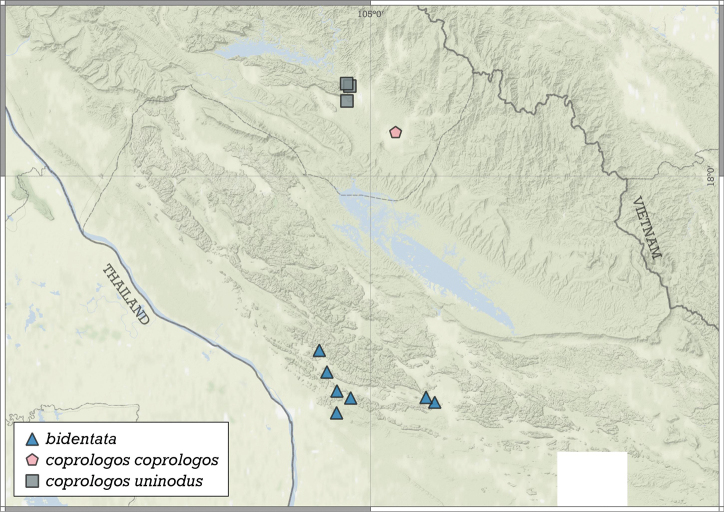
Distribution of *Angustopila* species in Laos.

*Angustopilabidentata* sp. nov. is known from Khammouane Province of Laos, in the limestone area east of Thakhek (Fig. 64).

##### Remarks.

This species shows a considerable variability in terms of shell size and some conchological characters. The populations JG2B, 3L07, 4L07, and 7L07 are indistinguishable from each other. Shells of population 25L07 are slightly larger than those in the other populations and bear a more elongated (less rounded) aperture, denser spiral striae, and show stronger radial sculpture. The single shell of the sample JG2A is very large compared to the others, and has a rounded aperture, similar to shells of the four aforementioned populations. The single shell from locality JG2A was already cracked and was subsequently damaged during imaging. However, other than shell size, no obvious morphological differences warrant distinction of that large specimen from the remaining *A.bidentata* sp. nov. shells. A single, toothless juvenile shell, similar in size to typical *A.bidentata* sp. nov., was also found in the same locality (JG2A). If that shell was an *A.bidentata* sp. nov. individual, then the large shell of the same locality represents a distinct species.

#### ﻿ Angustopila dominikae

Taxon classificationAnimaliaStylommatophoraGastrocoptidae

Páll-Gergely & Hunyadi, 2015

59AF6FCD-6DFC-5991-8EBD-4E8846E7EF57

Angustopila dominikae Páll-Gergely & Hunyadi in Páll-Gergely et al., 2015a: 34, figs 1, 12.

##### Type locality.

“China, Guangxi (广西), Hechi Shi (河池市), Bama Xian (巴马县), cliffs at the southern edge of Jiaole Cun (交乐村), 590 m, 24°7.045'N, 107°7.847'E”.

##### Diagnosis.

A medium-sized, corpulent (globular) *Angustopila* species with an elongated aperture having a parietal and an upper palatal tooth.

##### Differential diagnosis.

Differs from its morphologically closest congener, A.bidentata sp. nov., by its lower-spired globose shell and adnate oblique-oblong aperture. *Angustopilahuoyani* has a larger and more conical shell bearing more whorls, a narrower umbilicus and lacks or possesses weaker spiral striae on the entire shell. Differs from *A.steffeki* sp. nov. due to its oblique-oblong adnate aperture, two prominent apertural denticles, globose shell with tilted spire in respect to shell axis and a much narrower umbilicus. See also under *Angustopilaerawanica* sp. nov.

##### Distribution.

This species is known only from the type locality (Fig. 19).

#### ﻿ Angustopila gracilis

Taxon classificationAnimaliaStylommatophoraGastrocoptidae

Páll-Gergely & Hunyadi sp. nov.

745B12CD-0ABC-512D-A433-293226975E03

https://zoobank.org/34ADEA61-4E2E-4115-B925-D31F65AB9DAC

Fig. 65

##### Type material.

***Holotype***: Laos • 1 empty shell (H: 0.94 mm, D: 0.85 mm); Bolikhamsai Province, 15 km southeast from centre of Lak Sao, 4.5 km northeast on a side road, Phu Phako, limestone gorge (locality code: 2019/110); 18°06.55'N, 105°03.78'E; 510 m a.s.l.; 2 Oct. 2019; A. Hunyadi leg.; HNHM 105296.

***Paratypes***: Laos • 699 adult + 207 juvenile shells; same data as for the holotype; coll. HA (juvenile shells are also of characteristic shape for the species, and thus, they are also selected to be paratypes) • 10 shells, same data as for the holotype; coll. JJV.

##### Diagnosis.

A medium-sized, concave-conical *Angustopila* species with conspicuously narrow initial whorls and wide body whorl, aperture wide with a regularly developed parietal tooth, and a very weak, blister-like lower palatal tooth.

##### Description.

Shell of normal size for the genus, higher than wide or rarely slightly wider than high; off-white, concave-conical with conspicuously narrow initial whorls and wide body whorl; last whorl widest from standard apertural view; protoconch consists of 1.25 whorls, spirally striated with the exception of the first protoconch whorl; teleoconch with weak, irregular radial ribs and equidistantly, sparsely-spaced, spiral striae (ca. 12–16 on body whorl from standard apertural view); whorls 4.25–4.5, rounded; aperture oblique to shell axis in lateral view; umbilicus wide, slightly excentric; aperture nearly rounded, wide; sinulus wide, weakly separated; peristome expanded, not reflected; parietal callus not detached from penultimate whorl; parietal tooth elevated, straight, starts in some distance from peristome, rather long; lower palatal tooth tiny, blister-like.

**Figure 65.**
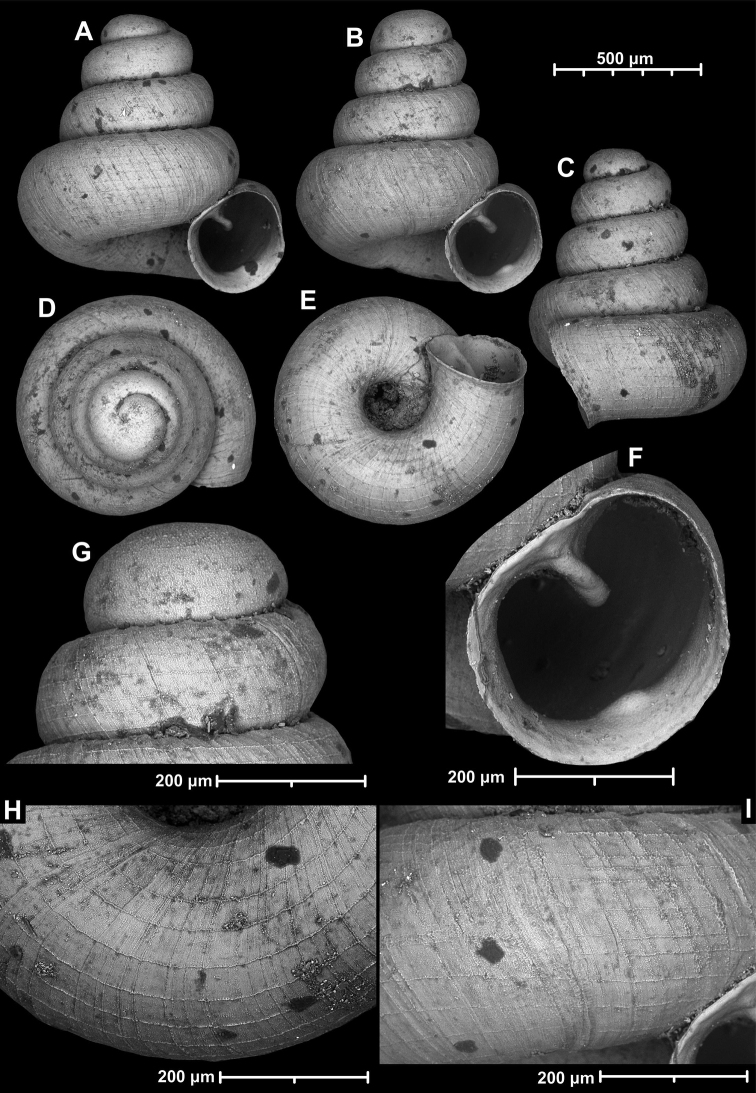
*Angustopilagracilis* Páll-Gergely & Hunyadi, sp. nov. **A** paratype **B–I** holotype (HNHM 105296). Apertural (**A, B**), lateral (**C**), apical (**D**) and ventral (**E**) sides of the shell; aperture (**F**); sculpture on the protoconch (**G**), ventral (**H**) and frontal (**I**) surface of the body whorl.

##### Measurements (in mm).

H = 0.89–0.97, D = 0.82–0.95, H/D*100 = 93.7–113.4 (*n* = 7), RUD = 26.5–31.8 (*n* = 4).

##### Differential diagnosis.

*Angustopilaantidomedon* sp. nov. and *A.pustulata* sp. nov. possess a less rounded (rather depressed-rounded) body whorl and a less rounded aperture. Moreover, none of those species have such slender initial whorls.

##### Etymology.

This species is named after the slender (Latin: *gracilis*) initial whorls.

##### Distribution.

*Angustopilagracilis* sp. nov. is known from the type locality only in Bolikhamsai Province of Laos (Fig. 31).

#### ﻿ Angustopila huoyani

Taxon classificationAnimaliaStylommatophoraGastrocoptidae

Jochum, Slapnik & Páll-Gergely, 2014

D1BB2739-1927-5908-918E-5C3033B8423B

Figs 66 , 67

Angustopila huoyani Jochum, Slapnik & Páll-Gergely in Jochum et al. 2014: 27, figs 4, 5, video 1.
Angustopila huoyani — Páll-Gergely et al. 2015a: 39, fig. 3.

##### Type locality.

“China, Hunan, Longshan (龙山县), Huoyan (火焰), Feihu Dong (飞虎洞), (29°12.53'N, 109°18.37'E, 550 m alt.)”.

##### Type material examined.

***Holotype***: China • 1 shell; Hunan, Longshan, Huoyan, Feihu Dong (“Cave of the Wild Tiger”), Kitajska; 13 Apr. 1997; Verovnik leg.; MCSMNH 50312/1.

***Paratypes***: China • 1 shell; same data as for holotype; MCSMNH 50312/2–3 • 3 shells; same data as for holotype; MCSMNH 50312/4–6 • 2 shells; same data as for holotype; MCSMNH 50312/7–9.

##### Additional material examined.

China • 65 complete shells; same data as for holotype; MCSMNH 50312 (separated into 4 vials based on morphological groups, see remarks) • 48 broken shells (in 2 vials); same data as for holotype; MCSMNH 50312.

##### Diagnosis.

A medium-sized to large, conical-globular *Angustopila* species with a very narrow umbilicus, a parietal tooth, and an upper palatal tooth.

##### Differential diagnosis.

The morphologically closest congener with two apertural denticles is *A.tamlod* known from Thailand; however, *Angustopilahuoyani* is tightly coiled, has a more conical shell, bears more pronounced apertural dentition, and has stronger and denser spiral striation. The shell of *A.dominikae* is more compact and is 2/3 the size of *A.huoyani*. Though also bearing two denticles, *A.uvula* sp. nov. is comparatively loosely coiled, has larger whorls, a bulbous protoconch and a slightly downwards directed tuba and bears a very prominent parietal denticle opposed by a weak palatal one. *Angustopilabidentata* sp. nov. has a narrower umbilicus and more oblique aperture. *Angustopilaerawanica* sp. nov. has a more horizontally oriented aperture that is impressed at the parietal lamella.

##### Measurements (in mm).

H = 0.96–1.31; D = 0.82–1.1, H/D*100 = 109.1–143.6 (*n* = 67), RUD = 14.3–18.8 (*n* = 6).

##### Distribution.

This species is known from two caves, one located in Guangxi, and the other in Hunan, China (Fig. 19).

##### Remarks.

The original description of the type material describes the holotype and eight paratypes. However, results in Jochum et al. (2014: table 2) were based on 30 specimens. For this revision, and because we intended to re-investigate this species and compare it with other congeners, we borrowed the type material of *A.huoyani* from its home museum in Ljubljana. The examination revealed that in addition to the holotype and the paratypes, there were 65 shells (previously unknown to Jochum et al. 2014) in perfect condition and several others that were somewhat damaged. The 65 shells showed extraordinary variation in terms of shell size and shape, while the positions of the apertural teeth and the sculpture did not vary. At first sight, the shells could be separated into four more or less clearly distinguishable groups as follows: (1) shell large, rounded, aperture ovoid, whorls: 4.75–5.5 (mean: 5.15); (2) shell large, with many whorls, aperture depressed (narrow), whorls: 5.5–6.5 (mean: 5.89) (3) shell very slender, aperture ovoid, whorls: 5–6 (mean: 5.47); and (4) shell small, rounded, aperture ovoid, whorls: 4.75–5.5 (mean: 5.26). After we measured all shells, it became evident that none of these groups are distinct based on shell measurements alone (see Fig. 67). Although the grouping of the shells into morphological groups was possible using a stereo microscope, these do not likely belong to a biologically distinct species. It is, however, possible that the soil sample containing all *A.huoyani* shells originated from multiple subpopulations living under different environmental conditions. Thus, the extreme variability we observed is explained by phenotypic plasticity.

**Figure 66.**
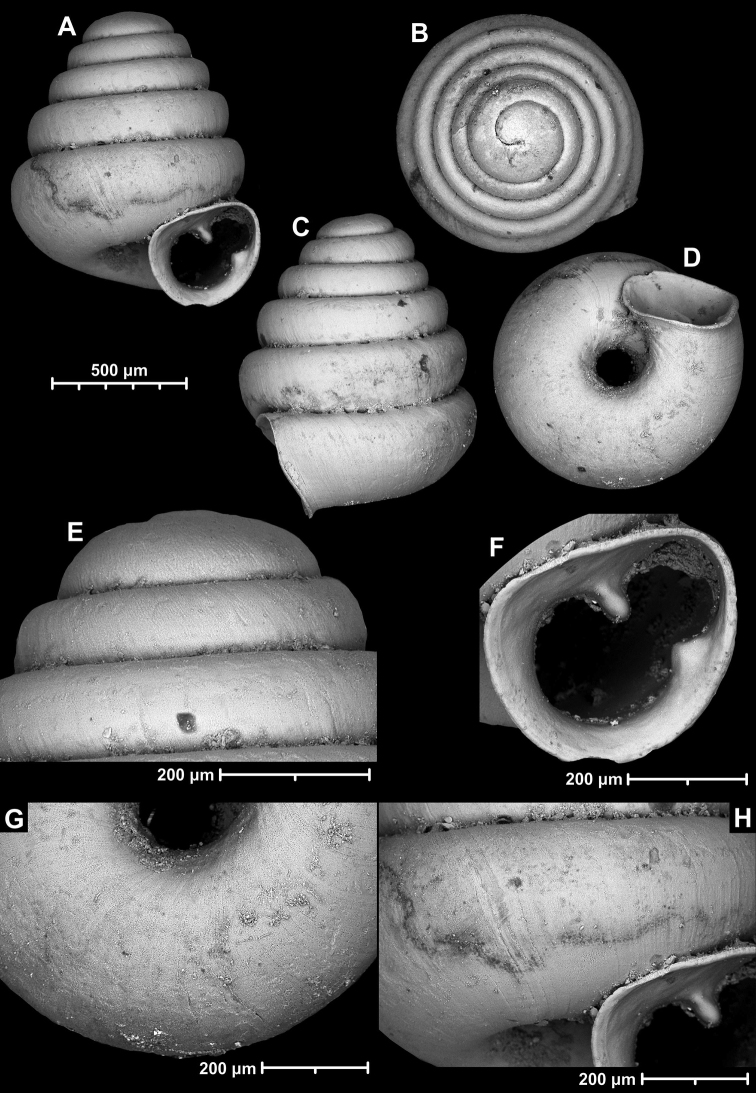
*Angustopilahuoyani* Jochum, Slapnik & Páll-Gergely, 2014 (holotype, MCSMNH 50312/1). Apertural (**A**), apical (**B**), lateral (**C**) and ventral (**D**) sides of the shell; sculpture on the protoconch (**E**), aperture (**F**); ventral (**G**) and frontal (**H**) surface of the body whorl.

**Figure 67.**
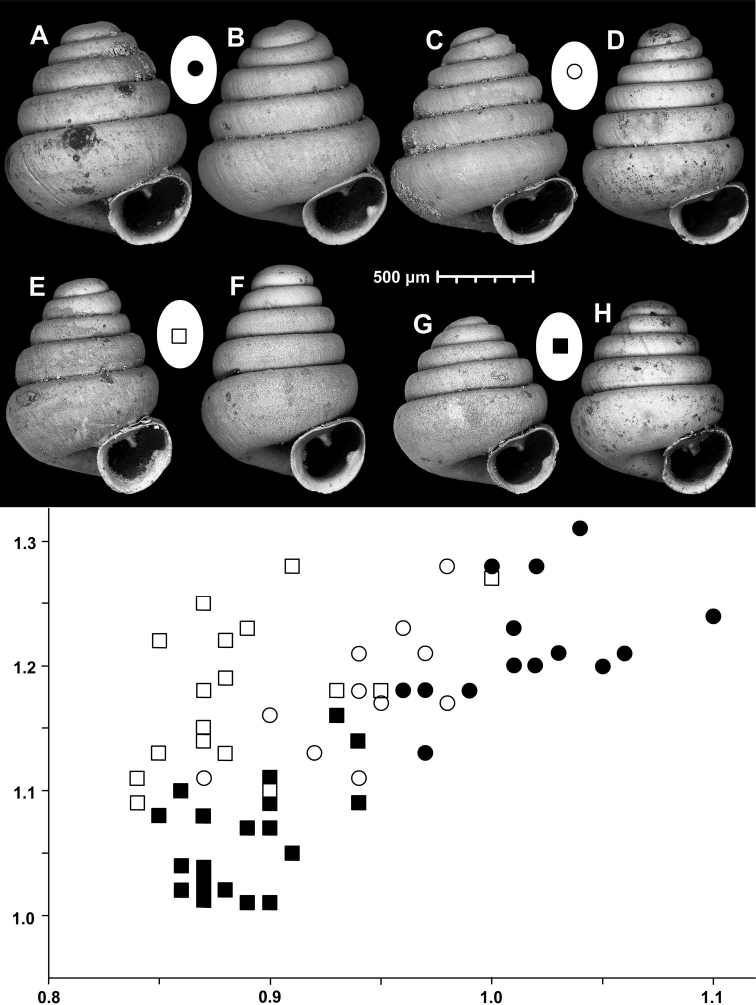
Conchological variability (upper plate) and Shell diameter (x axis, in mm) vs. shell height (y axis, in mm) (lower diagram) of *Angustopilahuoyani* Jochum, Slapnik & Páll-Gergely, 2014. Group 1 (**A, B**), Group 2 (**C, D**), Group 3 (**E, F**), Group 4 (**G, H**). Group 1 (dot), Group 2 (circle), Group 3 (empty square), Group 4 (filled square).

Measurements of the holotype (H: 1.14 mm, D: 0.94 mm) differ from those published in the original description (H: 1.09 mm, D: 0.87 mm).

#### ﻿ Angustopila majuscula

Taxon classificationAnimaliaStylommatophoraGastrocoptidae

Páll-Gergely & Hunyadi sp. nov.

760E26AB-618D-59AE-A9A4-F15E56FF9ED3

https://zoobank.org/F3393231-F280-4A63-BEDB-E1A28AA956D7

Figs 68 , 69

##### Type material.

***Holotype***: Thailand • 1 empty shell (H: 1.21 mm, D: 1.24 mm); Chiang Rai Province, 6 km S of Chiang Khian, toward Pa Ngae, Wat Phra That Charui (locality code: 2015/20); 19°34.41'N, 99°59.19'E; 420 m a.s.l.; 13 Feb. 2015; A. Hunyadi leg.; CUMZ 7438.

***Paratypes***: Thailand • 4 shells; same data as for holotype; coll. HA.

##### Additional material.

Thailand • 3 j/b shells; same data as for holotype; coll. HA • 1 imaged shell; Chiang Rai Province, Wat Doi Khong Khao Meditation Centre, 8 km NW of Chiang Rai, clay at the entrance of the cave (locality code: Th.6); 19°54.75'N, 99°46.60'E (approximate GPS coordinates); Nov. 2007; A. Reischütz leg.; HNHM 103482 • 4 shells + 13 j/b shells; same data as for preceding; coll. RE.

##### Diagnosis.

A large, conical *Angustopila* species with a wide umbilicus, a prominent palatal and a highly placed parietal tooth pointing towards the palatal.

##### Description.

Shell large for the genus, higher than wide, or rarely slightly wider than high; off-white, conical; body whorl widest from apertural view; protoconch consists of 1.5 whorls, with very slight indication of spiral striation preceding the first teleoconch whorl; teleoconch with fine, dense, irregularly spaced radial growth lines and somewhat stronger, equidistantly and sparsely-arranged spiral lines (ca. 12–17 on body whorl from standard apertural view); whorls 5, rounded or slightly shouldered; aperture oblique to shell axis from lateral view; umbilicus wide; aperture pear-shaped with distinct sinulus caused by the parietal and palatal teeth; peristome expanded, not reflected; parietal callus protruding, detached from penultimate whorl; parietal tooth oblique to parietal callus, strong but short, almost reaching peristome edge; upper palatal tooth points in direction of parietal tooth, blunter and wider than parietal tooth.

##### Measurements (in mm).

H = 1.21–1.31, D = 1.13–1.24, H/D*100 = 97.6–114.2 (*n* = 6), RUD = 33.3–36.3 (*n* = 4).

##### Differential diagnosis.

*Angustopilamajuscula* sp. nov. mostly resembles species classified as *Hypselostoma* (e.g., *H.socialis*, *H.lacrima*, etc.) by the relatively large shell, the parietal and palatal teeth that point towards each other, and the wide umbilicus. However, those “*Hypselostoma*” species possess much denser spiral striation and their apertural dentition is more developed. *Angustopilamajuscula* sp. nov. can be easily distinguished from its congeners by the arrangement of the teeth, the larger size, and the wide umbilicus. *Angustopilaconcava* lacks a palatal tooth, has a smaller parietal tooth, a concave-conical shell and a much more oblique aperture than *Angustopilamajuscula* sp. nov. See also under *A.akrodon* sp. nov., *A.parallela* sp. nov., and *A.tamlod*.

##### Etymology.

The name *majuscula* (somewhat larger in Latin) refers to the size of this species, which is the largest amongst all its congeners, but still a tiny land snail species.

##### Distribution.

This species is known from two nearby localities in Thailand’s Chiang Rai Province (Fig. 48).

##### Remarks.

The shells of sample Th.6 agree with those of the type series considering the most important characters (exactly conical shell, wide umbilicus, presence of two teeth). However, the parietal tooth of the former samples is much weaker than that in the typical shells. Therefore, we do not designate them as paratypes, but consider them conspecific.

This is the largest *Angustopila* species. At first sight it seemed to be a member of *Hypselostoma*, but that genus is characterised by denser spiral striation and an oblong-ovate apertural form.

**Figure 68.**
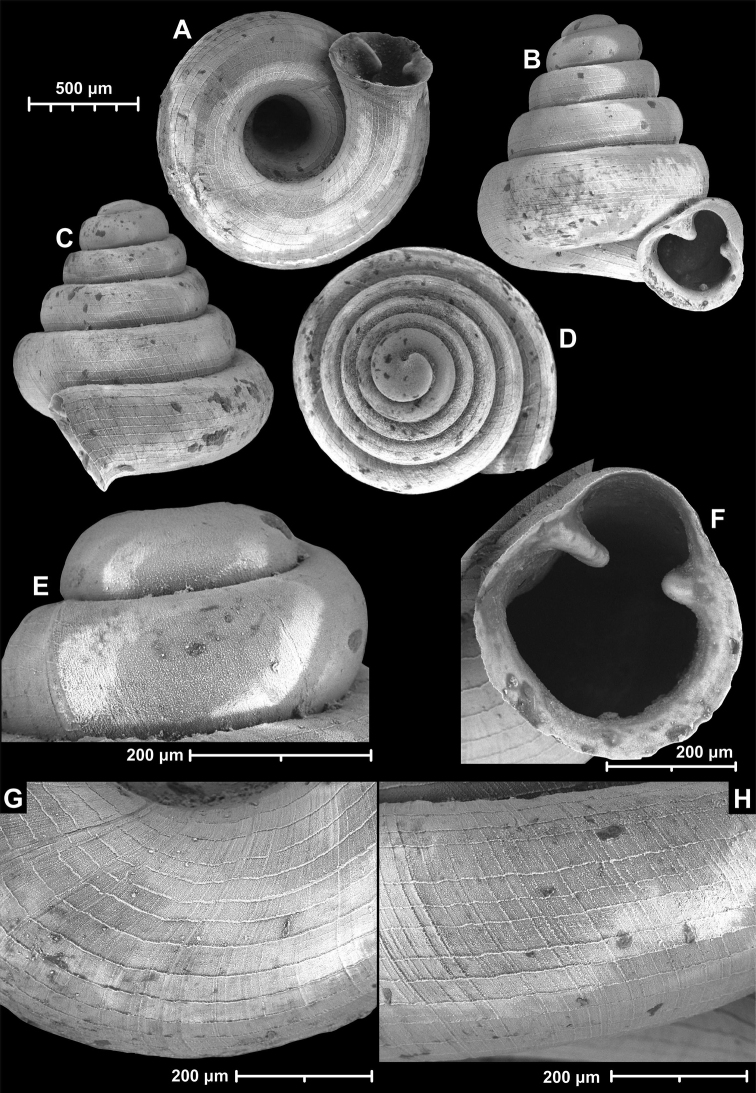
*Angustopilamajuscula* Páll-Gergely & Hunyadi, sp. nov. (holotype, CUMZ 7438). Ventral (**A**), apertural (**B**), lateral (**C**), and apical (**D**) sides of the shell; sculpture on the protoconch (**E**), aperture (**F**); ventral (**G**) and frontal (**H**) surface of the body whorl.

**Figure 69.**
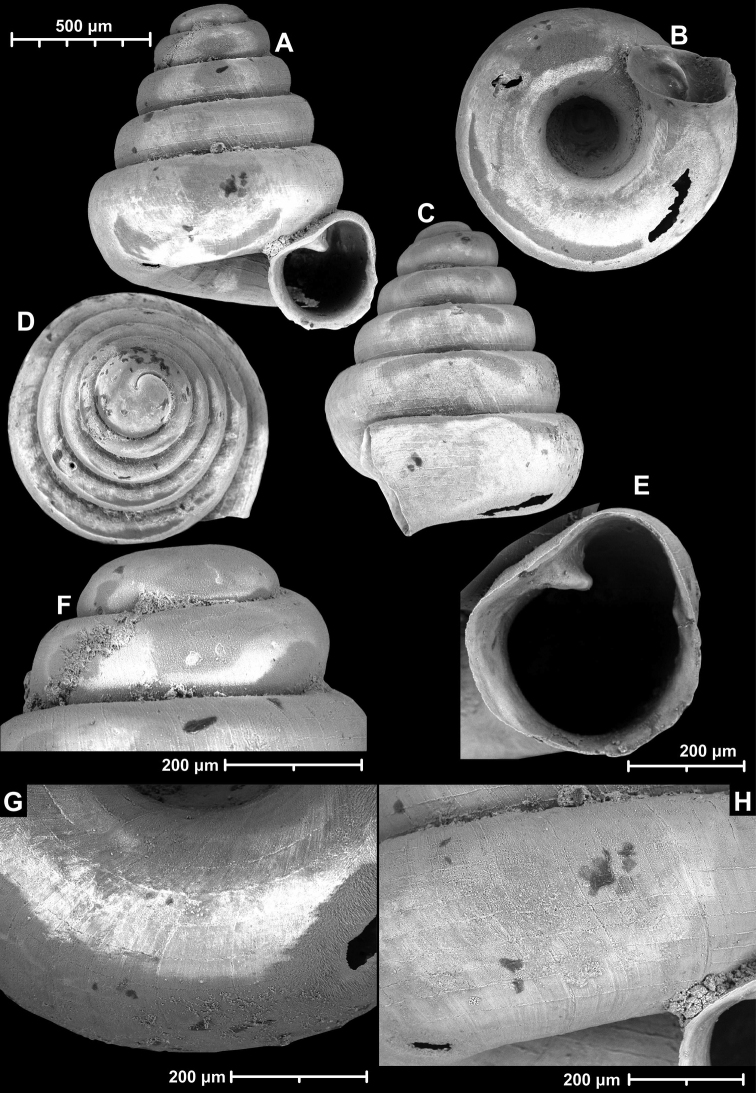
*Angustopilamajuscula* Páll-Gergely & Hunyadi, sp. nov., Th.6 (HNHM 103482). Apertural (**A**), ventral (**B**), apical (**C**) and lateral (**D**), sides of the shell; aperture (**E**); sculpture on the protoconch (**F**); ventral (**G**) and frontal (**H**) surface of the body whorl.

#### ﻿ Angustopila occidentalis

Taxon classificationAnimaliaStylommatophoraGastrocoptidae

Páll-Gergely & Hunyadi sp. nov.

6B0E9DA1-EF7A-5DA1-840E-900789C45F8A

https://zoobank.org/CA3710BC-8597-4662-92FF-5A6E16B2F8AE

Figs 70 , 71 , 72

##### Type material.

***Holotype***: Myanmar • 1 empty shell (H: 0.97 mm, D: 0.93 mm); Shan State, ca. 6 km east from Hsihseng centre, right side of rd. + 400 m on unpaved rd., limestone hill (locality code: 2018/40); 20°07.98'N, 97°18.15'E; 1010 m a.s.l.; 7 Oct. 2018; A. Hunyadi, K. Okubo & J.U. Otani leg.; HNHM 103483.

***Paratypes***: Myanmar • 18 shells; same data as for holotype; coll. HA • 17 shells; Shan State, 7.4 km from the centre of Hoponh towards Namsang along road no. 4, ca. 5 km north, Parpant Cave (locality code: 2018/36); 20°50.96'N, 97°14.26'E; 1170 m a.s.l.; 6 Oct. 2018; A. Hunyadi, K. Okubo & J.U. Otani leg.; coll. HA • 2 figured shells + 81 shells; Shan State, Pinlaung centre N 7.5 km, Tar Kge, near „Big Bang Cave” (locality codes: 2018/32 and 20181004D); 20°10.27'N, 96°47.44'E; 1540 m a.s.l.; 4 Oct. 2018; A. Hunyadi, K. Okubo & J.U. Otani leg.; coll. HA • 3 shells; Shan State, Hopong, Hopong Spring and cave (locality code: JG/2019/2); 20°49.05'N, 97°13.49'E; 2 Feb. 2019; J. Grego leg.; coll. JG • 5 shells; Kayah State, Hpruso District, Maw Thi Do Village, entrance of Phruno River Cave (locality code: JG/2019/102); 19°22.74'N, 97°02.57'E; 12 Dec. 2019; J. Grego leg.; coll. JG • 59 shells; Kayah State, Hpruso District, Hoyar village, Kyar Yin Cave (locality code: JG/2019/104a); 19°18.10'N, 96°56.40'E; 1100 m a.s.l.; 13 Dec. 2019; Mário Olšavský leg.; coll. JG.

##### Additional material.

Myanmar • 14 j/b shells; same data as for holotype; coll. HA • 1 juvenile shell (figured with insect in aperture) + 21 j/b shells; Shan State, 7.4 km from the centre of Hoponh towards Namsang along road no. 4, ca. 5 km north, Parpant Cave (locality code: 2018/36); 20°50.96'N, 97°14.26'E; 1170 m a.s.l.; 6 Oct. 2018; A. Hunyadi, K. Okubo & J.U. Otani leg.; coll. HA • 25 j/b shells; Shan State, Pinlaung centre N 7.5 km, Tar Kge, near „Big Bang Cave” (locality codes: 2018/32 and 20181004D); 20°10.27'N, 96°47.44'E; 1540 m a.s.l.; 4 Oct. 2018; A. Hunyadi, K. Okubo & J.U. Otani leg.; coll. HA.

Thailand • 1 figured shell; Chiang Mai Province, northeastern part of Doi Chiang Dao, Wat Tham Chiang Dao northwest 2 km (locality code: 2015/6); 19°24.02'N, 98°54.68'E; 835 m a.s.l.; 7 Feb. 2015; A. Hunyadi leg.; HNHM 100182.

##### Diagnosis.

A medium-sized *Angustopila* species with a relatively low conical shell, a strong parietal tooth and a weak lower palatal tooth (can be absent), and a moderately wide umbilicus.

##### Description.

Shell of normal size for the genus, slightly higher than wide or slightly wider than high; pale grey, translucent, conical; body whorl widest in standard apertural view; protoconch consists of 1.5 whorls with dense spiral striation on the entire protoconch; teleoconch finely ornamented with irregularly spaced, weak radial growth lines crossed by fine rows of regularly-spaced spiral striae (ca. 17–20 on body whorl from standard apertural view); on both ventral and dorsal surfaces of body whorl spiral lines dominant or spiral and radial lines are of comparable strength; whorls 4–4.25, rounded; aperture oblique to shell axis from lateral view; umbilicus moderately narrow; aperture ovoid or slightly reniform, sinulus wide; peristome expanded, not reflected; parietal callus not conspicuous, somewhat detached from penultimate whorl (aperture not adnate); parietal tooth well-developed, perpendicular to parietal wall; palatal side usually with a blunt lower palatal denticle (often absent, or a second one might be present), which is situated some distance inside from peristome edge.

##### Measurements (in mm).

H = 0.91–1.03, D = 0.89–1.06, H/D*100 = 88.3–108.5 (Myanmar, *n* = 16), RUD = 22.8–26.0 (*n* = 6); H = 1.02, D = 0.89, H/D*100 = 113.5, RUD = 21.9 (Thailand, *n* = 1).

##### Differential diagnosis.

*Angustopilaconcava* has a concave-conical shell, a wider umbilicus, a smaller parietal tooth, a more strongly oblique aperture, its parietal callus is more detached from the penultimate whorl and lacks a palatal tooth compared to this new species. See also under *A.antidomedon* sp. nov., *A.bathyodon* sp. nov., and *A.uvula* sp. nov.

##### Etymology.

Named after its geographic distribution compared to other *Angustopila* species (this is one of the westernmost occurring *Angustopila* species).

##### Distribution.

This species is known from one locality in Thailand’s Chiang Mai Province, and five localities from Myanmar’s Shan and Kayah States (Fig. 19).

##### Remarks.

The single shell from Thailand has a higher spire, and a more conical shell than those from Myanmar, and possesses an additional palatal denticle next to the “usual” lower palatal tooth. Thus, additional specimens are needed to infer the intraspecific diversity of that population, and to understand if it is conspecific with *A.occidentalis* sp. nov. populations of Myanmar. The shells from the populations from Myanmar show no notable conchological variation.

**Figure 70.**
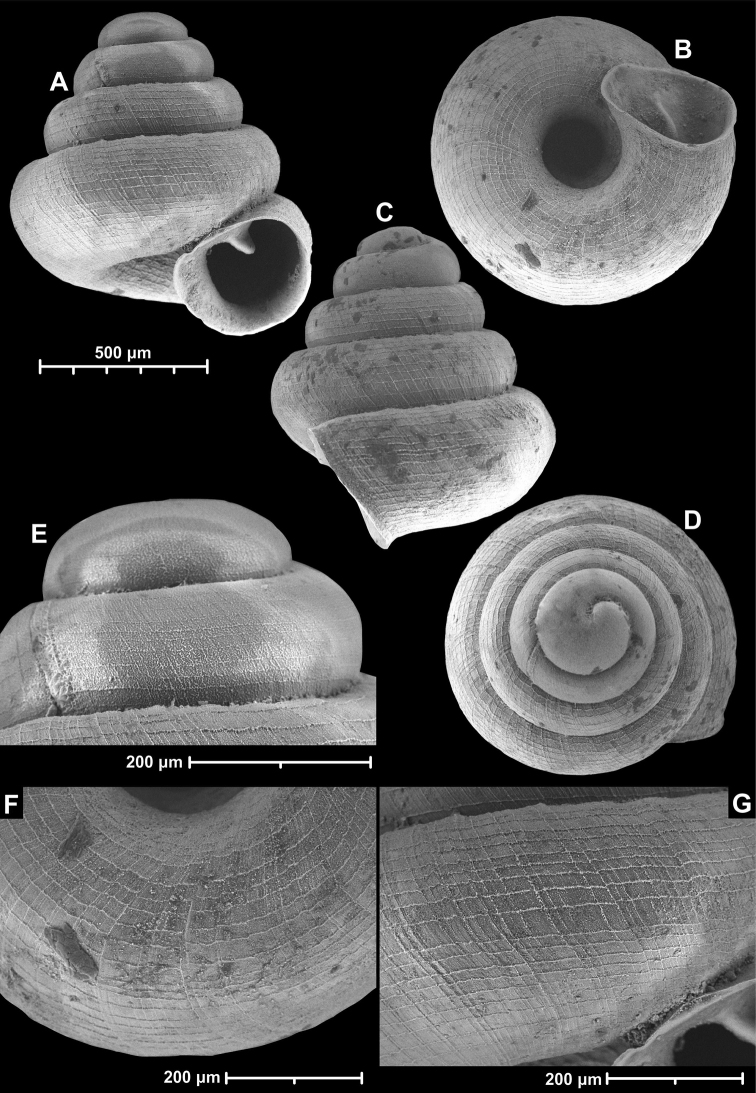
*Angustopilaoccidentalis* Páll-Gergely & Hunyadi, sp. nov. (holotype, HNHM 103483). Apertural (**A**), ventral (**B**), lateral (**C**) and apical (**D**) sides of the shell; sculpture on the protoconch (**E**), ventral (**F**) and frontal (**G**) surface of the body whorl.

**Figure 71.**
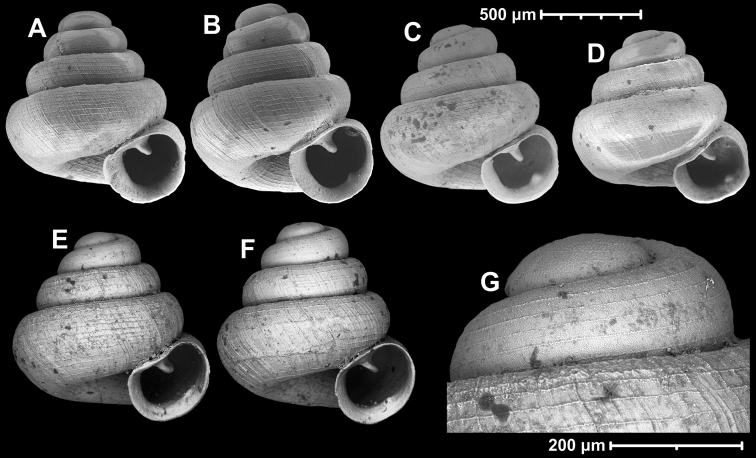
Variability of *Angustopilaoccidentalis* Páll-Gergely & Hunyadi, sp. nov. **A–D** 2018/40, (**A** holotype **B–D** paratypes) **E–G** 2018/32 (**E** and **G** specimen 1 **F** specimen 2).

**Figure 72.**
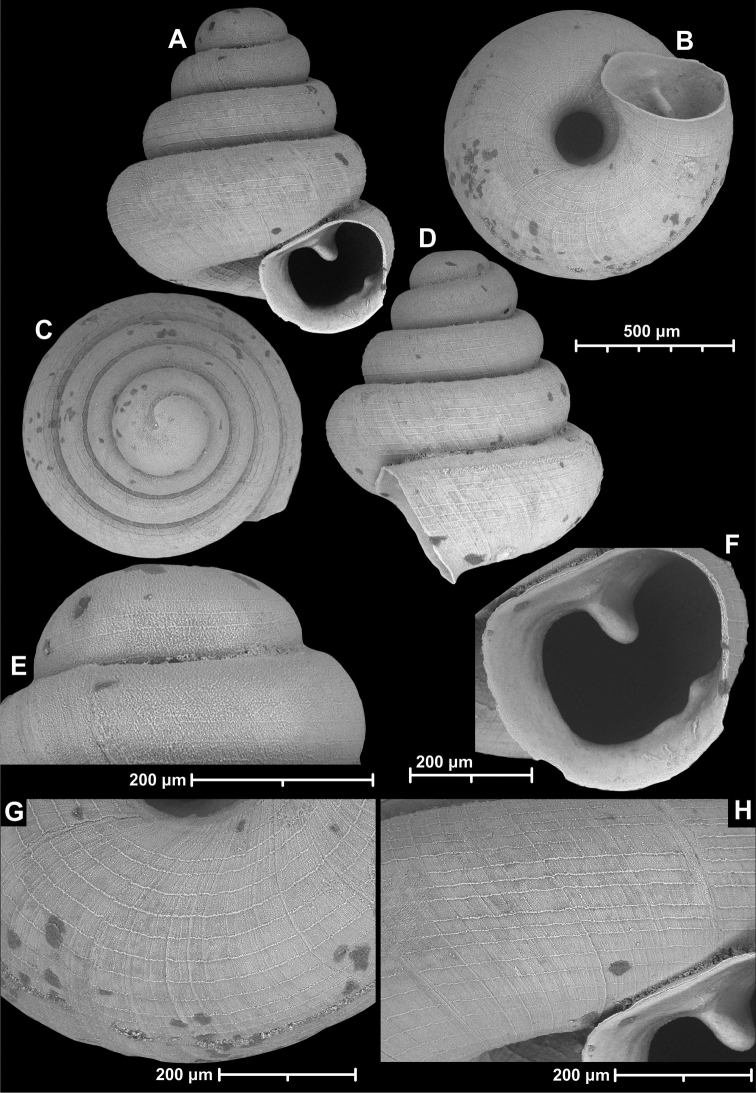
Angustopilacf.occidentalis Páll-Gergely & Hunyadi, sp. nov., Chiang Dao. Apertural (**A**), ventral (**B**), apical (**C**) and lateral (**D**) sides of the shell; sculpture on the protoconch (**E**); aperture (**F**); ventral (**G**) and frontal (**H**) surface of the body whorl.

#### ﻿ Angustopila pallgergelyi

Taxon classificationAnimaliaStylommatophoraGastrocoptidae

Dumrongrojwattana, Chuenit & Wongkamhaeng, 2021

0E915ADC-7C6F-52B6-8FCD-721499D9C049

Angustopila pallgergelyi Dumrongrojwattana, Chuenit & Wongkamhaeng, 2021: 104, figs 2, 3A, B.

##### Type locality.

“Thailand, Sa Kaeo Province, Klong Haad District, Tham Phet Pho Thong (Cave), an isolated limestone hill; 13°24'49.4"N 102°19'38.0"E; 280 m a.s.l.”

##### Diagnosis.

A small, depressed-globular *Angustopila* species with domed dorsal side, a kidney-shaped aperture with a slender sinulus, strong parietal and upper parietal tooth.

##### Differential diagnosis.

See under *A.somsaki* sp. nov. and *A.steffeki* sp. nov.

##### Distribution.

This species is known only from the type locality (Fig. 19).

#### ﻿ Angustopila papaver

Taxon classificationAnimaliaStylommatophoraGastrocoptidae

Páll-Gergely & Hunyadi sp. nov.

94F9FF48-9F54-5B33-9FA8-3931DBBA34AC

https://zoobank.org/31264F15-F05F-4C41-AA05-6B6C13D46B12

Fig. 73

##### Type material.

***Holotype***: Vietnam • 1 empty shell (H: 0.66 mm, D: 0.66 mm); Thanh Hóa Province, Như Thanh District, Hải Vân, Hang Lò Cao Kháng Chiến, vicinity of the cave (locality code: 2020/41); 19°37.08'N, 105°34.63'E; 20 m a.s.l.; 14 Feb. 2020; A. Hunyadi leg.; HNHM 105297.

***Paratypes***: Vietnam • 1 figured shell + 39 shells + 3 live-collected specimens covered in mud; same data as for holotype; coll. HA.

##### Diagnosis.

A small *Angustopila* species with a conical to conical-globular shell, a shouldered body whorl, a strong parietal tooth reaching the peristome edge, and an upper palatal tooth of ca. 1/2 size.

##### Description.

Shell small for the genus, shell slightly higher than wide or rarely as high as wide; off-white, translucent when fresh, conical to conical-globular; body whorl widest in apertural view; protoconch consists of 1–1.25 whorls with spiral striation preceding the first teleoconch whorl; teleoconch with very weak, regularly spaced radial ribs crossed by stronger spiral striae (ca. 12–15 on body whorl from apertural view); whorls ca. 3.5, slightly shouldered, “pushed” from baso-lateral direction from apertural view; aperture very slightly oblique to shell axis from lateral view; umbilicus moderately wide; aperture pear-shaped, sinulus narrow and strongly isolated due to the near joining of the strong parietal and upper palatal teeth; peristome expanded, not reflected; parietal callus slightly protruding, slightly detached from penultimate whorl (aperture not adnate); parietal tooth elevated, strong, short, reaches parietal callus, slightly curved in direction of palatal tooth; upper palatal tooth ca. 1/2 as high as parietal tooth, situated near peristome edge, just opposite of parietal tooth.

##### Measurements (in mm).

H = 0.66–0.75, D = 0.64–0.75, H/D*100 = 100–112.5 (*n* = 7), RUD = 26.6–29.2 (*n* = 3).

##### Differential diagnosis.

Similar only to *A.coprologos* and *A.psammion* in size. This new species can be distinguished from all congeners based on the combination of the very small, conical to conical-globular shell and the two apertural denticles.

##### Etymology.

The tiny shells of this species with approximately equal shell height and width resemble poppy (*Papaver*) seeds. The specific epithet is to be used as a noun in apposition.

**Figure 73.**
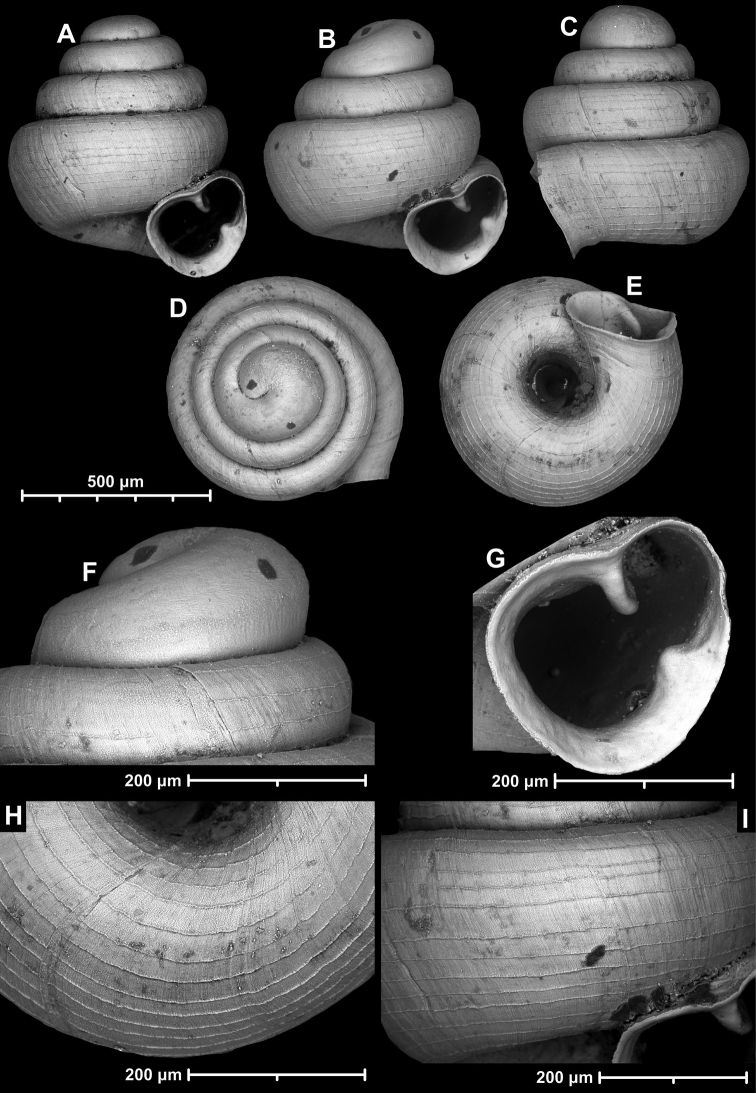
*Angustopilapapaver* Páll-Gergely & Hunyadi, sp. nov. **A, C–I** holotype (HNHM 105297) **B** paratype. Apertural (**A, B**), lateral (**C**), apical (**D**) and ventral (**E**) sides of the shell; sculpture on the protoconch (**F**); aperture (**G**); ventral (**H**) and frontal (**I**) surface of the body whorl.

##### Distribution.

*Angustopilapapaver* sp. nov. is known from the type locality only in Thanh Hóa Province of northern Vietnam (Fig. 48).

#### ﻿ Angustopila parallela

Taxon classificationAnimaliaStylommatophoraGastrocoptidae

Páll-Gergely & Hunyadi sp. nov.

60AD56B2-B2AC-5029-9E21-14539DBDEE9B

https://zoobank.org/8A123D0E-0E19-4834-A72C-EF96DD247846

Figs 74 , 75 , 76

##### Type material.

***Holotype***: Vietnam • 1 empty shell (H: 0.97 mm, D: 1.08 mm); Lạng Sơn Province, Tam Thanh, Núi Vọng Phu, Đền Chùa Thượng Ngàn (locality code: 2020/57); 21°51.21'N, 106°44.91'E; 250 m a.s.l.; 21 Feb. 2020; A. Hunyadi leg.; HNHM 105298.

***Paratypes***: Vietnam • 58 shells; same data as for holotype; coll. HA • 569 shells; Lạng Sơn Province, vicinity of Chùa Tam Thanh (locality code: 2020/58); 21°51.35'N, 106°44.81'E; 265 m a.s.l.; 21 Feb. 2020; A. Hunyadi leg.; coll. HA • 10 shells; same data as for preceding; coll. JJV.

##### Additional material.

Vietnam • 7 j/b shells; same data as for holotype; coll. HA • 115 j/b shells; Lạng Sơn Province, vicinity of Chùa Tam Thanh (locality code: 2020/58); 21°51.35'N, 106°44.81'E; 265 m a.s.l.; 21 Feb. 2020; A. Hunyadi leg.; coll. HA • 1 figured shell (Fig. 76); Thanh Hóa Province, Như Thanh District, Hải Vân, Hang Lò Cao Kháng Chiến, vicinity of the cave (locality code: 2020/41); 19°37.08'N, 105°34.63'E; 20 m a.s.l.; 14 Feb. 2020; A. Hunyadi leg.; HNHM 105299 • 7 shells; same data as for preceding; coll. HA.

**Figure 74.**
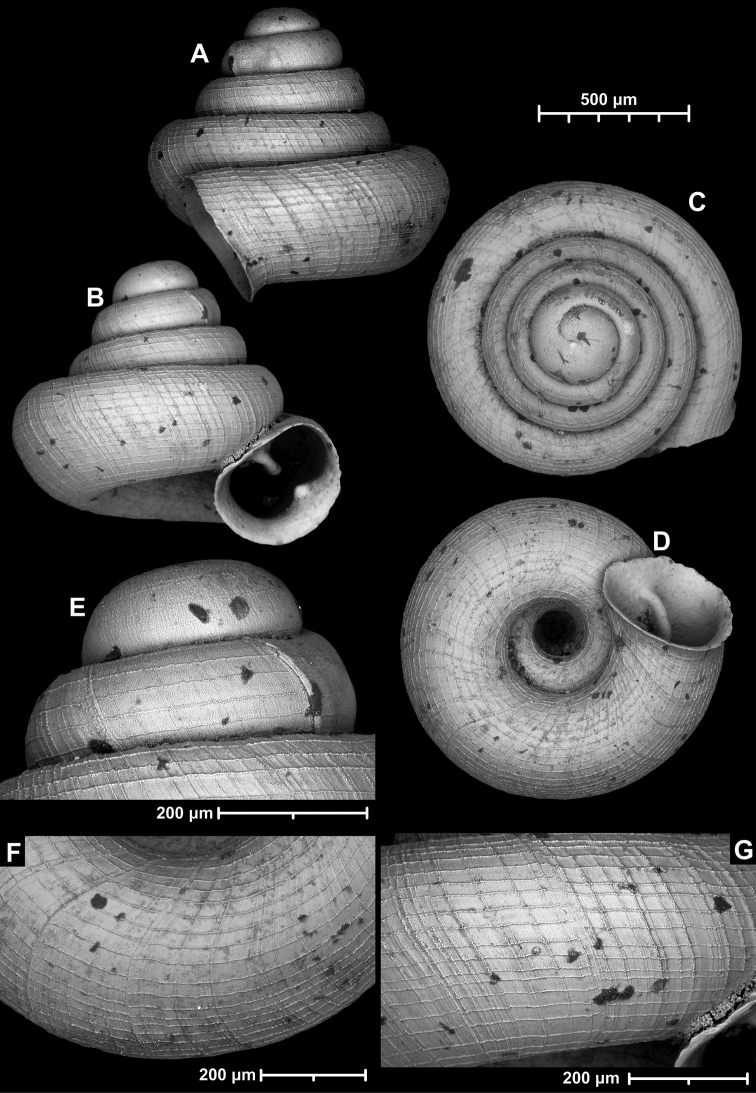
*Angustopilaparallela* Páll-Gergely & Hunyadi, sp. nov. **A, C–H** holotype (HNHM 105298) **B** paratype. (2020/57). Apertural (**A, B**), lateral (**C**), apical (**D**) and ventral (**E**) sides of the shell; sculpture on the protoconch (**F**), ventral (**G**) and frontal (**H**) surface of the body whorl.

**Figure 75.**
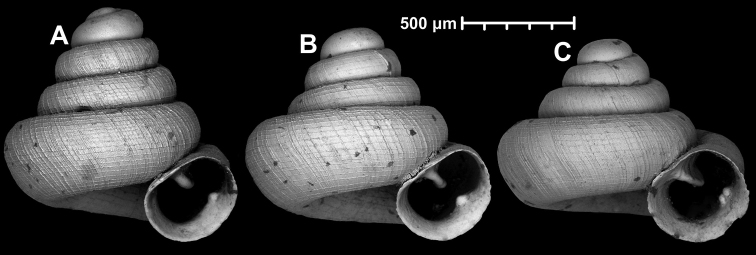
Variability of *Angustopilaparallela* Páll-Gergely & Hunyadi, sp. nov. from the type locality **A, B** holotype **C** paratype.

**Figure 76.**
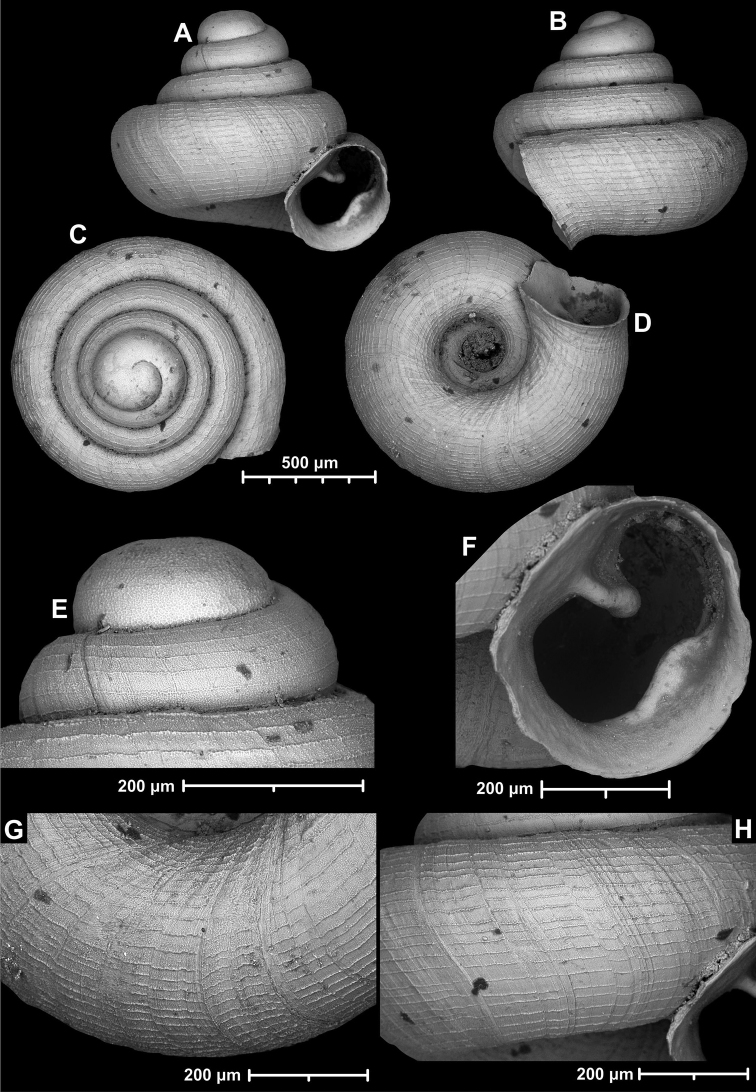
*Angustopilaparallela* Páll-Gergely & Hunyadi, sp. nov. (2020/41). Apertural (**A**), lateral (**B**), apical (**C**) and ventral (**D**) sides of the shell; sculpture on the protoconch with protoconch-teleoconch boundary (**E**); aperture (**F**); ventral (**G**) and frontal (**H**) surface of the body whorl.

##### Diagnosis.

A large, low or high concave-conical *Angustopila* species with a wide umbilicus, dense spiral striation, and two strong apertural barriers (elevated parietal, elongated or pointed lower palatal).

##### Description.

Shell large for the genus, wider than high or rarely slightly higher than wide; off-white, concave-conical, shell height highly variable from strongly depressed to triangular, with regularly growing whorls except for the last one; last whorl widest from apertural view; protoconch consists of 1.25–1.5 whorls, spirally striated with the exception of the first whorl; teleoconch with strong, rather dense, irregular radial ribs and equidistantly-spaced, dense spiral striae (ca. 19–21 on body whorl from apertural view); whorls 4–4.25, rounded with tendency towards shouldered contour (in apertural view, the body whorl is slightly pushed from baso-lateral direction); aperture oblique to shell axis in lateral view; umbilicus wide; aperture suboval; peristome expanded, not reflected; parietal callus not detached from penultimate whorl; parietal tooth elevated, straight, starts in some distance from peristome; lower palatal tooth runs parallel with the peristome on the lower 1/2 of the palatal region, in some specimens pointed, in others ridge-like, situated inside from peristome.

##### Measurements (in mm).

H = 0.85–1.12, D: 1.04–1.14, H/D*100 = 77.3–109.6 (*n* = 18), RUD = 29.0–33.3 (*n* = 5).

##### Differential diagnosis.

*Angustopilamajuscula* sp. nov. is similar to this species by the large shell size, wide umbilicus and the possession of two apertural denticles. However, it has a convex conical shell shape, denser spiral striation and the palatal tooth is situated further up in the aperture. See also under *A.pustulata* sp. nov.

##### Etymology.

Named after the palatal tooth that runs parallel with the peristome.

##### Distribution.

*Angustopilaparallela* sp. nov. is known from two localities in northern Vietnam (Lạng Sơn and Thanh Hóa Provinces) (Fig. 19).

##### Remarks.

Shells from population 2020/57 are generally more depressed than those of 2020/58, although the two groups overlap. The distance between these populations is ca. 300 m (!) in a straight line. Most shells of population 2020/41 possess an elongated palatal tooth, whereas that tooth is pointed in most shells of the other two populations. However, this difference is not exclusive, because there are individuals with a pointed palatal tooth in population 2020/41 and a more elongated tooth in the other two populations. Thus, even if the geographic distance is relatively large overall (ca. 280 km in a straight line), the morphological differences are insufficient to distinguish the population 2020/41 from the other two.

#### ﻿ Angustopila pustulata

Taxon classificationAnimaliaStylommatophoraGastrocoptidae

Páll-Gergely & Hunyadi sp. nov.

F3A99B72-70D2-5576-812D-3DFE2CBA2C74

https://zoobank.org/FDDCB18C-2F2C-43AB-A39D-7B74B2586580

Fig. 77

##### Type material.

***Holotype***: Vietnam • 1 empty shell (H: 0.84 mm, D: 0.93 mm); Hòa Bình Province, Hưng Thi, 37.8 km from Nho Quan towards Hanoi on the Hồ Chí Minh road, left side (locality code: 2020/46); 20°32.74'N, 105°41.89'E; 15 m a.s.l.; 16 Feb. 2020; A. Hunyadi leg.; HNHM 105312.

***Paratypes***: Vietnam • 1 imaged shell + 58 shells; same data as for holotype; coll. HA • 5 shells; same data as for holotype; coll. JJV.

##### Additional material.

Vietnam • 13j/b shells; same data as for holotype; coll. HA.

##### Diagnosis.

A medium-sized, conical or (more often) concave-conical *Angustopila* species with a relatively narrow umbilicus, weak sculpture, comparatively large aperture, a short parietal tooth and a wart-like lower palatal tooth.

##### Description.

Shell of normal size for the genus, slightly higher than wide or slightly wider than high; off-white, conical or more often concave-conical; body whorl widest from apertural view; protoconch consists of 1.25 whorls, with weak spiral striation on entire protoconch; teleoconch with fine, dense, irregularly spaced radial growth lines and somewhat stronger, equidistantly and sparsely-arranged spiral lines (ca. 16–19 on body whorl from standard apertural view); sculpture is weaker than in other *Angustopila* species; whorls 4 or slightly fewer, rounded or slightly shouldered; aperture slightly oblique to shell axis from lateral view; umbilicus relatively narrow; aperture subovoid or reniform with very wide, not distinctly separated sinulus; peristome expanded, not reflected; parietal callus not protruding, not detached from penultimate whorl; parietal tooth straight, strong, relatively low, situated in some distance from peristome edge; lower palatal tooth very small and blunt, blister-like, situated some distance inside the shell from peristome edge.

**Figure 77.**
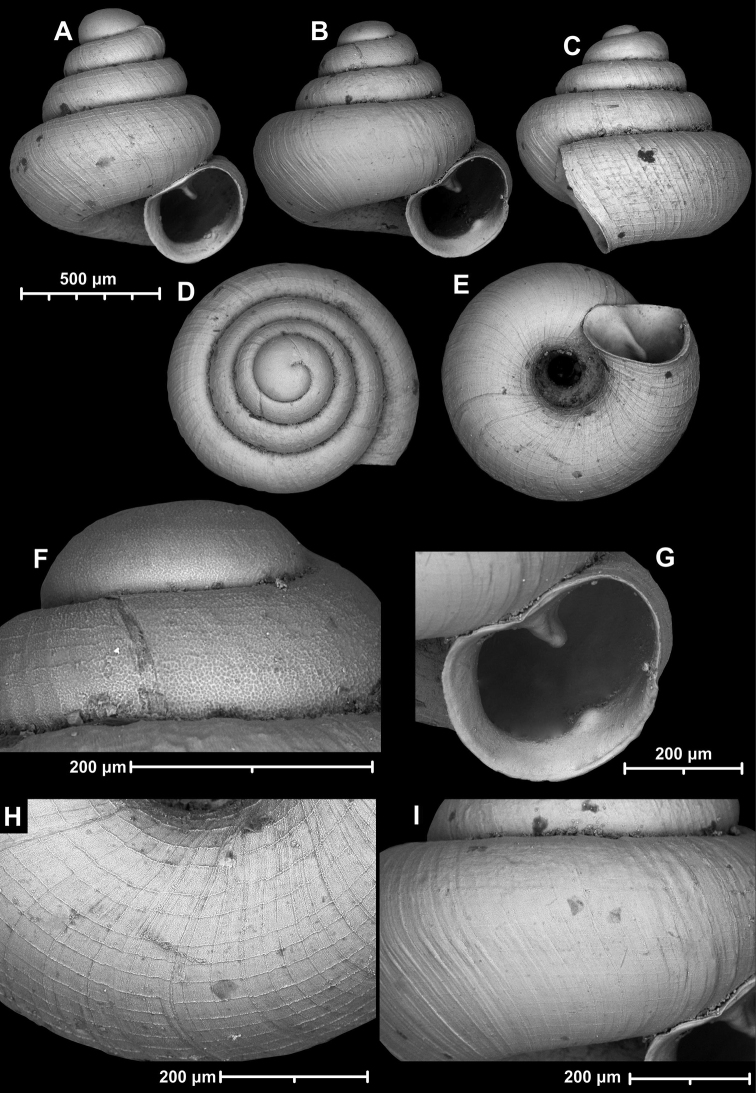
*Angustopilapustulata* Páll-Gergely & Hunyadi, sp. nov. **A** paratype **B–I** holotype (HNHM 105312). Apertural (**A, B**), lateral (**C**), apical (**D**) and ventral (**E**) sides of the shell; sculpture of the protoconch showing protoconch-teleoconch boundary (**F**); aperture (**G**); ventral (**H**) and frontal (**I**) surface of the body whorl.

##### Measurements (in mm).

H = 0.84–1.00, D = 0.87–0.93 H/D*100 = 90.3–108.0 (*n* = 6), RUD = 21.8–26.9 (*n* = 2).

##### Differential diagnosis.

*Angustopilaparallela* sp. nov. is larger, has a stronger palatal tooth, stronger spiral striation, and more depressed, more strongly concave shell shape. See also under *A.antidomedon* sp. nov., *A.bathyodon* sp. nov., *A.gracilis* sp. nov. and *A.bathyodon* sp. nov.

##### Etymology.

The specific epithet *pustulata* (blister-bearing in Latin) refers to the low, blister-like palatal tooth.

##### Distribution.

*Angustopilapustulata* sp. nov. is known from the type locality only in Hòa Bình Province of northern Vietnam (Fig. 27).

#### ﻿ Angustopila reticulata

Taxon classificationAnimaliaStylommatophoraGastrocoptidae

Páll-Gergely & Hunyadi sp. nov.

9F8657CF-60CA-5460-AA6D-4B2EAEFDFB2D

https://zoobank.org/22AA6729-A371-4656-B3F5-3BE9EEE42E28

Fig. 78

##### Type material.

***Holotype***: Vietnam • 1 empty shell (H: 0.94 mm, D: 0.86 mm); Thanh Hóa Province, Quan Hóa District, Khu di tích Hang Ma, gorge of Sȏng Luồng (locality code: 2020/30); 20°23.89'N, 105°04.02'E; 100 m a.s.l.; 12 Feb. 2020; A. Hunyadi leg.; HNHM 105313.

***Paratypes***: Vietnam • 1 figured shell + 15 shells; same data as for holotype; coll. HA.

##### Diagnosis.

A medium-sized *Angustopila* species with a conical shell, strong radial sculpture, a robust strong parietal tooth reaching the peristome edge, and an upper palatal tooth of comparable height, situated in the middle of the palatal region.

##### Description.

Shell of normal size for the genus, slightly higher than wide or rarely slightly wider than high; off-white, translucent when fresh, conical; body whorl widest in standard apertural view; protoconch consists of 1.25–1.5 whorls with spiral striation on the entire protoconch; teleoconch with strong, dense, irregularly spaced, radial ribs crossed by similarly strong spiral striae (ca. 15–16 on body whorl from standard apertural view); on both ventral and dorsal surfaces of body whorl spiral lines dominant or spiral and radial lines are of comparable strength; whorls 4, rounded or very slightly shouldered; aperture very slightly oblique to shell axis from lateral view; umbilicus wide; aperture pear-shaped, sinulus very narrow and strongly isolated due to the near joining of the strong parietal and palatal teeth; peristome expanded, not reflected; parietal callus protruding, detached from penultimate whorl (aperture not adnate); parietal tooth elevated but short, reaches parietal callus, perpendicular to parietal wall; upper palatal tooth almost as high as parietal one, situated near peristome edge just opposite of parietal tooth; a very tiny columellar tooth is indicated in the minority of examined shells; peristome protruding anterior to the palatal tooth.

**Figure 78.**
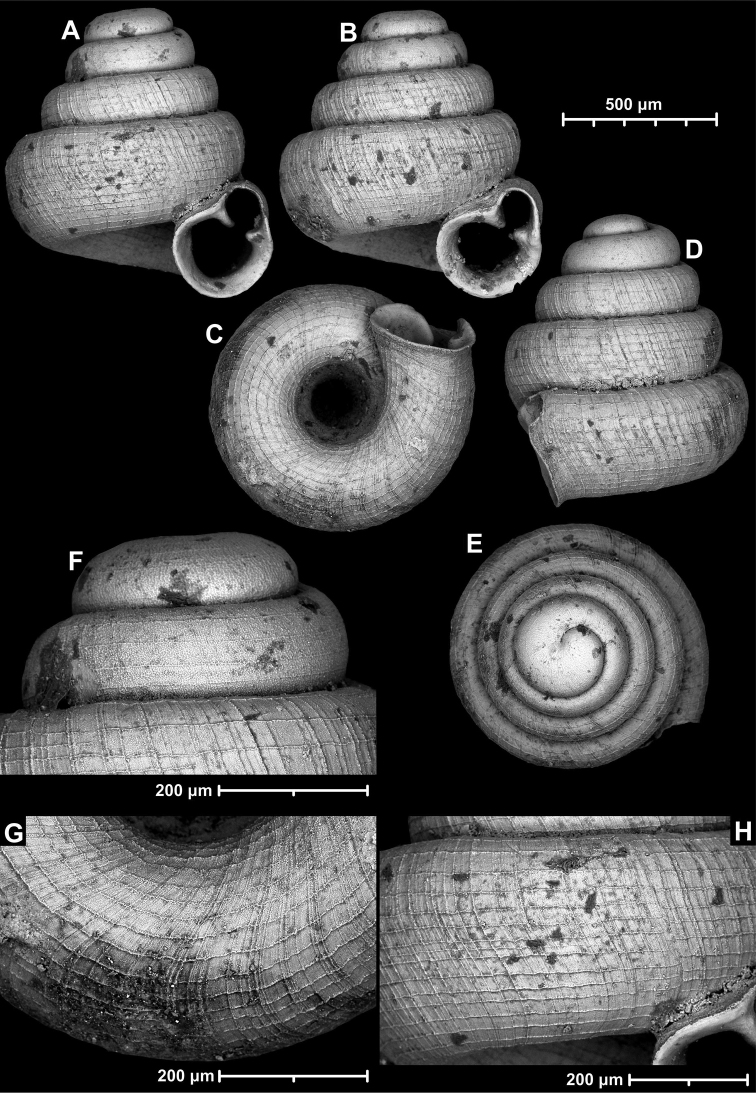
*Angustopilareticulata* Páll-Gergely & Hunyadi, sp. nov. **A, C–H** holotype (HNHM 105313) **B** paratype. Apertural (**A, B**), ventral (**C**), lateral (**D**), and apical (**E**) sides of the shell; sculpture on the protoconch (**F**), ventral (**G**) and frontal (**H**) surface of the body whorl.

##### Measurements (in mm).

H = 0.9–0.95, D = 0.86–0.93, H/D*100 = 86.9–109.3 (*n* = 5), RUD = 32.2 (*n* = 2).

##### Differential diagnosis.

See under *A.tweediei* sp. nov.

##### Etymology.

This new species is named after its pronounced reticulated sculpture.

##### Distribution.

*Angustopilareticulata* sp. nov. is known from the type locality only in Hòa Bình Province of northern Vietnam (Fig. 27).

#### ﻿ Angustopila somsaki

Taxon classificationAnimaliaStylommatophoraGastrocoptidae

Páll-Gergely & Hunyadi sp. nov.

C7795E87-8450-5338-B4D1-2CED0202F308

https://zoobank.org/0AC429B8-70E3-47E4-8C29-889B5A07C5AB

Figs 79 , 80

##### Type material.

***Holotype***: Thailand • 1 empty shell (H: 0.66 mm, D: 0.78 mm); Chiang Mai Province, northeastern part of Doi Chiang Dao, Wat Tham Chiang Dao northwest 2 km (locality code: 2015/6); 19°24.02'N, 98°54.68'E; 835 m a.s.l.; 7 Feb. 2015; A. Hunyadi leg.; CUMZ 7440.

***Paratypes***: Thailand • 1 shell; same data as for holotype; NMBE 550642 • 9 shells; same data as for holotype; coll. HA • 3 shells; Chiang Rai Province, 7 to 9 km SSW of Mae Sai, around Wat Tham Pla (locality code: 2015/18); 20°19.72'N, 99°51.82'E; 400 m a.s.l.; 12 Feb. 2015; A. Hunyadi leg.; coll. HA • 7 shells; Chiang Rai Province, 6 km S of Chiang Khian, toward Pa Ngae, Wat Phra That Charui (locality code: 2015/20); 19°34.41'N, 99°59.19'E; 420 m a.s.l.; 13 Feb. 2015; A. Hunyadi leg.; coll. HA • 1 imaged shell; same data as for preceding; HNHM 103480.

##### Additional material.

Thailand • 2 juvenile shells; same data as for holotype; coll. HA.

##### Diagnosis.

A small, depressed-globular *Angustopila* species with domed dorsal side, a pear-shaped aperture having an apertural axis nearly parallel to shell axis, a strong parietal tooth, and a very weak upper palatal tooth.

##### Description.

Shell small for the genus, wider than high; off-white, depressed globular with domed dorsal side; body whorl widest from standard apertural view; protoconch consists of 1.25 whorls with four spiral striae; teleoconch ornamented by fine radial growth lines and stronger, equidistantly- arranged spiral striae (ca. 12–20 on body whorl from standard apertural view); whorls 3.5, slightly shouldered; aperture slightly oblique to shell axis from lateral view; umbilicus very wide and perspectival; aperture pear-shaped with slight indentation at the parietal side; apertural axis and shell axis join under a small angle (nearly parallel with each other); sinulus relatively wide; peristome expanded, not reflected; parietal callus strongly protruding slightly beyond width of penultimate whorl, visible even from lateral view behind palatal peristome, detached from penultimate whorl; irregular parietal tooth relatively weak, low, but quite long, nearly reaching peristome, oblique to parietal side (joins at an angle less than 90 degrees); upper palatal tooth very weak and low, situated on opposite side to parietal tooth, and set in some distance from peristome.

**Figure 79.**
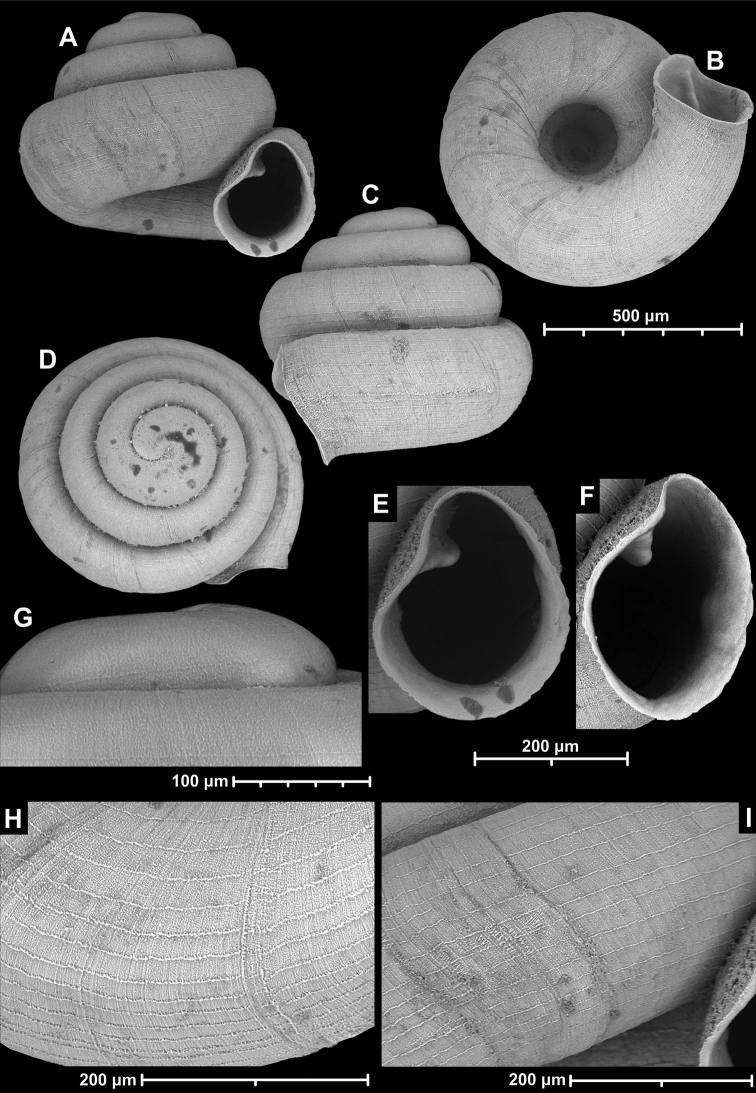
*Angustopilasomsaki* Páll-Gergely & Hunyadi, sp. nov. (holotype, CUMZ 7440). Apertural (**A**), ventral (**B**), lateral (**C**) and apical (**D**) sides of the shell; aperture (**E, F**); sculpture of the protoconch (**G**), ventral (**H**) and frontal (**I**) surface of the body whorl.

**Figure 80.**
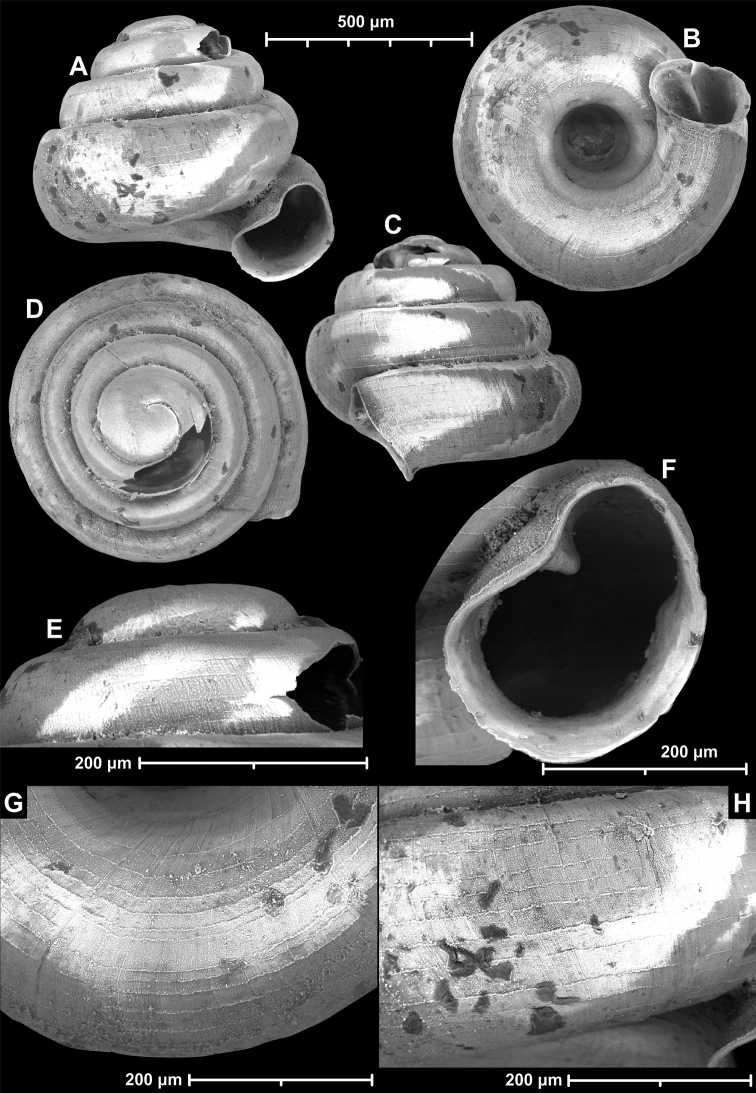
*Angustopilasomsaki* Páll-Gergely & Hunyadi, sp. nov., sample 2015/20, HNHM 103480. Apertural (**A**), ventral (**B**), lateral (**C**), and apical (**D**) sides of the shell; sculpture of the protoconch (**E**); aperture (**F**); ventral (**G**) and frontal (**H**) surface of the body whorl.

##### Measurements (in mm).

H = 0.62–0.67, D = 0.75–0.8, H/D*100 = 80–89.5 (*n* = 9), RUD = 32.9–37.8 (*n* = 4).

##### Differential diagnosis.

*Angustopilapallgergelyi* has a deeper parietal incision and a stronger (more elevated and pointed) upper palatal tooth. See under *A.maasseni* sp. nov.

##### Etymology.

The species is dedicated to Prof. Somsak Panha (Chulalongkorn University, Thailand) to acknowledge his work, which resulted in the discovery of numerous new invertebrate species in Southeast Asia and initiated the careers of many young taxonomists.

##### Distribution.

This species is known from three localities in Chiang Mai and Chiang Rai Provinces in northern Thailand. The largest distance between the localities is ca. 145 km (Fig. 55).

##### Remarks.

Although some small differences could be determined between the two figured shells deriving from two different localities, we found no notable conchological differences between the three populations.

#### ﻿ Angustopila steffeki

Taxon classificationAnimaliaStylommatophoraGastrocoptidae

Páll-Gergely & Grego sp. nov.

DA3F784A-5E2C-55B2-ACA4-61EC4E615FCB

https://zoobank.org/76FA631F-18B7-4743-993F-B6D626BB982D

Fig. 81

##### Type material.

***Holotype***: Laos • 1 empty shell (H: 0.64 mm, D: 0.74 mm); Bolikhamsay Province, large spring lake at S foot of limestone massif 2 km NNE of Na Pavan village, ca. 5 km from Phontan crossing at road 8 (locality code: JG16); 18°13.30'N, 104°44.81'E; 18 Feb. 2017; J. Grego leg.; NHMUK 20170296.

##### Diagnosis.

A small, depressed-globular *Angustopila* species with the aperture oblique to shell axis, a strongly protruding aperture, and relatively weak parietal and rather strong upper palatal tooth.

##### Description.

Shell small, wider than high; off-white, depressed-globular with domed spire; body whorl widest in standard apertural view; protoconch consists of 1.25 whorls, microstructure finely pitted and granular with a powdery superficial texture and with weak spiral striae (although holotype corroded; ca. 19 on body whorl from standard apertural view); teleoconch ornamented by some fine irregular, radial growth lines and stronger, equidistantly-arranged spiral striae; whorls 4, shouldered; aperture not oblique to shell axis from lateral view (although the peristome might be broken); umbilicus very wide; aperture pear-shaped with strongly distinct, relatively narrow sinulus and wider main section; peristome slightly expanded, not reflected; parietal callus strongly protruding, detached from penultimate whorl; upper parietal tooth relatively weak, low, but quite long, reaching peristome, perpendicular to parietal side; palatal tooth relatively strong, situated directly opposite of parietal tooth just at peristome edge.

**Figure 81.**
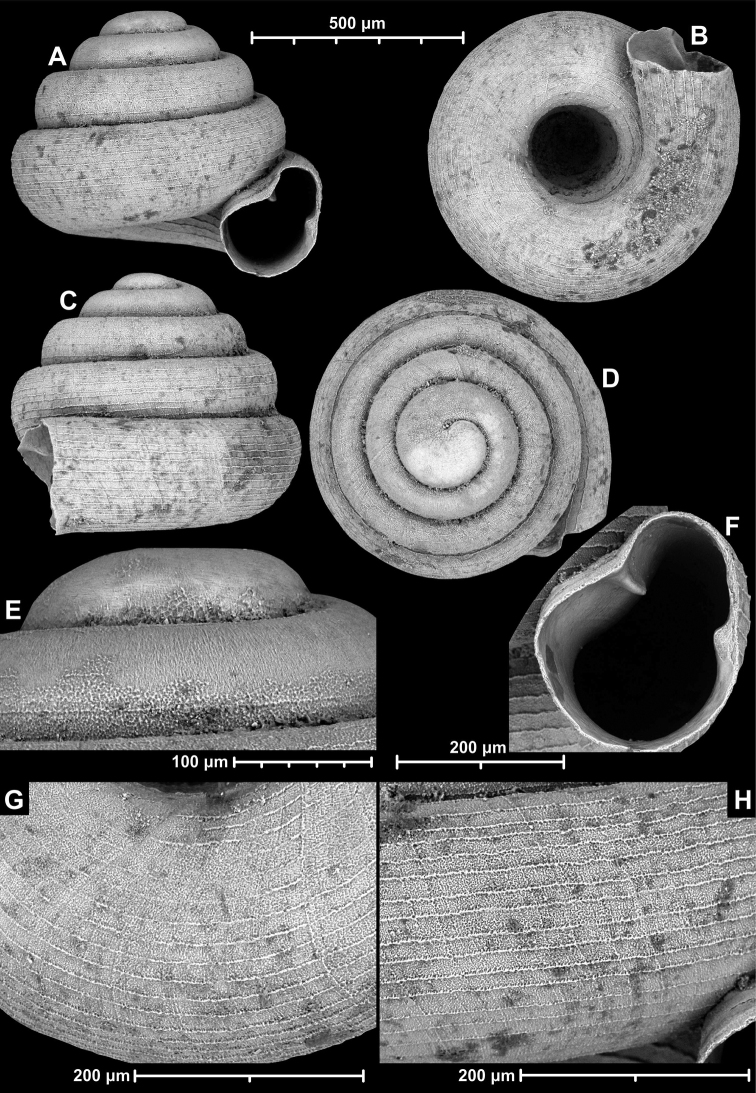
*Angustopilasteffeki* Páll-Gergely & Grego, sp. nov. (holotype, NHMUK 20170296). Apertural (**A**), ventral (**B**), lateral (**C**) and apical (**D**) sides of the shell; sculpture of the protoconch (**E**); aperture (**F**); ventral (**G**) and frontal (**H**) surface of the body whorl.

##### Measurements (in mm).

H = 0.64, D = 0.74, H/D*100 = 86.5, RUD = 36.3 (holotype).

##### Differential diagnosis.

In comparison to other *Angustopila* species possessing two apertural denticles, *Angustopilasteffeki* sp. nov. shows a more depressed shell with a dome-like appearance. *Angustopilapallgergelyi* has a more conical shell, a lower spire, and denser spiral striation. *Angustopilasomsaki* sp. nov. has a less globular shell, less cylindrical (more funnel-shaped) umbilicus, and a less pointed palatal tooth.

##### Etymology.

This species is dedicated to and named after Jozef Šteffek (1952–2013), Slovak malacologist.

##### Distribution.

This species is known from the type locality only in Bolikhamsay Province of Laos (Fig. 48).

#### ﻿ Angustopila tamlod

Taxon classificationAnimaliaStylommatophoraGastrocoptidae

(Panha & Burch, 2002)

2738CC6C-B316-53C7-9D6D-43794A0A5686

Fig. 82

Systenostoma tamlod Panha & Burch, 2002: 118–121, fig. 3.
Angustopila tamlod — Jochum et al. 2014: 30.

##### Type locality.

“Lod Cave, Pang Ma Pa District, Mae Hong Son Province, 19°29'36"N, 98°17'18"E and 10°34'03"N, 98°16'41"E, 800 meters elevation (CUIZM, Ver 025), Thailand. All specimens were collected inside the cave, almost two kilometres from the entrance.”

##### Material examined.

Holotype CUIZM 2839 (Ver 025) (reimaged for this study); Thailand (Mae Hong Son): Pangmapa District: Lod Cave, ex S. Panha 2008 (CUMZ 2831), SMF 331472/1 paratype.

##### Diagnosis.

A medium-sized to large, conical *Angustopila* species with relatively dense spiral striation, a strong parietal tooth, and a weaker upper palatal tooth.

##### Differential diagnosis.

*Angustopilabidentata* sp. nov. has a strongly oblique aperture, more rounded whorls, and its parietal callus is detached from the penultimate whorl. *Angustopiladominikae* is smaller, has a globular shell with a narrower, more horizontally oriented aperture. *Angustopilamajuscula* sp. nov. is larger, has a half whorl more and a wider umbilicus. See also under *A.huoyani*.

**Figure 82.**
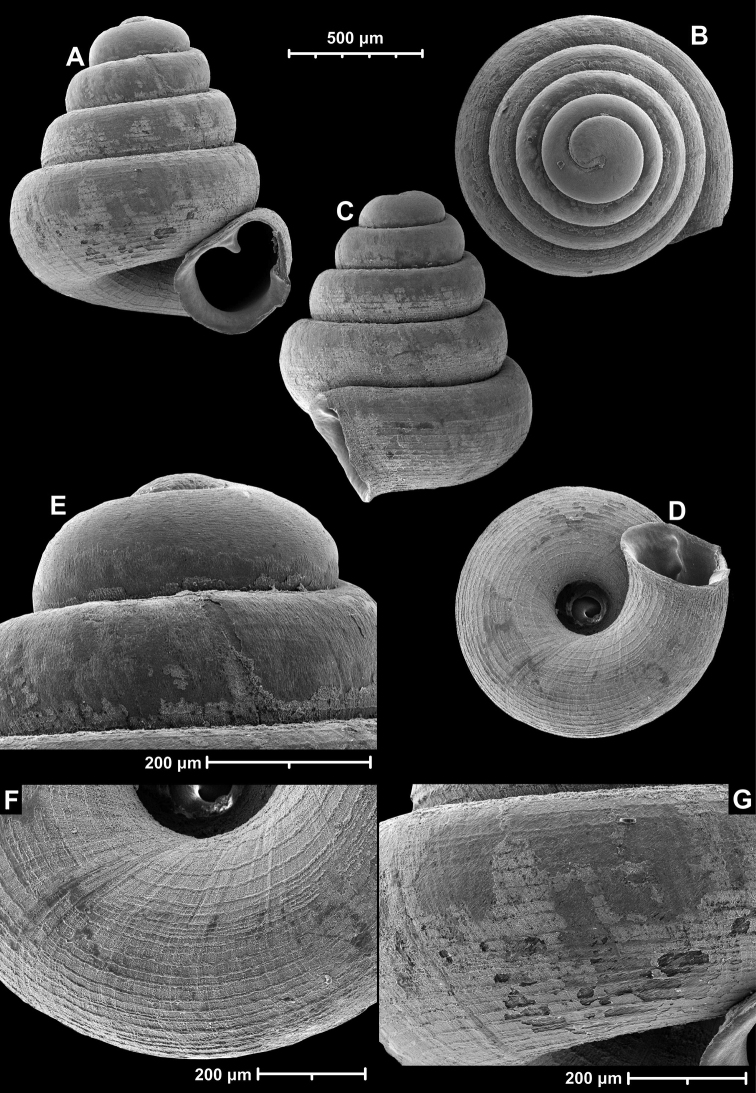
Holotype of *Angustopilatamlod* (Panha & Burch, 1999). Apertural (**A**), apical (**B**), lateral (**C**) and ventral (**D**) sides of the shell; eroded sculpture on the protoconch (**E**), ventral (**F**) and frontal (**G**) surface of the body whorl. SEM Images: Chirasak Sutcharit.

##### Distribution.

This species is known only from the type locality (Fig. 31).

#### ﻿ Angustopila tweediei

Taxon classificationAnimaliaStylommatophoraGastrocoptidae

Páll-Gergely & Hunyadi sp. nov.

5E469A23-F5D9-578A-A3BB-EF4F046E82DF

https://zoobank.org/9D51E177-4754-417E-8F0E-5A139A01C84B

Fig. 83

##### Type material.

***Holotype***: Vietnam • 1 empty shell (H: 0.9 mm, D: 0.81 mm); Sơn La Province, 10 km from centre of Mộc Châu towards Sơn La, right side of road no. 6, south of Tất Ngoằng (locality code: 2020/5); 20°52.58'N, 104°35.34'E; 715 m a.s.l.; 5 Feb. 2020; A. Hunyadi leg.; HNHM 105314.

***Paratypes***: Vietnam • 13 adult shells; same data as for holotype; coll. HA.

##### Additional material.

Vietnam • 1 j/b shell; same data as for holotype; coll. HA.

##### Diagnosis.

A medium-sized *Angustopila* species with a conical (very slightly conical-globular) shell, a strong parietal tooth reaching the peristome edge, and an upper palatal tooth of comparable height.

##### Description.

Shell of normal size for the genus, higher than wide; off-white, translucent, conical to very slightly conical-globular; body whorl widest in standard apertural view; protoconch consists of 1.5 whorls with rather weak spiral striation preceding the first teleoconch whorl; teleoconch with very fine irregularly spaced, weak radial growth lines crossed by much stronger irregularly and sparsely-spaced spiral striae (ca. 11–14 on body whorl from apertural view); on both ventral and dorsal surfaces of body whorl spiral lines dominant or spiral and radial lines are of comparable strength; whorls 4–4.25, rounded or very slightly shouldered; aperture slightly oblique to shell axis from lateral view; umbilicus moderately wide; aperture reniform, sinulus relatively narrow and isolated due to the pronounced parietal and palatal teeth; peristome expanded, not reflected; parietal callus protruding, detached from penultimate whorl (aperture not adnate); parietal tooth elevated, strong, but short, reaches parietal callus, perpendicular to parietal wall; palatal tooth almost as high as parietal one, situated near peristome edge, at middle of palatal region, just opposite of parietal tooth.

**Figure 83.**
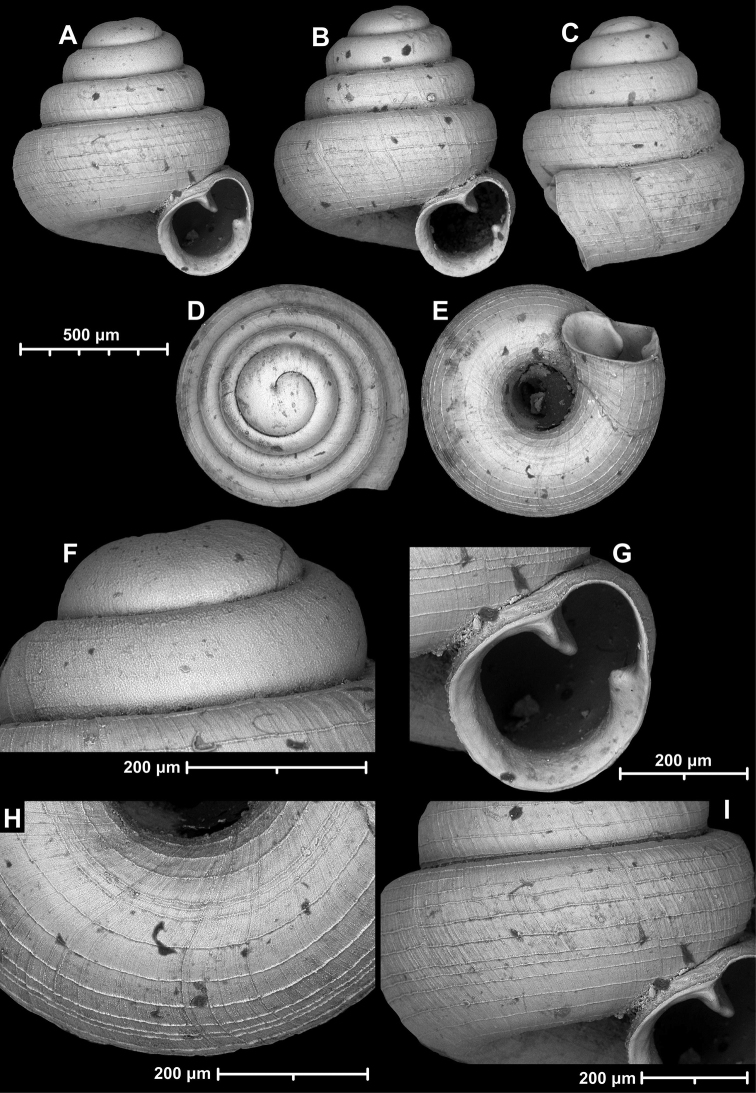
*Angustopilatweediei* Páll-Gergely & Hunyadi, sp. nov. **A** paratype **B–I** holotype (HNHM 105314). Apertural (**A, B**), lateral (**C**), apical (**D**) and ventral (**E**) sides of the shell; sculpture on the protoconch (**F**); aperture (**G**); ventral (**H**) and frontal (**I**) surface of the body whorl.

##### Measurements (in mm).

H = 0.86–0.9; D = 0.81–0.85, H/D*100 = 102.4–111.1 (*n* = 4), RUD = 26.5–28.4 (*n* = 2).

##### Differential diagnosis.

*Angustopilatweediei* sp. nov. is most similar to *A.bathyodon* sp. nov. in terms of shell size and shape, and the position of apertural barriers. However, the spire of the latter species is lower, and both parietal and palatal teeth are situated much deeper inside the aperture. *Angustopilareticulata* sp. nov. is similar in general shell shape and size and the formation of the apertural barriers. However, it has a thicker peristome, a protruding palatal lip anterior to the palatal tooth, a rather reticulated superficial sculpture (i.e., the radial lines are strong), and slightly wider umbilicus. The apertural denticles of *A.huoyani* are similar, but that species is much larger, has more whorls, and a more horizontally oriented aperture than this new species.

##### Etymology.

This new species is dedicated to and named after Michael W.F. Tweedie (1907–1993), English naturalist and malacologist, who pioneered collecting Southeast Asian land snails via sediment sampling methods (Tweedie 1961).

##### Distribution.

This species is known from the type locality only (Fig. 27).

#### ﻿ Angustopila uvula

Taxon classificationAnimaliaStylommatophoraGastrocoptidae

Páll-Gergely & Hunyadi sp. nov.

8821DBD2-C757-5350-9B4E-4BE8A56A9503

https://zoobank.org/08C2B4F0-F809-4656-B593-088EAC4F6FC6

Fig. 84

##### Type material.

***Holotype***: Thailand • 1 empty shell (H: 1.0 mm, D: 0.95 mm); Chumphon Province, Pathio NE 2.5 km, Tham Khao Phlu (locality code: 2015/37); 10°43.85'N, 99°19.24'E; 30 m a.s.l.; 23 Feb. 2015; A. Hunyadi leg.; CUMZ 7439.

***Paratypes***: Thailand • 2 shells; same data as for holotype; NMBE 550647 • 2 shells; same data as for holotype; coll. PGB • 62 shells; same data as for holotype; coll. HA • 3 shells; same data as for holotype; coll. JJV.

##### Additional material.

Thailand • 16 j/b shells; same data as for holotype; coll. HA.

##### Diagnosis.

A medium-sized *Angustopila* species with concave-conical shell, widened body whorl, wide umbilicus, and strong parietal and lower palatal teeth.

##### Description.

Shell of normal size for the genus, slightly higher than wide or slightly wider than high; pale grey, concave-conical with broadly widened body whorl; protoconch consists of 1.25 whorls, with very weak signs of spiral striation on its last whorl; teleoconch finely ornamented with irregularly spaced, strong radial growth lines crossed by fine rows of regularly spaced spiral striae (ca. 16–18 on body whorl from apertural view); on ventral surface of body whorl radial lines dominant (in ca. half of specimens radial lines not conspicuous); frontal surface of body whorl with equally strong radial and spiral lines, or spiral ones stronger; whorls 4.5, rounded; aperture oblique to shell axis from lateral view; umbilicus wide; aperture ovoid, divided into two sections by long parietal tooth; sinulus wide, even larger in area than other portion of aperture; peristome expanded, not reflected; parietal callus not conspicuous, detached from penultimate whorl; parietal tooth very high along the parietal side, perpendicular to shell wall; palatal tooth minute, blunt, situated opposite and slightly to the left of the parietal tooth, and inside the aperture a small distance from peristome.

**Figure 84.**
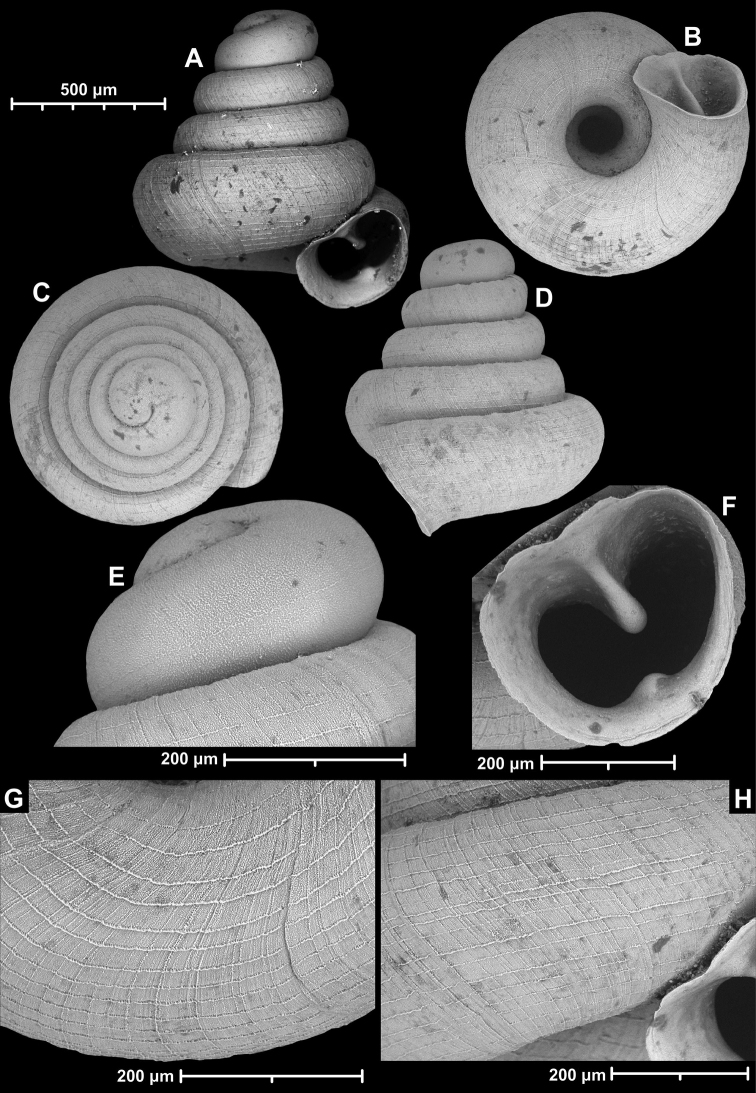
*Angustopilauvula* Páll-Gergely & Hunyadi, sp. nov. (holotype, CUMZ 7439). Apertural (**A**), ventral (**B**), apical (**C**) and lateral (**D**) sides of the shell; sculpture on the protoconch (**E**); aperture (**F**); ventral (**G**) and frontal (**H**) surface of the body whorl.

##### Measurements (in mm).

H = 0.86–0.98, D = 0.93–0.99, H/D*100 = 86.9–106.5 (*n* = 13), RUD = 30.9–32.0 (*n* = 3).

##### Differential diagnosis.

*Angustopilauvula* sp. nov. is most similar to *Angustopilaoccidentalis* sp. nov., but differs from that species in the following characters: shell concave-conical (instead of conical), body whorl wider, radial lines often stronger on the body whorl, parietal tooth higher and ends closer to peristome. *Angustopilaconcava* is larger than *A.uvula* sp. nov., its aperture is more oblique, lacks a palatal tooth, and has a weaker parietal tooth. The shell shape of *A.uvula* sp. nov. is also similar to that of other *Angustopila* species possessing a single apertural tooth (*A.fabella*, *A.monodon* sp. nov.). See also under *A.antidomedon* sp. nov.

##### Etymology.

The parietal tooth of this new species is reminiscent of the fleshy uvula structure at the back of the human throat. The specific epithet *uvula* is to be used as a noun in apposition.

##### Distribution.

This species is known only from the type locality in Thailand’s Chumphon Province (Fig. 19).

#### ﻿ Angustopila vitrina

Taxon classificationAnimaliaStylommatophoraGastrocoptidae

Páll-Gergely & Hunyadi sp. nov.

26D79591-94A3-5F6E-86AE-2E508658D13E

https://zoobank.org/0E157582-B54B-4DAB-81ED-7523D56E5A83

Fig. 85

##### Type material.

***Holotype***: Vietnam • 1 empty shell (H: 0.83 mm, D: 0.78 mm); Thanh Hóa Province, Như Thanh District, 600 m south from Xuân Khang along road no. 45, around the cave temple (locality code: 2020/40); 19°40.35'N, 105°31.07'E; 80 m a.s.l.; 14 Feb. 2020; A. Hunyadi leg.; HNHM 105315.

***Paratypes***: Vietnam • 7 shells; same data as for holotype; coll. HA.

##### Diagnosis.

A small to medium-sized, conical *Angustopila* species with a strong, elevated, clavate parietal tooth, and a small, blunt, blister-like subcolumellar tooth.

##### Description.

Shell small to medium-sized for the genus, slightly higher than wide; pale grey, translucent, conical or slightly concave-conical with an elevated spire; body whorl widest from standard apertural view; protoconch consists of ca. 1.5 whorls, with weak signs of spiral structure preceding the first teleoconch whorl; teleoconch with some fine, irregular radial growth lines and similarly fine, irregularly-arranged spiral striae (ca. 14 or 15 on body whorl from standard apertural view); whorls 4.5–4.75, slightly shouldered; aperture only very slightly oblique to shell axis from lateral view; umbilicus moderately wide; aperture ovate, sinulus broad; peristome expanded, not reflected; parietal callus not protruding, slightly detached from penultimate whorl; parietal tooth prominent, high, extends beyond half of aperture width, with club-like cross section, perpendicular to parietal side, almost reaching edge of peristome; subcolumellar tooth blister-like (see also remarks), rarely absent, situated in some distance from peristome.

**Figure 85.**
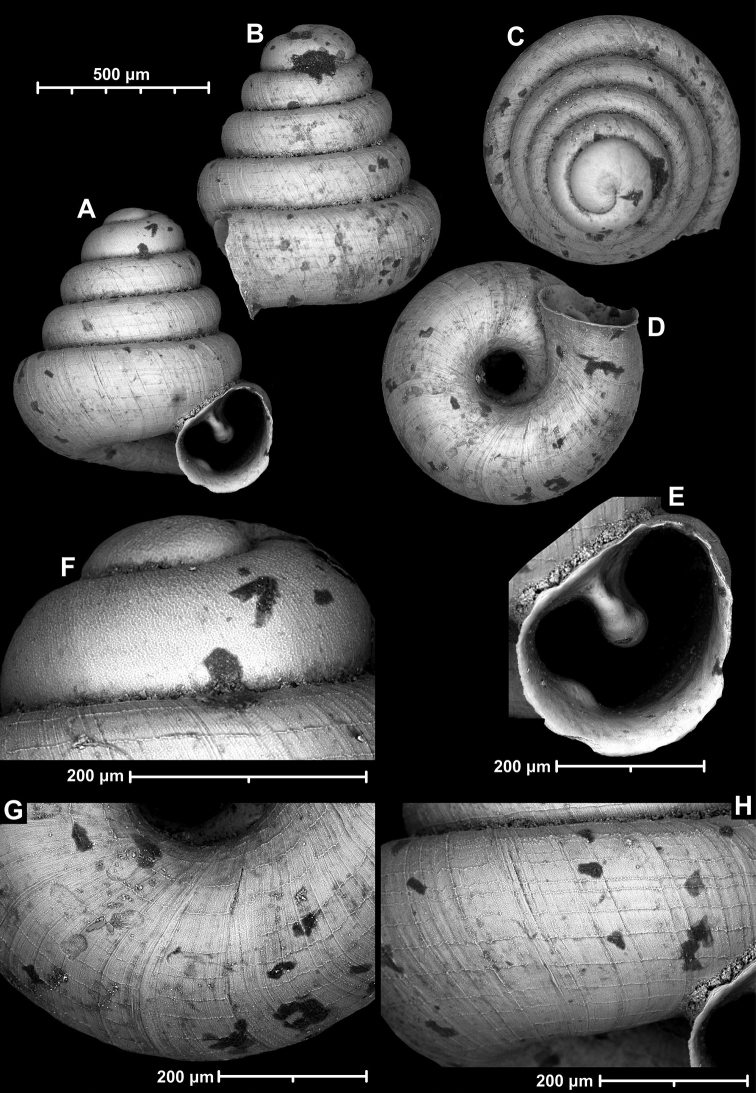
*Angustopilavitrina* Páll-Gergely & Hunyadi, sp. nov. (holotype, HNHM 105315). Apertural (**A**), lateral (**B**), apical (**C**), ventral (**D**) sides of the shell; aperture (**E**); sculpture of the protoconch (**F**), ventral (**G**) and frontal (**H**) surface of the body whorl.

##### Measurements (in mm).

H = 0.82–0.87, D = 0.74–0.85, H/D*100 = 100–112.2 (*n* = 5), RUD = 26.9–28.6 (*n* = 2).

##### Differential diagnosis.

*Angustopilamargaritarion* sp. nov. is the most similar species due to the strong, club-shaped parietal tooth. It has 1 less whorl and a conical-ovoid shape and lacks the subcolumellar tooth.

##### Etymology.

The specific epithet refers to the glass-like transparent shell of this new species.

##### Distribution.

*Angustopilavitrina* sp. nov. is known from the type locality only in Thanh Hóa Province, Vietnam (Fig. 27).

##### Remarks.

One out of the seven paratypes also lacks the subcolumellar tooth. The species is still recognisable and can be distinguished from other *Angustopila* by possessing a single parietal tooth as well by its distinct morphology (having a club-like cross section).

#### ﻿ Angustopila

Taxon classificationAnimaliaStylommatophoraGastrocoptidae

sp. 1

E6AA0CA4-8212-552A-B7A3-810A6FA8C2C1

Suppl. material 3: fig. S42

##### Material examined.

Laos • 1 shell; Luang Prabang Province, 3.1 km from centre of Nong Khiaw towards Pak Xeng, Tham Pha Toke, around the cave (locality code: 2019/114); 20°33.22'N, 102°37.72'E; 345 m a.s.l.; 5 Oct. 2019; A. Hunyadi leg.; coll. HA.

##### Measurements (in mm).

H = 0.91, D = 0.87, H/D*100 = 104.6, RUD = 29.5.

##### Differential diagnosis.

The single shell is similar in size, shape, and arrangement of the teeth to *A.tweediei* sp. nov., but differs in the ovate aperture, the weaker teeth and thinner peristome. More material is needed to examine whether these differences are constant and sufficient for species-level distinction.

##### Distribution.

Known from a single site only (Fig. 55).

### ﻿Species with three or more apertural denticles

#### ﻿ Angustopila apiaria

Taxon classificationAnimaliaStylommatophoraGastrocoptidae

Páll-Gergely & Hunyadi sp. nov.

A9433A21-3AA8-5238-83A6-185B6337A7D5

https://zoobank.org/519DFD96-14C6-423E-9D5C-8FD6A6D4AE2F

Fig. 86

##### Type material.

***Holotype***: Vietnam • 1 empty shell (H: 1.04 mm, D: 0.85 mm); Ðà Nẵng, Ngũ Hành Sơn, Thái Sơn, vicinity of Chùa Quán Thế Äm (locality code: 2019/25a); 15°59.94'N, 108°15.33'E; 10 m a.s.l.; 11 Feb. 2019; A. Hunyadi leg.; HNHM 105316.

***Paratypes***: Vietnam • 37 shells; same data as for holotype; coll. HA • 3 shells; same data as for holotype; coll. JJV.

##### Additional material.

Vietnam • 4 j/b shells; same data as for holotype; coll. HA.

##### Diagnosis.

A medium-sized, high conical *Angustopila* species with a narrow umbilicus, protruding aperture, widely spaced and strong spiral striation, and three prominent apertural barriers (slightly curved parietal, deeply situated lower palatal, inward running subcolumellar).

##### Description.

Shell of normal size for the genus, higher than wide; off-white, conical with slowly increasing whorls; body whorl widest from standard apertural view; protoconch consists of 1.25 whorls, with very slight indication of spiral striation preceding the first teleoconch whorl; teleoconch ornamented by fine, irregular and weak, radial growth lines and conspicuous, prominently raised and equidistantly-spaced spiral striae (ca. 12 or 13 on body whorl from standard apertural view); whorls 4.75, rounded; aperture only slightly oblique to shell axis from lateral view; umbilicus moderately wide; aperture protruding obliquely (visible from basal view), aperture subquadrate with straight parietal side; peristome expanded, not reflected; parietal callus protruding, detached from penultimate whorl; parietal tooth tongue-like and extended, its base straight, its tip slightly turned up in palatal direction, but bent away from palatal tooth; lower palatal tooth low but prominent, wide, elongated, almost perpendicular to the peristome, its outer end situated close to basal lip; subcolumellar tooth strong, as high as the palatal but less wide, elongated, perpendicular to peristome, its outer end situated on the lower part of columellar side of peristome.

**Figure 86.**
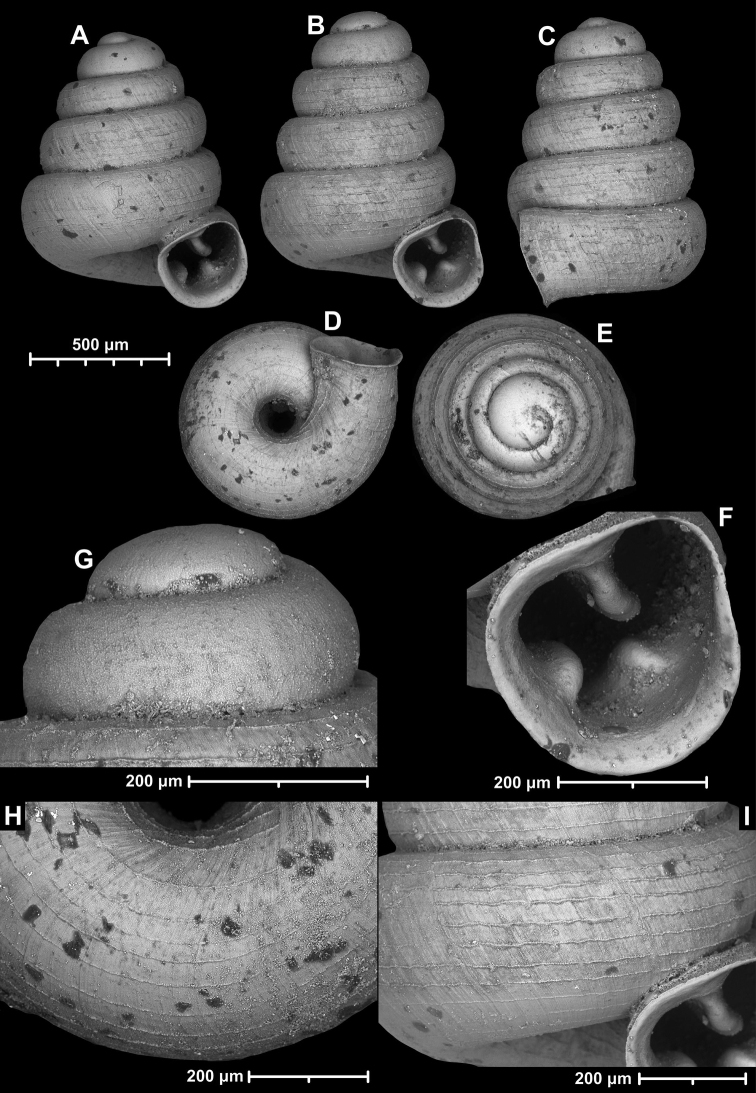
*Angustopilaapiaria* Páll-Gergely & Hunyadi, sp. nov. **A** paratype (specimen 2) **B–I** holotype (HNHM 105316). Apertural (**A, B**), lateral (**C**), ventral (**D**), and apical (**E**) sides of the shell; aperture (**F**); sculpture on the protoconch (**G**), ventral (**H**) and frontal (**I**) surface of the body whorl.

##### Measurements (in mm).

H = 0.93–1.04; D = 0.8–0.86, H/D*100 = 113.4–126.3 (*n* = 6), RUD = 25.9–27.2 (*n* = 2).

##### Differential diagnosis.

The high conical shell shape, narrow umbilicus, protruding aperture, prominent, widely spaced spiral striae, and three strong apertural teeth distinguish this species from all other *Angustopila* species. The most similar species is *Angustopilatridentata* sp. nov., which is concave-conical instead of ovoid or high-conical and bears a wider umbilicus and denser spiral striation. The columellar tooth of *A.apiaria* sp. nov. runs perpendicular to the shell axis (runs inside the aperture) whereas that of *A.tridentata* sp. nov. runs along the peristome. The parietal tooth of *A.apiaria* sp. nov. is located deeper and positioned lower than that of *A.tridentata* sp. nov.

##### Etymology.

Named after its shape reminiscent of a classic apiary, beehive (*apiarium*) in Latin.

##### Distribution.

This species is known only from the type locality region of Chùa Quán Thế Äm, Vietnam (Fig. 19).

##### Remarks.

This is the only *Angustopila* species possessing teeth other than the parietal lamella (lower palatal and subcolumellar teeth) that are perpendicular to the peristome, i.e., run into the aperture. This may mean that *A.apiaria* sp. nov. would deserve a genus of its own. Moreover, this is the most south-eastern occurring *Angustopila* species. Central Vietnam so far seems to have different land snail fauna than northern Vietnam.

#### ﻿ Angustopila apokritodon

Taxon classificationAnimaliaStylommatophoraGastrocoptidae

Páll-Gergely & Hunyadi sp. nov.

518B854C-2FC8-5CBD-A694-CB3BCA1CBA86

https://zoobank.org/A9BB647B-6D1B-4FB1-BD26-0644957B9A7D

Fig. 87

##### Type material.

***Holotype***: Vietnam • 1 empty shell (H: 0.87 mm, D: 0.81 mm); Sơn La Province, Quỳnh Nhai district, 20 km north from crossing point to Thuận Châu, Chiềng Khoang, cave above the village (locality code: 2020/9); 21°33.44'N, 103°40.91'E; 315 m a.s.l.; 7 Feb. 2020; A. Hunyadi leg.; HNHM 105317.

***Paratypes***: Vietnam • 4 shells; same data as for holotype; coll. HA.

##### Diagnosis.

A medium-sized, conical *Angustopila* species with three prominent apertural barriers (long parietal tooth reaching parietal callus, prominent lower palatal tooth, prominent subcolumellar tooth).

##### Description.

Shell of normal size for the genus, slightly higher than wide; off-white, translucent; conical with regularly growing whorls; body whorl widest in standard apertural view; protoconch consists of 1.5 whorls, with slight spiral striation on the last protoconch whorl; teleoconch with some strong to weak radial striae, occasionally grouped in multiple thread-like lines between stronger radial growth lines and spiral striae (ca. 12–14 on body whorl from standard apertural view) of comparable strength, resulting in a reticulated sculpture; whorls 4.25–4.5, rounded; aperture slightly oblique to shell axis in lateral view; umbilicus wide; aperture broad piriform-subquadrate with straight concave parietal part; peristome expanded, not reflected; parietal callus not detached from penultimate whorl; parietal tooth large and straight, directed towards lower palatal tooth; anterior to large parietal tooth (on the parietal callus) the callus is supported with a whitish triangular thickening facing to the front; palatal tooth situated on lower part of palatal wall (probably homologous with the lower palatal tooth), small, denticle-like, sits close to basal lip; subcolumellar tooth situated at junction of columellar and basal positions, similar in size and alignment to the peristome edge and in line with the palatal tooth.

**Figure 87.**
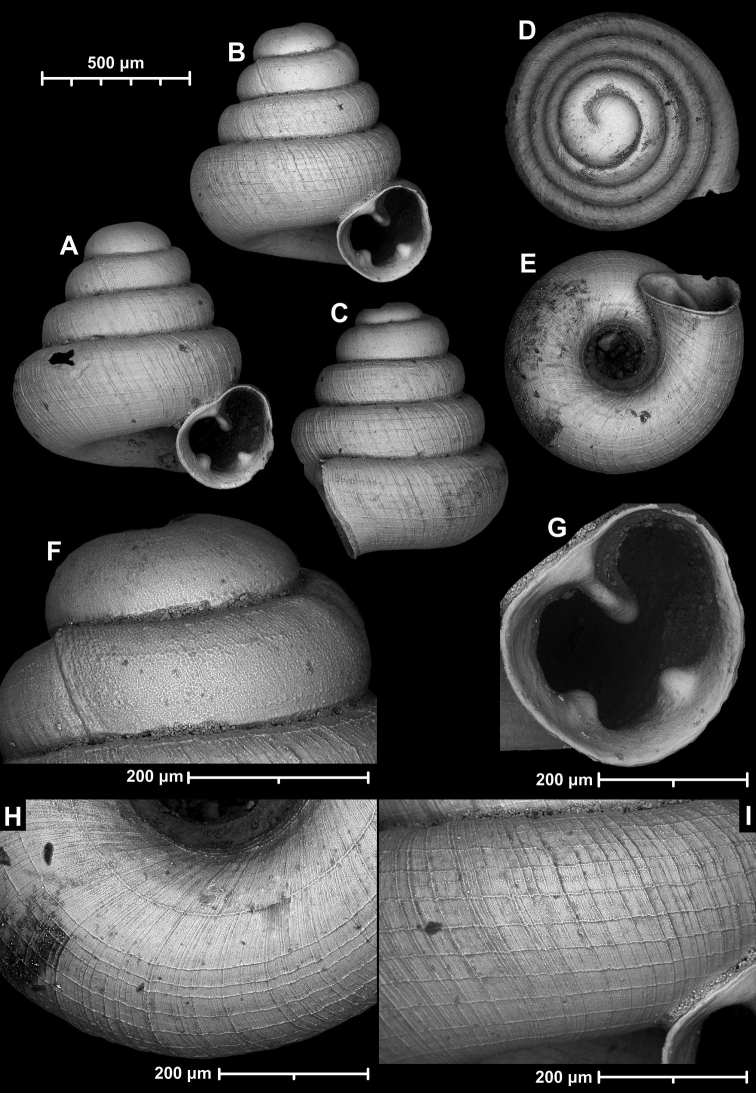
*Angustopilaapokritodon* Páll-Gergely & Hunyadi, sp. nov. **A** paratype **B–I** holotype (HNHM 105317). Apertural (**A, B**), lateral (**C**), apical (**D**) and ventral (**E**) sides of the shell; sculpture of the protoconch with protoconch-teleoconch boundary (**F**); aperture (**G**); ventral (**H**) and frontal (**I**) surface of the body whorl.

##### Measurements (in mm).

H = 0.87–0.92, D = 0.81–0.86, H/D*100 = 101.2–109.6 (*n* = 4), RUD = 30.5–30.7 (*n* = 2).

##### Differential diagnosis.

No other *Angustopila* species shows similar arrangement of apertural barriers (parietal + lower palatal + subcolumellar), and the thick callus anterior to the parietal tooth has not been observed in any other congeners. Maybe *Angustopilatetradon* sp. nov. could be considered as being most similar, but it has an additional upper palatal tooth and a lower conical shell shape. Furthermore, *A.rara* sp. nov. has a narrower umbilicus, a wider subcolumellar tooth, and a more apically situated palatal tooth.

##### Etymology.

The specific epithet *apokritodon* derives from the Greek words *ἀπόκριτος* (= separated) and *ὀδούς* (= tooth) referring to the large distance between the palatal and subcolumellar teeth.

##### Distribution.

This species is only known from the type locality in Sơn La Province in Vietnam (Fig. 27).

#### ﻿ Angustopila coprologos

Taxon classificationAnimaliaStylommatophoraGastrocoptidae

Páll-Gergely, Jochum & Hunyadi, 2022

E3BDB1C2-2683-5C6A-83AB-646838C2AAA0

##### Diagnosis.

This species is characterised by strong spiral sculpture consisting of a series of coarse elevations (flat-topped beads) in a chain-like pattern and three or four well-developed teeth (one parietal, one or two palatal, one subcolumellar).

##### Differential diagnosis.

The unique sculpture together with the shell and aperture shape and the arrangement of apertural barriers distinguishes this species from all congeners.

##### Remarks.

Two subspecies are distinguished based on differences in the apertural barriers.

Living specimens of both subspecies place mud granules (possibly mucus-packaged faeces) on their shells, which is most probably for camouflage, and/or may play a role in biochemical communication and water retention (Páll-Gergely et al. 2022).

#### ﻿ Angustopila coprologos coprologos

Taxon classificationAnimaliaStylommatophoraGastrocoptidae

Páll-Gergely, Jochum & Hunyadi, 2022

43BC8F48-8450-5BD0-B08F-2E48399A130A

Angustopila coprologos Páll-Gergely, Jochum & Hunyadi, in Páll-Gergely et al. 2022: 66, figs 1, 2.

##### Type locality.

“Laos, Bolikhamsai Province, 15 km southeast + 4.5 km northeast (on a side road) towards centre of Lak Sao, Phu Phako, limestone gorge, 510 m a.s.l., 18°06.546'N, 105°03.778'E”.

##### Measurements (in mm).

H = 0.49–0.58, D = 0.66–0.76; H/D*100 = 70.7–80.6 (*n* = 19).

##### Distribution.

This subspecies is known from the type locality only (Fig. 64).

#### ﻿ Angustopila coprologos uninodus

Taxon classificationAnimaliaStylommatophoraGastrocoptidae

Páll-Gergely & Grego ssp. nov.

4D5E7110-9374-5557-83BE-DD1CE0C6D3C9

https://zoobank.org/6D78E5AA-97D0-42D8-B01D-8A111729AE3D

Fig. 88

##### Type material.

***Holotype***: Laos • 1 empty shell (H: 0.54 mm, D: 0.7 mm); Bolikhamsay Province, NE foot of Pha Hông limestone massif 7 km N of Lak Sao (locality code: JG12); 18°13.91'N, 104°56.41'E; 16 Feb. 2017; J. Grego leg.; NHMUK 20170295.

***Paratypes***: Laos • 3 shells; same data as for holotype; coll. JG • 1 shell; same data as for holotype; NMBE 550641 • 152 shells; Bolikhamsai Province, 5.2 km northwest from centre of Lak Sao, 600 m east of quarry, limestone rock (locality code: 2019/111); 18°13.54'N, 104°56.89'E; 515 m a.s.l.; 2 Oct. 2019; A. Hunyadi leg.; coll. HA • 5 shells; same data as for preceding; coll. JJV • 14 shells; Bolikhamsai Province, 5.3 km west-southwest of centre of Lak Sao, rock wall above Wat Pa Pha Nang Rong (locality code: 2019/109); 18°11.30'N, 104°56.45'E; 520 m a.s.l.; 1 Oct. 2019; A. Hunyadi leg.; coll. HA.

##### Differential diagnosis.

This subspecies differs from the nominotypical subspecies in the possession of a single (instead of two) palatal denticle. Judging from the relative position, the single palatal tooth of *Angustopilacoprologosuninodus* ssp. nov. is homologous with the upper palatal tooth of *A.coprologoscoprologos*.

**Figure 88.**
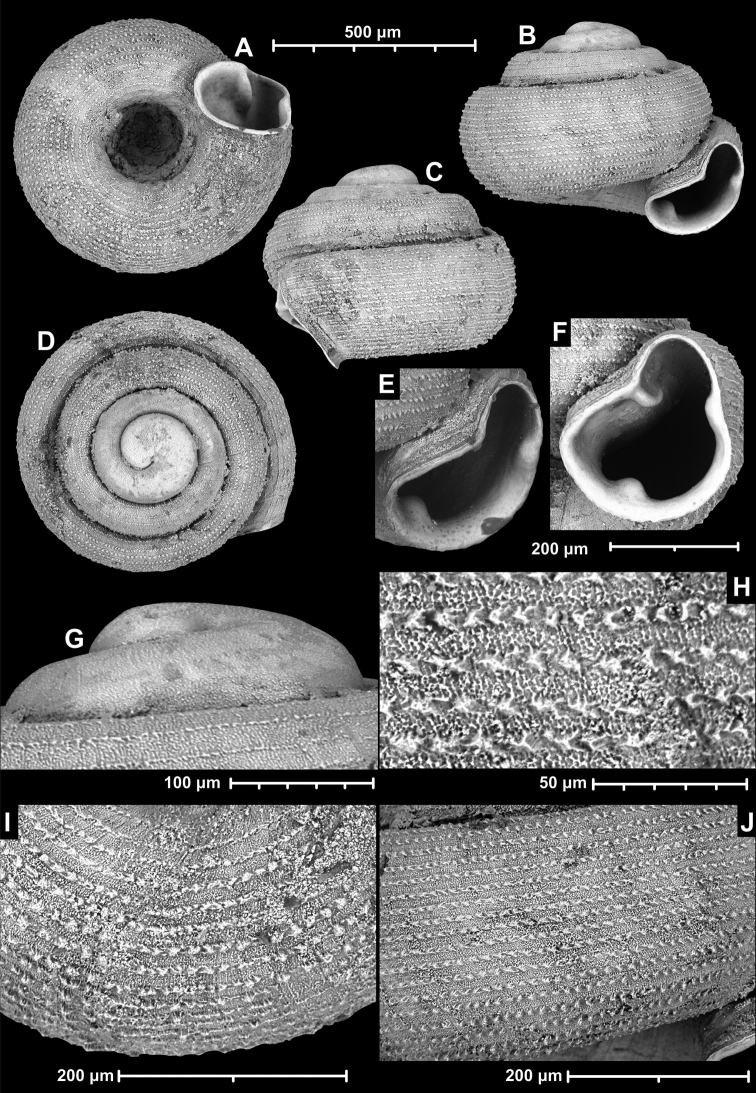
*Angustopilacoprologosuninodus* Páll-Gergely & Grego, ssp. nov. (holotype, NHMUK 20170295). Ventral (**A**), apertural (**B**), lateral (**C**), and apical (**D**) sides of the shell; aperture (**E–F**); sculpture of the protoconch (**G**), fine, chain-like sculpture of the body whorl (**H**), ventral (**I**) and frontal (**J**) surface of the body whorl.

##### Measurements (in mm).

H = 0.52–0.55, D = 0.7–0.75, H/D*100 = 73.3–77.1 (*n* = 3: JG12), RUD = 28.2–28.8 (*n* = 3).

##### Etymology.

The subspecific name *uninodus* (one node) refers to the single palatal denticle of this subspecies, instead of the two palatal teeth of the nominotypical subspecies.

##### Distribution.

This subspecies is known from three nearby localities in Bolikhamsay Province, Laos. The type locality of the nominotypical subspecies is located on a mountain ca. 14 km to the southeast (Fig. 64).

#### ﻿ Angustopila hyron

Taxon classificationAnimaliaStylommatophoraGastrocoptidae

Páll-Gergely & Vermeulen sp. nov.

3E185668-2983-5765-BB61-21BB995360C7

https://zoobank.org/1BBC0CCE-F3AE-42C3-BBE0-CDB17A8AE0BF

Fig. 89

##### Type material.

***Holotype***: Vietnam • 1 empty shell (H: 0.8 mm, D: 0.8 mm); Quang Ninh Province, Halong Bay area, unnamed island in Dau Moi Temple area (locality code: WMVT.0338); 20°55.69'N, 107°09.40'E; 13 Sep. 2003; W.J.M. Maassen leg.; RMNH.5006716.

***Paratypes***: Vietnam • 22 shells; Quang Ninh Province, Halong-Campha area, 4.5 km SW Quang Hanh (locality code: JJV6229); 20°58.98'N, 107°11.83'E; 29 Sep. 1998; J.J. Vermeulen & A.J. Whitten leg.; JJV6229 • 2 shells, same data as for preceding; coll. HA.

##### Additional material.

Vietnam • 1 shell (has only a palatal tooth and appears to be more triangular); same data as for holotype; RMNH.347776.

##### Diagnosis.

A small *Angustopila* species with a domed, conical-globular shell, strong parietal tooth, and two deeply set upper palatal and subcolumellar teeth.

##### Description.

Shell small for the genus, slightly higher than wide or slightly wider than high; colourless, translucent when fresh, dorsal side conical-globular to domed; body whorl widest in standard apertural view; protoconch consists of 1.5 whorls, with very slight indication of spiral striation preceding the first teleoconch whorl (visible only from lateral, not apical view); teleoconch weakly sculptured by some fine radial growth lines and much stronger, equidistantly-arranged spiral striae (ca. 16 on body whorl from standard apertural view); whorls 4, very slightly shouldered; aperture slightly oblique to shell axis from lateral view; umbilicus wide; aperture pear-shaped, sinulus wide; peristome expanded, not reflected; parietal callus protruding, detached from penultimate whorl; parietal tooth strongly developed, high, perpendicular to parietal side; upper palatal tooth weak, low, deeply-set; subcolumellar tooth also weak, blunt, also situated inside some distance from peristome.

**Figure 89.**
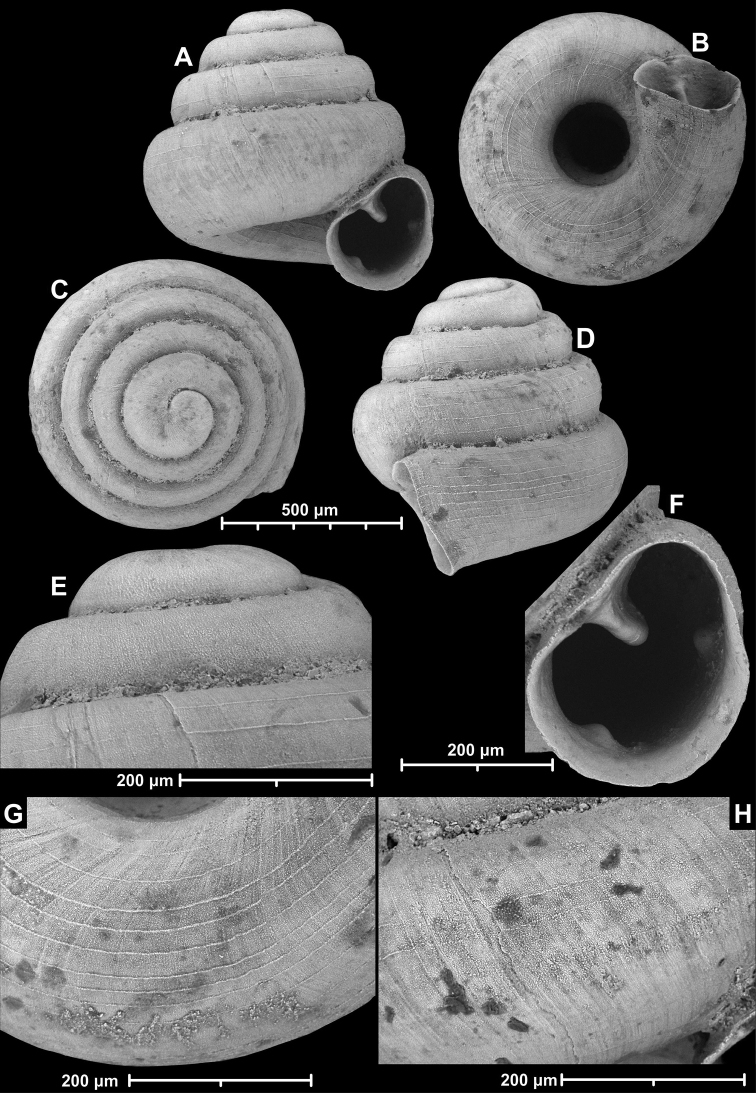
*Angustopilahyron* Páll-Gergely & Vermeulen, sp. nov. (holotype, RMNH.5006716). Apertural (**A**), ventral (**B**), apical (**C**) and lateral (**D**) sides of the shell; sculpture of the protoconch (**E**), aperture (**F**); ventral (**G**) and frontal (**H**) surface of the body whorl.

##### Measurements (in mm).

H = 0.77–0.83, D = 0.75–0.86, H/D*100 = 91.9–106.7 (*n* = 7), RUD = 31.3–32.9 (*n* = 3).

##### Differential diagnosis.

*Angustopilahyron* sp. nov. can be distinguished from all other *Angustopila* species based on the depressed conical-globular shell, the domed form, and the three apertural teeth. The most similar and geographically close, *A.quadridens* sp. nov., is larger, has stronger spiral striation and bears four teeth, which are more pronounced than those in *A.hyron* sp. nov. Although *Angustopilacoprologosuninodus* ssp. nov. also bears three denticles, it differs from *A.hyron* in that it is smaller, more depressed, and has a more prominent sculpture.

##### Etymology.

The specific epithet *hyron* (Greek: beehive) refers to the shape of the shell, and to be used as a noun in apposition.

##### Distribution.

This species is known from two geographically adjacent sites in Halong Bay, northern Vietnam (Fig. 24).

#### ﻿ Angustopila quadridens

Taxon classificationAnimaliaStylommatophoraGastrocoptidae

Páll-Gergely & Vermeulen sp. nov.

BD8A0335-6D8C-5DA5-B6A1-6943F516699D

https://zoobank.org/F879A238-01D6-4227-B452-39D9BA896094

Figs 90 , 91

##### Type material.

***Holotype***: Vietnam • 1 empty shell (H: 0.93 mm, D: 0.96 mm); Haiphong Province, Halong Bay area, Cat Ba Island, Cave Qua Vang, around cave entrance, rocky limestone slope with low, rather mature forest; 20°48.64'N, 107°04.64'E; 100 m a.s.l.; 6 Jun. 2017; J.J. Vermeulen & K. Anker leg.; HNHM 105318.

***Paratypes***: Vietnam • 8 shells; same data as for holotype; JJV 16595 • 4 shell; Haiphong Province, Halong Bay area, Cat Ba Island, Cave Qua Vang, inside cave, large, ecologically intact active cave with speleothems; 20°48.64'N, 107°04.64'E; 60 m a.s.l.; 6 Jun. 2017; J.J. Vermeulen & K. Anker leg.; JJV 16592 • 4 shells; Haiphong Province, Halong Bay area, unnamed island off E Coast Cat Ba, south facing bay with beach and densely-vegetated limestone scree slope; 20°45.19'N, 107°04.46'E; 1 Oct. 1998; J.J. Vermeulen & A.J. Whitten leg.; JJV 6216.

##### Diagnosis.

A small to medium-sized, conical-globular *Angustopila* species with a strong parietal tooth, a blunt subcolumellar tooth, and two rather blunt, palatal teeth situated far from the peristome edge.

##### Description.

Shell small- to normal-sized for the genus, slightly higher than wide or slightly wider than high; pale grey, translucent, conical with variable shell height; body whorl widest from standard apertural view; protoconch consists of ca. 1.25–1.5 whorls, with strong spiral structure on the entire protoconch; teleoconch with some (mostly weak but occasionally stronger) irregular radial growth lines and equidistantly-arranged spiral striae (ca. 15–20 on body whorl from standard apertural view); whorls 4–4.25, slightly shouldered; aperture only very slightly oblique to shell axis from lateral view; umbilicus wide; aperture pear-shaped, sinulus relatively wide; peristome expanded, not reflected; parietal callus protruding, detached from penultimate whorl; parietal tooth prominent, high, perpendicular to parietal side, almost reaching edge of peristome; palatal wall with two strong palatal teeth (upper and lower palatal teeth), situated in large distance from peristome; subcolumellar tooth well-developed, blunt, nearly reaching peristome edge.

##### Measurements (in mm).

H = 0.81–1.05, D = 0.81–0.96, H/D*100 = 94.3–113.5 (*n* = 8), RUD = 29.7–32.3 (*n* = 3).

##### Differential diagnosis.

*Angustopilaquadridens* sp. nov. can be distinguished from all other *Angustopila* species based on the relatively large, domed shell, and the four apertural teeth. For differences with the most similar *A.hyron* sp. nov. and *A.tetradon* sp. nov., see under those species.

##### Etymology.

The specific epithet refers to the four teeth of this new species.

##### Distribution.

This species is known from two geographically adjacent sites in the Halong Bay Area: Qua Vang Cave on Cat Ba Island, and ca. 6.4 km south on a small unnamed island (Fig. 24).

##### Remarks.

Shells of sample JJV 6216 are slightly smaller and have a lower spire than those of the type lot at the entrance of the Cave Qua Vang. However, other shells collected inside the cave also bear lower spires. Thus, we consider this trait to be intraspecifically variable.

**Figure 90.**
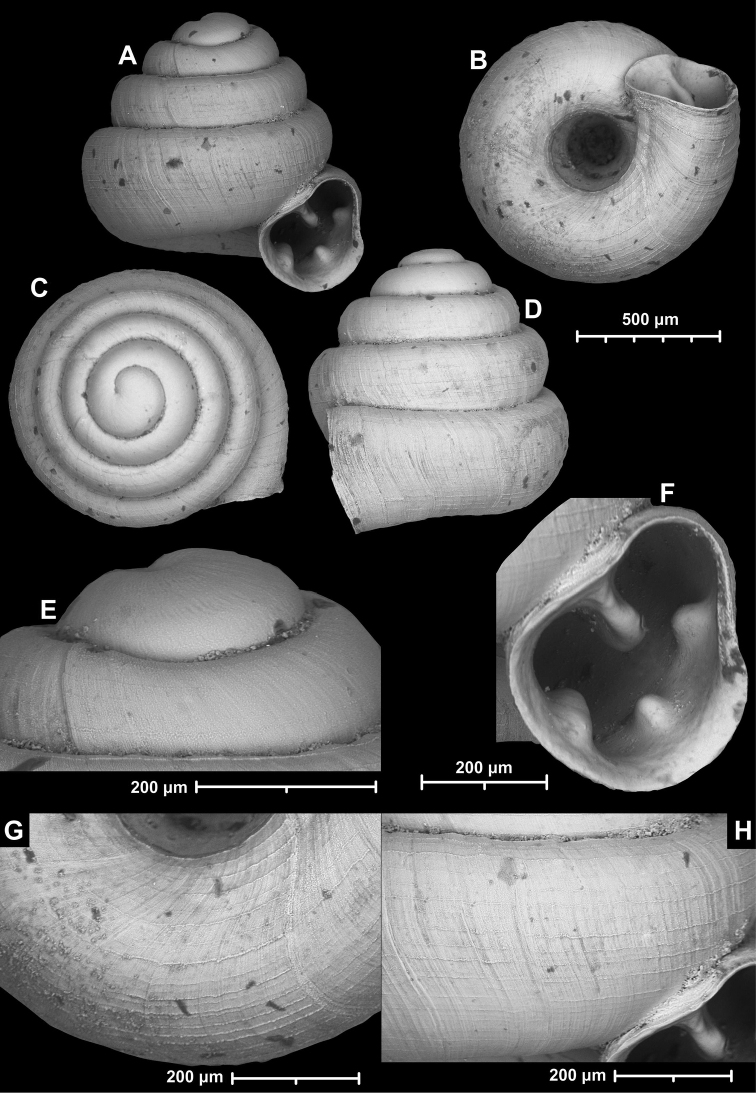
*Angustopilaquadridens* Páll-Gergely & Vermeulen, sp. nov. (holotype, HNHM 105318). Apertural (**A**), ventral (**B**), apical (**C**) and lateral (**D**) sides of the shell; sculpture on the protoconch with protoconch-teleoconch boundary (**E**), aperture (**F**), ventral (**G**) and frontal (**H**) surface of the body whorl.

**Figure 91.**
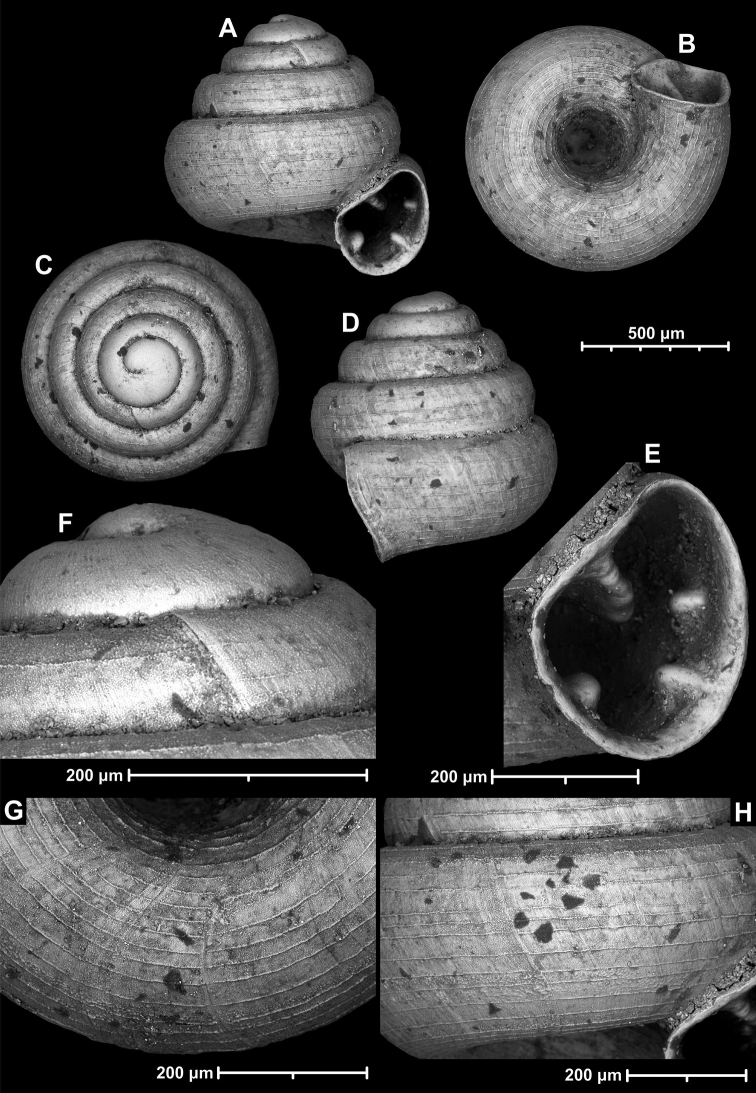
*Angustopilaquadridens* Páll-Gergely & Vermeulen, sp. nov., JJV 6216. Apertural (**A**), ventral (**B**), apical (**C**) and lateral (**D**), sides of the shell; aperture (**E**); sculpture on the protoconch with protoconch-teleoconch boundary (**F**), ventral (**G**) and frontal (**H**) surface of the body whorl.

#### ﻿ Angustopila rara

Taxon classificationAnimaliaStylommatophoraGastrocoptidae

Páll-Gergely & Hunyadi sp. nov.

E7E07CB4-8AFD-5027-9F38-7FAF27D39396

https://zoobank.org/F90E5718-275A-4DC4-A6DA-C70FB85150E6

Fig. 92

##### Type material.

***Holotype***: Vietnam • 1 empty shell (H: 0.8 mm; D: 0.72 mm); Thanh Hóa Province, 3.6 km northwest from centre of Ngọc Lặc on road no. 15, 600 m north from Làng Săt, rock wall (locality code: 2020/35); 20°05.90'N, 105°21.58'E; 55 m a.s.l.; 12 Feb. 2020; A. Hunyadi leg.; HNHM 105319.

##### Diagnosis.

A small, conical *Angustopila* species with a narrow umbilicus, few (13) spiral striae on the body whorl, and strong apertural barriers (elevated parietal, rather pointed palatal, rather blunt columellar).

##### Description.

Shell small for the genus, higher than wide; off-white, conical with slowly increasing whorls; body whorl widest from standard apertural view; protoconch consists of nearly 1.5 whorls, with slight indication of spiral striation at the end; teleoconch with some fine, weak, irregular radial growth lines and much stronger, elevated, widely-spaced spiral striae (ca. 10 or 11 on body whorl from standard apertural view); whorls 4, rounded; aperture only slightly oblique to shell axis from lateral view; umbilicus moderately wide; aperture broadly heart shaped (although the palatal region of the holotype is slightly broken) with straight parietal side; peristome expanded, not reflected; parietal callus not detached from penultimate whorl; parietal tooth elevated, straight, tongue-like with narrower (slender) base and thickened (widened) end (i.e., the cross sectional view of the parietal lamella is club-shaped); palatal tooth situated in the middle of the palatal side, small, denticle-like, situated close to basal lip; columellar tooth situated on the lower portion of the columellar area, as high as palatal tooth but more elongated, and thus, seemingly more blunt.

##### Measurements (in mm).

H = 0.8; D = 0.72, H/D*100 = 111.1, RUD = 29.6 (holotype).

##### Differential diagnosis.

*Angustopilarara* sp. nov. is most similar to *A.tridentata* sp. nov. in the formation of the apertural barriers. However, it is much smaller, has a conical rather than a concave-conical shell shape, a much narrower umbilicus (this trait is not independent from the previous one) and fewer spiral striae on the body whorl. The columellar tooth of *A.rara* sp. nov. is situated lower (i.e., closer to the basal region) than that in *A.tridentata* sp. nov., which resembles a low wart at the middle of the columellar peristome. Among the species possessing two well-developed teeth (parietal + palatal), *A.antidomedon* sp. nov., *A.reticulata* sp. nov., and *A.tweediei* sp. nov. may have an additional, very weak (sub)columellar tooth. Those species differ from *A.rara* sp. nov. in several other conchological characters. See also under *A.apokritodon* sp. nov.

**Figure 92.**
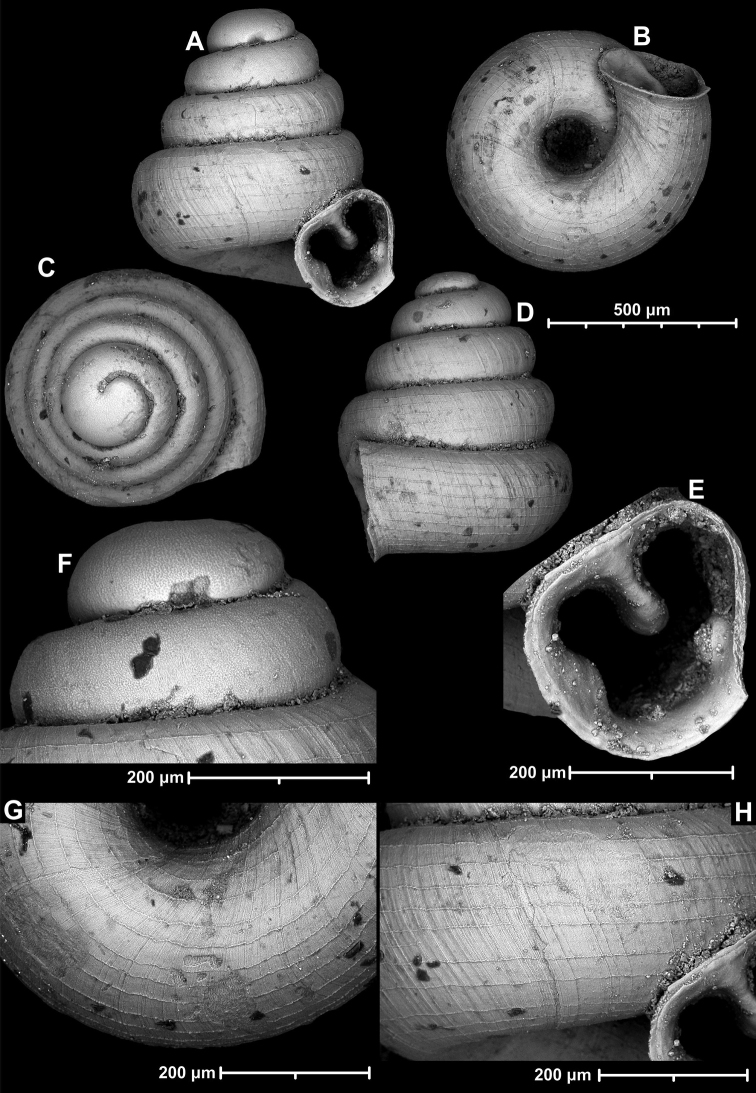
*Angustopilarara* Páll-Gergely & Hunyadi, sp. nov. (holotype, HNHM 105319). Apertural (**A**), ventral (**B**), apical (**C**) and lateral (**D**) sides of the shell; aperture (**E**); sculpture on the protoconch (**F**), ventral (**G**) and frontal (**H**) surface of the body whorl.

##### Etymology.

The specific epithet *rara* (rare in Latin) refers to the rarity of this species, as only a single shell is known.

##### Distribution.

This species is known from the type locality only (Fig. 27).

#### ﻿ Angustopila tetradon

Taxon classificationAnimaliaStylommatophoraGastrocoptidae

Páll-Gergely & Hunyadi sp. nov.

CCD43665-80D9-5139-BA3F-3FC55EFC40D5

https://zoobank.org/F84D6F13-0CC2-4D02-955A-1C4549C121B7

Figs 93 , 94

##### Type material.

***Holotype***: Vietnam • 1 empty shell (H: 0.82 mm, D: 0.79); Thanh Hóa Province, Như Thanh District, Hải Vân, Hang Lò Cao Kháng Chiến, vicinity of the cave (locality code: 2020/41); 19°37.08'N, 105°34.63'E; 20 m a.s.l.; 14 Feb. 2020; A. Hunyadi leg.; HNHM 105320.

***Paratypes***: Vietnam • 76 shells; same data as for holotype; coll. HA • 3 shells; same data as for holotype; coll. JJV • 48 shells; Thanh Hóa Province, Như Thanh District, 600 m south from Xuân Khang along road no. 45, around the cave temple (locality code: 2020/40); 19°40.35'N, 105°31.07'E; 80 m a.s.l.; 14 Feb. 2020; A. Hunyadi leg.; coll. HA.

##### Additional material.

Vietnam • 22 j/b shells; same data as for holotype; coll. HA • 1 j/b shell; Thanh Hóa Province, Như Thanh District, 600 m south from Xuân Khang along road no. 45, around the cave temple, (locality code: 2020/40); 19°40.35'N, 105°31.07'E; 80 m a.s.l.; 14 Feb. 2020; A. Hunyadi leg.; coll. HA.

##### Diagnosis.

A small, conical *Angustopila* species with a strong parietal tooth, a strong subcolumellar tooth, and two strong palatal teeth situated relatively close to peristome edge.

##### Description.

Shell small for the genus, slightly higher than wide or slightly wider than high; colourless, translucent, conical-globular with a high-domed spire; body whorl widest from standard apertural view; protoconch consists of ca. 1.5 whorls, with weak signs of spiral striation preceding the first teleoconch whorl; teleoconch with some fine, irregular radial growth lines and much stronger, equidistantly-arranged spiral striae (ca. 15–17 on body whorl from standard apertural view); whorls 3.75–4, slightly shouldered; aperture slightly curved and in line with shell axis in lateral view; umbilicus wide; aperture subquadrate, sinulus broad; peristome expanded, not reflected; parietal callus not protruding, not detached from penultimate whorl; parietal tooth prominent, high, perpendicular to parietal side or slightly bent, almost reaching edge of peristome; palatal wall with two rather low but prominent palatal teeth (upper and lower palatal teeth), situated in rather small distance from peristome; subcolumellar tooth low, blunt, nearly reaching peristome edge.

**Figure 93.**
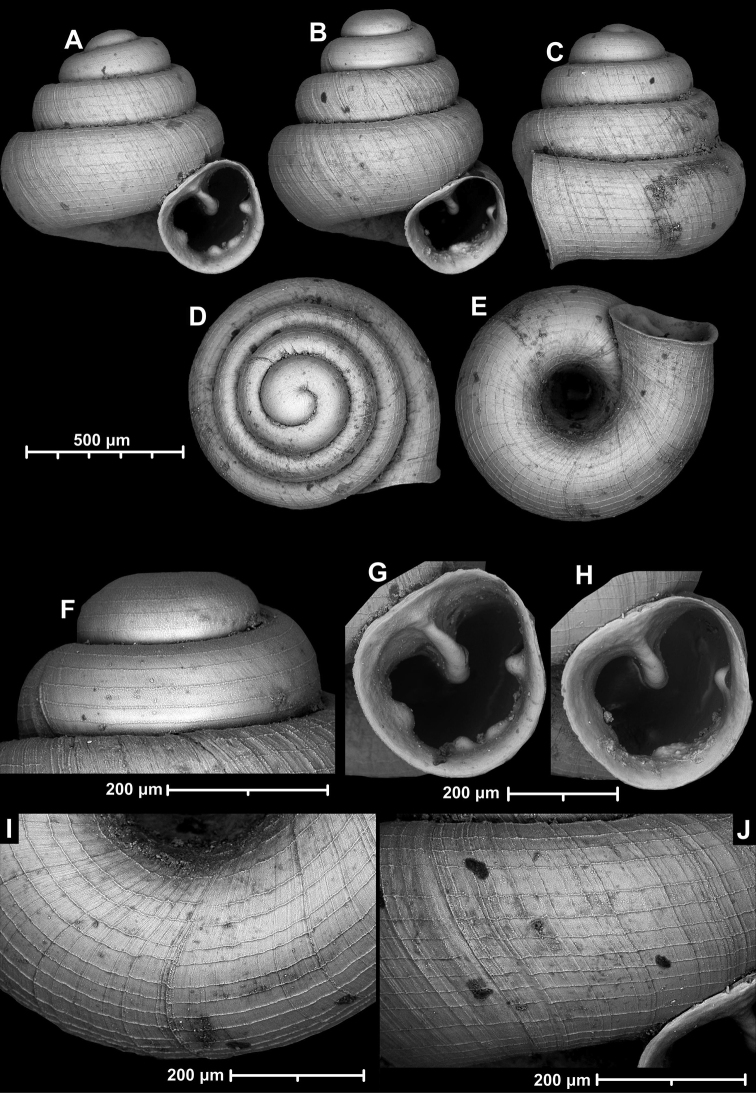
*Angustopilatetradon* Páll-Gergely & Hunyadi, sp. nov. Paratype (**A, G**) and holotype (HNHM 105320) (**B–F, H–J**). Apertural (**A, B**), lateral (**C**), apical (**D**) and ventral (**E**) sides of the shell; sculpture on the protoconch (**F**); aperture (**G, H**); ventral (**I**) and frontal (**J**) surface of the body whorl.

**Figure 94.**
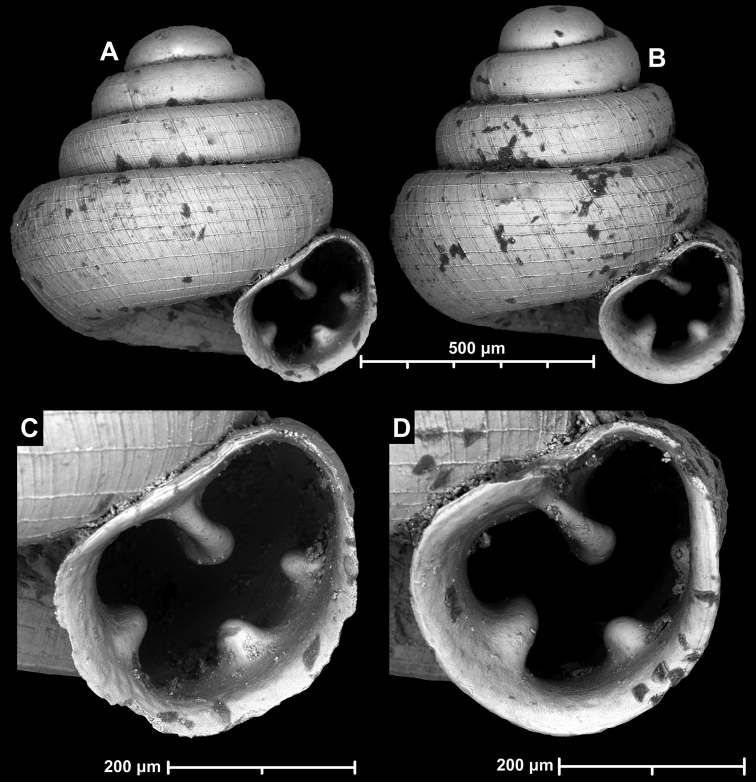
*Angustopilatetradon* Páll-Gergely & Hunyadi, sp. nov., sample 2020/40. Specimen 1 (**A, C**) and Specimen 2 (**B, D**).

##### Measurements (in mm).

H = 0.73–0.87, D = 0.74–0.84, H/D*100 = 92.8–113 (*n* = 14), RUD = 26.3–30.8 (*n* = 4).

##### Differential diagnosis.

The upper palatal tooth of *A.quadridens* sp. nov. is situated deeper, its subcolumellar tooth is more elevated, and its sinulus is narrower resulting in a more elongated aperture shape. Moreover, the shells of *A.tetradon* sp. nov. are more conical (instead of conical-globular) than those of *A.quadridens* sp. nov.

##### Etymology.

The specific epithet refers to the four apertural barriers of this new species (tetradon: *τετρα* = four, and *ὀδούς* = tooth).

##### Distribution.

This species is known from two geographically adjacent sites in Thanh Hóa Province, northern Vietnam (Fig. 55).

#### ﻿ Angustopila tridentata

Taxon classificationAnimaliaStylommatophoraGastrocoptidae

Páll-Gergely & Hunyadi sp. nov.

26BF2BBD-0FF1-5342-AACF-7E8B6DF4E131

https://zoobank.org/477B871F-CCA1-478C-985B-7FE20BBBA22A

Figs 95 , 96

##### Type material.

***Holotype***: Vietnam • 1 empty shell (H: 0.92 mm, D: 0.9 mm); Hòa Bình Province, Kim Bȏi District, Cao Dương, north of Đồng Phú, 58 km from Nho Quan towards Hanoi on the Hồ Chí Minh road (locality code: 2020/47); 20°42.59'N, 105°39.30'E; 10 m a.s.l.; 16 Feb. 2020; A. Hunyadi leg.; HNHM 105321.

***Paratypes***: Vietnam • 2 figured shells; same data as for holotype; HNHM 105322 • 87 shells; same data as for holotype; coll. HA • 3 shells; same data as for holotype; coll. JJV.

##### Additional material.

Vietnam • 21 j/b shells; same data as for holotype; coll. HA.

##### Diagnosis.

A medium-sized, concave-conical *Angustopila* species with a wide umbilicus, and three prominent apertural barriers (elevated parietal, pointed upper palatal, blunt, elongated columellar).

##### Description.

Shell small- to medium-sized for the genus, slightly higher than wide or slightly wider than high; off-white, concave-conical with regularly growing whorls except for the last one; body whorl widest from standard apertural view; protoconch consists of 1.5 whorls, with slight indication of spiral striation preceding the first teleoconch whorl; teleoconch with some fine, weak, irregular radial growth lines and much stronger, elevated, spiral striae of variable density (ca. 9–16 on body whorl in standard apertural view); whorls 4–4.5, rounded; aperture only slightly oblique to shell axis from lateral view; umbilicus wide; aperture suboval with straight parietal part; peristome expanded, not reflected; parietal callus not detached from penultimate whorl; parietal tooth elevated, mostly straight; palatal tooth situated in the middle of the palatal wall (probably homologous with the upper palatal tooth), small, denticle-like, situated close to basal lip; columellar tooth situated in the middle of columellar area, runs along peristome, lower and longer than palatal tooth.

**Figure 95.**
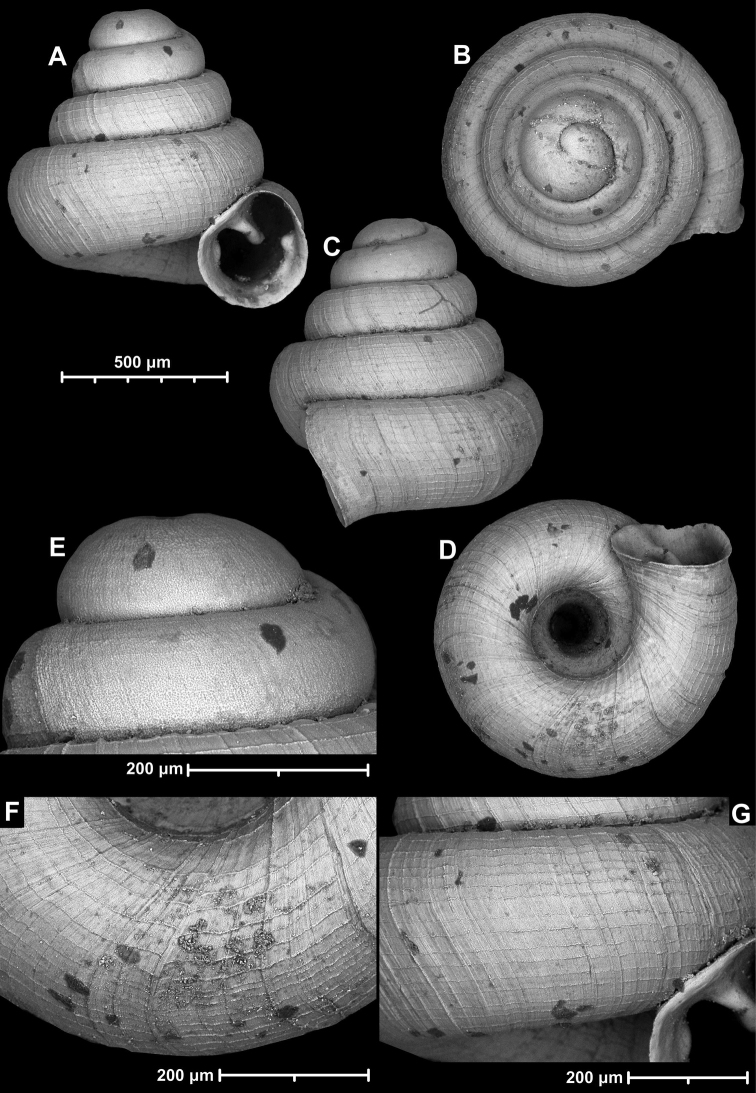
*Angustopilatridentata* Páll-Gergely & Hunyadi, sp. nov. (holotype, HNHM 105321). Apertural (**A**), apical (**B**), lateral (**C**) and ventral (**D**) sides of the shell; sculpture on the protoconch (**E**), ventral (**F**) and frontal (**G**) surface of the body whorl.

**Figure 96.**
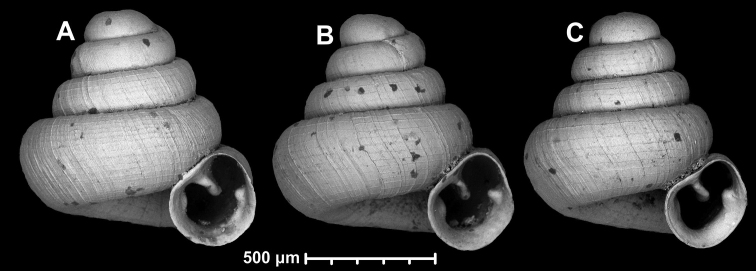
Variability of *Angustopilatridentata* Páll-Gergely & Hunyadi, sp. nov. **A** holotype (specimen 3) **B** specimen 2 **C** specimen 1.

##### Measurements (in mm).

H = 0.76–0.99; D = 0.82–0.95, H/D*100 = 91.6–112.5 (*n* = 9), RUD = 32.9–34.1 (*n* = 3).

##### Differential diagnosis.

See under *A.apiaria* sp. nov. and *A.rara* sp. nov. Some other species that normally have two apertural barriers may have a third one, see differential diagnosis under *A.rara* sp. nov.

##### Etymology.

This new species is named after its three apertural barriers.

##### Distribution.

This species is only known from the type locality of Cao Dương in Hòa Bình Province, Vietnam (Fig. 27).

## ﻿Results and discussion

### ﻿Species defined based on morphology

Here we described 42 species and one subspecies of the genus *Angustopila* new to science, which up to now, comprised only 13 species, of which two are synonymised with other species in this work. All *Angustopila* species in this and in previous studies are defined and distinguished from each other based on morphological (conchological) traits. Multi-gene molecular phylogeny could provide strong support for the morphology-based species hypotheses; however, only three *Angustopila* species (6% of all known species) have ever been found alive, and it would be nearly impossible to elevate this to a reasonably high number, given that the species in question are subterranean and cave-dwelling. Therefore, the lack of living specimens makes phylogenetic studies clearly impossible. Even if living specimens were present, phylogenetic work would be too time-consuming and expensive, especially given that taxonomy and systematics are poorly funded worldwide (Wheeler 2004).

While academic positions for taxonomists are being lost (Engel et al. 2021), a considerable number of new species are being described by amateur scientists (Fontaine et al. 2012), who have little opportunity to support their hypotheses by means of phylogenetic information (Bouchet et al. 2016). In fact, only less than 10% of newly described molluscan species are described with any kind of sequence information (Bouchet et al. 2016), and obviously, a much smaller percentage of species are described with molecular phylogenetic data sufficient enough to be informative in species level taxonomy. At the same time, we are experiencing the sixth mass extinction in the history of our planet (Ceballos et al. 2015), and only a small percentage of the living terrestrial species has already been described (Mora et al. 2011). Terrestrial molluscs (Lydeard et al. 2004; Regnier et al. 2009; Richling and Bouchet 2013), especially those inhabiting limestone outcrops (Clements et al. 2006; Hughes 2017), are specifically highly impacted. Under such dire circumstances, delaying description for better DNA access would significantly slow down description of new species, which is the first step towards their recognition and protection. Each species is a hypothesis (Wheeler 2004), based on unique traits or unique combinations of characters, and these hypotheses can be subsequently tested by means of molecular phylogeny or any other methods. Here we emphasize the importance of describing morphologically recognisable species to foster high awareness of the stunning biodiversity of small faunas before they disappear.

### ﻿Species diversity

The number of observed species, the number of single-site endemic species, and the estimated number of possible species using Chao2-bc and iChao2 estimators (with 95% confidence intervals) are compiled in Table 3. The Chao2-bc and iChao2 models estimated 80 and 91 *Angustopila* species in total with 64–123 and 73–127 species as 95% confidence intervals, respectively.

**Table 3.** The number of observed species, the number of single-site endemic species, and the estimated number of possible species using Chao2-bc and iChao2 estimators (with 95% confidence intervals) in the entire area and in the three relatively well-studied sites corresponding with Fig. 1.

	Entire area	Annamite Mts	Central Laos	Halong Bay
observed species	54	18	8	14
single site endemics	28	13	5	2
**Chao2-bc Estimate**	**81**	**55**	**17**	**14**
Chao2-bc 95%Lower	64	28	10	14
Chao2-bc 95%Upper	123	162	59	18
**iChao2 Estimate**	**91**	**99**	**18**	**14**
iChao2 95%Lower	73	32	11	14
iChao2 95%Upper	127	482	50	19

Previously, 13 *Angustopila* species were known from 13 sites. The exact match between the number of species and the number of localities is merely coincidental. Namely, in a few cases, more than one species was described from a single site, and some species were known from more than a single site. The data presented here revealed as many as 53 species from 223 sites. Three species (*A.elevata*, *A.fabella*, *A.szekeresi*) have a wide distributional area, three species are known from two localities that are situated far (280–500 km) from each other, 19 species are small range endemics (5–240 km, but typically less than 60 km), and 28 species are single-site endemics (Table 4). The presence of widely distributed species also indicates that for the description of a new microsnail, it is critical to compare any potential new species with wide range species to establish its novelty.

**Table 4.** Distribution types of *Angustopila* species.

(Sub)species	Distribution	Distribution types	Area width (km)
*A.akrodon* sp. nov.	northern Vietnam	single site	n.a.
*A.antidomedon* sp. nov.	northern Vietnam	single site	n.a.
*A.apiaria* sp. nov.	central Vietnam	single site	n.a.
*A.apiostoma* sp. nov.	northern Vietnam	small range	28
*A.apokritodon* sp. nov.	northern Vietnam	single site	n.a.
*A.babel* sp. nov.	northern Vietnam	small range	28
*A.bathyodon* sp. nov.	central Laos	single site	n.a.
*A.bidentata* sp. nov.	central Laos	small range	34
*A.cavicola* sp. nov.	northern Thailand, northern Laos (?)	disjunct	320
*A.cicatricosa* sp. nov.	northern Vietnam	single site	n.a.
*A.concava*	Thailand	single site	n.a.
*A.coprologoscoprologos*	central Laos	single site	n.a.
*A.coprologosuninodus* ssp. nov.	central Laos	small range	5
*A.dominikae*	Guangxi (China)	single site	n.a.
*A.elevata*	widest in the genus: southern Thailand to Guangxi	wide	2060
*A.erawanica* sp. nov.	Thailand	single site	n.a.
*A.fabella*	northern Laos, northern Vietnam, Guangxi	wide	640
*A.fratermajor* sp. nov.	northern Vietnam	small range	220
*A.fraterminor* sp. nov.	northern Vietnam	small range	14
*A.gracilis* sp. nov.	central Laos	single site	n.a.
*A.halongensis* sp. nov.	northern Vietnam	small range	22
*A.huoyani*	Guangxi, Hunan (China)	disjunct	500
*A.hyron* sp. nov.	northern Vietnam	small range	7
*A.maasseni* sp. nov.	northern Vietnam	small range	6
*A.majuscula* sp. nov.	northern Thailand	small range	40
*A.margaritarion* sp. nov.	northern Laos	small range	23
*A.megastoma* sp. nov.	northern Vietnam	small range	23
*A.milium*	northeastern India	single site	n.a.
*A.occidentalis* sp. nov.	northern Thailand, central Myanmar	small range	240
*A.oostoma* sp. nov.	northern Vietnam	small range	56
*A.pallgergelyi*	central Thailand	single site	n.a.
*A.papaver* sp. nov.	northern Vietnam	single site	n.a.
*A.parallela* sp. nov.	northern Vietnam	disjunct	280
*A.prolixa* sp. nov.	northern Vietnam	single site	n.a.
*A.psammion*	northern Vietnam	single site	n.a.
*A.pusilla* sp. nov.	northern Laos	single site	n.a.
*A.pustulata* sp. nov.	northern Vietnam	single site	n.a.
*A.quadridens* sp. nov.	northern Vietnam	small range	6
*A.rara* sp. nov.	northern Vietnam	single site	n.a.
*A.reticulata* sp. nov.	northern Vietnam	single site	n.a.
*A.somsaki* sp. nov.	northern Thailand	small range	140
*A.steffeki* sp. nov.	central Laos	single site	n.a.
*A.szekeresi*	northern Laos, northern Vietnam, Guangxi	wide	650
*A.tamlod*	northern Thailand	single site	n.a.
*A.tetradon* sp. nov.	northern Vietnam	small range	9
*A.thersites* sp. nov.	northern Vietnam	small range	39
*A.tonkinospiroides* sp. nov.	northern Vietnam	single site	n.a.
*A.tridentata* sp. nov.	northern Vietnam	single site	n.a.
*A.tweediei* sp. nov.	northern Vietnam	single site	n.a.
*A.uvula* sp. nov.	southern Thailand	single site	n.a.
*A.vandevenderi* sp. nov.	northern Vietnam	single site	n.a.
*A.vitrina* sp. nov.	northern Vietnam	single site	n.a.
*A.vomer* sp. nov.	northern Vietnam	single site	n.a.
*A.werneri* sp. nov.	northern Vietnam	small range	21

In the species diversity models we used, as the number (proportion) of single-size endemic species increases, and the species found in multiple sites increases, the standard error and the 95% confidence interval increases. In the Halong Bay area, where single-site endemics were a minority, both models predicted approximately as many species as we found with the low 95% confidence interval. On the other hand, in the Annamite Mountains, where many single-site endemic species were found, the models predicted far more species with extremely high confidence intervals. Central Laos was somewhat intermediate between the two other sites. Similarly, for the entire distributional area of the genus *Angustopila*, the models could only predict much more species than observed with extremely high confidence intervals.

The sites with multiple (2–4) *Angustopila* species reveal that this genus is most diverse in northern Vietnam, northern Laos, and the neighbouring Chinese Guangxi Province and Myanmar. The number of *Angustopila* species per site can be as high as six (site 2020/41, see Fig. 99) and seven (Qua Vang Cave in the Halong Bay, see Fig. 100). Both these high incidences derive from northern Vietnam, an indication that this region is the very centre of the diversity of *Angustopila* (Fig. 101). We remark that we examined relatively few samples from Guangxi, where the diversity of *Angustopila* might be richer than we currently know.

The ca. 600 km^2^ area of the Halong Bay (calculated using Google Earth Pro, including areas covered by water) was investigated intensively by JJV and W.J.M. Maassen using soil samples. As a result, *Angustopila* species were found in 30 sites within this area. Amongst other microsnail genera (see Páll-Gergely et al. 2019, 2020), the Halong Bay region is inhabited by 14 species of *Angustopila* (Fig. 102), some of them are single-site endemics, others are widespread across the area, and one species (*A.fabella*) occupies the largest area amongst all *Angustopila* species. These results show that even such a study, incorporating dozens of new species, that we are still scratching the surface if the sampling is not dense enough.

*Angustopilamilium* (Benson, 1853) was originally described as a *Cyclostoma* (caenogastropod) species. This and other cases (Páll-Gergely 2020; Páll-Gergely et al. 2020) indicate that microsnails could have been classified into groups in which they do not systematically belong due to the combination of insufficient knowledge regarding conchological characters of certain snail groups and poor quality of microscopes way back when. Therefore, it seems inevitable that we will continue to find more members of hypselostomatid species still historically classified within caenogastropod groups.

### ﻿Relation of shell shape to size

**Figure 97.**
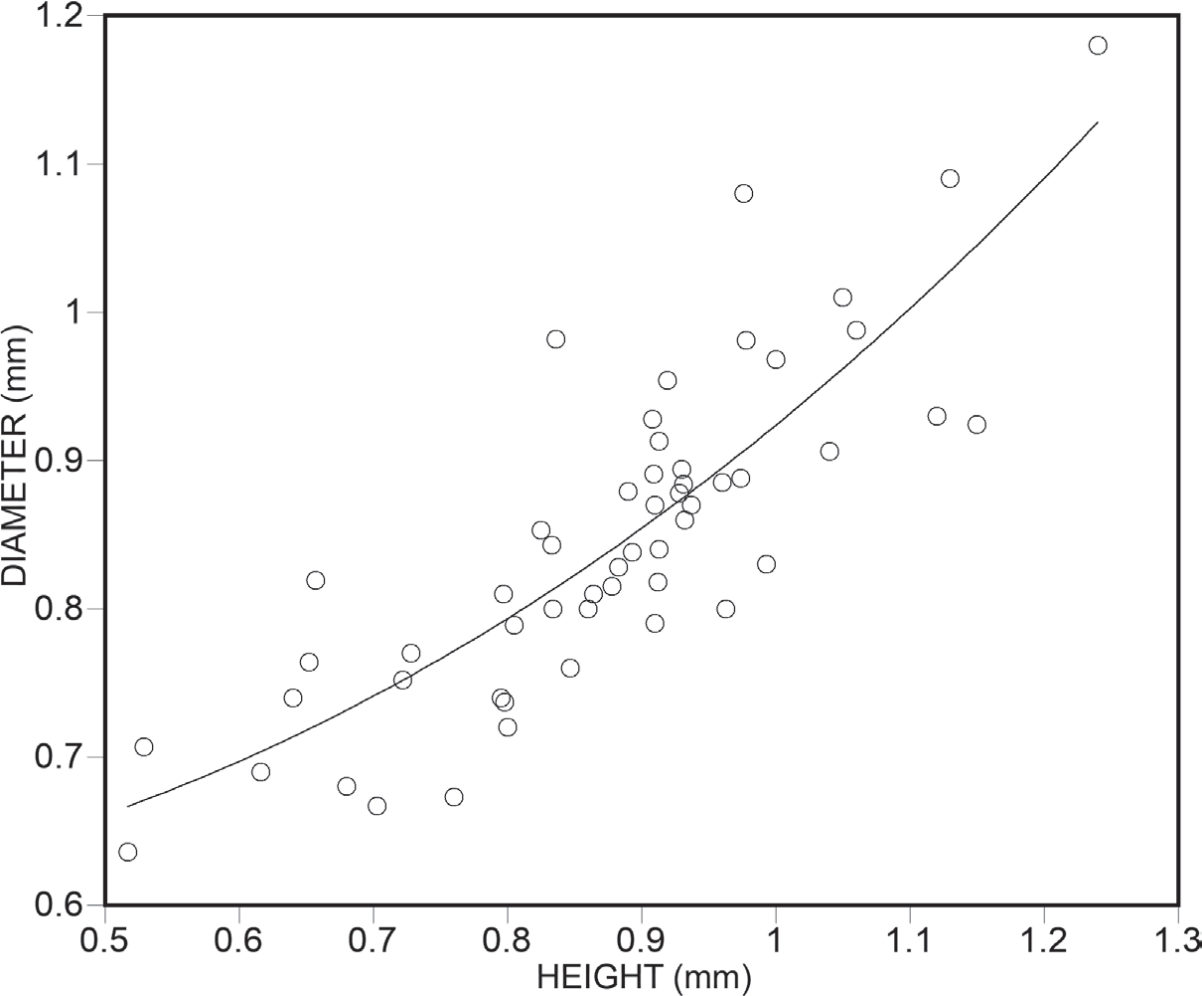
Interdependence of shell dimensions for all *Angustopila* species. The plotted values are the means (when more than one measurement was available) for each species. The fitted curve has the equation D = 0.59 + 0.33H^2.2^ (r^2^ = 0.70).

**Figure 98.**
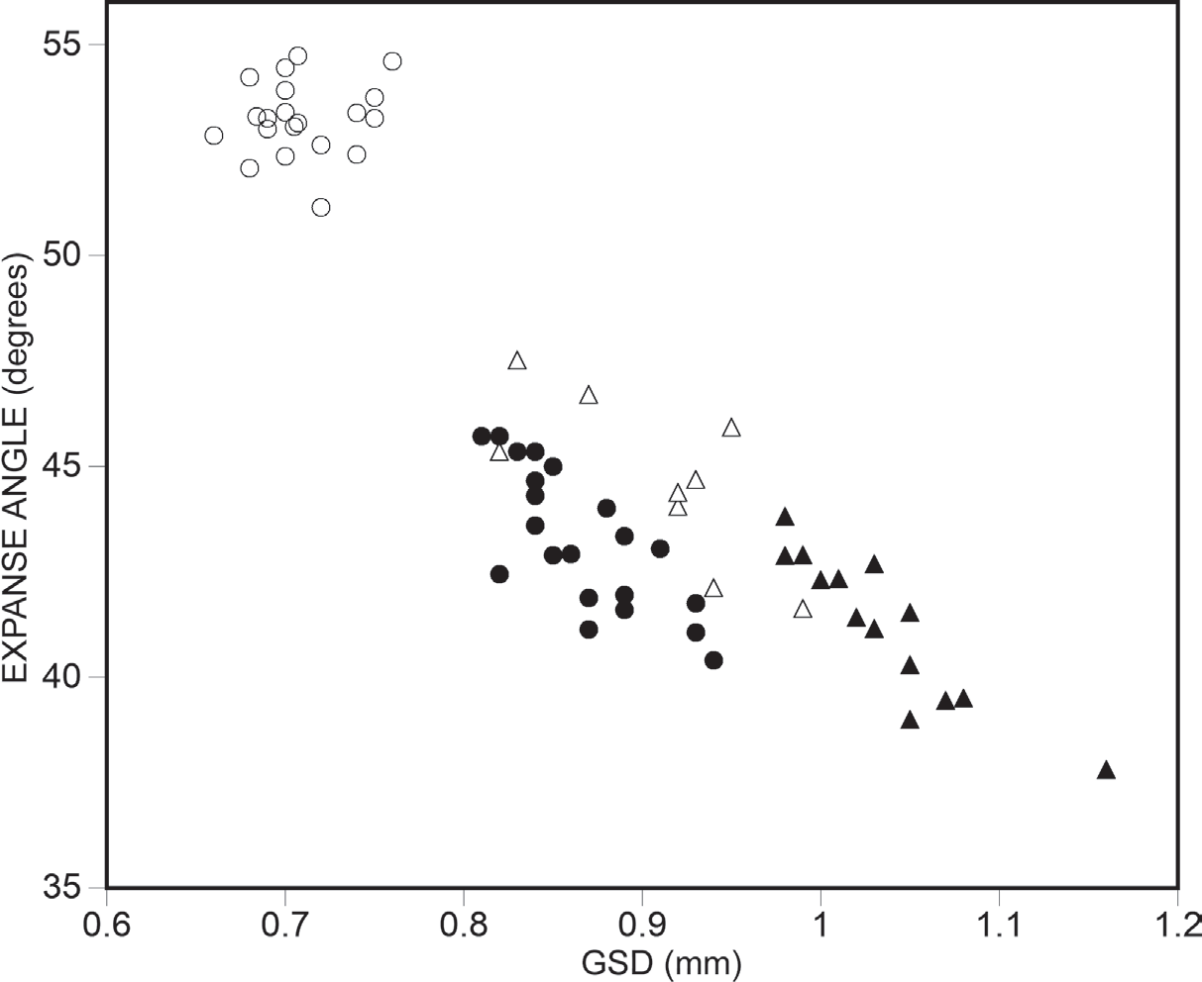
Relation of the expanse angle on the greater shell dimension (GSD) in individual specimens of *A.coprologos* (open circles), *A.tridentata* sp. nov. (open triangles), *A.fratermajor* sp. nov. (closed circles) and *A.tonkinospiroides* sp. nov. (closed triangles).

An analysis of the dependence of shell shape to size in more than 2400 genera of stylommatophoran land snails showed that in terms of the mean genus values, all genera with shells smaller than 1.5 mm had wide (D > H) shells (Örstan 2018). The overall trend observed in the shapes of the individual *Angustopila* species agrees with these results (Fig. 97). All *Angustopila* species with shells higher than ca. 1.0 mm have tall (H > D) shells. At shell heights between 0.7 mm and 1.0 mm, tall species still predominate, but several species have either approximately equiaxial (H = D) or wide shells. Below the shell height of ca. 0.7 mm, all species have wide (D > H) shells. One hypothesized constraint of shape in the smallest snails is the requirement that the body whorl be wide enough to accommodate at least one egg with a diameter that is assumed to have reached a minimum possible value in the smallest snails (Örstan 2018; Páll-Gergely et al. 2022). This leads to the prediction that in the smallest species, the shells of smaller adults should be wider than those of larger adults (Örstan 2018). If the shell shape is expressed as the expanse angle (ε obtained by taking the inverse tangent of the ratio D/H (Örstan 2018)), the values of ε are predicted to increase with decreasing greater shell dimension (GSD, larger with height or diameter) within each species. The dependence of ε to GSD in several *Angustopila* species conforms to this prediction (Fig. 98). Especially in species with tall shells (ε < 45°), there is a more or less uniform increase in the value of ε with decreasing GSD, while in species with shells that are already wide (ε > 45°), the correlation is less pronounced or absent as, for example, in *A.coprologos* (Fig. 98).

**Figure 99.**
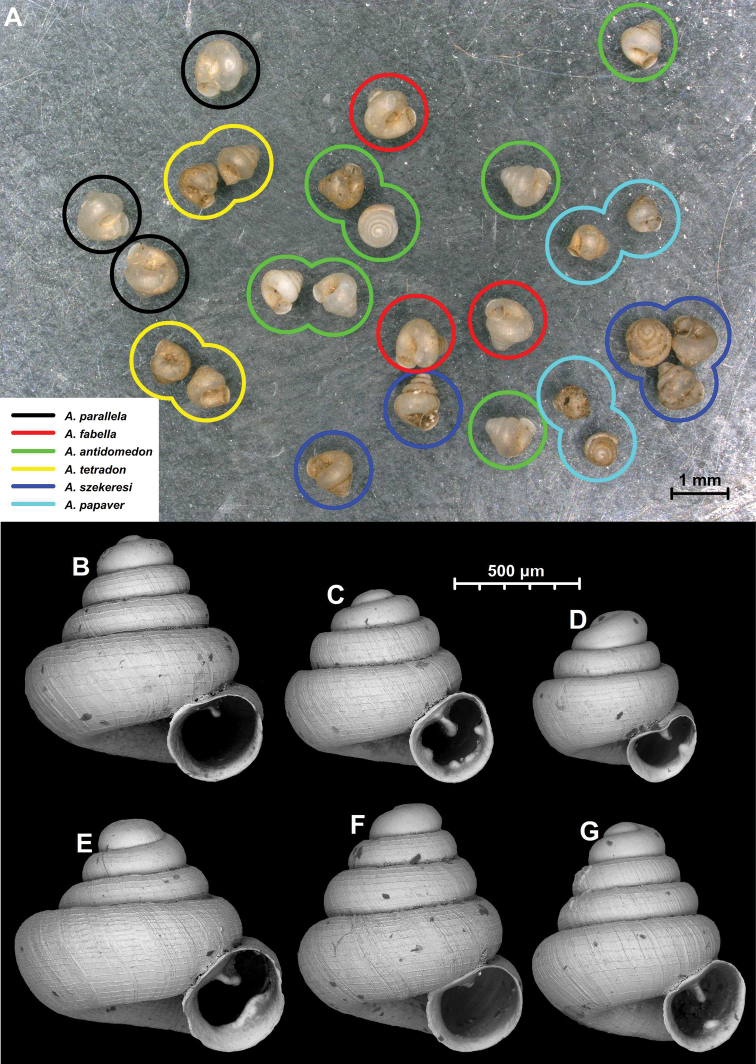
Six sympatric *Angustopila* species collected at Hang Lò Cao Kháng Chiến (Thanh Hóa Province, Vietnam) **A** synoptic photo of shells of all 6 species **B***A.fabella* Páll-Gergely & Hunyadi, 2015 **C***A.tetradon* Páll-Gergely & Hunyadi, sp. nov. **D***A.papaver* Páll-Gergely & Hunyadi, sp. nov. **E***A.parallela* Páll-Gergely & Hunyadi, sp. nov. **F***A.szekeresi* Páll-Gergely & Hunyadi, 2015 **G***A.antidomedon* Páll-Gergely & Hunyadi, sp. nov.

**Figure 100.**
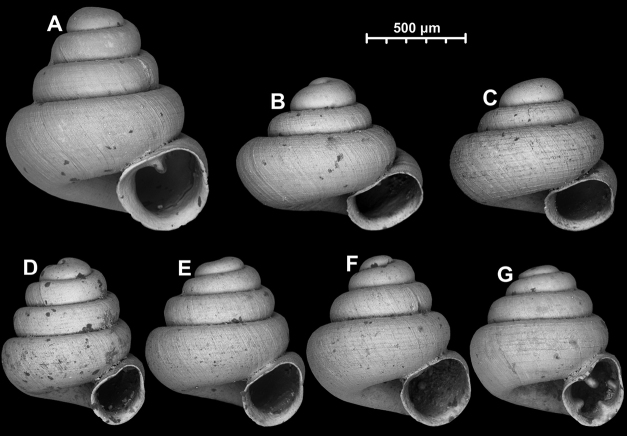
Seven sympatric *Angustopila* species collected in Qua Vang Cave, Cat Ba Island, Halong Bay, Vietnam **A***A.babel* Páll-Gergely & Vermeulen, sp. nov. **B***A.megastoma* Páll-Gergely & Vermeulen, sp. nov. **C***A.thersites* Páll-Gergely & Vermeulen, sp. nov. **D***A.apiostoma* Páll-Gergely & Vermeulen, sp. nov. **E***A.fratermajor* Páll-Gergely & Vermeulen, sp. nov. **F***A.halongensis* Páll-Gergely & Vermeulen, sp. nov. **G***A.quadridens* Páll-Gergely & Vermeulen, sp. nov.

**Figure 101.**
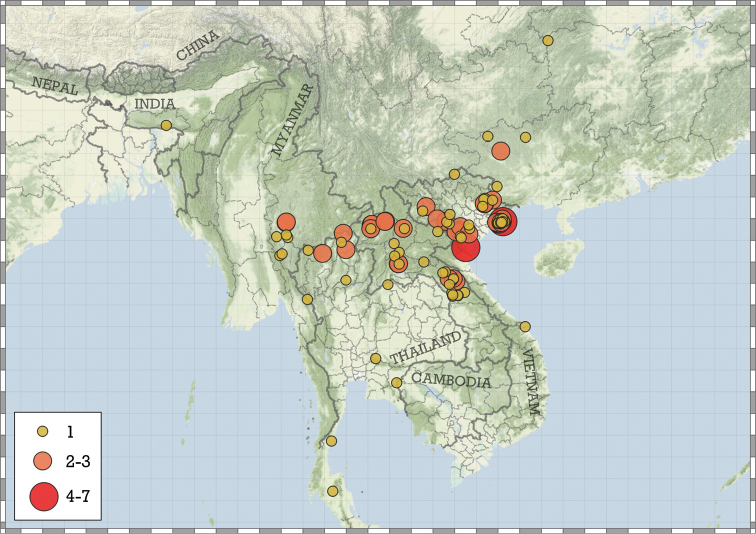
Distribution of the genus *Angustopila*. Sites with 1, 2–3, and 4–7 species are indicated differently to show the centre of diversity.

**Figure 102.**
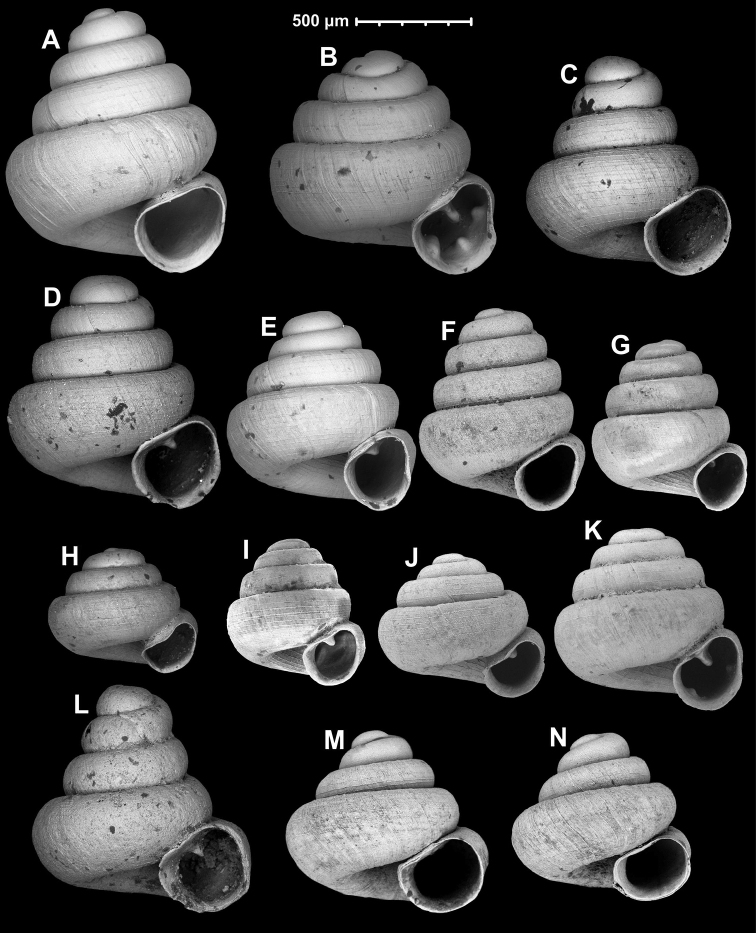
All *Angustopila* species of the Halong Bay area **A***A.tonkinospiroides* Páll-Gergely & Vermeulen, sp. nov. **B***A.quadridens* Páll-Gergely & Vermeulen, sp. nov. **C***A.halongensis* Páll-Gergely & Vermeulen, sp. nov. **D***A.babel* Páll-Gergely & Vermeulen, sp. nov. **E***A.fratermajor* Páll-Gergely & Vermeulen, sp. nov. **F***A.apiostoma* Páll-Gergely & Vermeulen, sp. nov. **G***A.fraterminor* Páll-Gergely & Vermeulen, sp. nov. **H***A.psammion* Páll-Gergely, Vermeulen & Anker, 2022 **I***A.cicatricosa* Páll-Gergely & Vermeulen, sp. nov. **J***A.maasseni* Páll-Gergely & Vermeulen, sp. nov. **K***A.hyron* Páll-Gergely & Vermeulen, sp. nov. **L***A.fabella* Páll-Gergely & Hunyadi, 2015 **M***A.megastoma* Páll-Gergely & Vermeulen, sp. nov. **N***A.thersites* Páll-Gergely & Vermeulen, sp. nov.

The shells of eight *Angustopila* species have toothless apertures; the shells of the remaining species may have one to four apertural teeth. The shells of all species with a GSD smaller than 0.8 mm have either one or two teeth in their apertures except for *A.coprologos*, which has three to four teeth. The species with larger shells have zero to four apertural teeth. Therefore, there is no obvious correlation between the number of apertural teeth and shell shape or size. We also suspect that the eggs are soft and malleable, such that they can pass the aperture even if it has barriers.

## Supplementary Material

XML Treatment for Angustopila

XML Treatment for Angustopila apiostoma

XML Treatment for Angustopila elevata

XML Treatment for Angustopila halongensis

XML Treatment for Angustopila milium

XML Treatment for Angustopila thersites

XML Treatment for Angustopila tonkinospiroides

XML Treatment for Angustopila werneri

XML Treatment for Angustopila babel

XML Treatment for Angustopila cavicola

XML Treatment for Angustopila cicatricosa

XML Treatment for Angustopila concava

XML Treatment for Angustopila erawanica

XML Treatment for Angustopila fabella

XML Treatment for Angustopila fratermajor

XML Treatment for Angustopila fraterminor

XML Treatment for Angustopila maasseni

XML Treatment for Angustopila margaritarion

XML Treatment for Angustopila megastoma

XML Treatment for Angustopila oostoma

XML Treatment for Angustopila prolixa

XML Treatment for Angustopila psammion

XML Treatment for Angustopila pusilla

XML Treatment for Angustopila szekeresi

XML Treatment for Angustopila vandevenderi

XML Treatment for Angustopila vomer

XML Treatment for Angustopila akrodon

XML Treatment for Angustopila antidomedon

XML Treatment for Angustopila bathyodon

XML Treatment for Angustopila bidentata

XML Treatment for Angustopila dominikae

XML Treatment for Angustopila gracilis

XML Treatment for Angustopila huoyani

XML Treatment for Angustopila majuscula

XML Treatment for Angustopila occidentalis

XML Treatment for Angustopila pallgergelyi

XML Treatment for Angustopila papaver

XML Treatment for Angustopila parallela

XML Treatment for Angustopila pustulata

XML Treatment for Angustopila reticulata

XML Treatment for Angustopila somsaki

XML Treatment for Angustopila steffeki

XML Treatment for Angustopila tamlod

XML Treatment for Angustopila tweediei

XML Treatment for Angustopila uvula

XML Treatment for Angustopila vitrina

XML Treatment for Angustopila

XML Treatment for Angustopila apiaria

XML Treatment for Angustopila apokritodon

XML Treatment for Angustopila coprologos

XML Treatment for Angustopila coprologos coprologos

XML Treatment for Angustopila coprologos uninodus

XML Treatment for Angustopila hyron

XML Treatment for Angustopila quadridens

XML Treatment for Angustopila rara

XML Treatment for Angustopila tetradon

XML Treatment for Angustopila tridentata

## Supplementary materials

Supplementary material 1 Dataset to estimate the number of unobserved species Data type table (excel spreadsheet) Explanation note Regions correspond with those of Fig. 6. https://binary.pensoft.net/file/808912This dataset is made available under the Open Database License (http://opendatacommons.org/licenses/odbl/1.0/). The Open Database License (ODbL) is a license agreement intended to allow users to freely share, modify, and use this Dataset while maintaining this same freedom for others, provided that the original source and author(s) are credited.Barna Páll-Gergely, András Hunyadi, Jaap J. Vermeulen, Jozef Grego, Chirasak Sutcharit, Alexander Reischütz, Pongrat Dumrongrojwattana, Zoltán Botta-Dukát, Aydin Örstan, Judit Fekete, Adrienne Jochum

Supplementary material 2 Measurement data (in mm) of all *Angustopila* species Data type table (excel spreadsheet) https://binary.pensoft.net/file/808913This dataset is made available under the Open Database License (http://opendatacommons.org/licenses/odbl/1.0/). The Open Database License (ODbL) is a license agreement intended to allow users to freely share, modify, and use this Dataset while maintaining this same freedom for others, provided that the original source and author(s) are credited.Barna Páll-Gergely, András Hunyadi, Jaap J. Vermeulen, Jozef Grego, Chirasak Sutcharit, Alexander Reischütz, Pongrat Dumrongrojwattana, Zoltán Botta-Dukát, Aydin Örstan, Judit Fekete, Adrienne Jochum

Supplementary material 3 SEM images of *Angustopila* shells Data type figures (PDF file) https://binary.pensoft.net/file/808914This dataset is made available under the Open Database License (http://opendatacommons.org/licenses/odbl/1.0/). The Open Database License (ODbL) is a license agreement intended to allow users to freely share, modify, and use this Dataset while maintaining this same freedom for others, provided that the original source and author(s) are credited.Barna Páll-Gergely, András Hunyadi, Jaap J. Vermeulen, Jozef Grego, Chirasak Sutcharit, Alexander Reischütz, Pongrat Dumrongrojwattana, Zoltán Botta-Dukát, Aydin Örstan, Judit Fekete, Adrienne Jochum
