# Supplementary material for: ﻿Five times over: 42 new Angustopila species highlight Southeast Asia’s rich biodiversity (Gastropoda, Stylommatophora, Hypselostomatidae)
Source: Zookeys. 2023 Feb 13;1147:1–177. doi: 10.3897/zookeys.1147.93824 (PMC10297824; doi:10.3897/zookeys.1147.93824)
Supplement: Supplementary material 3 — SEM images of Angustopila shells [file zookeys-1147-001_article-93824__-s003.pdf]

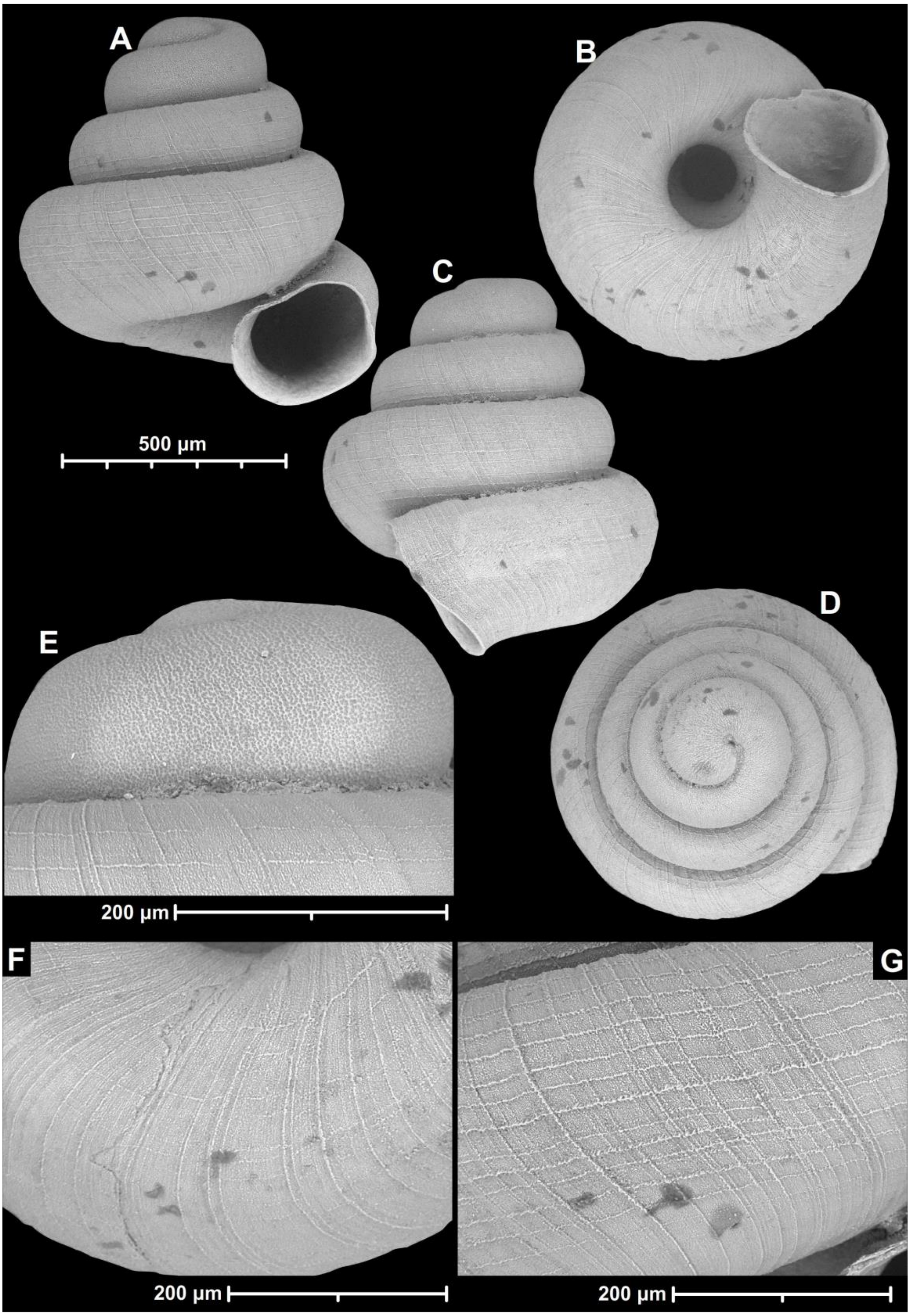

Supplementary Figure 1. *Angustopila elevata* (Thompson & Upatham, 1997) (specimen from Tha Song Yang). Apertural (**A**), ventral (**B**), lateral (**C**) and apical (**D**) sides of the shell; granular sculpture on the protoconch (**E**), ventral (**F**) and frontal (**G**) surface of the body whorl.

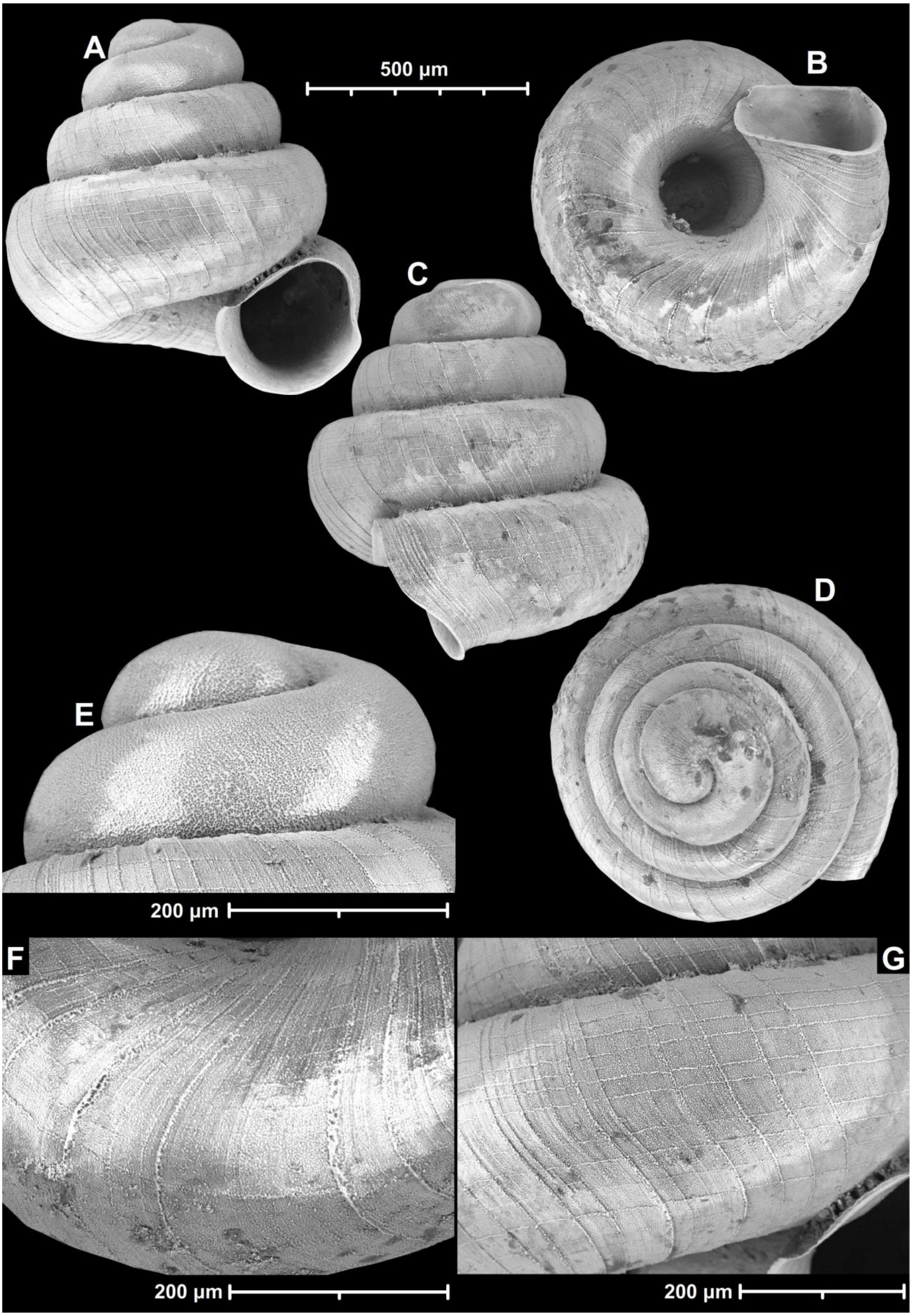

Supplementary Figure 2. *Angustopila elevata* (Thompson & Upatham, 1997), sample 2018/36. Apertural (**A**), ventral (**B**), lateral (**C**) and apical (**D**) sides of the shell; granular sculpture on the protoconch (**E**), ventral (**F**) and frontal (**G**) surface of the body whorl.

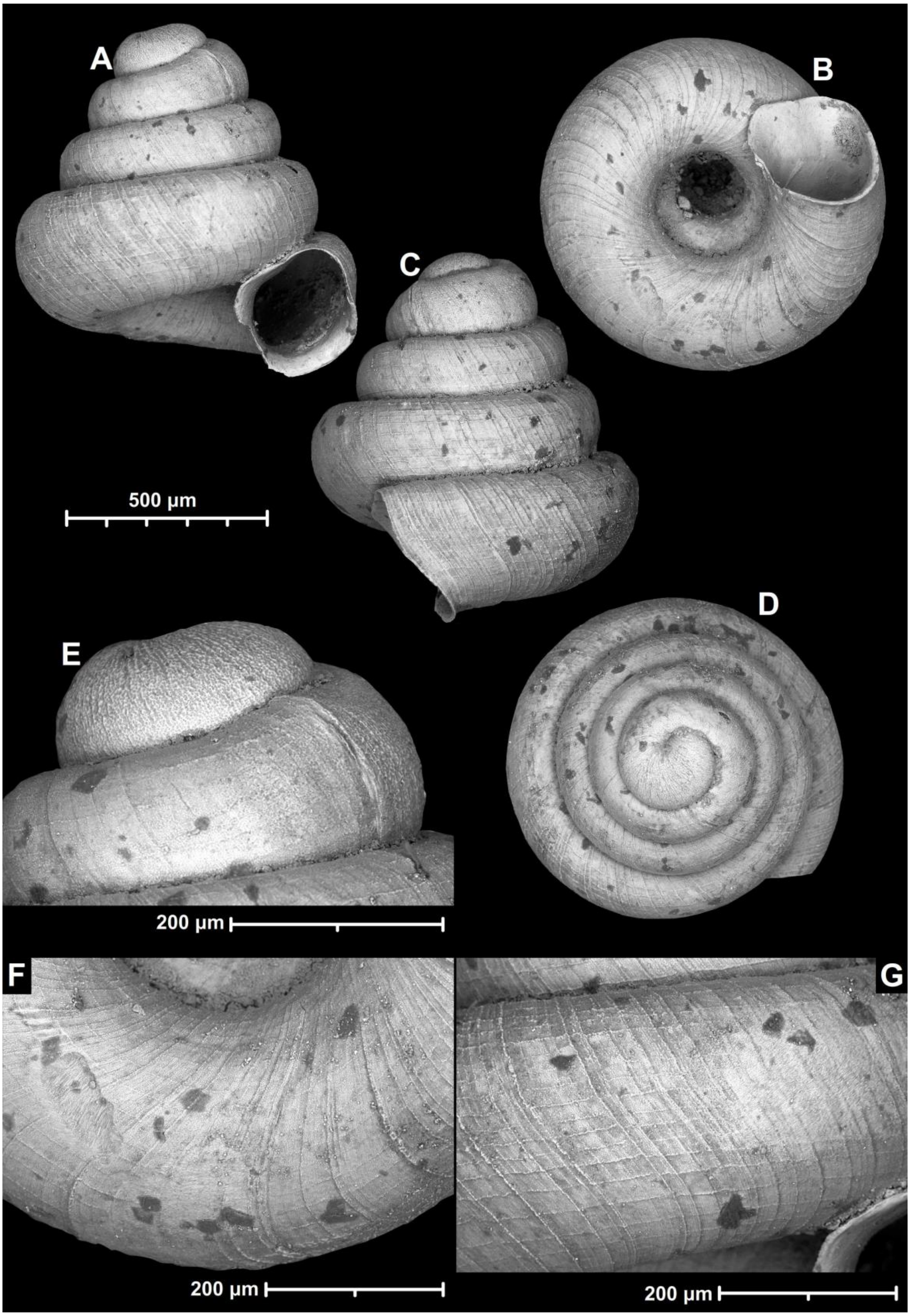

Supplementary Figure 3. *Angustopila elevata* (Thompson & Upatham, 1997), sample 2015/31. Apertural (**A**), ventral (**B**), lateral (**C**) and apical (**D**) sides of the shell; granular sculpture of the protoconch with protoconch-teleoconch boundary (**E**), ventral (**F**) and frontal (**G**) surface of the body whorl.

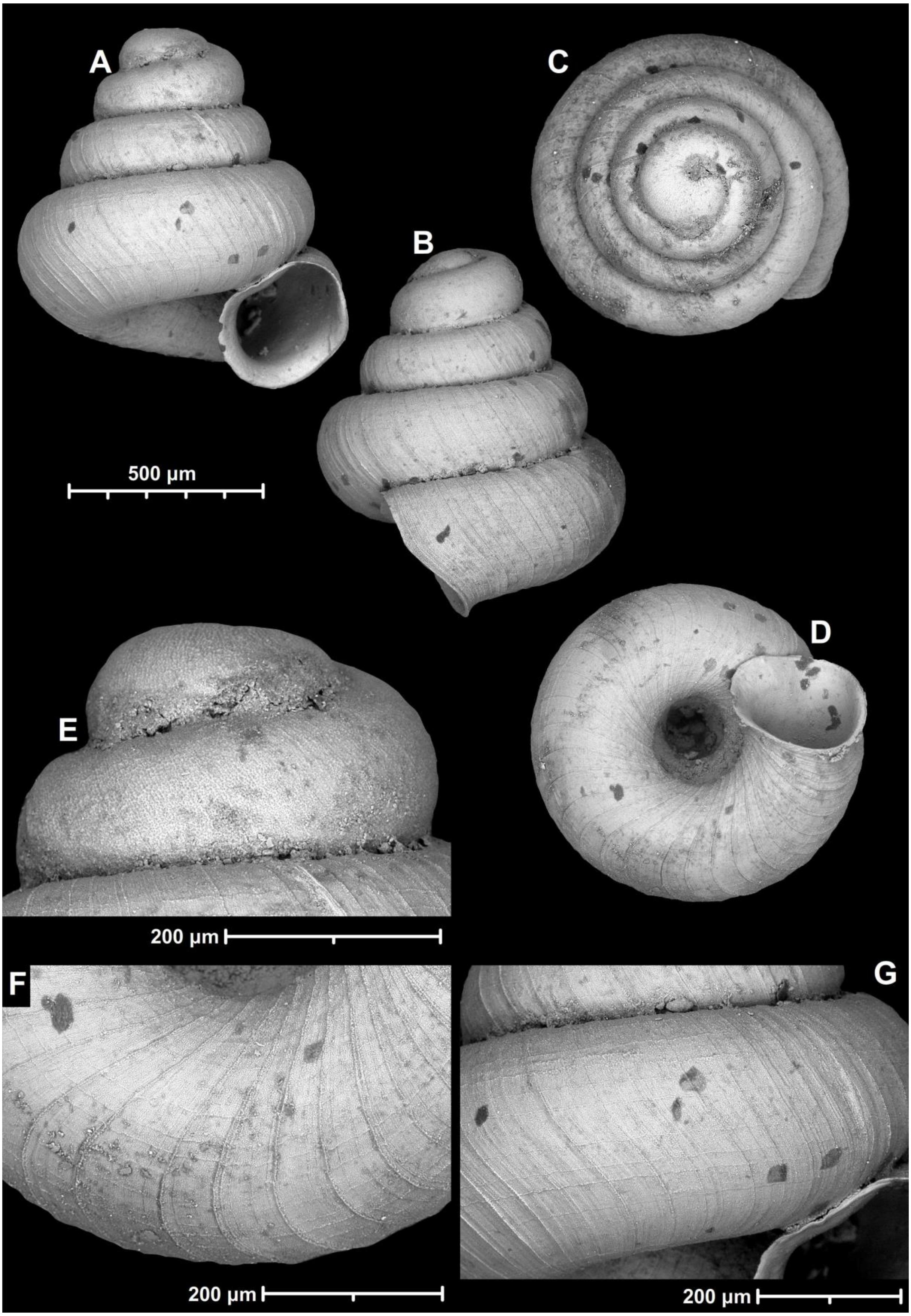

Supplementary Figure 4. *Angustopila elevata* (Thompson & Upatham, 1997), sample 2019/122. Apertural (**A**), lateral (**B**), apical (**C**) and ventral (**D**) sides of the shell; granular sculpture on the protoconch (**E**), ventral (**F**) and frontal (**G**) surface of the body whorl.

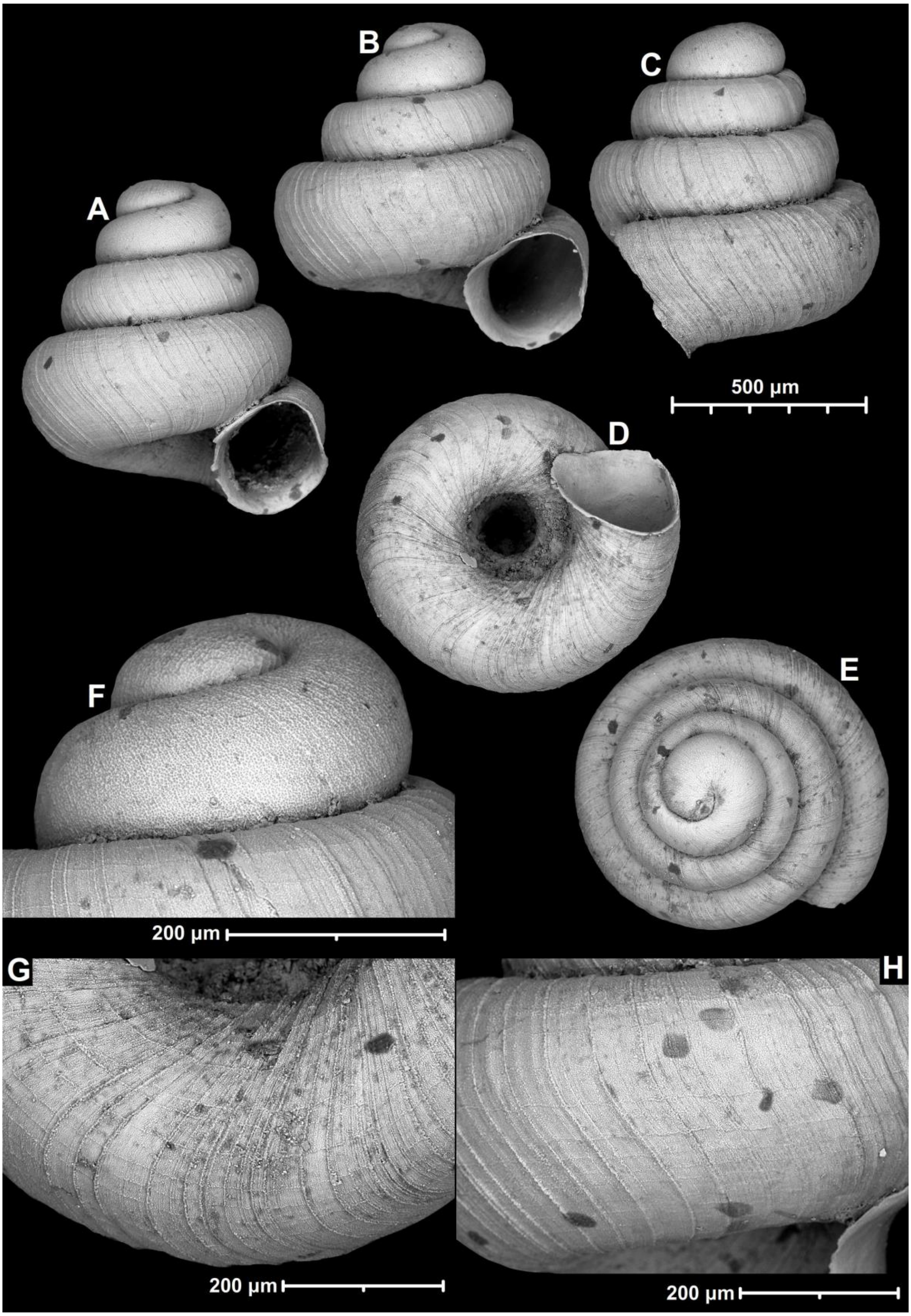

Supplementary Figure 5. *Angustopila elevata* (Thompson & Upatham, 1997), sample 2020/18. **A**: Specimen2, **B–H**: Specimen1. Apertural (**A–B**), lateral (**C**), ventral (**D**) and apical (**E**) sides of the shell; granular sculpture on the protoconch (**F**), ventral (**G**) and frontal (**H**) surface of the body whorl.

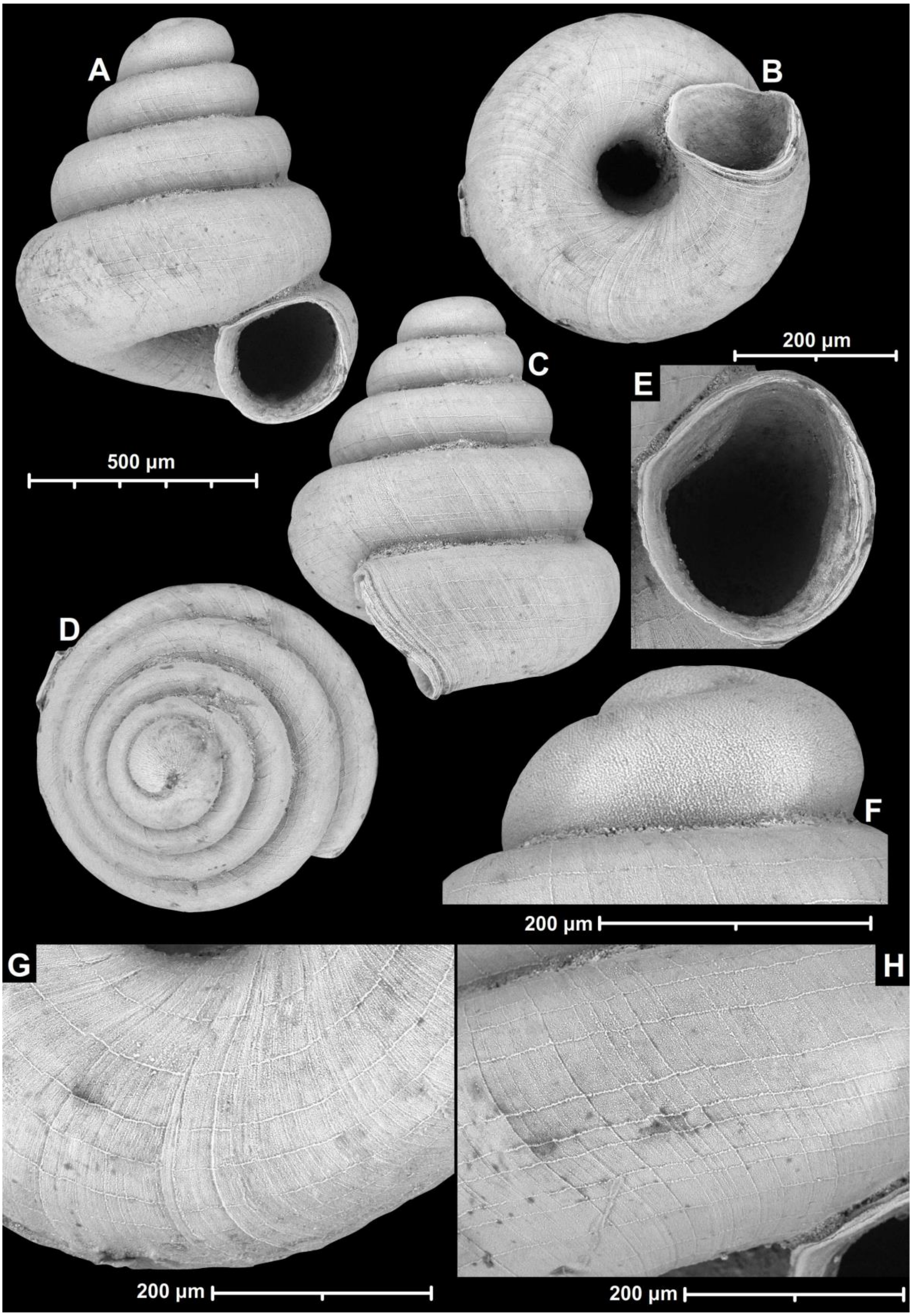

Supplementary Figure 6. *Angustopila elevata* (Thompson & Upatham, 1997) (La.2, specimen1). Apertural (**A**), ventral (**B**), lateral (**C**), and apical (**D**) sides of the shell; aperture (**E**); granular surface sculpture on the protoconch (**F**), spiral and radial structure on ventral (**G**) and frontal (**H**) surface of the body whorl.

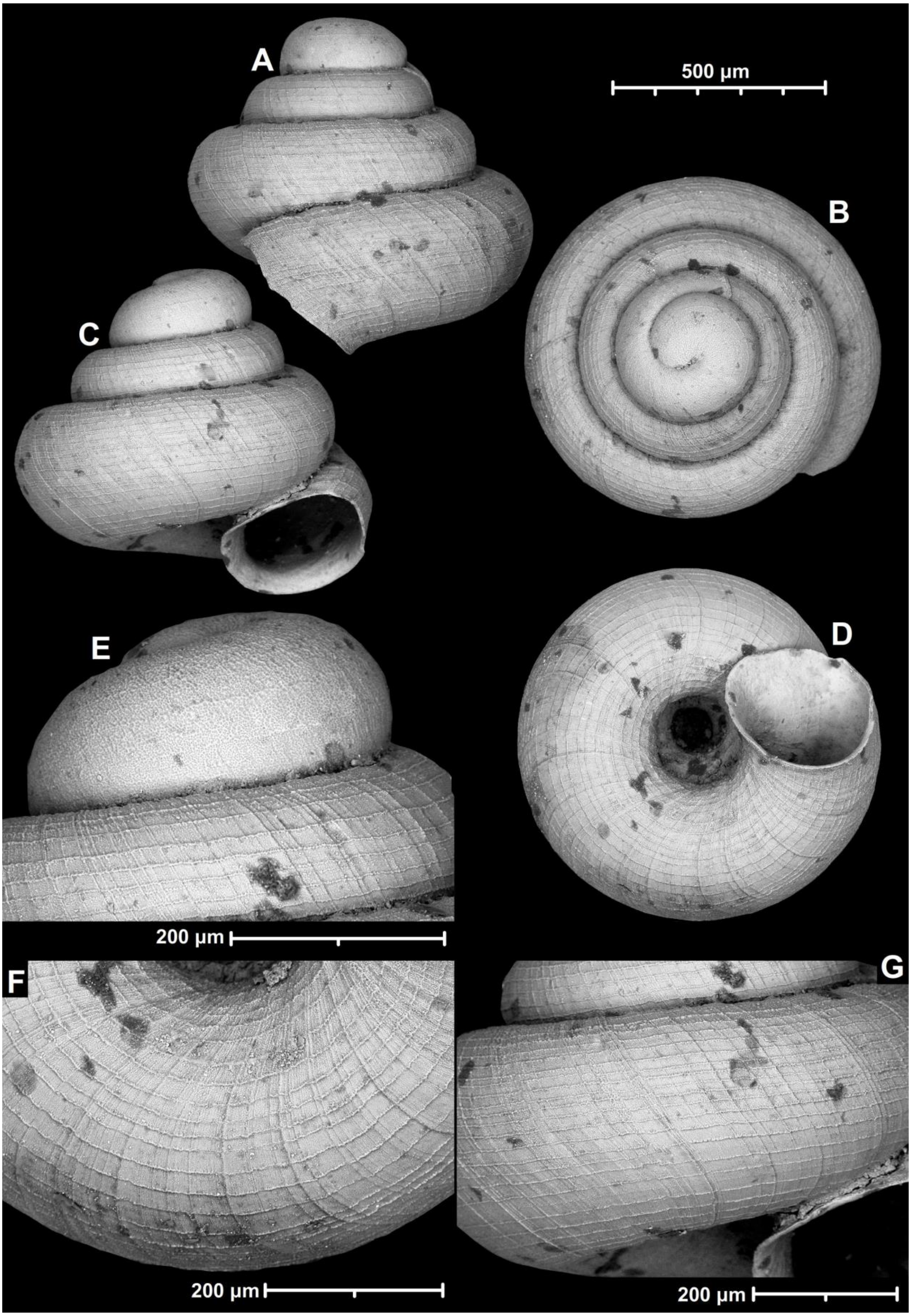

Supplementary Figure 7. *Angustopila thersites* Páll-Gergely & Vermeulen, sp. n., sample JJV 17654 (ex JJV 6237). Lateral (**A**), apical (**B**), apertural (**C**) and ventral (**D**) sides of the shell; sculpture of the protoconch (**E**), ventral (**F**) and frontal (**G**) surface of the body whorl.

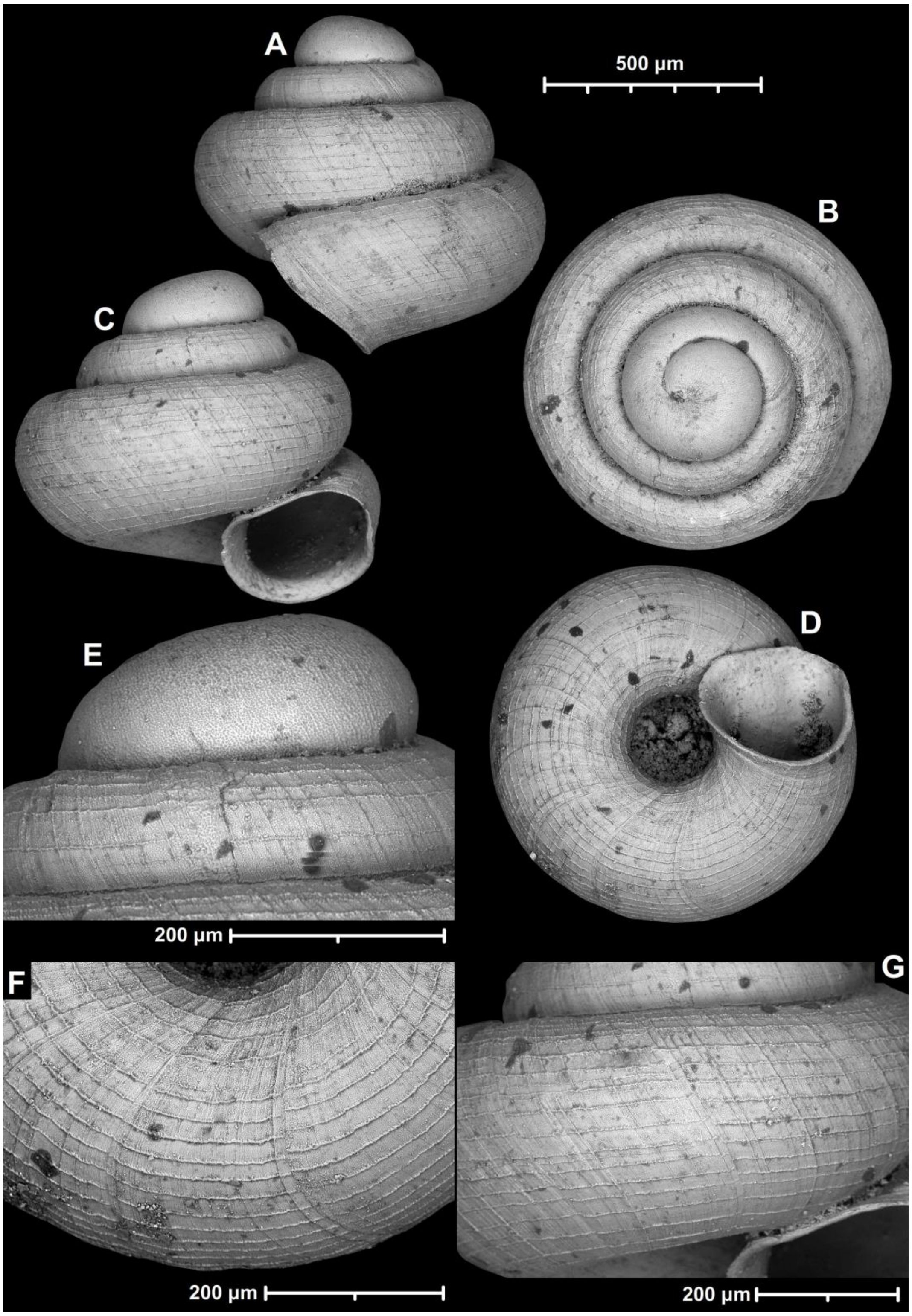

Supplementary Figure 8. *Angustopila thersites* Páll-Gergely & Vermeulen, sp. n., sample JJV 16621. Lateral (**A**), apical (**B**), apertural (**C**) and ventral (**D**) sides of the shell; sculpture of the protoconch (**E**), ventral (**F**) and frontal (**G**) surface of the body whorl.

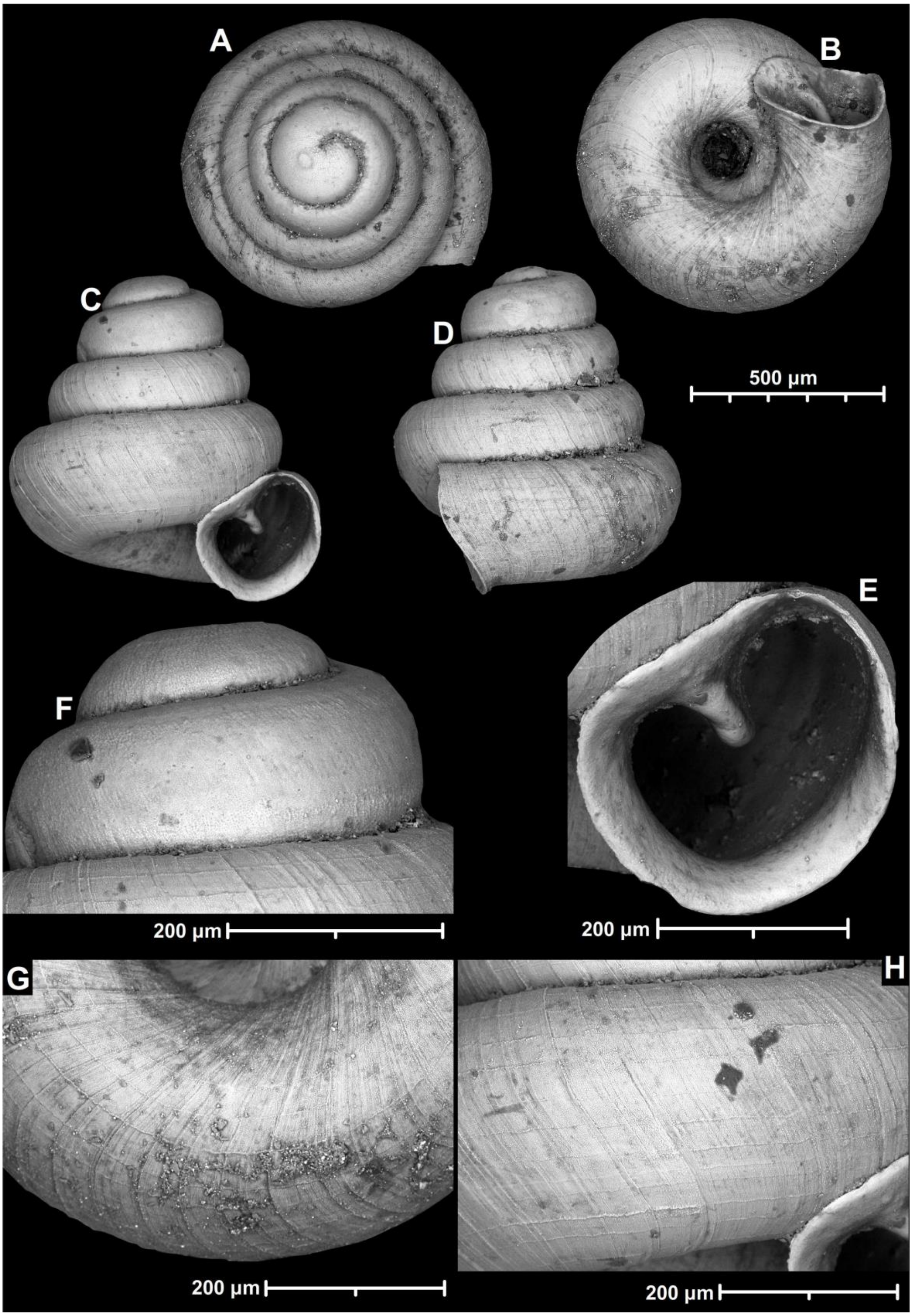

Supplementary Figure 9. *Angustopila fabella* Páll-Gergely & Hunyadi, 2015, sample 2019.118, species2 Apical (**A**), ventral (**B**), apertural (**C**) and lateral (**D**) sides of the shell; aperture (**E**), microstructure of the protoconch (**F**), microstructure on ventral (**G**) and frontal (**H**) surface of the body whorl.

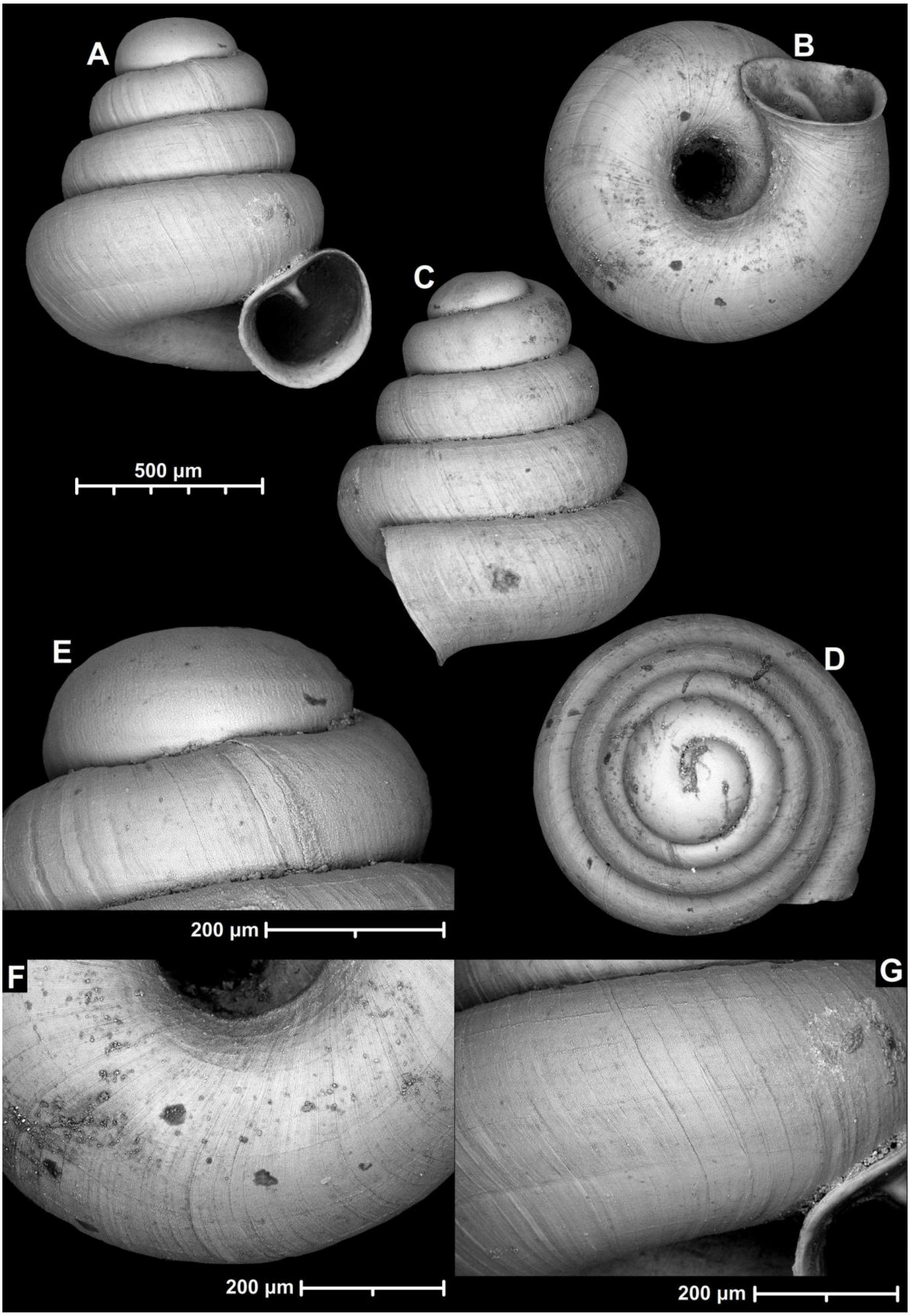

Supplementary Figure 10. *Angustopila fabella* Páll-Gergely & Hunyadi, 2015, sample 2019.118, "SpeciesX". Apertural (**A**), ventral (**B**), lateral (**C**) and apical (**D**) sides of the shell; microstructure of the protoconch showing protoconch-teleoconch boundary (**E**), ventral (**F**) and frontal (**G**) surface of the body whorl.

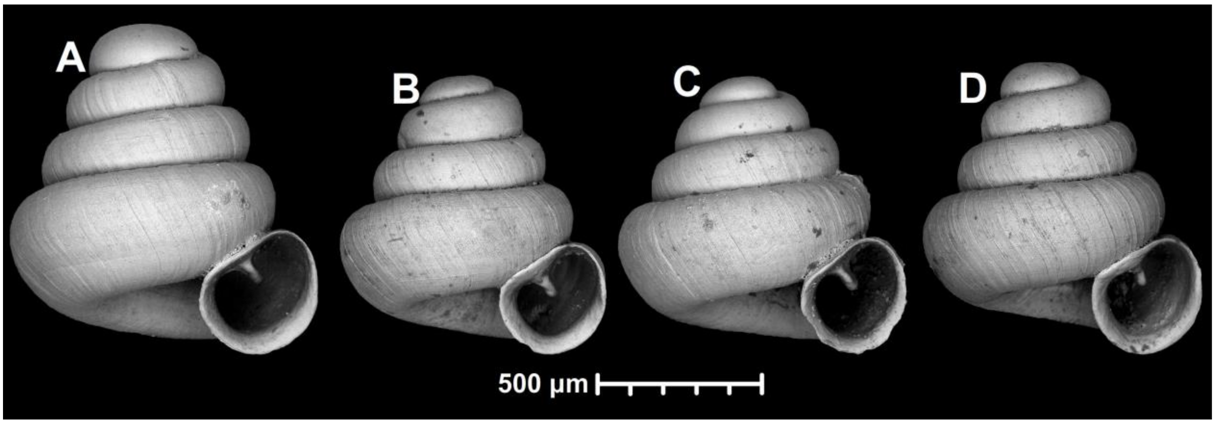

Supplementary Figure 11. *Angustopila fabella* Páll-Gergely & Hunyadi, 2015, sample 2019.118, **A**: "SpeciesX **B–D**: "Species2".

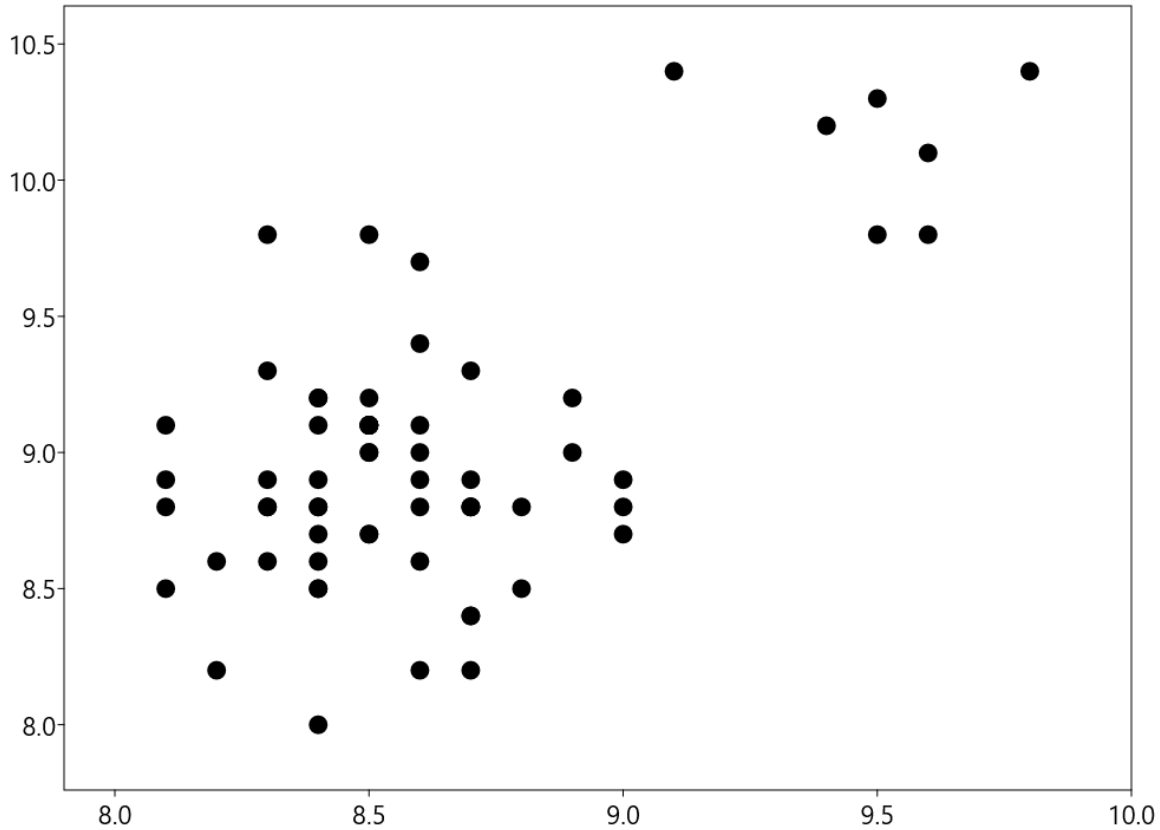

Supplementary Figure 12. Shell height (Y axis, x0.1 mm) vs. shell width (X axis, x0.1 mm) of sample 2019.118 of *Angustopila fabella* Páll-Gergely & Hunyadi, 2015. This graph shows that the shells from this locality form non-overlapping clusters. The lower left cluster represents "*Angustopila fabella* 2019.118, Species2" (Supplementary Figure 8, 10B–D), while the upper right cluster represents "*Angustopila fabella* 2019.118, Species x" (Supplementary Figure 9, 10A).

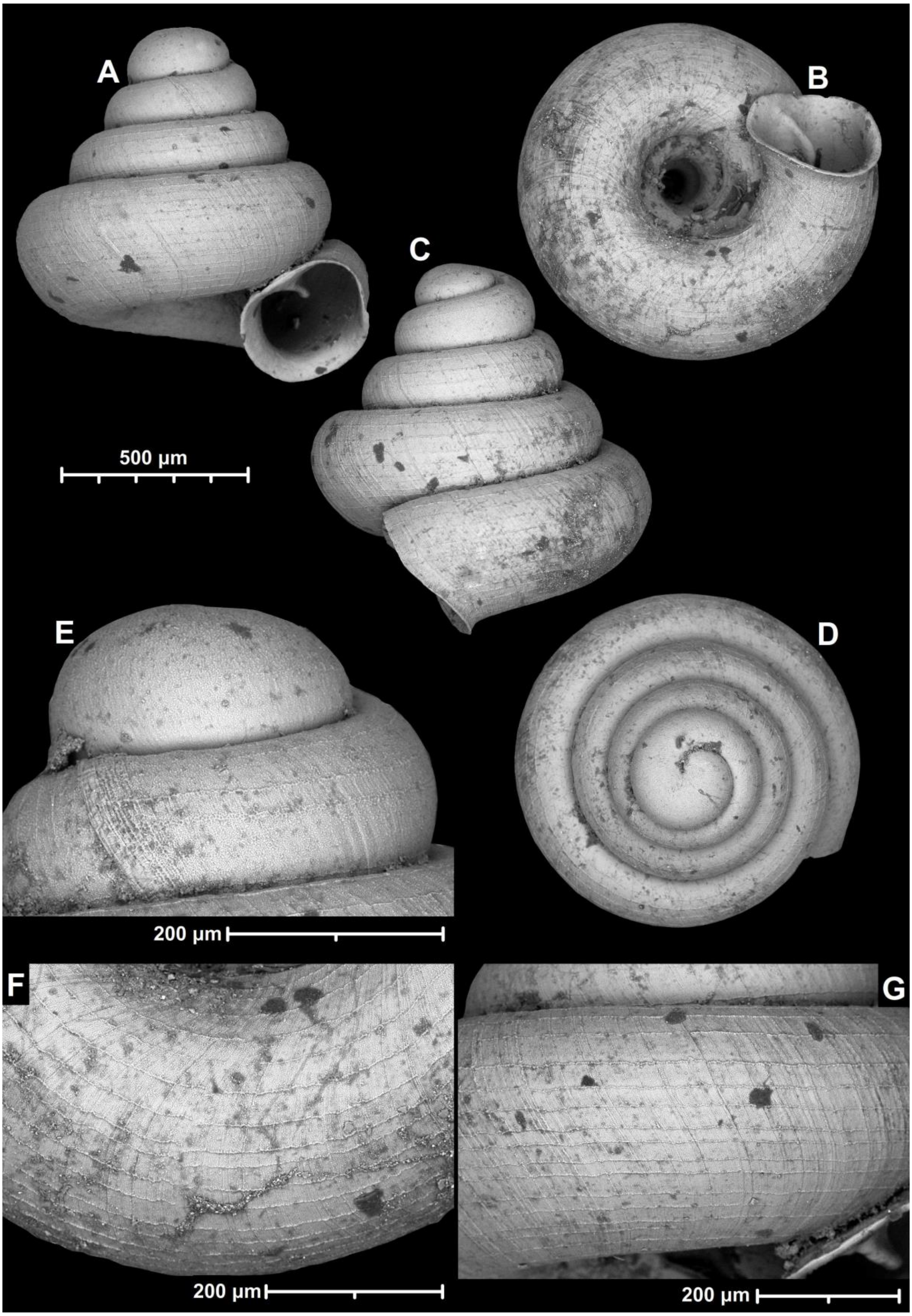

Supplementary Figure 13. *Angustopila fabella* 2019.128.type1. Apertural (**A**), ventral (**B**), lateral (**C**) and apical (**D**) sides of the shell; microstructure of the protoconch showing protoconch-teleoconch boundary (**E**), microstructure of ventral (**F**) and frontal (**G**) surface of the body whorl.

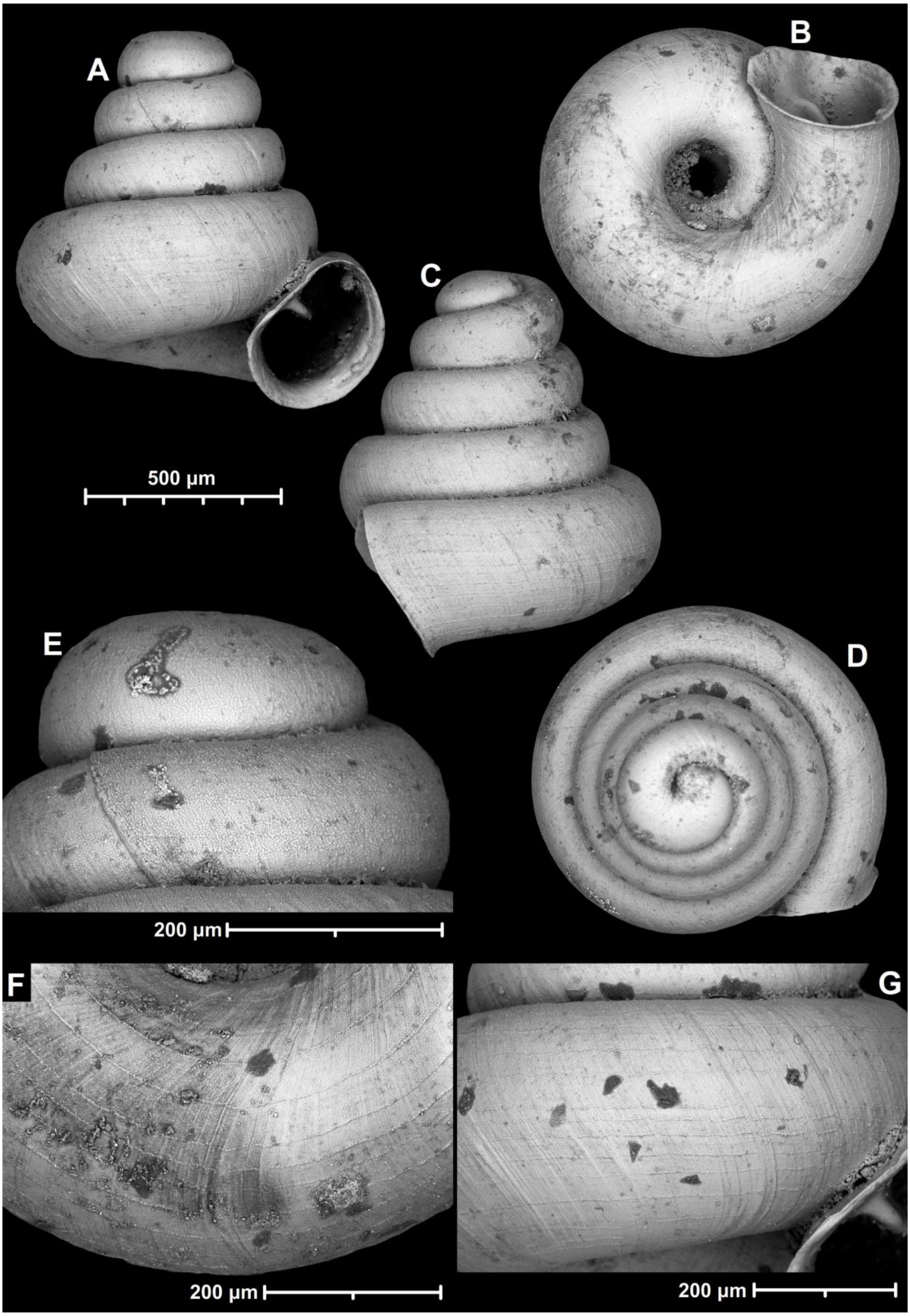

Supplementary Figure 14. *Angustopila fabella* 2019.128.type2. Apertural (**A**), ventral (**B**), lateral (**C**) and apical (**D**) sides of the shell; microstructure of the protoconch showing protoconch-teleoconch boundary (**E**), microstructure of ventral (**F**) and frontal (**G**) surface of the body whorl.

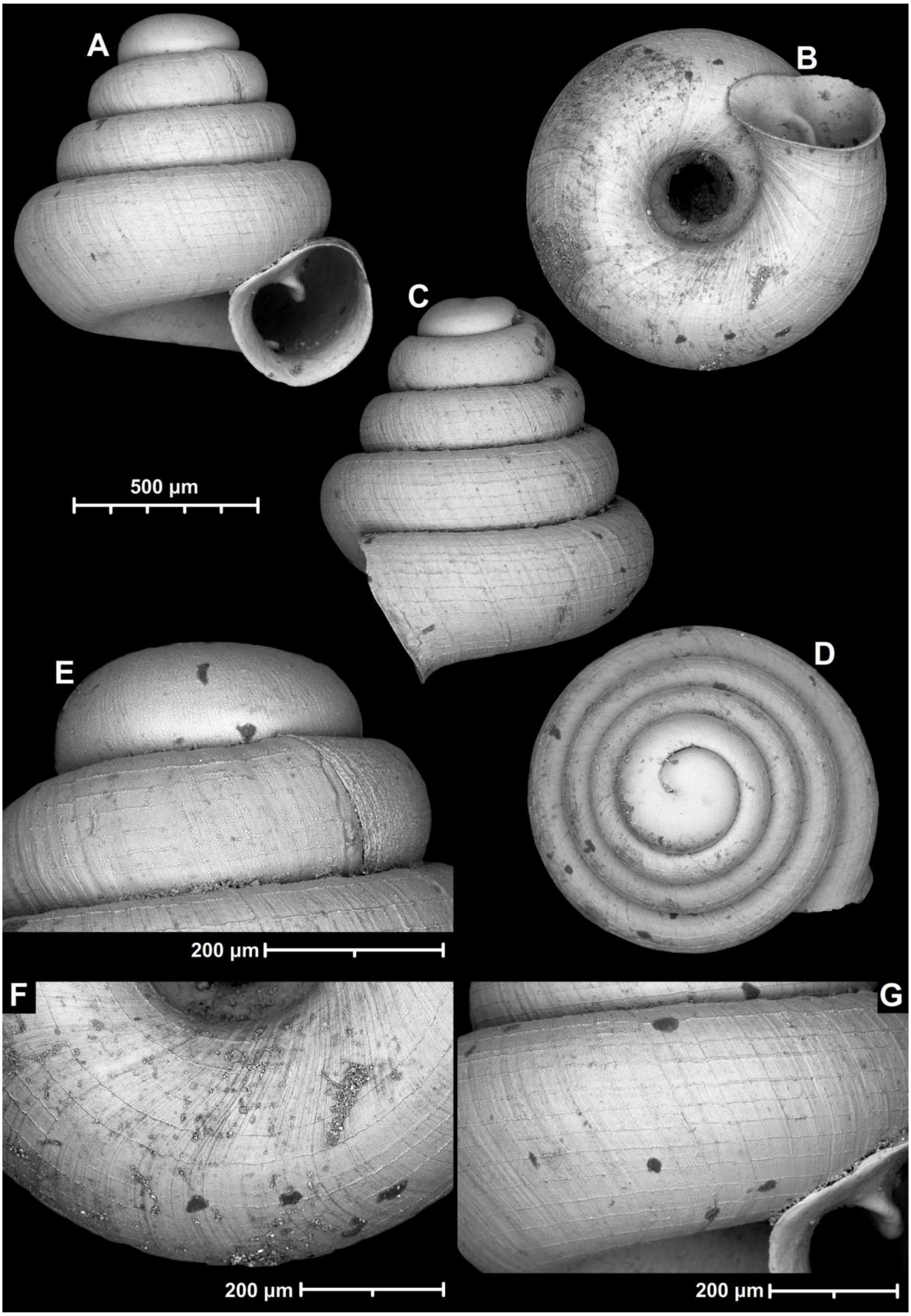

Supplementary Figure 15. *Angustopila fabella* 2019.128.type3. Apertural (**A**), ventral (**B**), lateral (**C**) and apical (**D**) sides of the shell; microstructure of the protoconch showing protoconch-teleoconch boundary (**E**), microstructure of ventral (**F**) and frontal (**G**) surface of the body whorl.

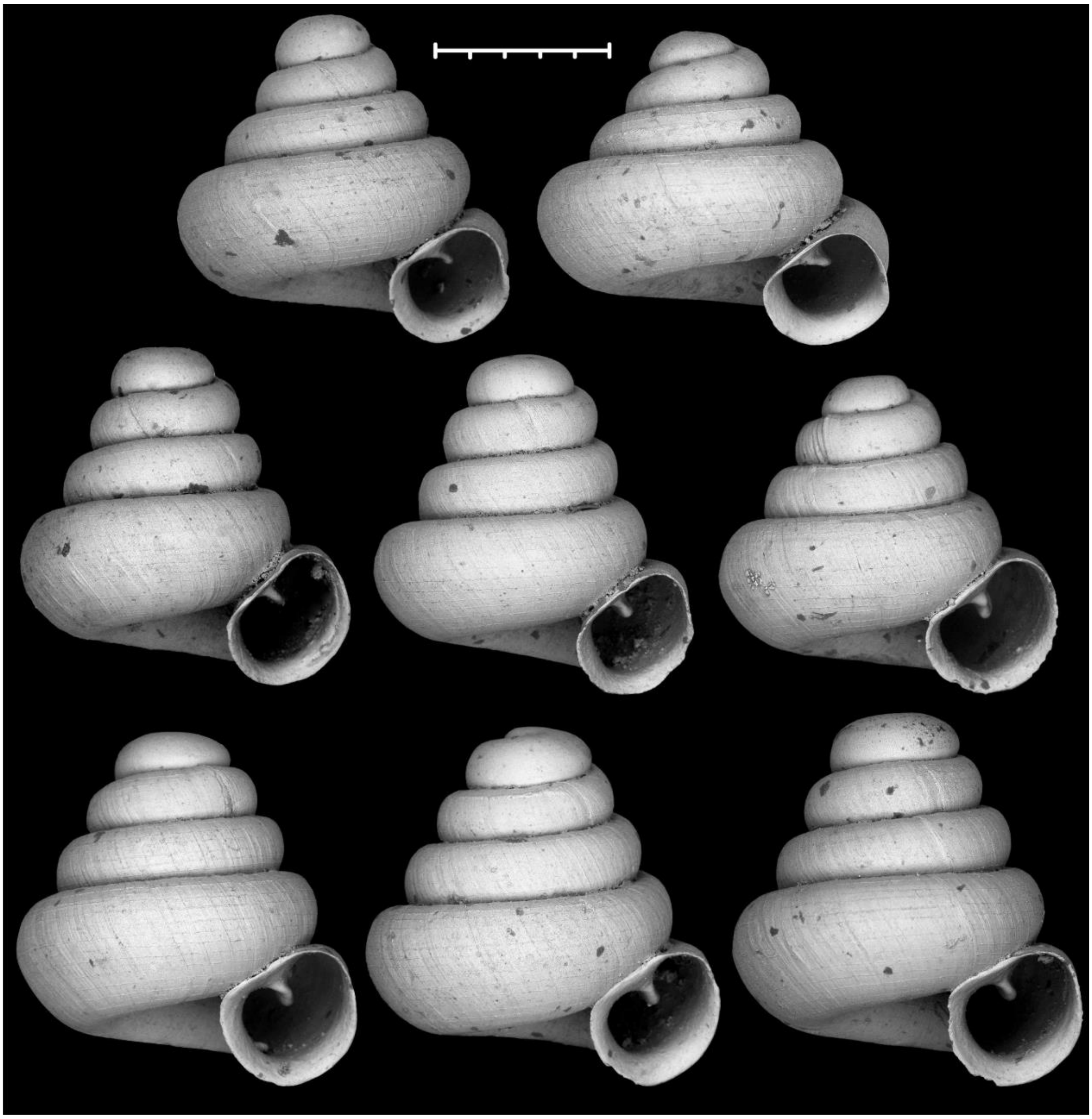

Supplementary Figure 16. *Angustopila fabella* 2019.128 three types, synoptic. First row: type1 (Supplementary Figure 12), second row: type2 (Supplementary Figure 13), third row: type3 (Supplementary Figure 14).

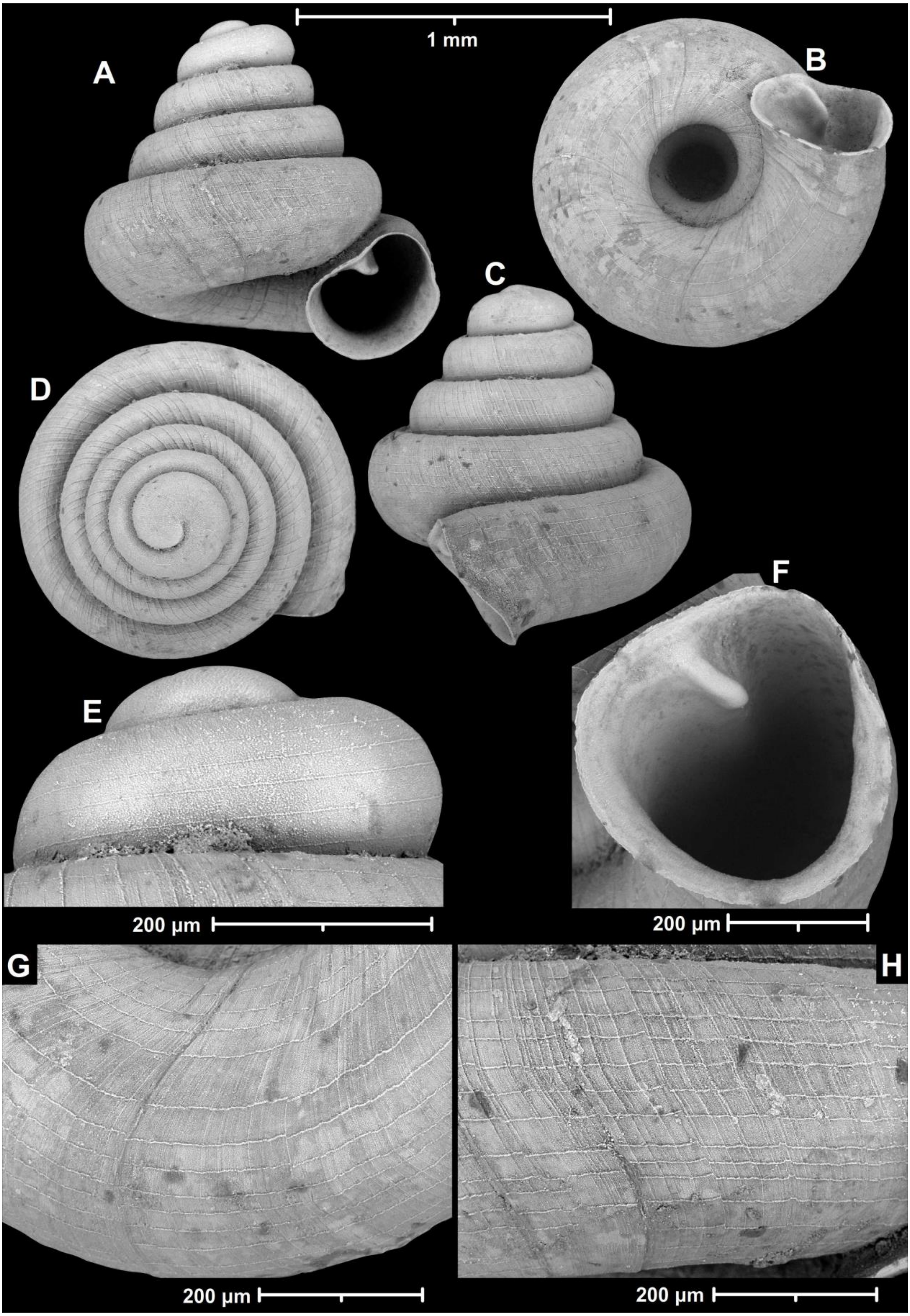

Supplementary Figure 17. *Angustopila fabella* 15L06_b (MNHN-IM-2014-6414). Apertural (**A**), ventral (**B**), lateral (**C**) and apical (**D**) sides of the shell; microstructure of the protoconch (**E**), aperture (**F**), ventral (**G**) and frontal (**H**) surface of the body whorl.

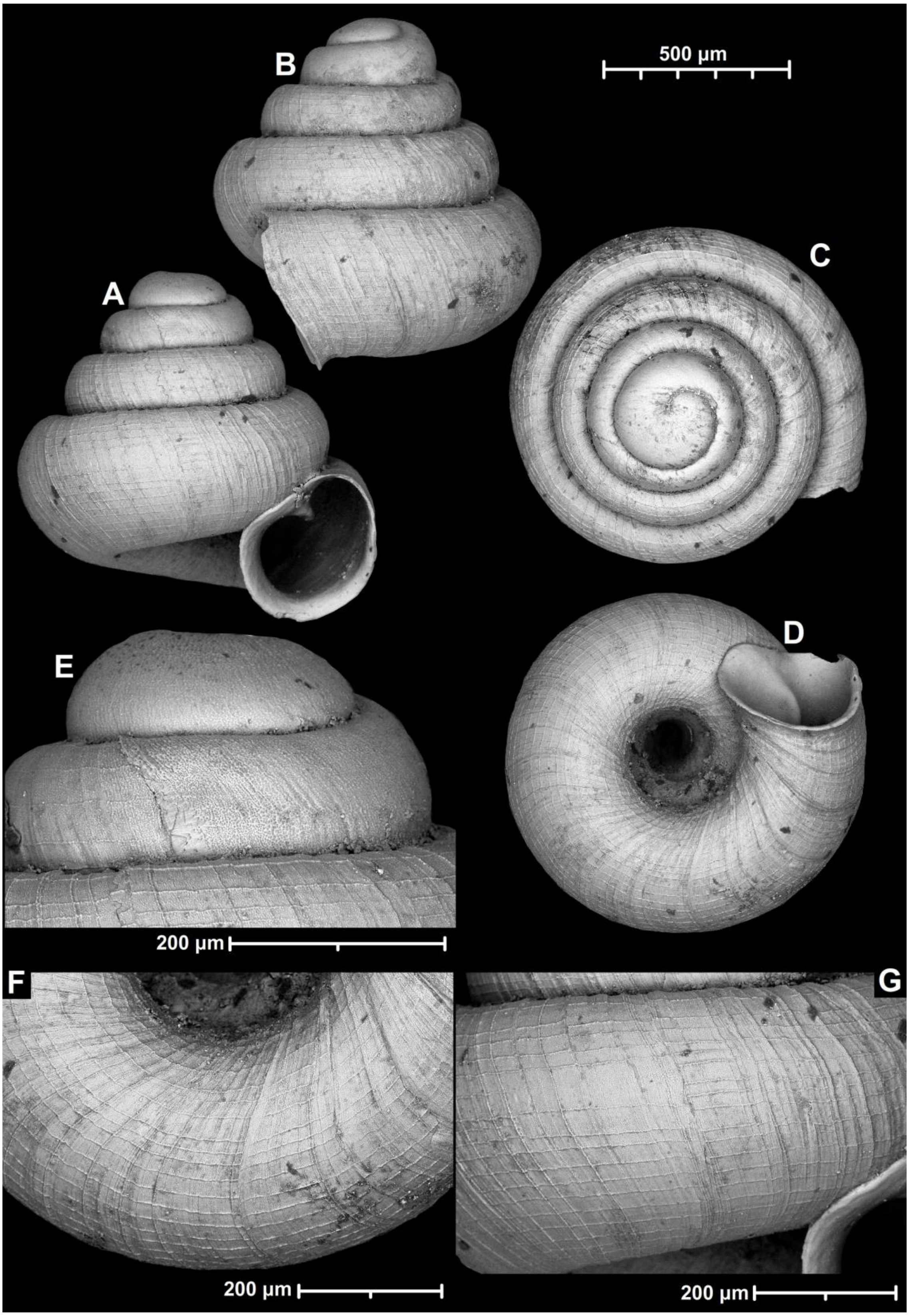

Supplementary Figure 18. *Angustopila fabella* Páll-Gergely & Hunyadi, 2015 (conical-globular type) 2020.20_species2. Apertural (**A**), lateral (**B**), apical (**C**) and ventral (**D**) sides of the shell; microstructure of the protoconch showing protoconch-teleoconch boundary (**E**), ventral (**F**) and frontal (**G**) surface of the body whorl.

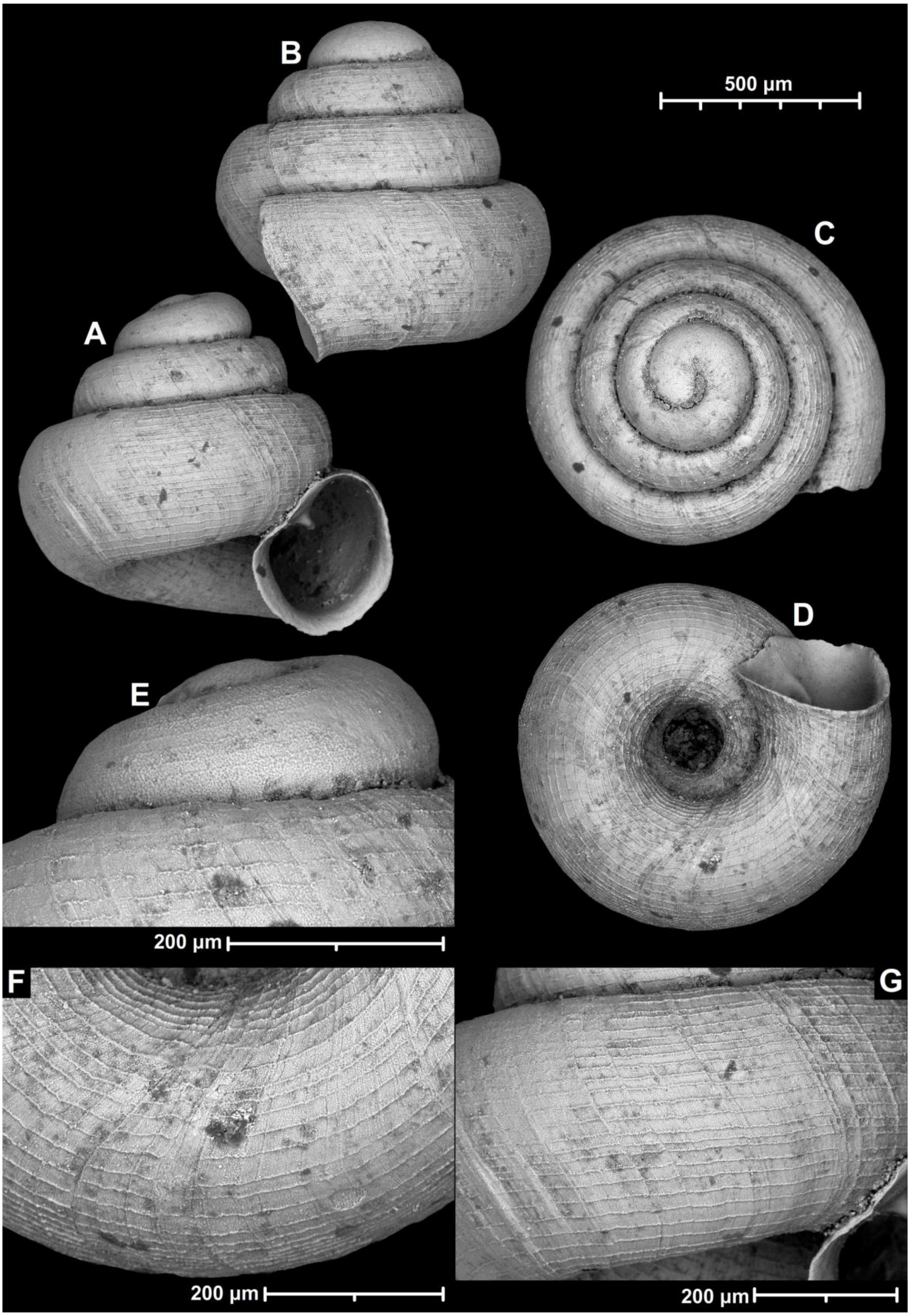

Supplementary Figure 19. *Angustopila fabella* Páll-Gergely & Hunyadi, 2015 (conical-globular type) 2020.24_specimen1. Apertural (**A**), lateral (**B**), apical (**C**) and ventral (**D**) sides of the shell; microstructure of the protoconch (**E**), ventral (**F**) and frontal (**G**) surface of the body whorl.

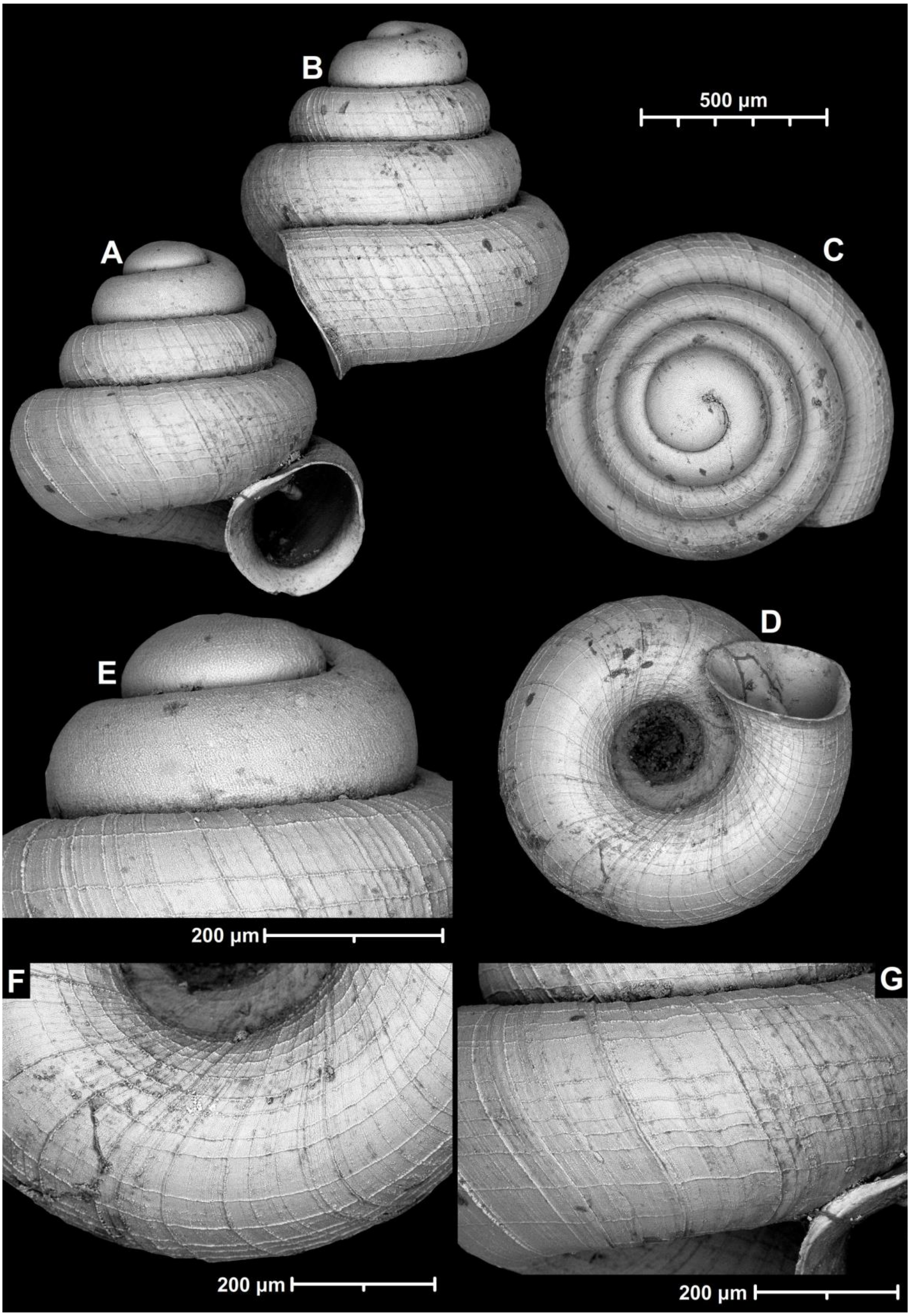

Supplementary Figure 20. *Angustopila fabella* Páll-Gergely & Hunyadi, 2015, sample 2020/9 (conical-globular type with wide umbilicus: "wu_specimen1"). Apertural (**A**), lateral (**B**), apical (**C**) and ventral (**D**) sides of the shell; microstructure of the protoconch (**E**), ventral (**F**) and frontal (**G**) surface of the body whorl.

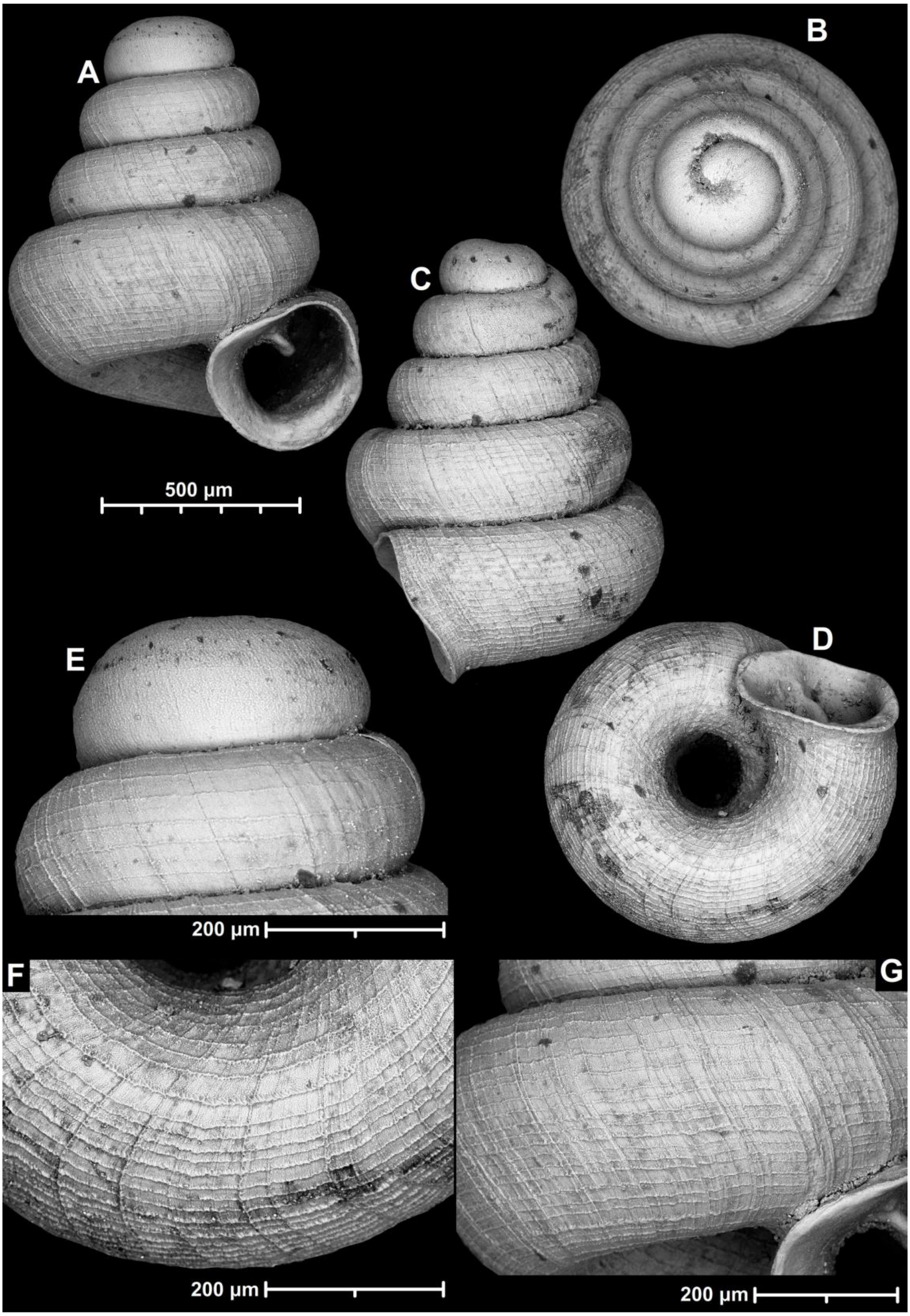

Supplementary Figure 21. *Angustopila fabella* Páll-Gergely & Hunyadi, 2015, sample 2020/9 (conical type with narrow umbilicus: "nu_specimen1"). Apertural (**A**), apical (**B**), lateral (**C**), and ventral (**D**) sides of the shell; microstructure of the protoconch (**E**), ventral (**F**) and frontal (**G**) surface of the body whorl.

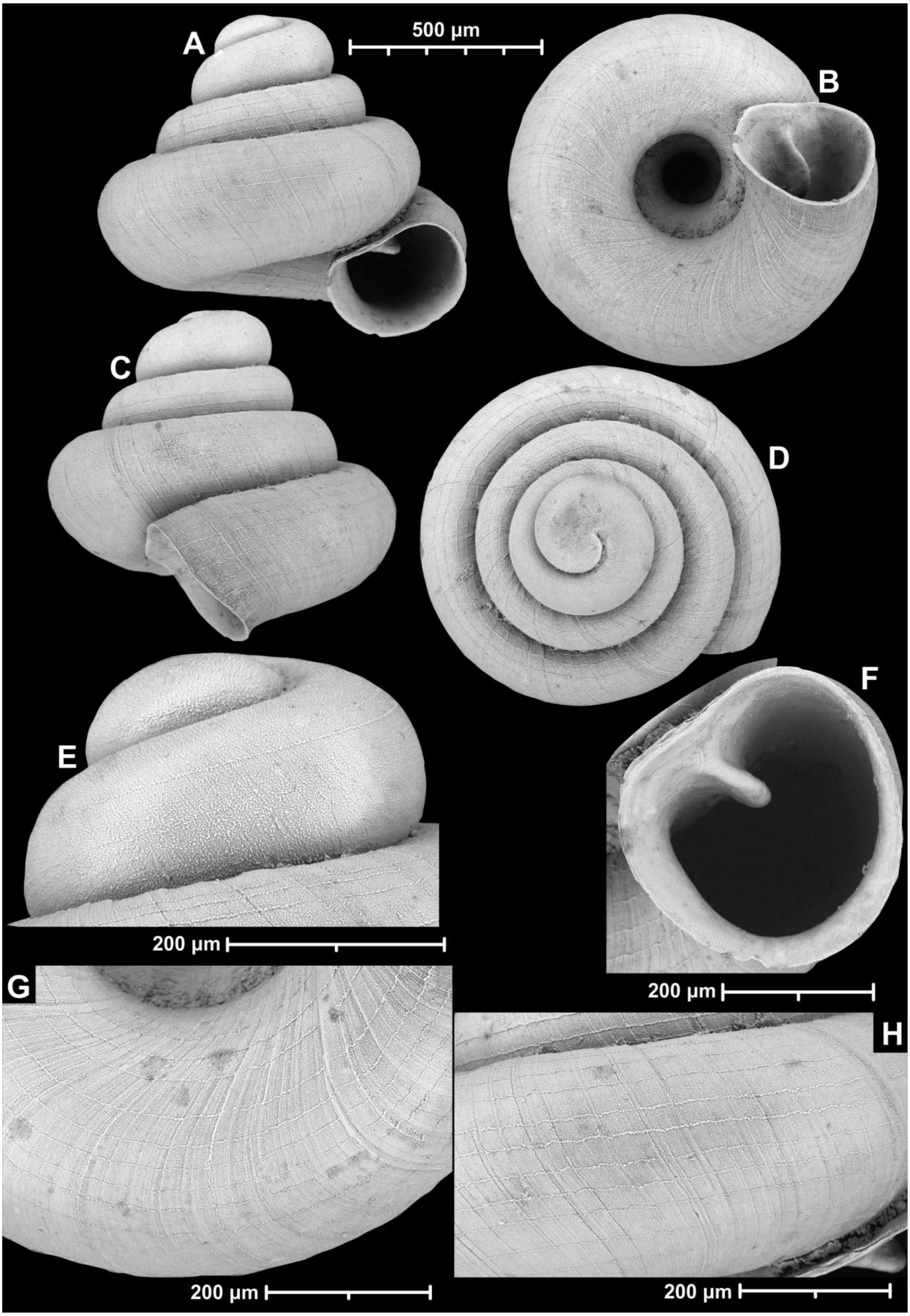

Supplementary Figure 22. *Angustopila fabella* Páll-Gergely & Hunyadi, 2015, sample 32L06. Apertural (**A**), ventral (**B**), lateral (**C**) and apical (**D**) sides of the shell; aperture (**F**), microstructure of the protoconch (**E**), ventral (**G**) and frontal (**H**) surface of the body whorl.

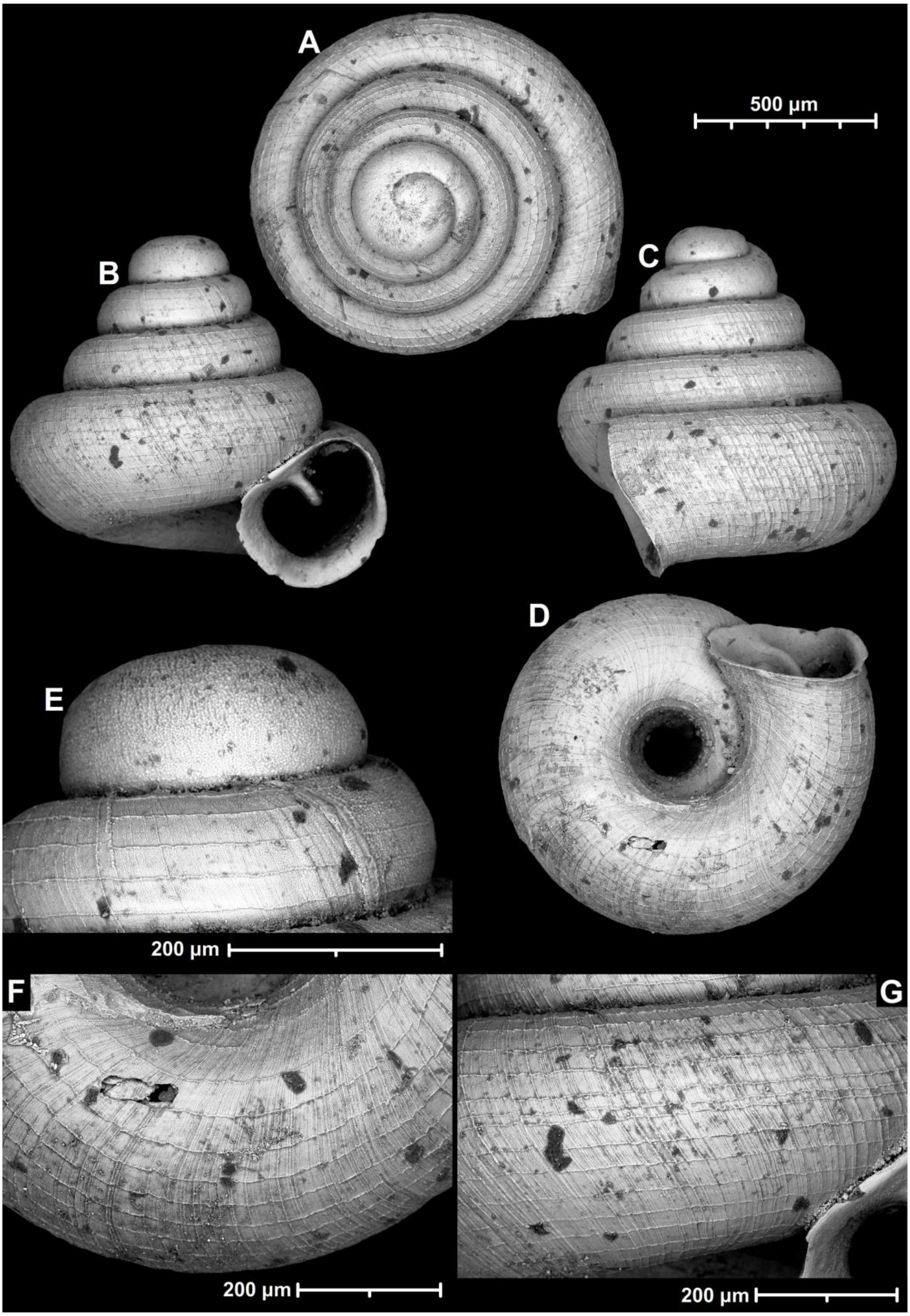

Supplementary Figure 23. *Angustopila fabella* Páll-Gergely & Hunyadi, 2015 2020.30.species1. Apical (**A**), apertural (**B**), lateral (**C**) and ventral (**D**) sides of the shell; microstructure of the protoconch showing the protoconch-teleoconch boundary (**E**), ventral (**F**) and frontal (**G**) surface of the body whorl.

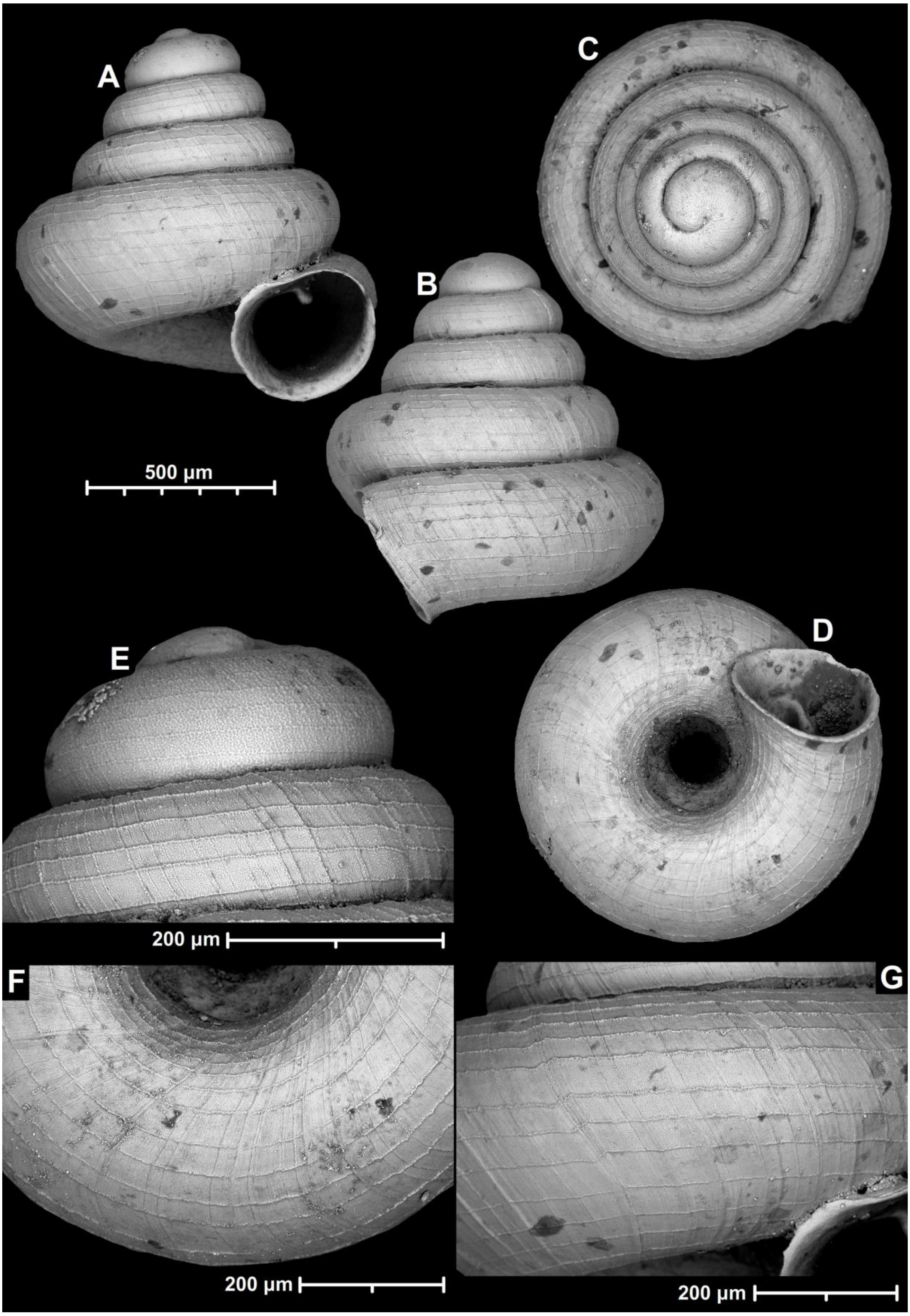

Supplementary Figure 24. *Angustopila fabella* Páll-Gergely & Hunyadi, 2015, sample 2020/41, species6. Apertural (**A**), lateral (**B**), apical (**C**) and ventral (**D**) sides of the shell; microstructure of the protoconch (**E**), ventral (**F**) and frontal (**G**) surface of the body whorl.

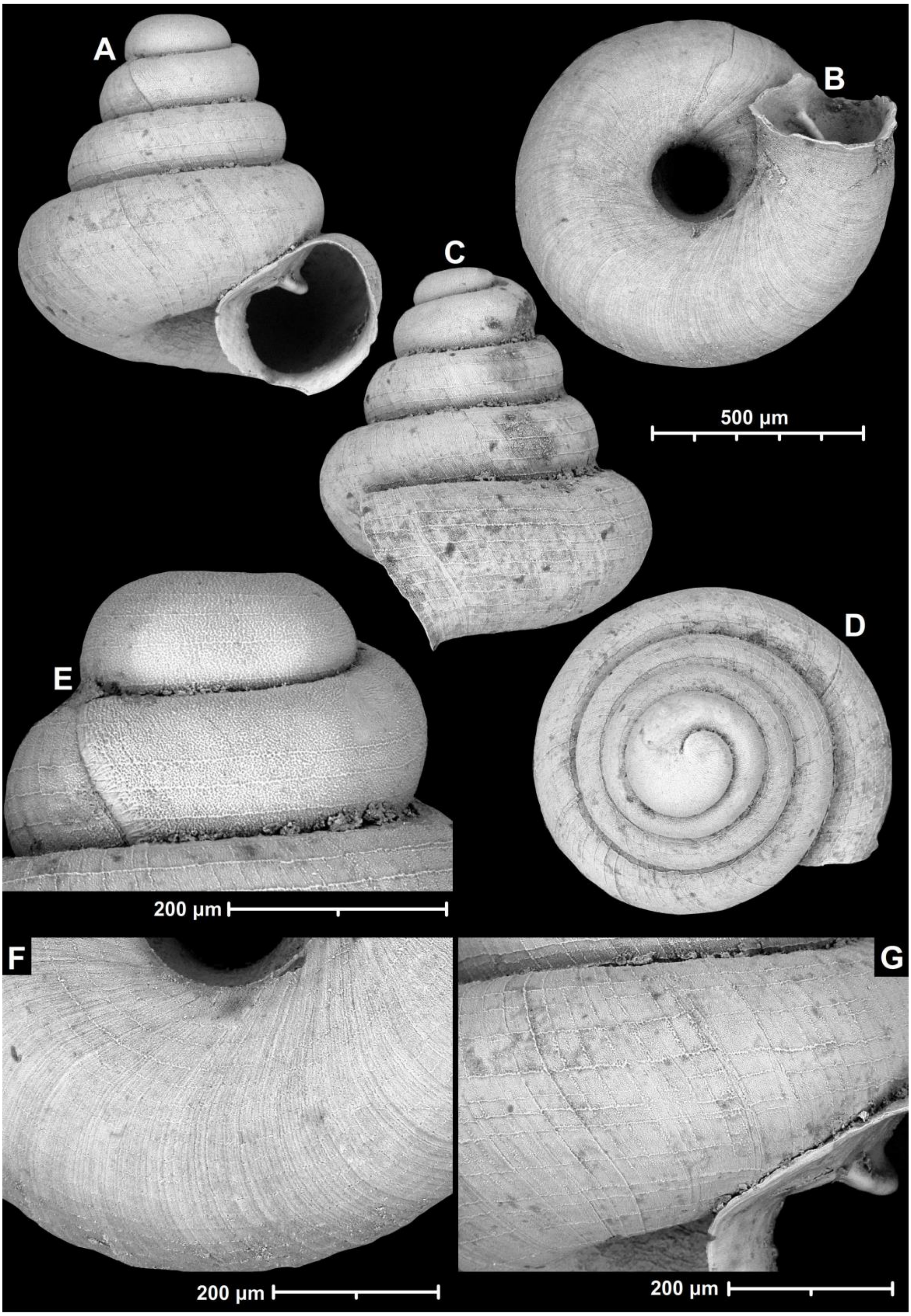

Supplementary Figure 25. *Angustopila fabella* Páll-Gergely & Hunyadi, 2015, sample JG12, specimen1_b. Apertural (**A**), ventral (**B**), lateral (**C**) and apical (**D**) sides of the shell; microstructure of the protoconch showing protoconch-teleoconch boundary (**E**), ventral (**F**) and frontal (**G**) surface of the body whorl.

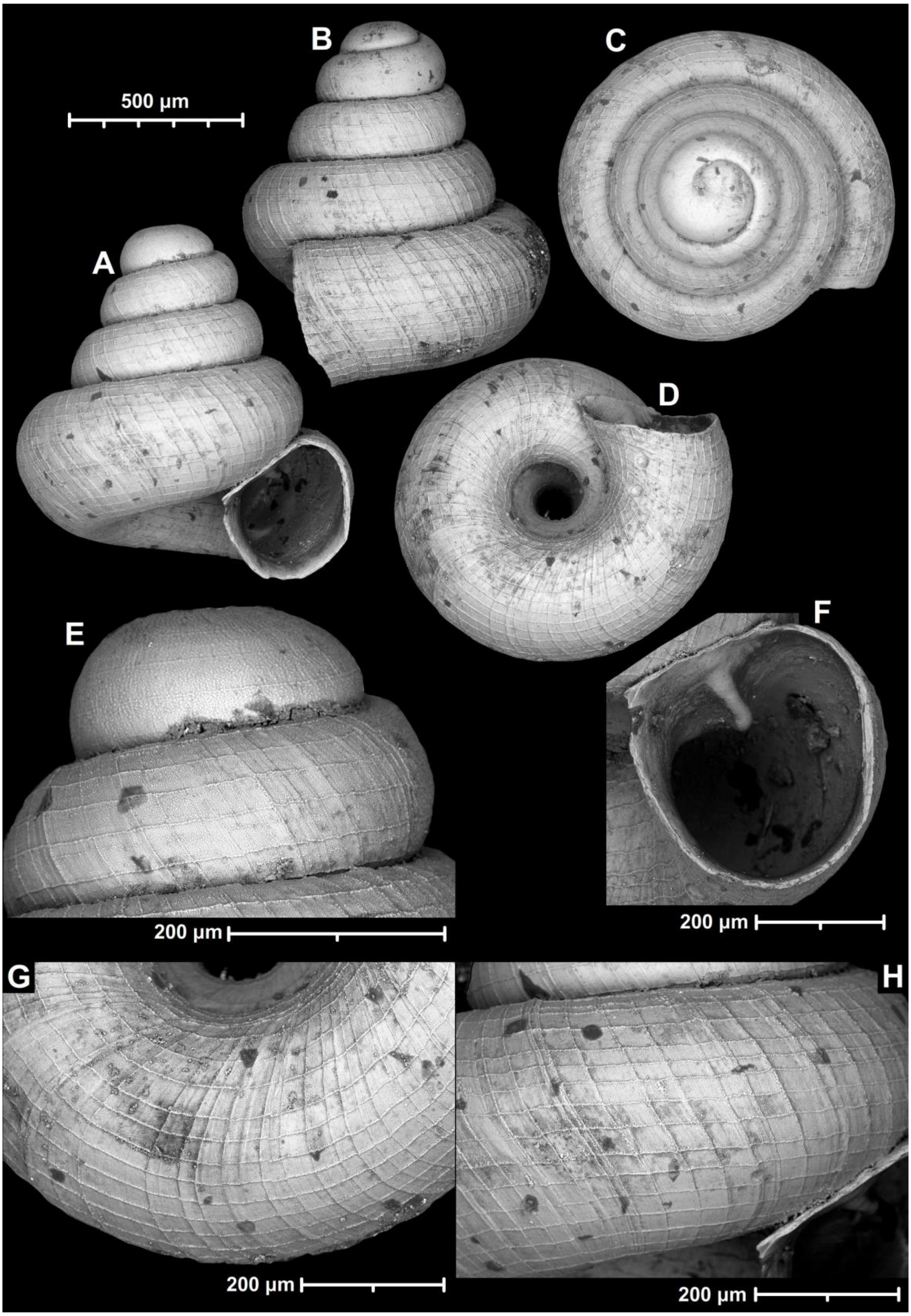

Supplementary Figure 26. *Angustopila fabella* Páll-Gergely & Hunyadi, 2015, JJV 6223, specimen1. Apertural (**A**), lateral (**B**), apical (**C**) and ventral (**D**) sides of the shell; microstructure of the protoconch (**E**) aperture (**F**), ventral (**G**) and frontal (**H**) surface of the body whorl.

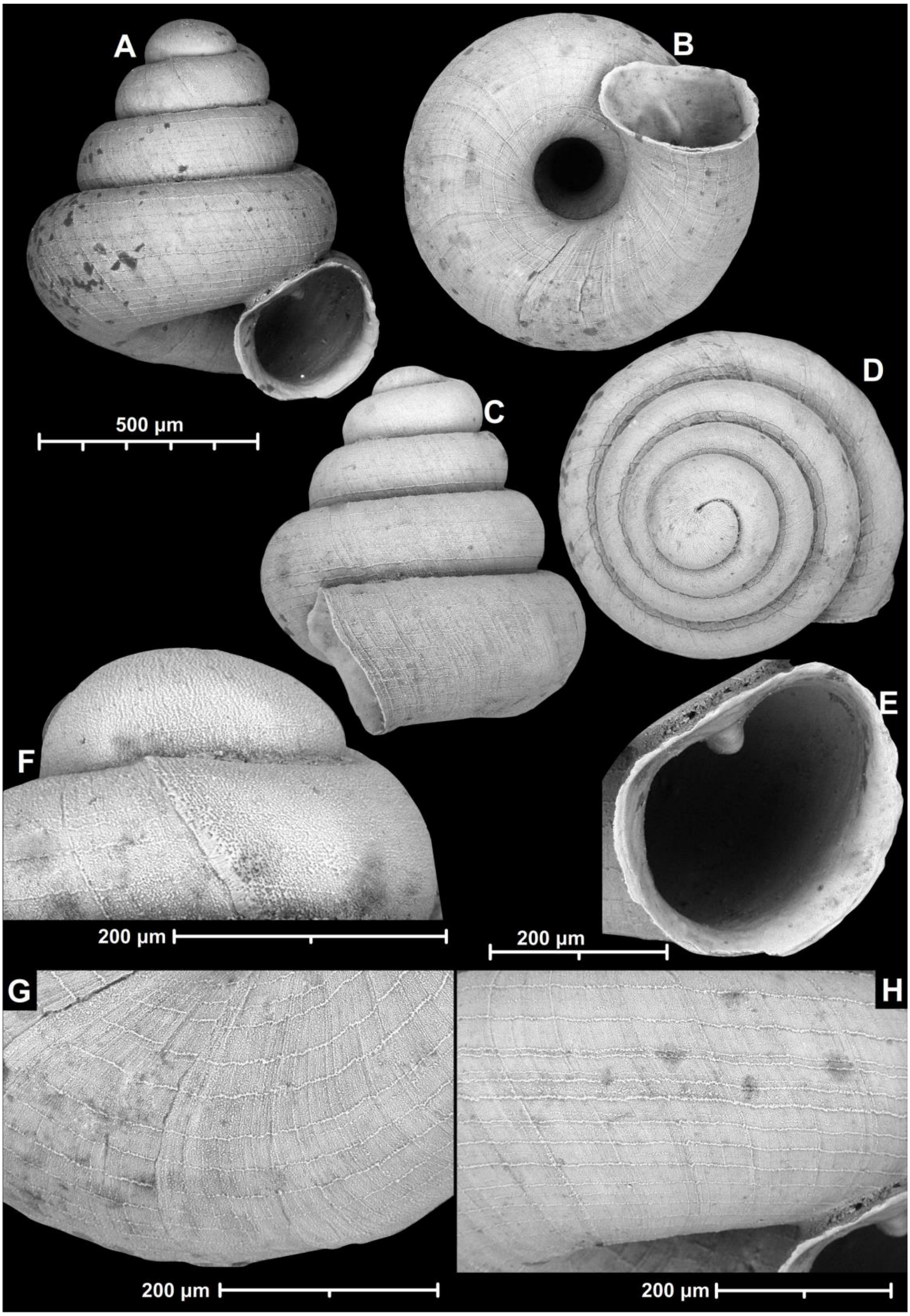

Supplementary Figure 27. *Angustopila fabella* Páll-Gergely & Hunyadi, 2015, sample La.11. Apertural (**A**), ventral (**B**), lateral (**C**) and apical (**D**) sides of the shell; aperture (**E**), microstructure of the protoconch showing protoconch-teleoconch boundary (**F**), ventral (**G**) and frontal (**H**) surface of the body whorl.

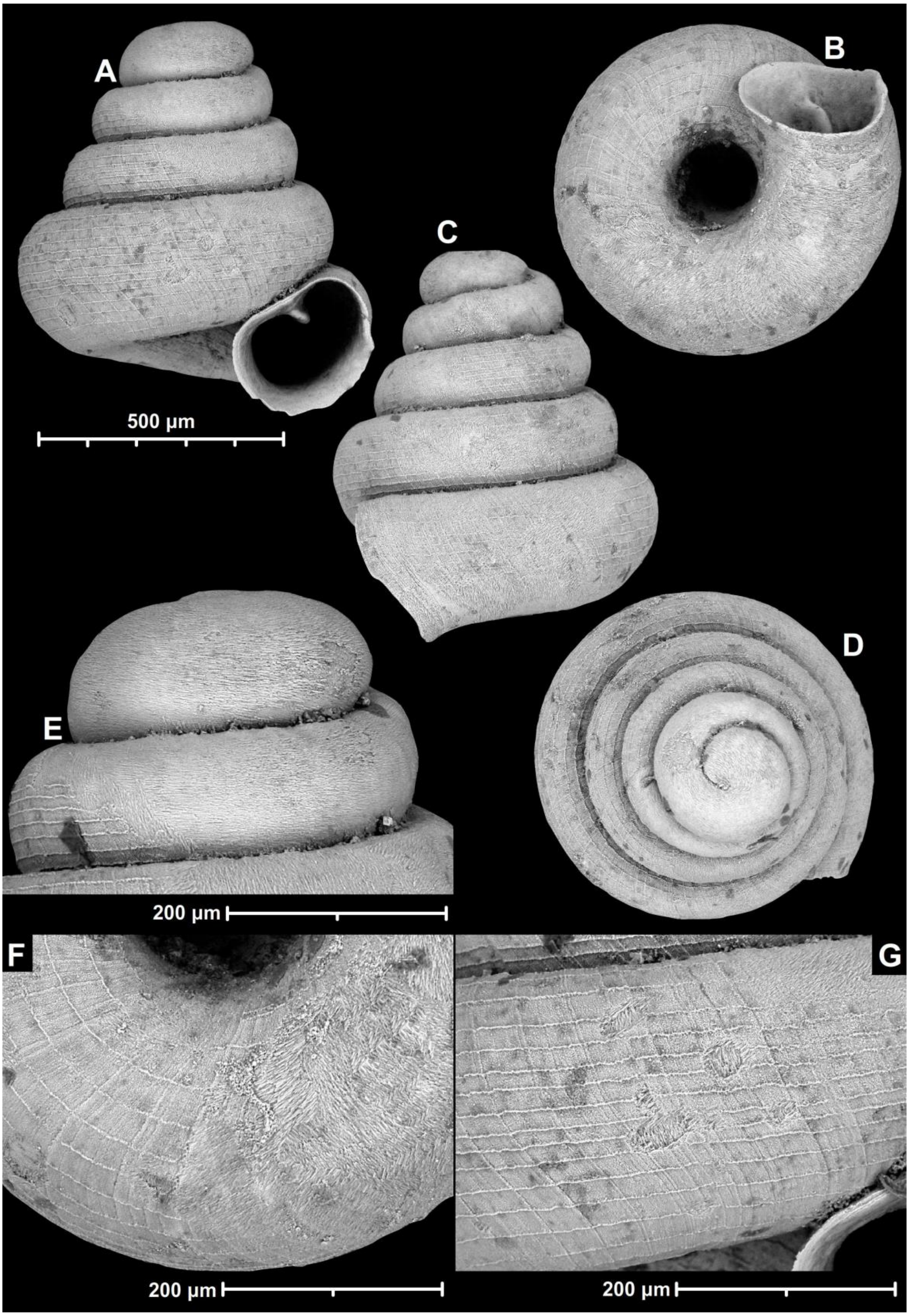

Supplementary Figure 28. *Angustopila fabella* Páll-Gergely & Hunyadi, 2015, sample JG26, specimen1. Apertural (**A**), ventral (**B**), lateral (**C**) and apical (**D**) sides of the shell; eroded protoconch surface showing underlying crossed lamellar microstructure (**E**), eroded ventral (**F**) and partially eroded frontal (**G**) surface of the body whorl.

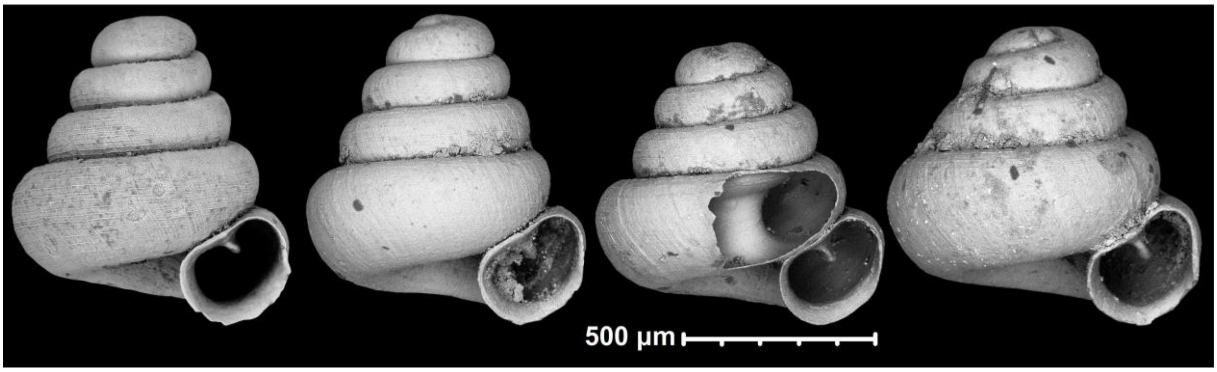

Supplementary Figure 29. Variability of *Angustopila fabella* Páll-Gergely & Hunyadi, 2015, sample JG26.

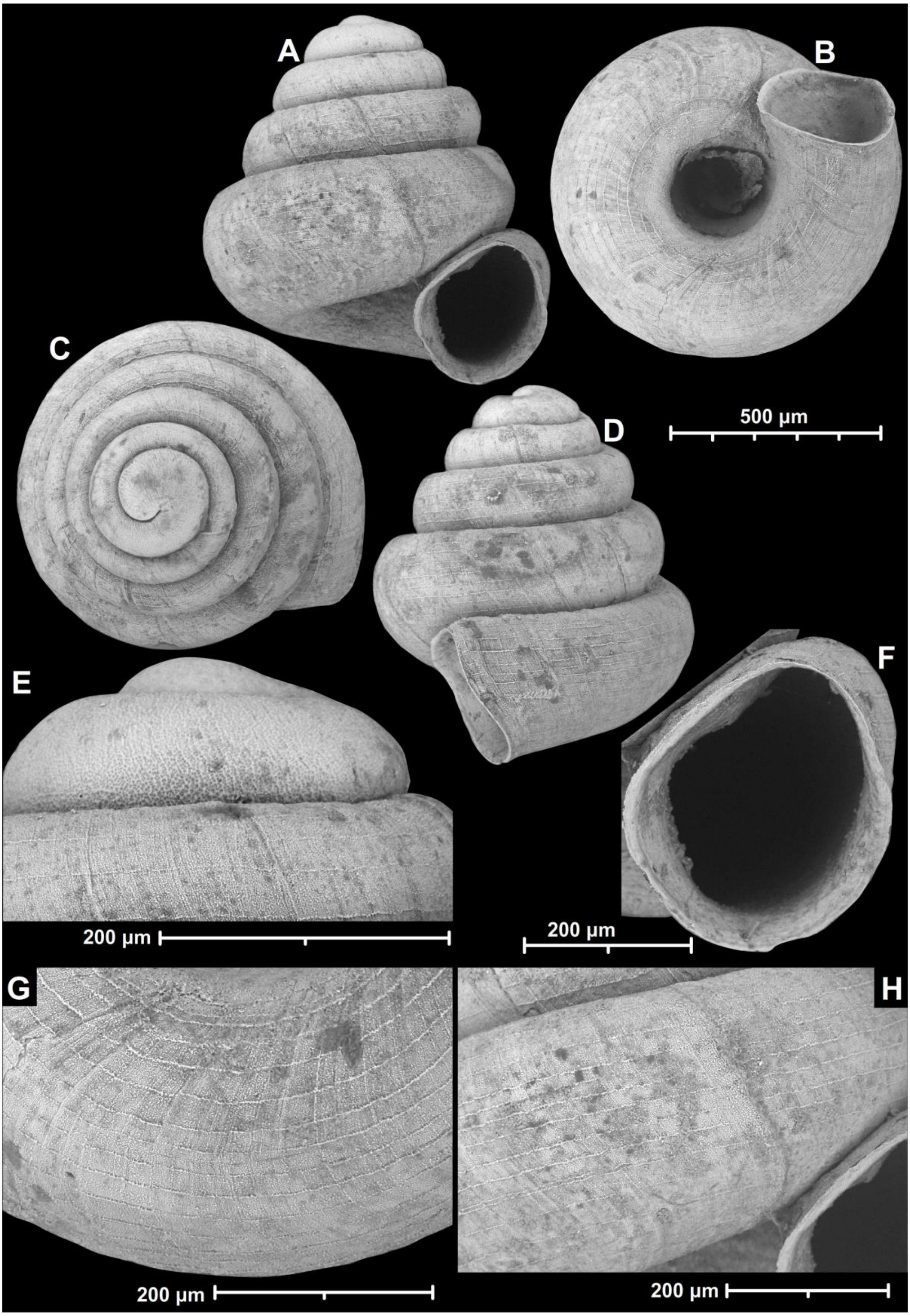

Supplementary Figure 30. *Angustopila* cf. *fratermajor* Páll-Gergely & Vermeulen, sp. n., sample WMVT.0344. Apertural (**A**), ventral (**B**), apical (**C**) and lateral (**D**) sides of the shell; aperture (**F**), microstructure of the protoconch (**E**), ventral (**G**) and frontal (**H**) surface of the body whorl.

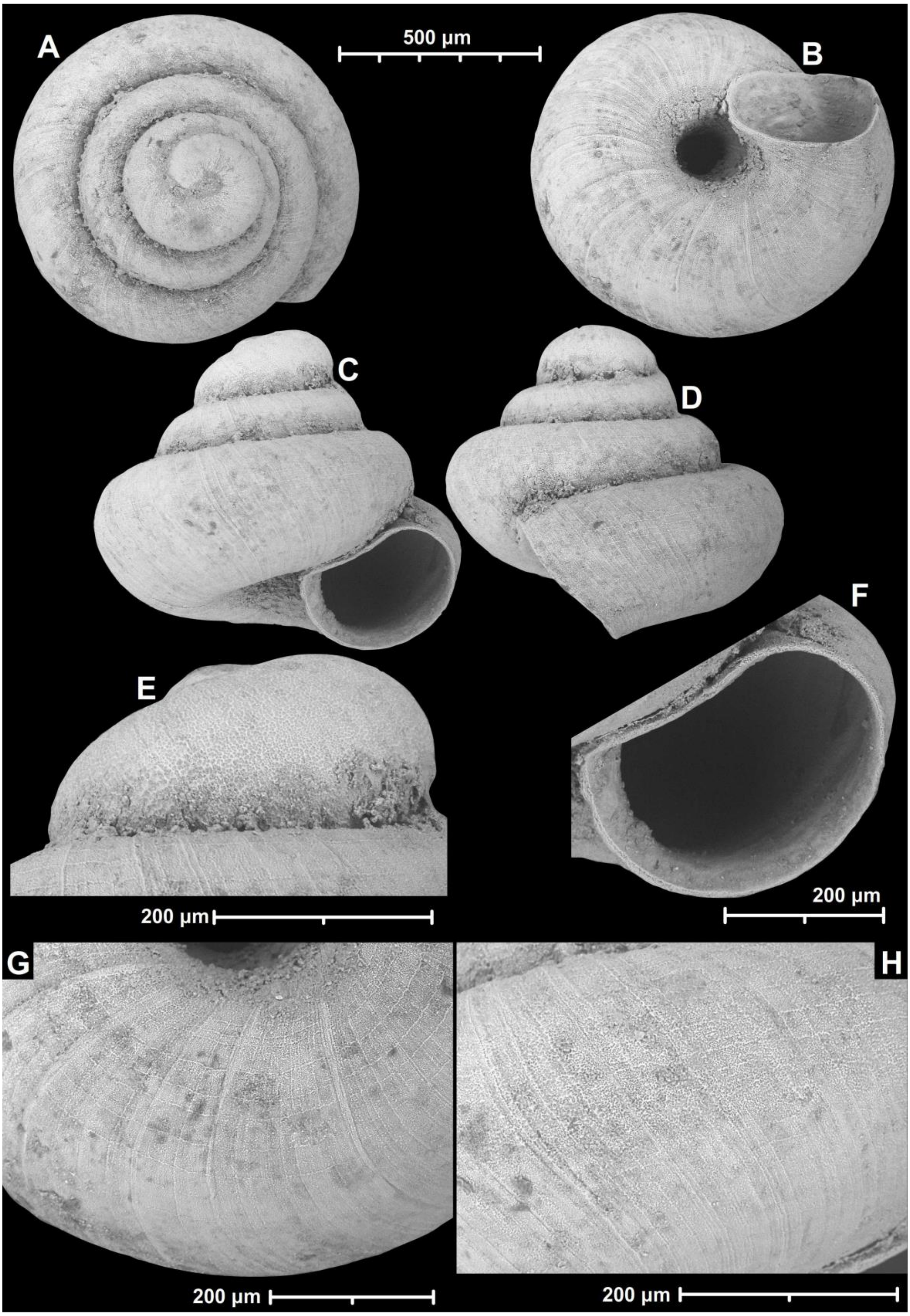

Supplementary Figure 31. *Angustopila megastoma* Páll-Gergely & Vermeulen, sp. n., (sample "Vietnam2", paratype: specimen1). Apical (**A**), ventral (**B**), apertural (**C**) and lateral (**D**) sides of the shell; aperture (**F**), sculpture on the protoconch (**E**), ventral (**G**) and frontal (**H**) surface of the body whorl.

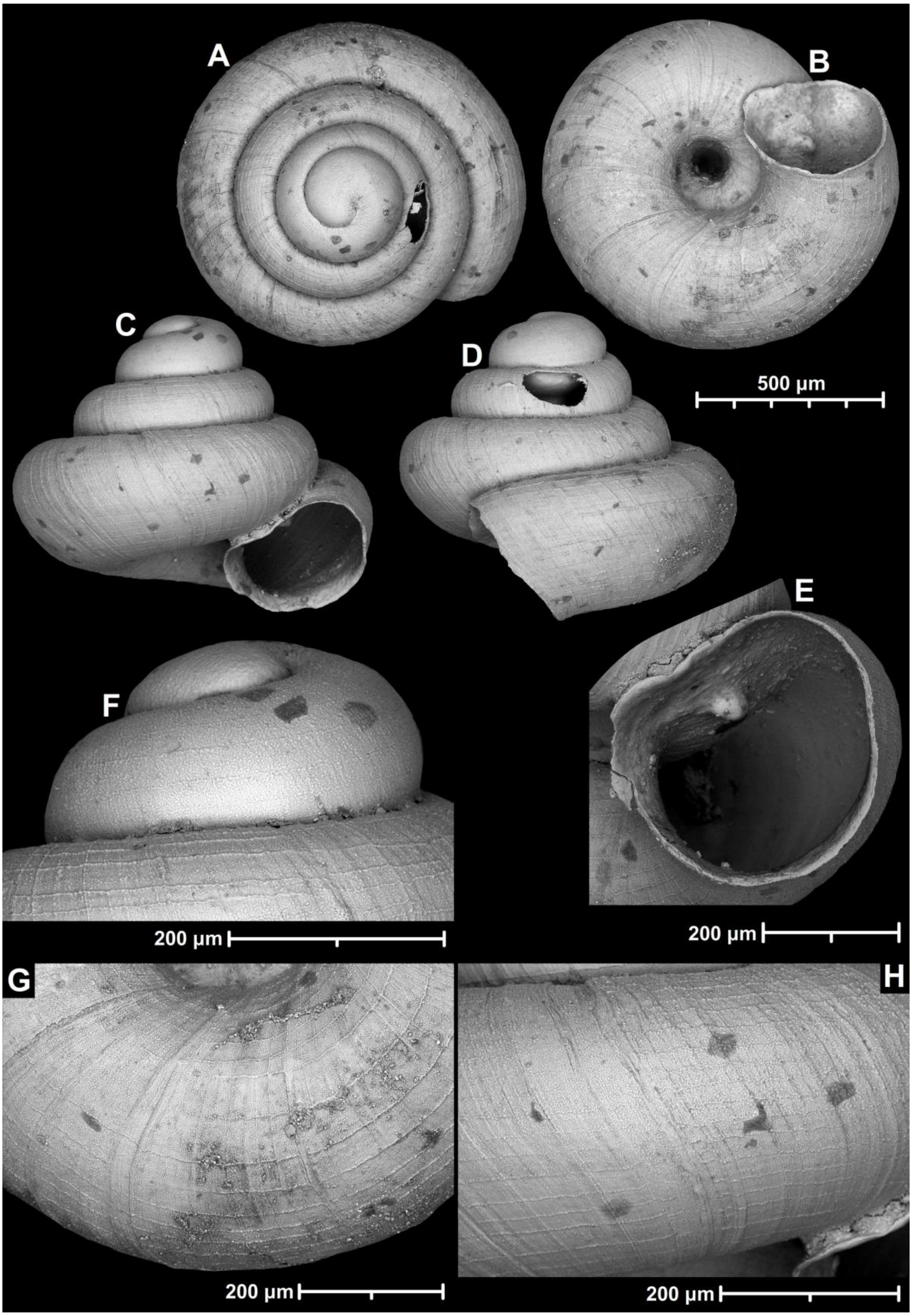

Supplementary Figure 32. *Angustopila megastoma* Páll-Gergely & Vermeulen, sp. n., sample JJV 6237, specimen1. Apical (**A**), ventral (**B**), apertural (**C**) and lateral (**D**), sides of the shell; aperture (**E**), sculpture on the protoconch (**F**), ventral (**G**) and frontal (**H**) surface of the body whorl.

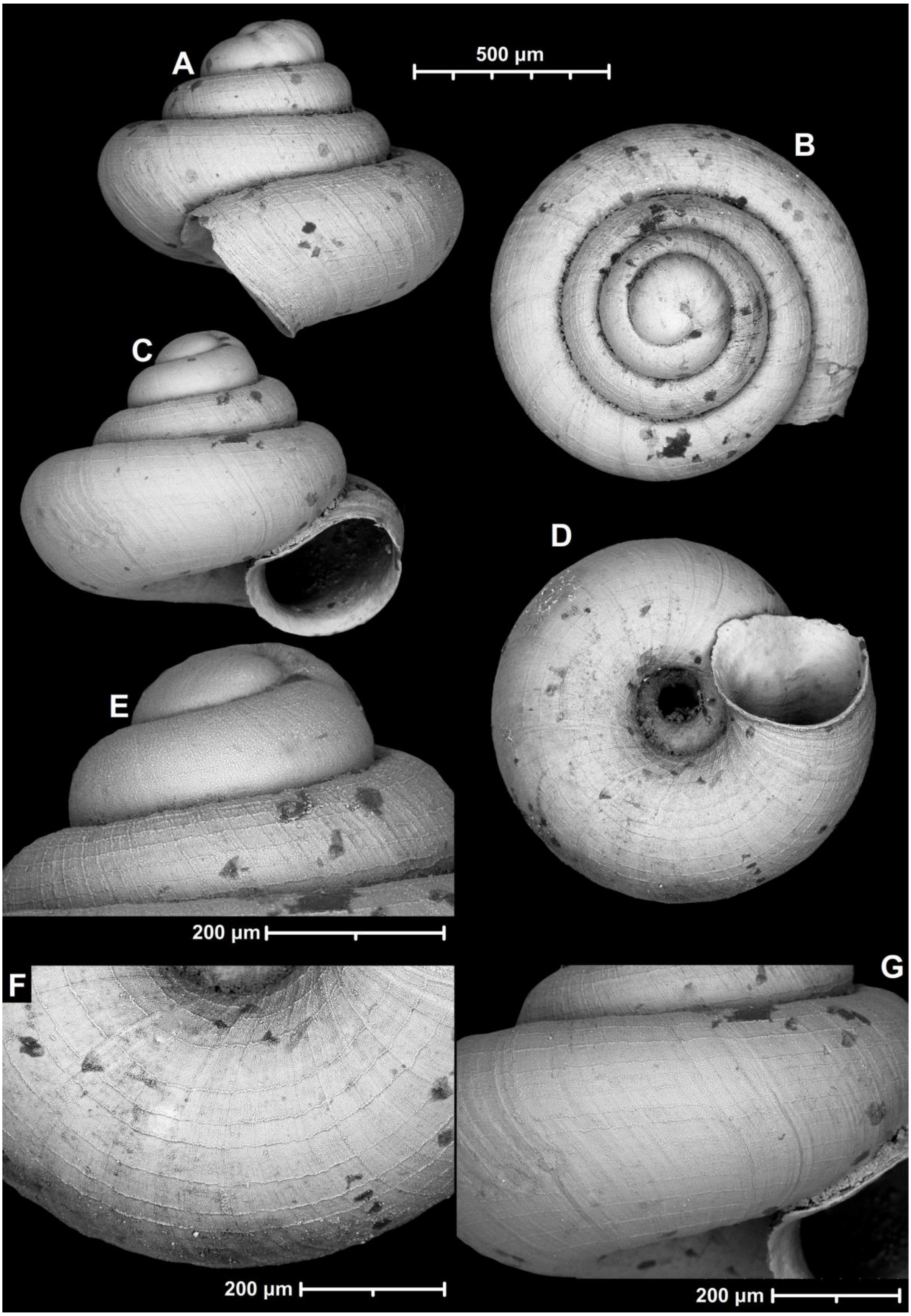

Supplementary Figure 33. *Angustopila megastoma* Páll-Gergely & Vermeulen, sp. n., sample JJV 6237, specimen3. Lateral (**A**), apical (**B**), apertural (**C**) and ventral (**D**) sides of the shell; sculpture of the protoconch (**E**), ventral (**F**) and frontal (**G**) surface of the body whorl.

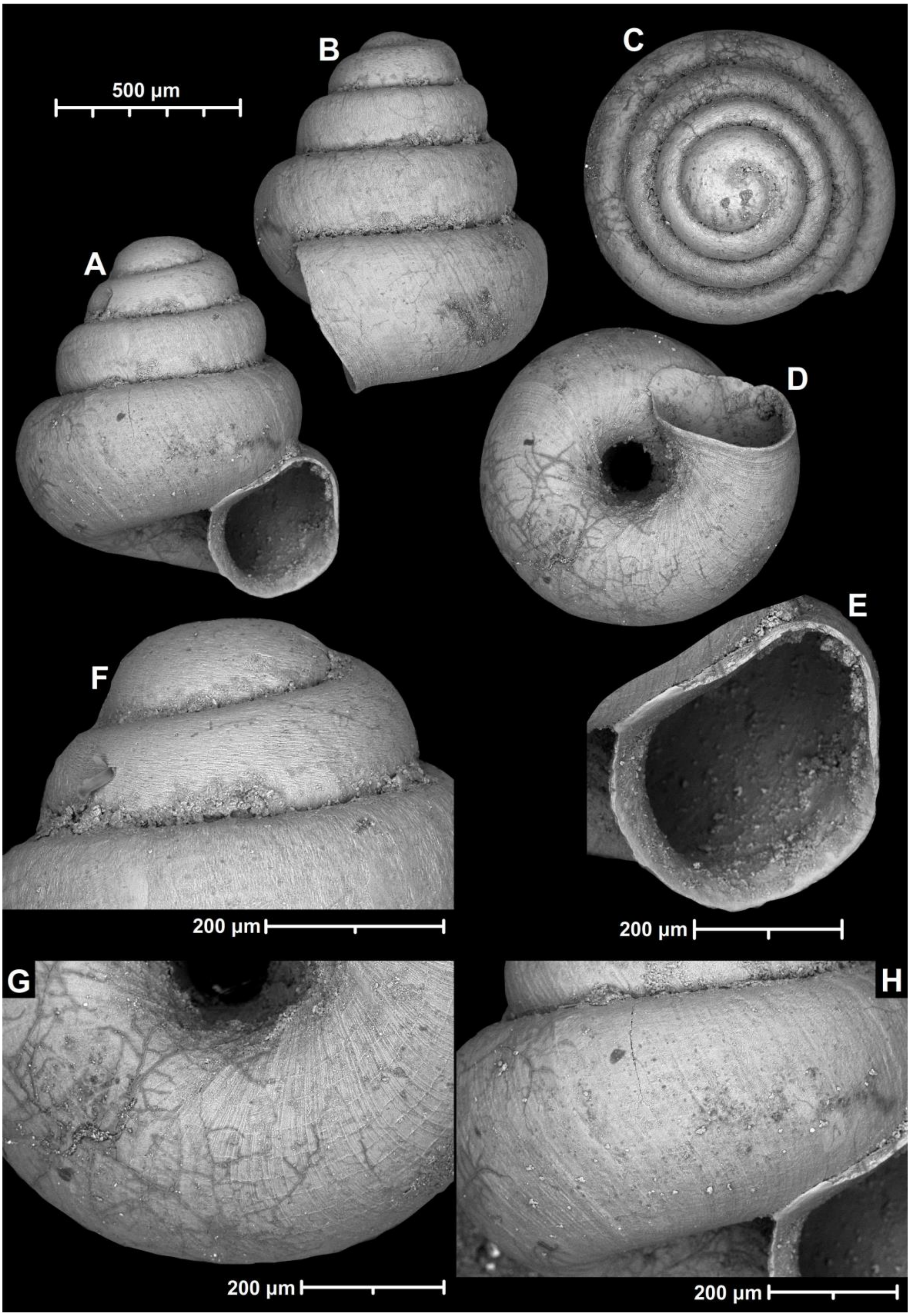

Supplementary Figure 34. *Angustopila* cf. *oostoma* Páll-Gergely & Vermeulen, sp. n., sample JJV 6252. Apertural (**A**), lateral (**B**), apical (**C**) and ventral (**D**) sides of the shell; aperture (**E**), eroded surface showing underlying crossed lamellar microstructure on the protoconch (**F**), ventral (**G**) and frontal (**H**) surface of the body whorl.

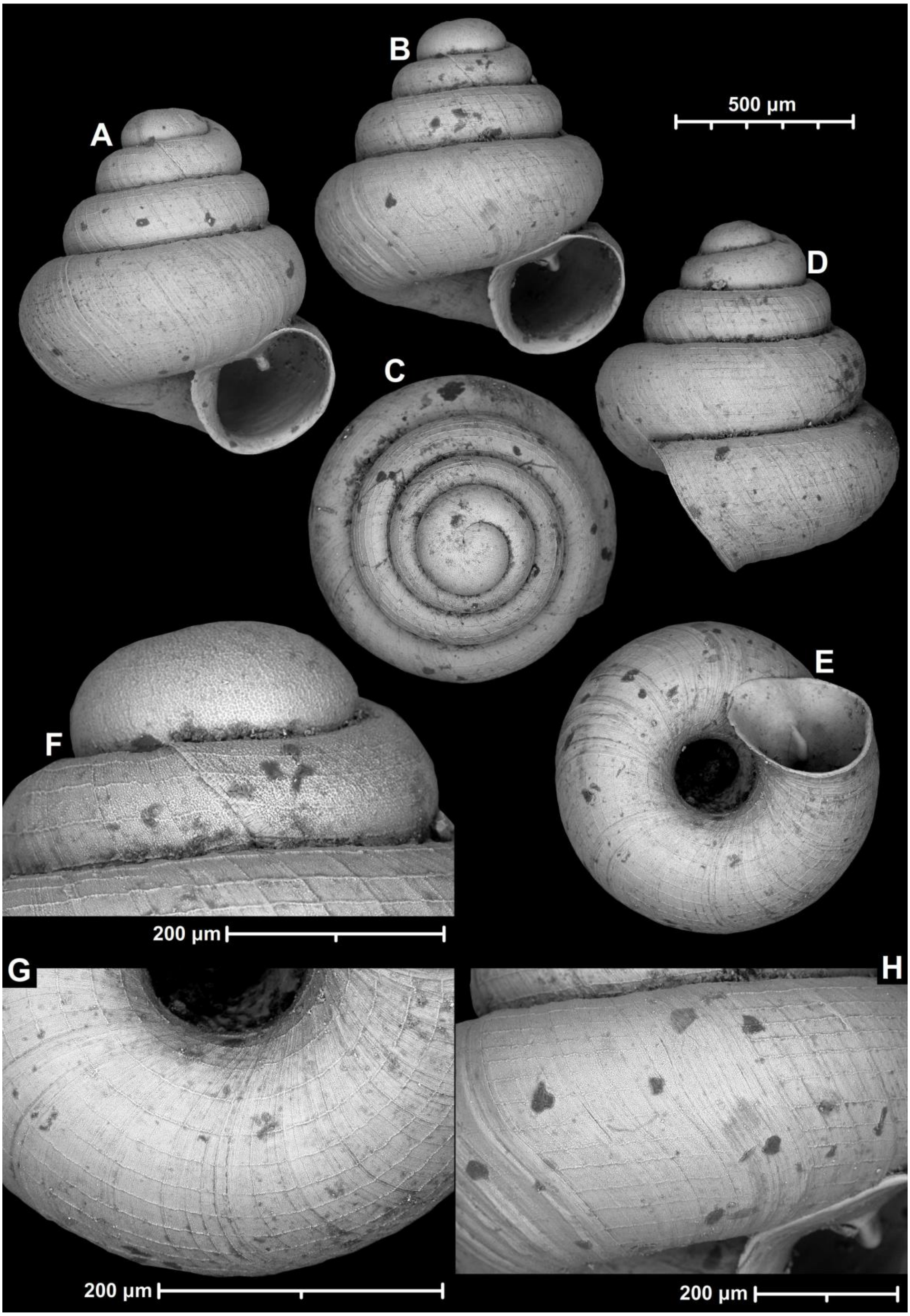

Supplementary Figure 35. *Angustopila szekeresi* Páll-Gergely & Hunyadi, 2015, sample 2019/118. Specimen2: **A**, Specimen1: **B**–**H**. Apertural (**A**–**B**), apical (**C**), lateral (**D**) and ventral (**E**) sides of the shell; microstructure of the protoconch (**F**), ventral (**G**) and frontal (**H**) surface of the body whorl.

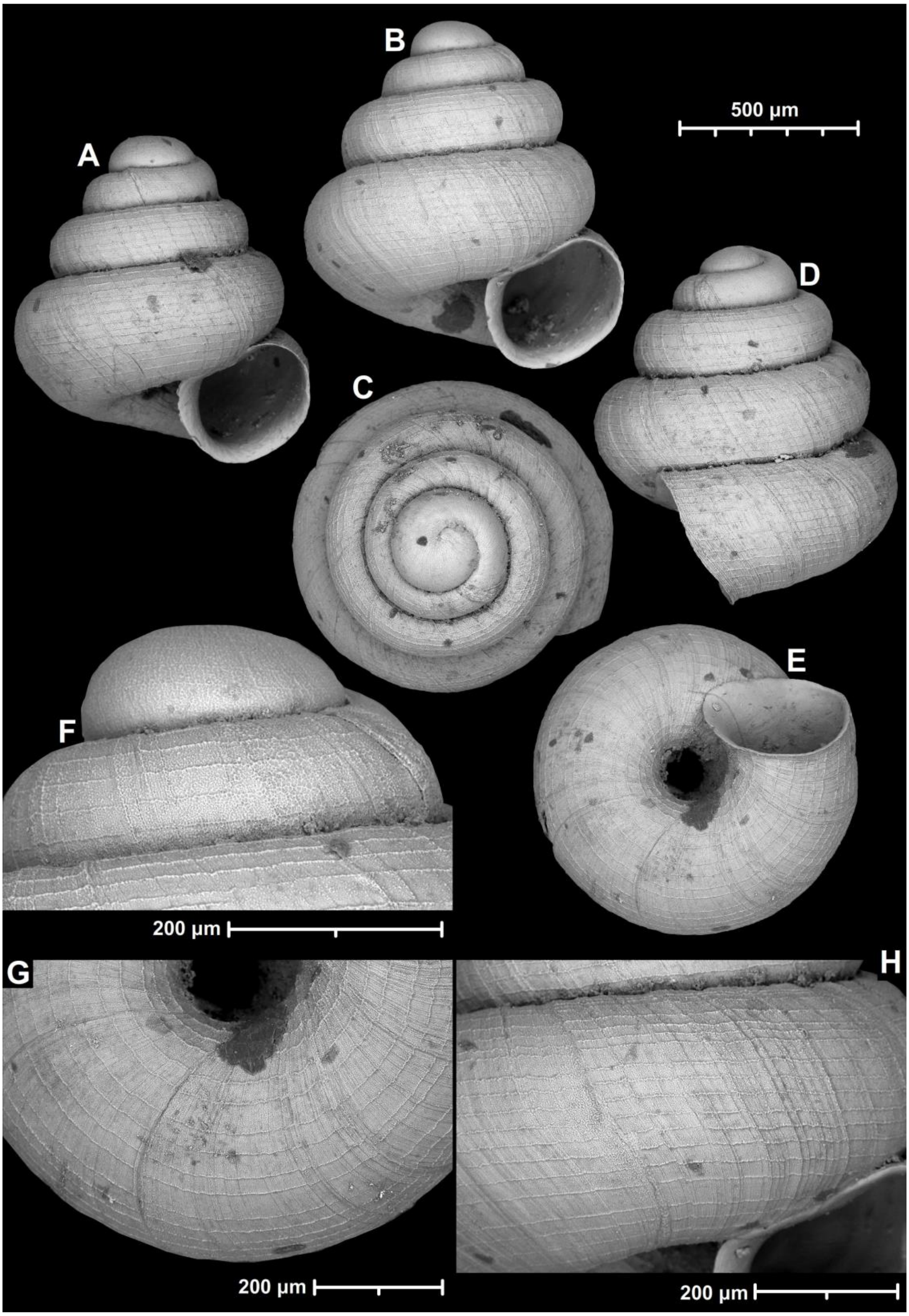

Supplementary Figure 36. *Angustopila szekeresi* Páll-Gergely & Hunyadi, 2015, sample 2020/32. Specimen2: **A**, Specimen1: **B–H**. Apertural (**A–B**), apical (**C**), lateral (**D**) and ventral (**E**) sides of the shell; microstructure of the protoconch (**F**), ventral (**G**) and frontal (**H**) surface of the body whorl.

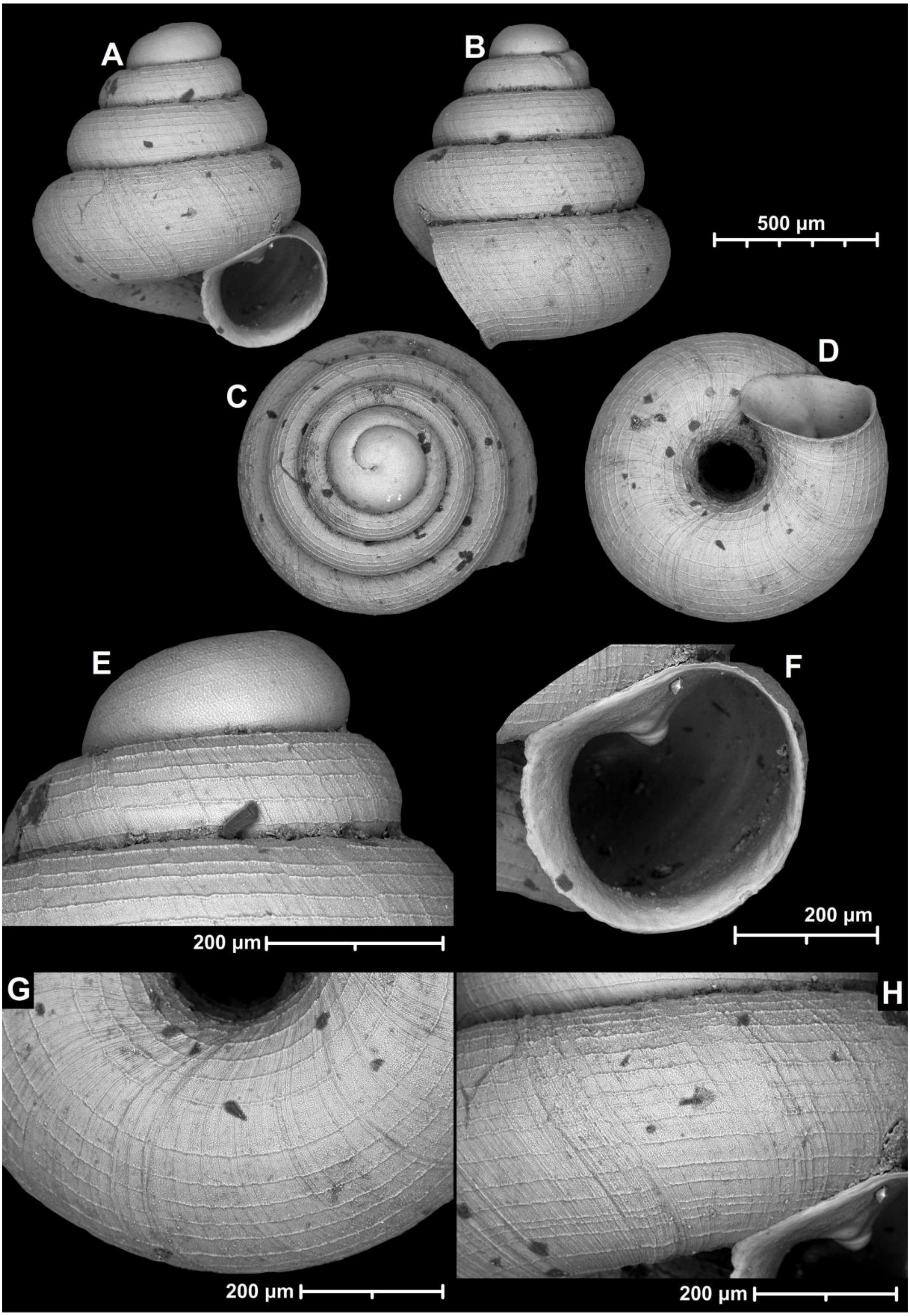

Supplementary Figure 37. *Angustopila szekeresi* Páll-Gergely & Hunyadi, 2015, sample 2020/41. Apertural (**A**), lateral (**B**), apical (**C**) and ventral (**D**) sides of the shell; microstructure of the protoconch (**E**), aperture (**F**), ventral (**G**) and frontal (**H**) surface of the body whorl.

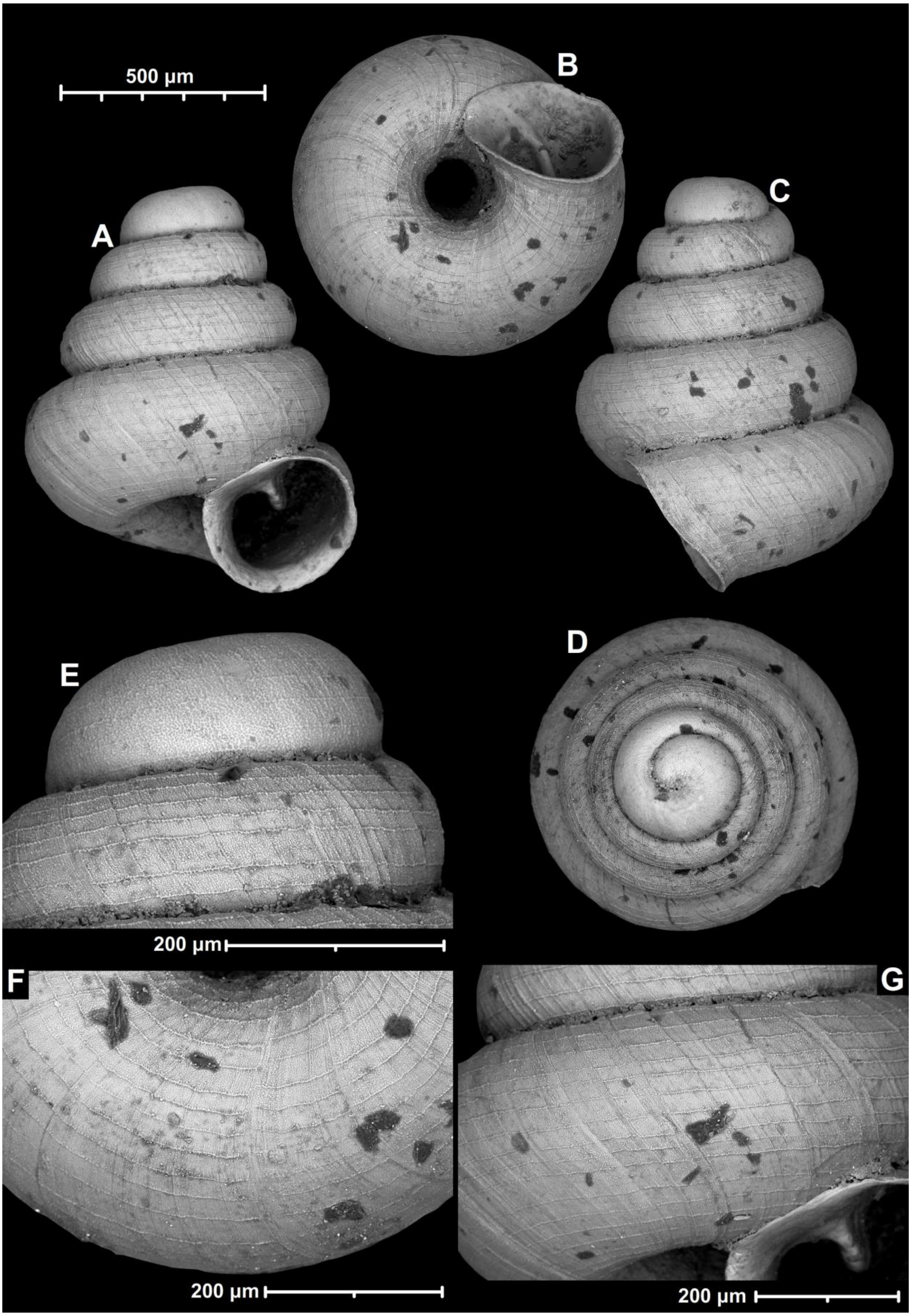

Supplementary Figure 38. *Angustopila* cf. *szekeresi* Páll-Gergely & Hunyadi, 2015, sample 2020/53. Apertural (**A**), ventral (**B**), lateral (**C**) and apical (**D**) sides of the shell; microstructure of the protoconch (**E**), ventral (**F**) and frontal (**G**) surface of the body whorl.

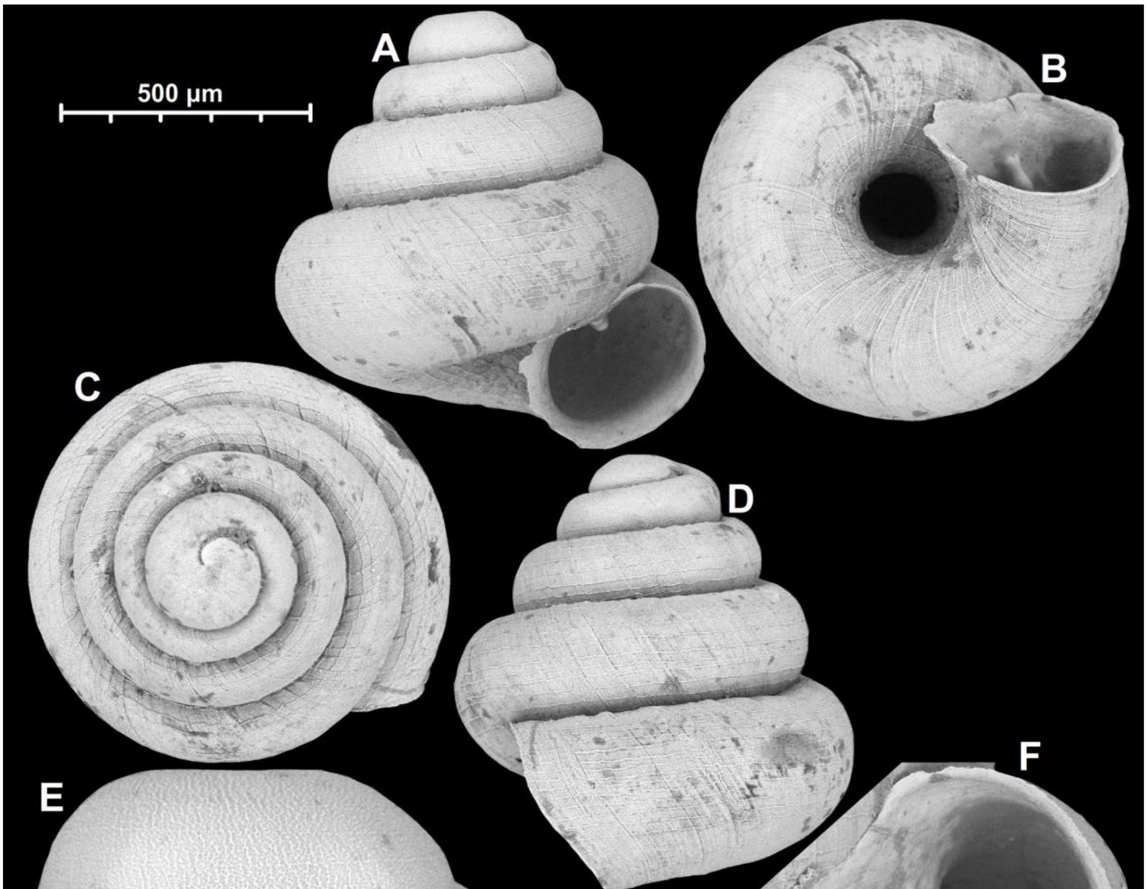

Supplementary Figure 39. *Angustopila szekeresi* Páll-Gergely & Hunyadi, 2015, sample WMVT.0344. Apertural (**A**), ventral (**B**), apical (**C**) and lateral (**D**) sides of the shell; microstructure of the protoconch showing protoconch-teleoconch boundary (**E**), aperture (**F**), ventral (**G**) and frontal (**H**) surface of the body whorl.

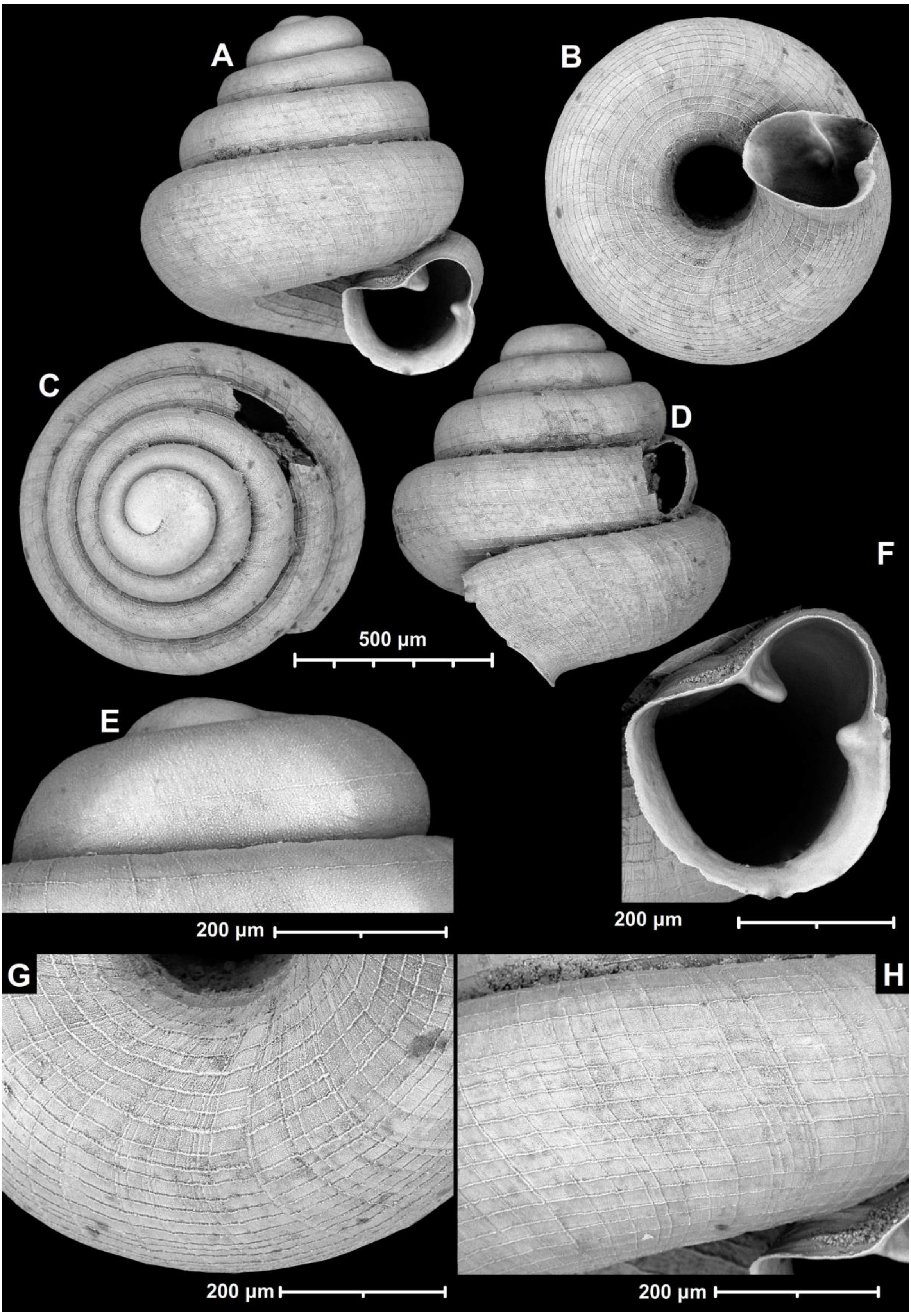

Supplementary Figure 40. *Angustopila bidentata* Páll-Gergely & Jochum, sp. n., sample 25L07, specimen1. Apertural (**A**), ventral (**B**), apical (**C**) and lateral (**D**) sides of the shell; aperture (**F**); sculpture on the protoconch (**E**), ventral (**G**) and frontal (**H**) surface of the body whorl.

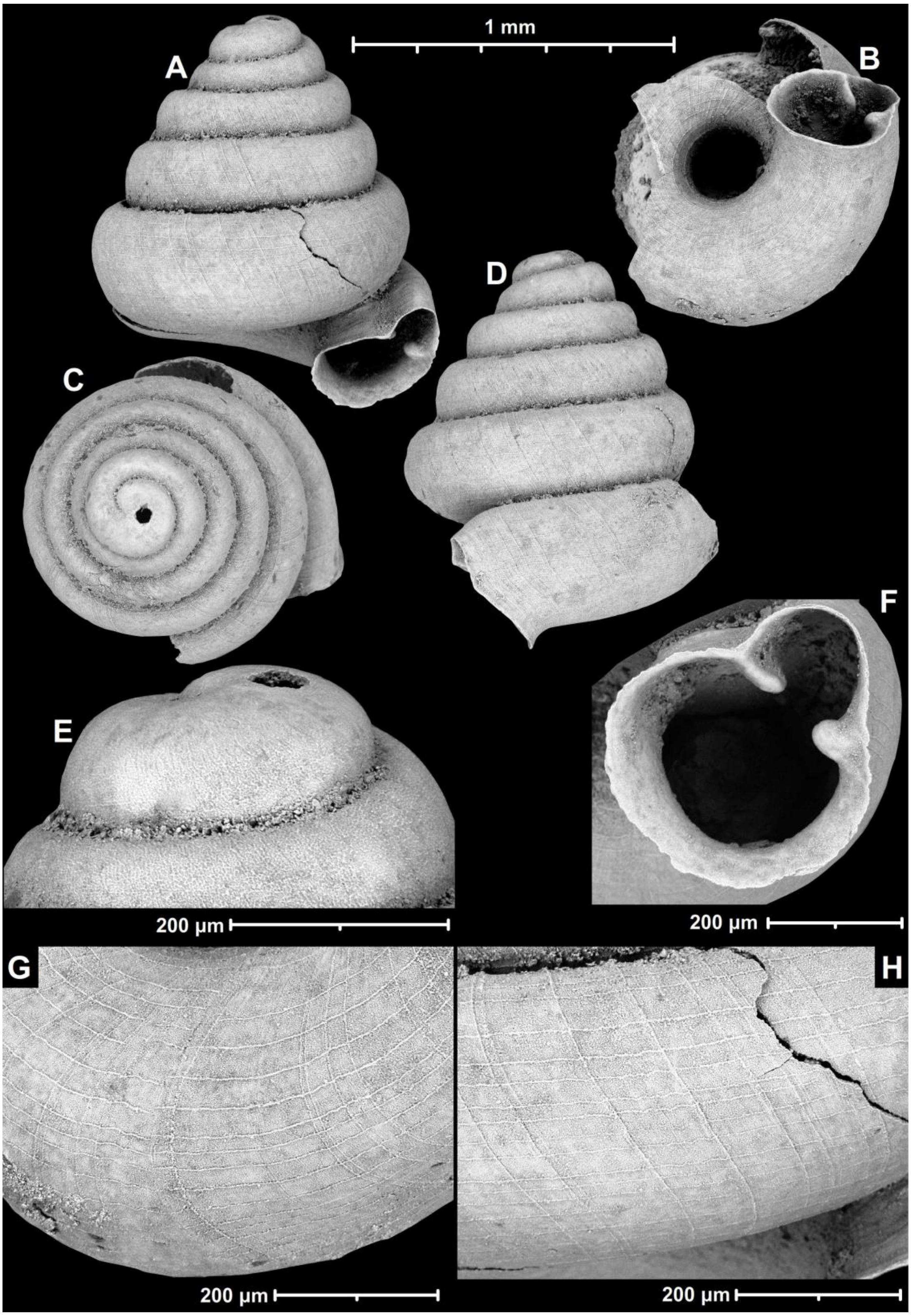

Supplementary Figure 41. *Angustopila bidentata* Páll-Gergely & Jochum, sp. n., sample JG2A. Apertural (**A**), ventral (**B**), apical (**C**) and lateral (**D**) sides of the shell; aperture (**F**); sculpture on the protoconch (**E**), ventral (**G**) and frontal (**H**) surface of the body whorl.

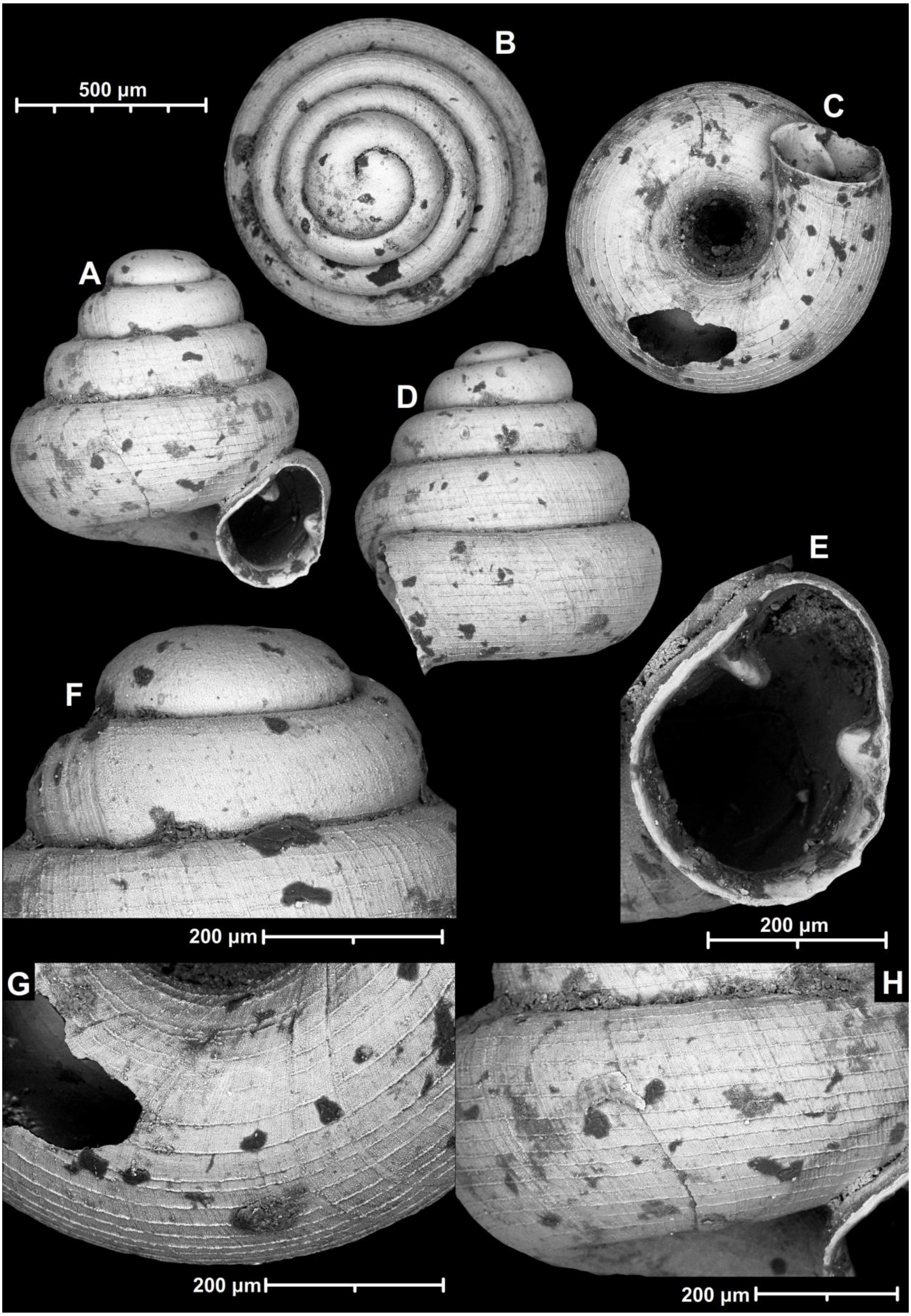

Supplementary Figure 42. *Angustopila* sp.1. Apertural (**A**), apical (**B**), ventral (**C**) and lateral (**D**) sides of the shell; aperture (**E**); sculpture on the protoconch (**F**), ventral (**G**) and frontal (**H**) surface of the body whorl.
